# Supplementary material for: Future trends of marine fish biomass distributions from the North Sea to the Barents Sea
Source: Nat Commun. 2024 Jul 5;15:5637. doi: 10.1038/s41467-024-49911-9 (PMC11224334; doi:10.1038/s41467-024-49911-9)
Supplement: Supplementary file 1 — Supplementary Information [file 41467_2024_49911_MOESM1_ESM.pdf]

SUPPLEMENTARY INFORMATION

FIGURES

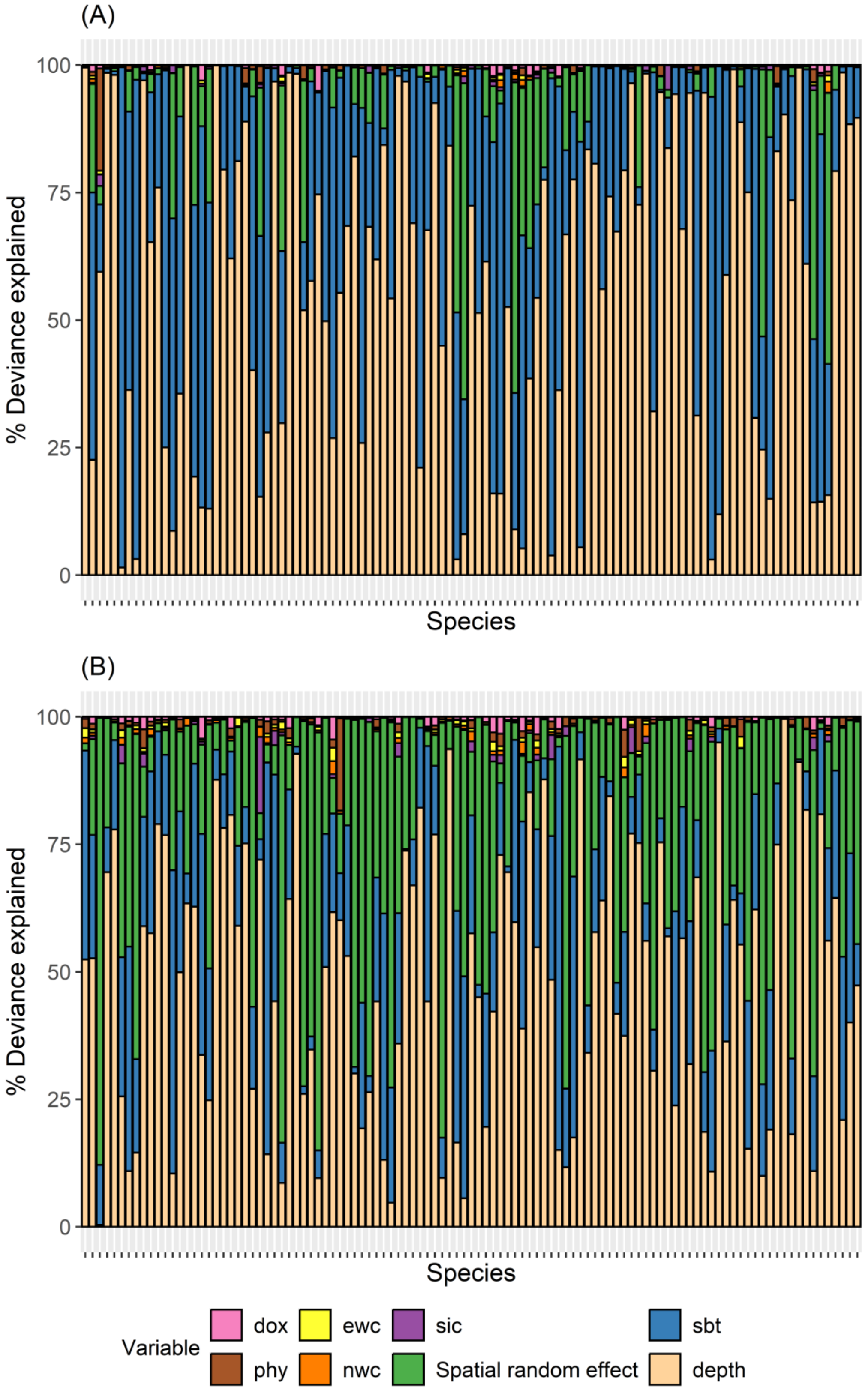

Supplementary Figure 1. Variance partitioning of (A) presence-absence model, and (B) biomass model, both components of the Hurdle model.

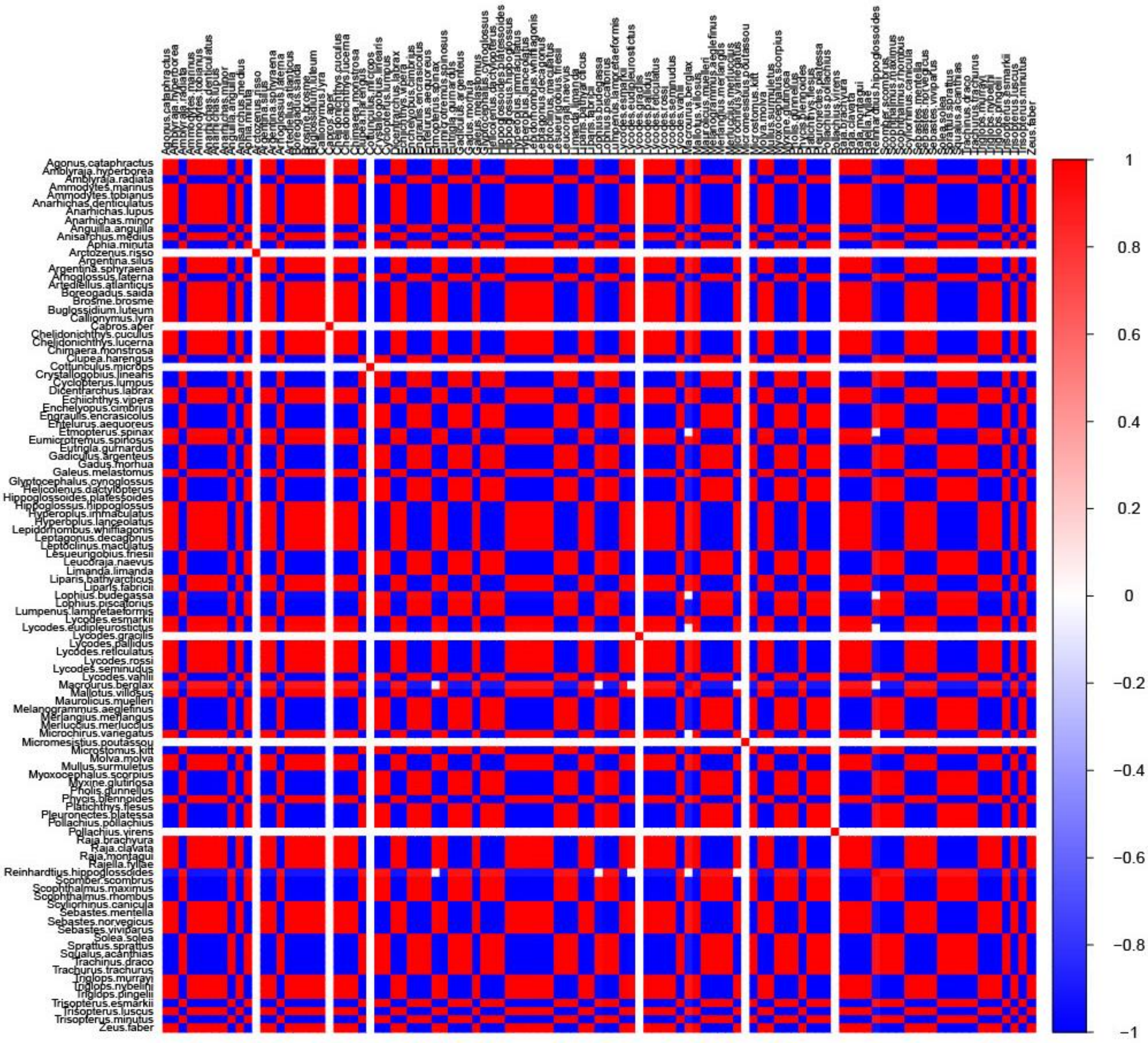

Supplementary Figure 2. Species co-occurrence patterns of the occurrence model.

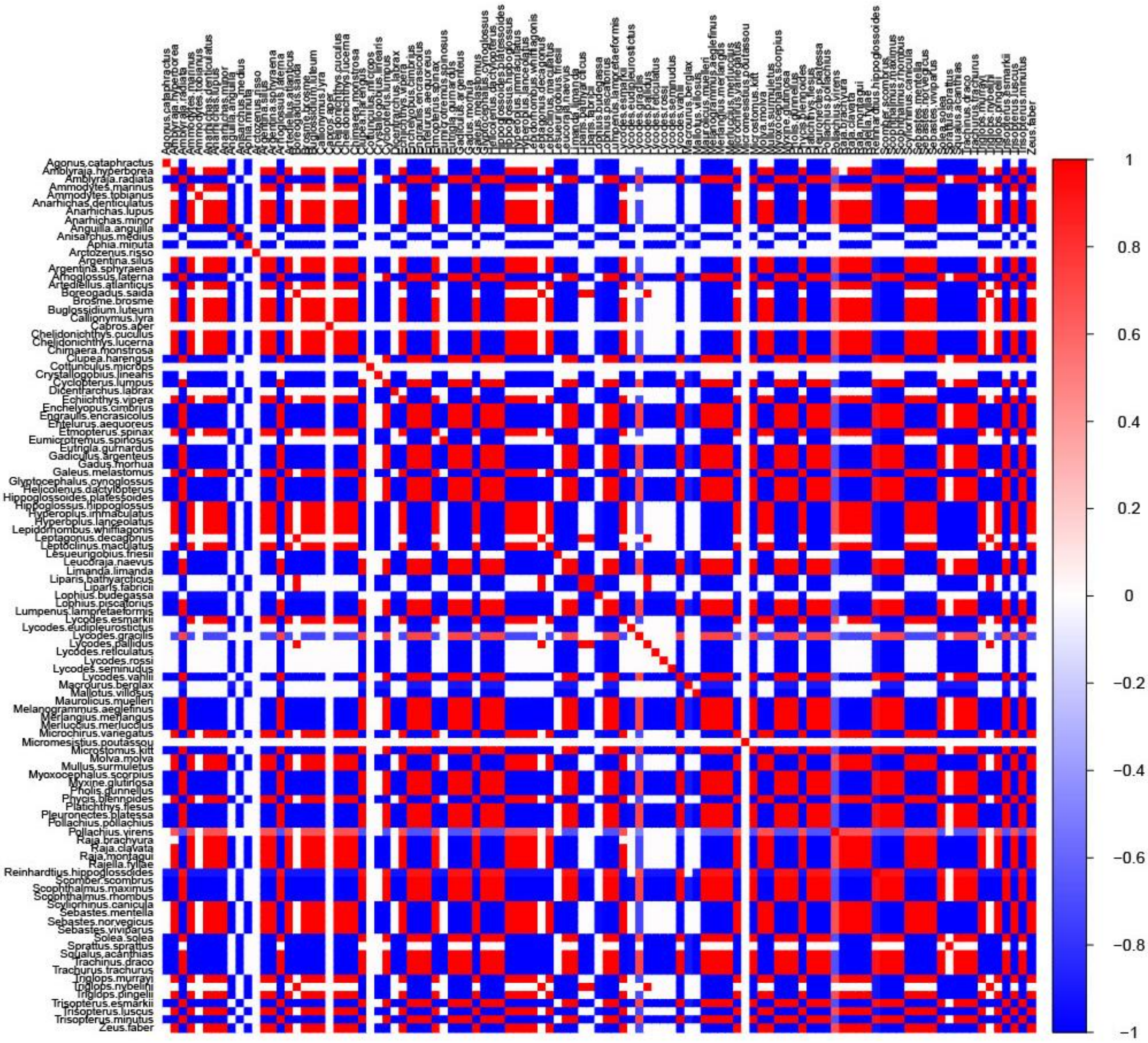

Supplementary Figure 3. Species co-occurrence patterns of the occurrence model.

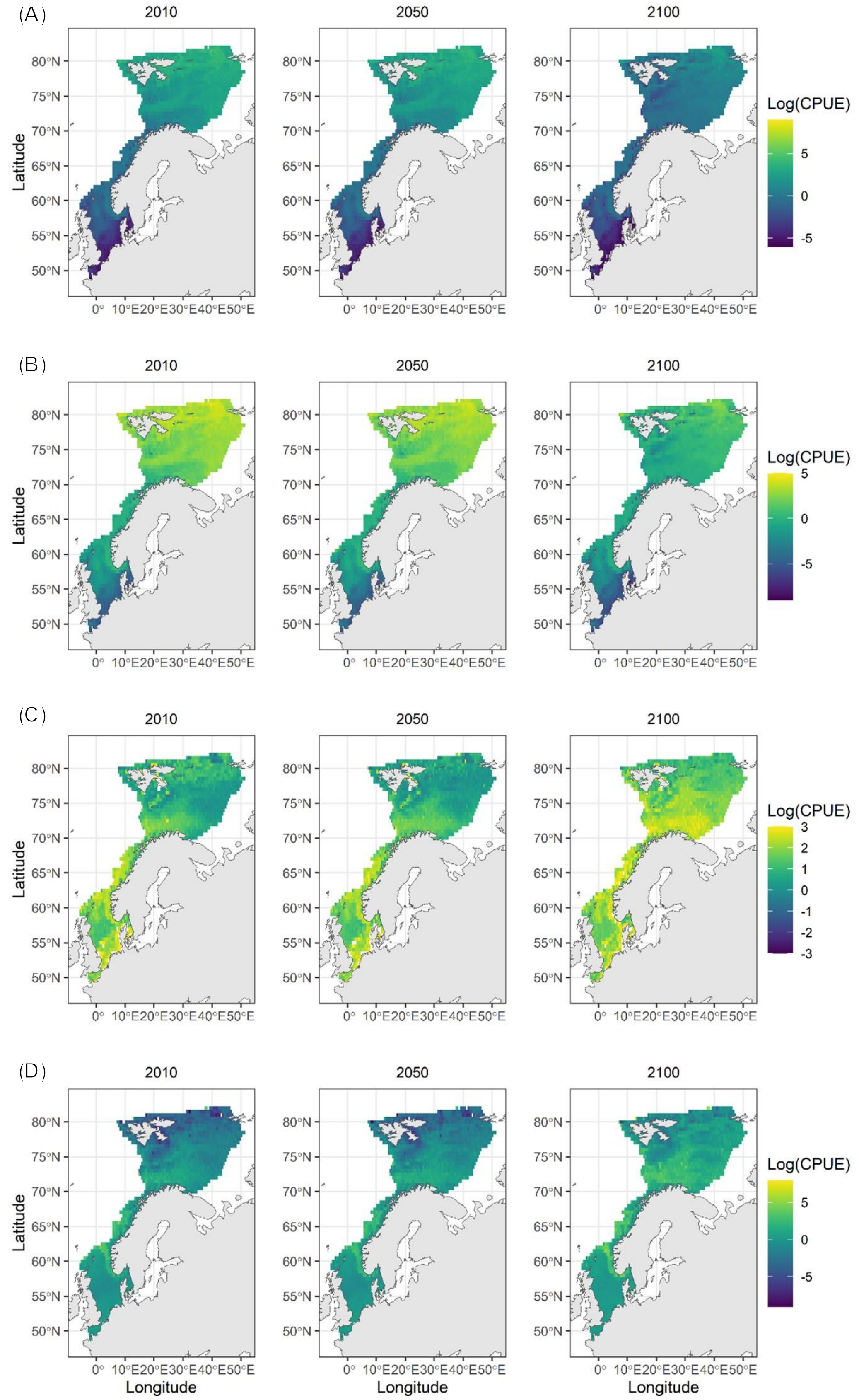

Supplementary Figure 4. Mean future biomass projections under SSP1-2.6 for (A) Arctic (n = 3), (B) Arctic-Boreal (n = 8), (C) Boreal (n = 40), and (D) Temperate and Subtropical (n = 5) species. Log CPUE corresponds to log of catch per unit effort (fish/min).

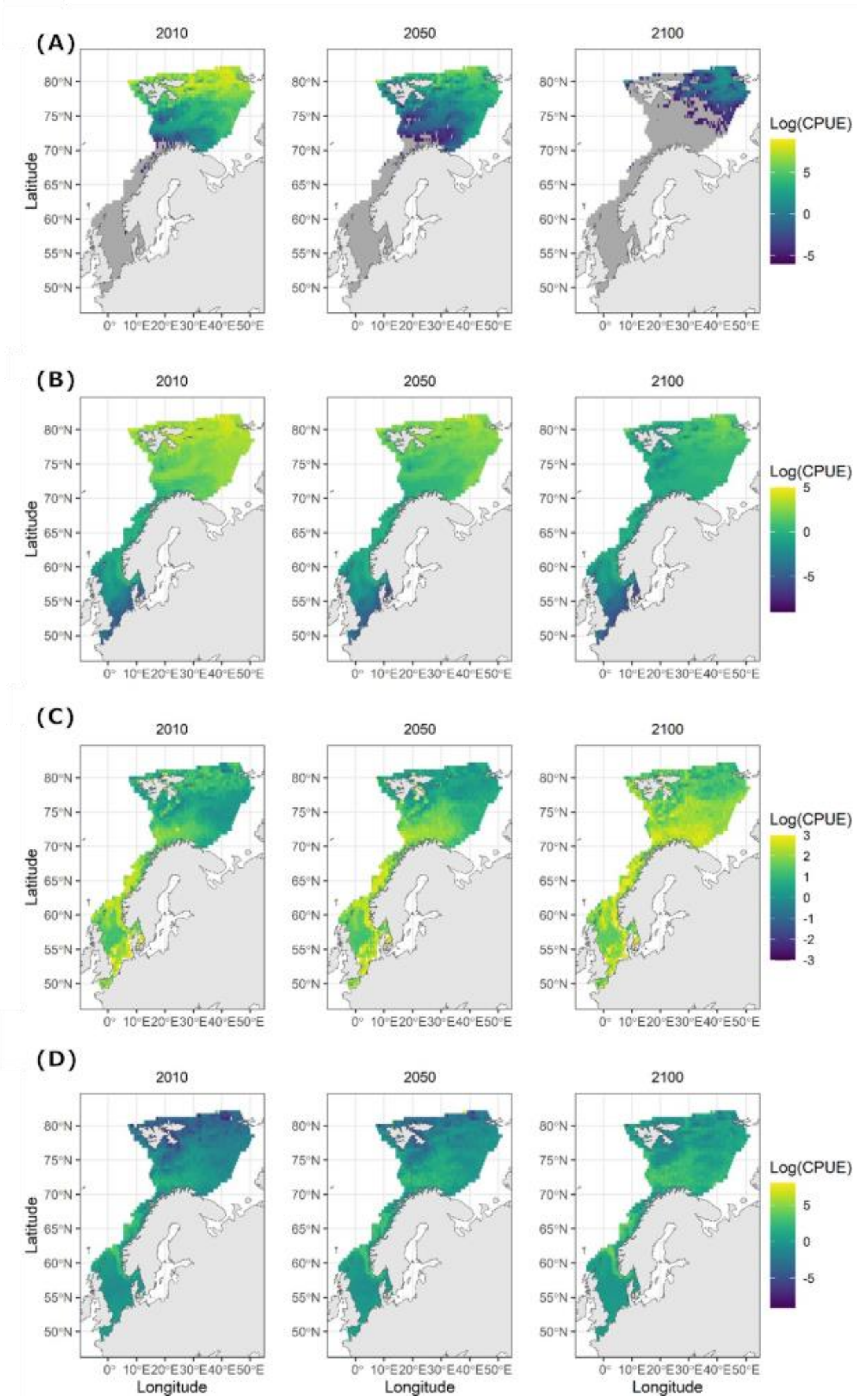

Supplementary Figure 5. Mean future biomass projections under SSP2-4.5 for (A) Arctic (n = 3), (B) Arctic-Boreal (n = 8), (C) Boreal (n = 40), and (D) Temperate and Subtropical (n = 5) species. Log CPUE corresponds to log of catch per unit effort (fish/min).

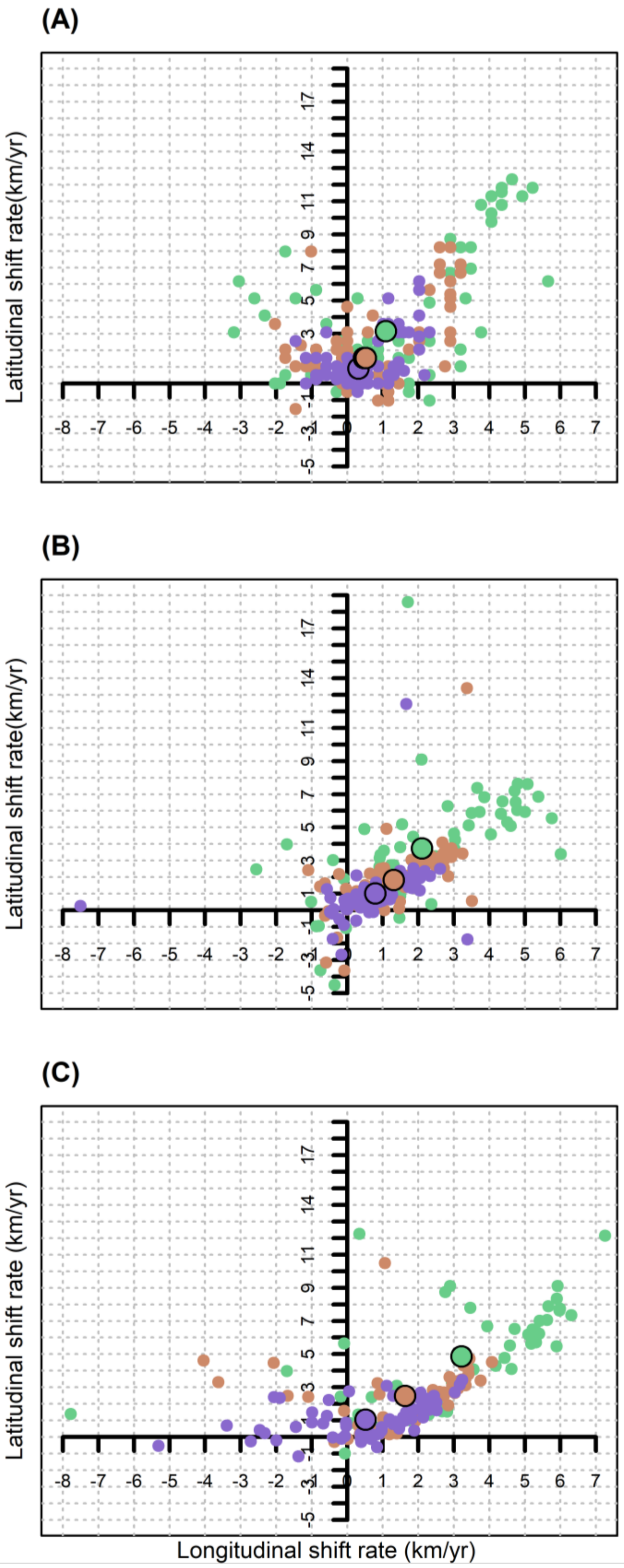

Supplementary Figure 6. Projected shift in distribution (km/yr) between 2010 and 2100 of (A) geographic range centroid (n = 107 species); (B) Biomass weighted mean geographic range centroid (n = 61 species), and (C) core range biomass weighted mean geographic centroid (61 species). Bigger points show the each climate scenario mean shift, weighted by the kernel density estimate (purple, SSP1 – 2.6; orange, SSP2 – 4.5; green, SSP5 – 8.5).

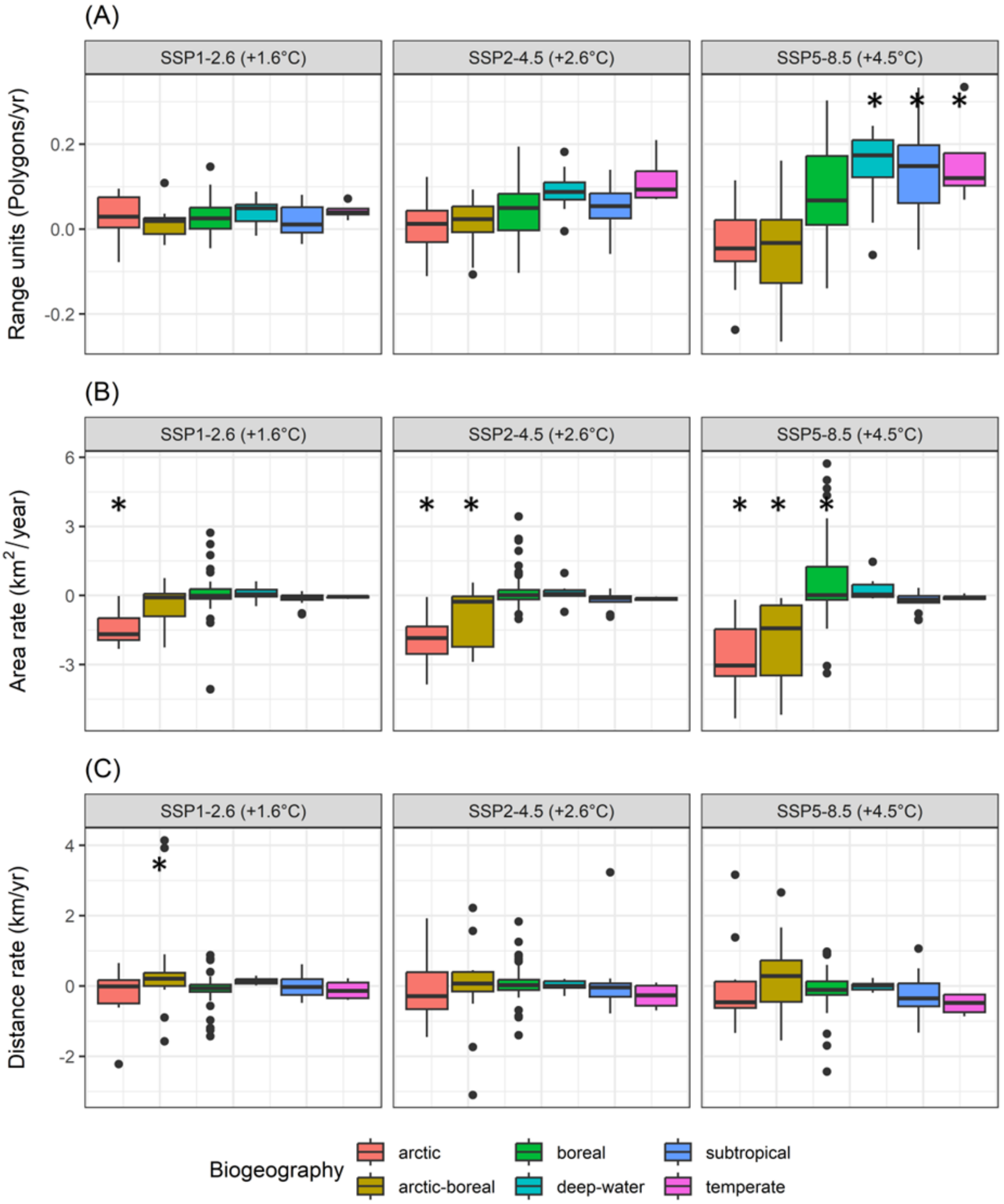

Supplementary Figure 7. Geographic range fragmentation change per zoogeograhpic class and shared socioeconomic pathway from 2010 to 2100. (A) Change in number of geographic range units; (B) change in mean area per geographic range unit, and (C) change in distance between geographic range units. Geographic range units are the individual polygons that compose a species' projected range. Whiskers correspond to maximum and minimum values, calculated as Q3 + 1.5xIQR and Q1 – 1.5xIQR, and points correspond to outliers. indicate significant effect of zoogeographic class (multiple linear regression $p < 0.05$).Sample sizes (arctic: n=11; arctic-boreal n = 13; boreal n = 57; deep-water n = 8, subtropical n = 14; temperate n = 4).

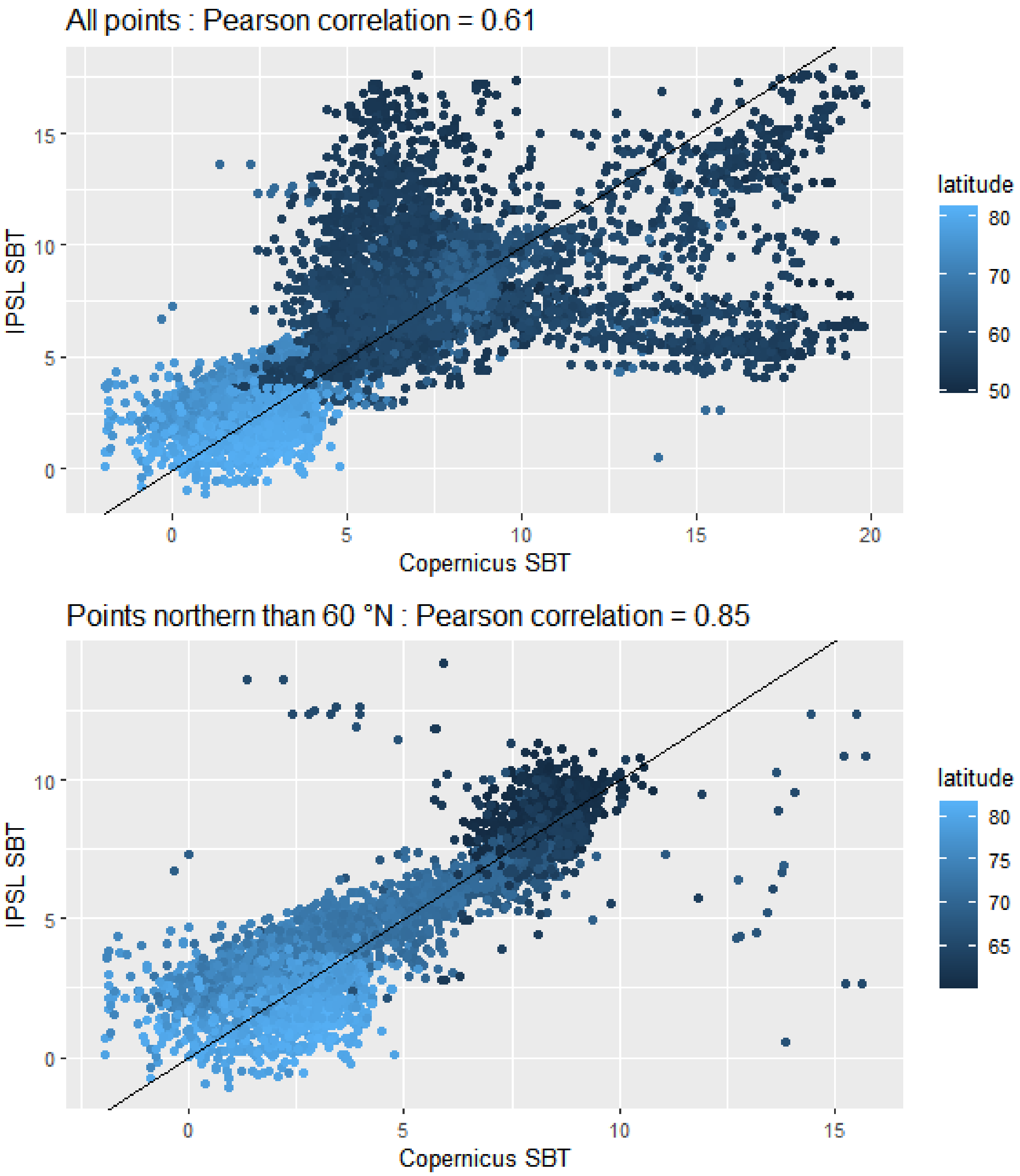

Supplementary Figure 8. Correlation analysis between monthly SBT from the copernicus analysis forecast, and the historical run of the CMIP6-IPSL global earth system model, for each of the sampling points between 2004 and 2014 (CMIP6-IPSL historical run only runs up to 2014)**,** with and without the North Sea (top and bottom respectively).

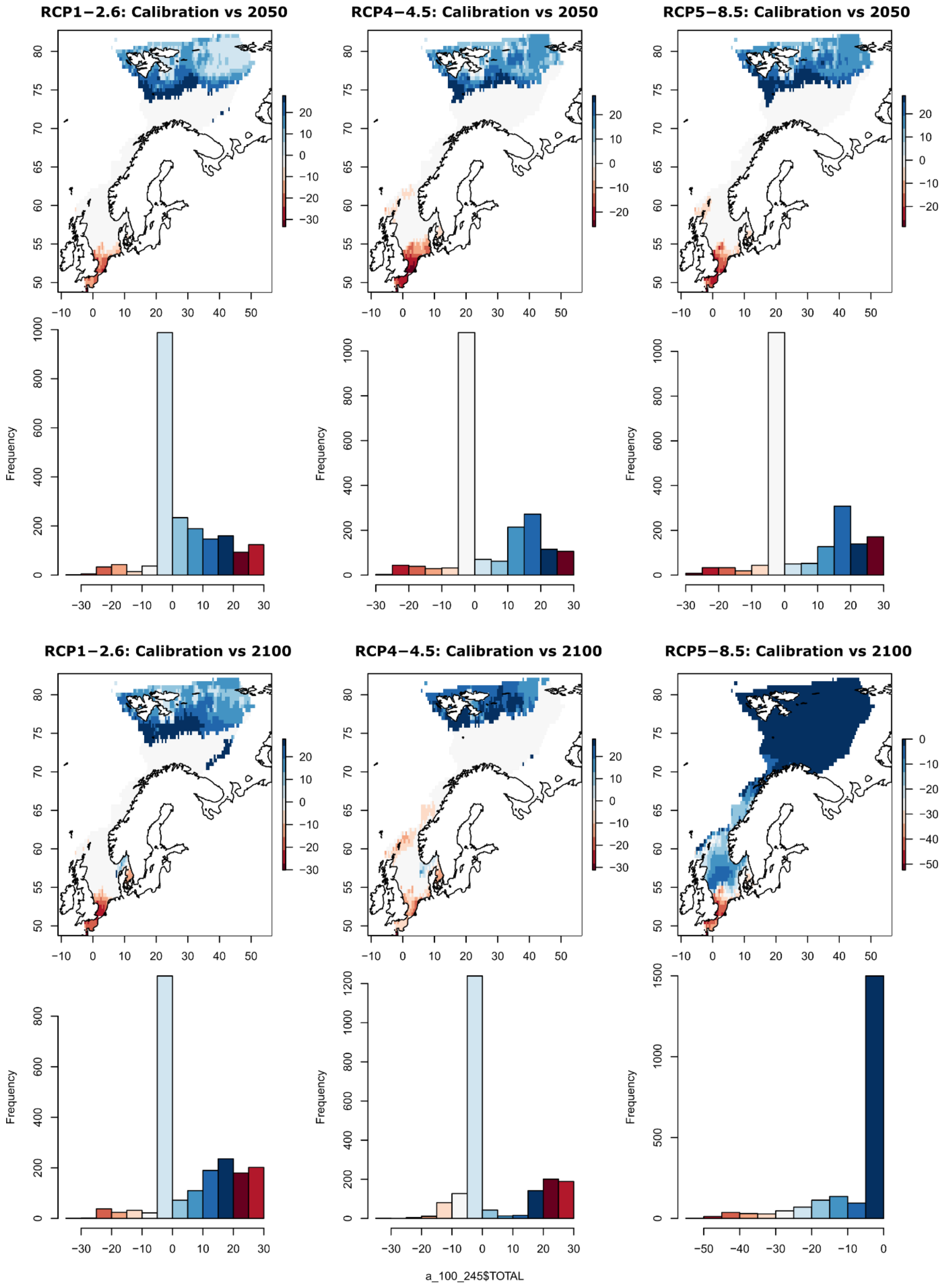

Supplementary Figure 9. MESS analysis including all environmental variables and comparing training data to 2050, and 2100 predictive data, in each of the three emission scenarios. Strongly negative values represent those sections of the environmental space poorly sampled during model calibration, and therefore projections in those areas are less reliable.

TABLES

Supplementary Table 1. Explanatory variables considered in fittng the Joint Species Distribution Models (JSDMs) and projecting them in the future.

Model fitting			
**Variable**	**Units**	**Resolution**	**Source**
**Bathymetry**	m	0.08 °	Bio Oracle
**Bottom temperature**	°C	0.08 °	Global Ocean Physics Reanalysis
**Bottom dissolved oxygen**	mmol/m3	0.25 °	Global Ocean Biogeochemistry Hindcast
**Phytoplankton**	mg/m3	0.25 °	Global Ocean Biogeochemistry Hindcast
**Northward surface current**	m/s	0.08 °	Global Ocean Physics Reanalysis
**Eastward surface current**	m/s	0.08 °	Global Ocean Physics Reanalysis
**Sea ice concentration**	fraction of 1	0.08 °	Global Ocean Physics Reanalysis
**Future projections**			
**Variable**	**Units**	**Resolution**	**Source**
**Bathymetry**	m	0.08 °	IPSL Global earth model
**Bottom temperature**	°C	0.08 °	IPSL Global earth model
**Bottom dissolved oxygen**	mmol/m3	0.25 °	IPSL Global earth model
**Phytoplankton**	mg/m3	0.25 °	IPSL Global earth model
**Northward surface current**	m/s	0.08 °	IPSL Global earth model
**Eastward surface current**	m/s	0.08 °	IPSL Global earth model
**Sea ice concentration**	fraction of 1	0.08 °	IPSL Global earth model
