# Supplementary material for: Future trends of marine fish biomass distributions from the North Sea to the Barents Sea
Source: Nat Commun. 2024 Jul 5;15:5637. doi: 10.1038/s41467-024-49911-9 (PMC11224334; doi:10.1038/s41467-024-49911-9)
Supplement: Supplementary file 3 — Description of Additional Supplementary Files [file 41467_2024_49911_MOESM3_ESM.pdf]

Description of Additional Supplementary Files

Supplementary Data 1: Species rate of change between 2010 and 2100 at each shared socio-economic pathway. Species with "NA" in the last three columns correspond to species for which biomass models did not pass the quality threshold.

Supplementary Data 2: Species' probability of occurrences projected at present-day conditions and 2100 under three socioeconomic pathways.

Supplementary Data 3: Species' biomasses projected at present-day conditions and 2100 under three socioeconomic pathways.

Supplementary Data 4: Species' geographic range fragmentation between 2010 and 2100 at each shared socio-economic pathway.

Supplementary Data 5: Species included in the analysis and number of trawls in which each species was recorded from the total of 16,345 trawls. Five-fold diagnostics AUC and r2 were used to filter out species with poor predictive performance, which was detected in 41 species' biomass models (r2 < 0.05, red coloured). Those species were excluded in analyses of biomass-based range shifts. Species are ranked by R2.
