# Supplementary material for: Future trends of marine fish biomass distributions from the North Sea to the Barents Sea
Source: Nat Commun. 2024 Jul 5;15:5637. doi: 10.1038/s41467-024-49911-9 (PMC11224334; doi:10.1038/s41467-024-49911-9)
Supplement: Supplementary file 5 — Supplementary Data 2 [file 41467_2024_49911_MOESM5_ESM.zip › Supplementary Data 2/Supplementary Data 2.pdf]

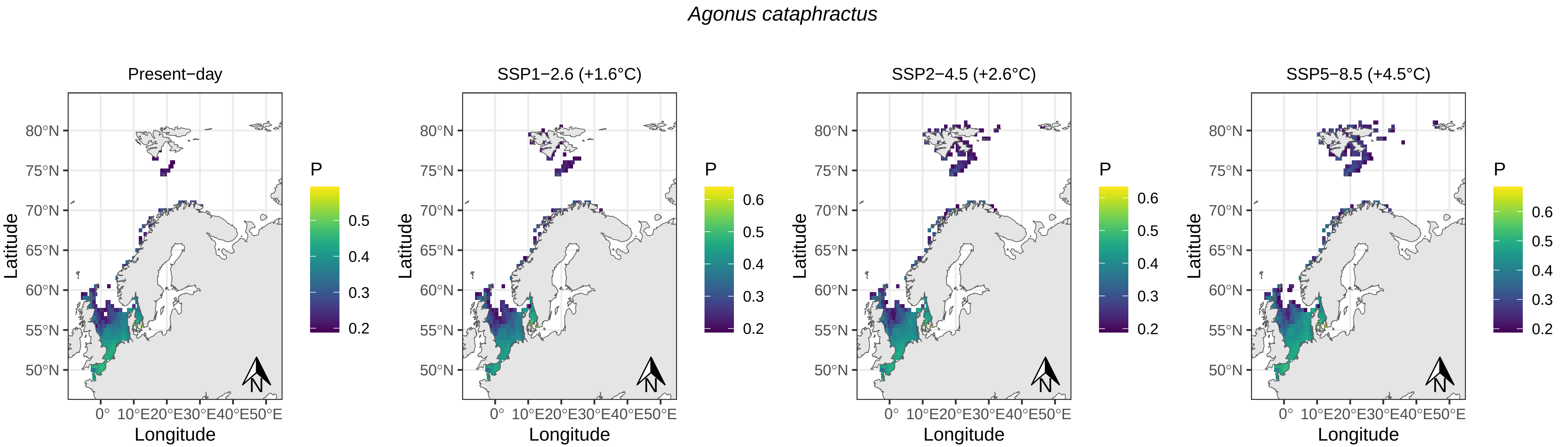

Agonus cataphractus
Present−day
SSP1−2.6 (+1.6°C)
SSP2−4.5 (+2.6°C)
SSP5−8.5 (+4.5°C)
80°N
75°N
70°N
65°N
60°N
55°N
50°N
Latitude
0°
10°E
20°E
30°E
40°E
50°E
Longitude
P
0.2
0.3
0.4
0.5
0.6
N

*Amblyraja hyperborea*

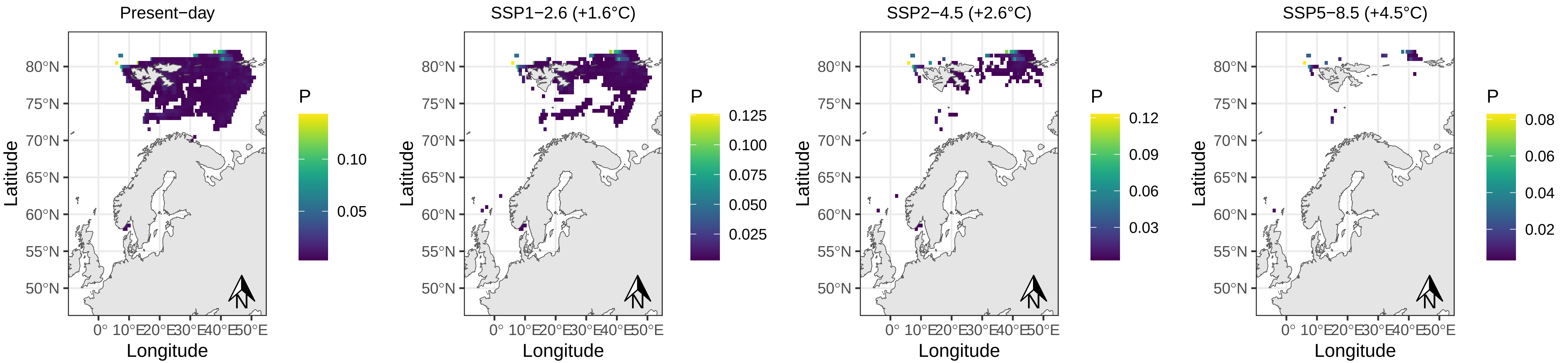

*Amblyraja radiata*

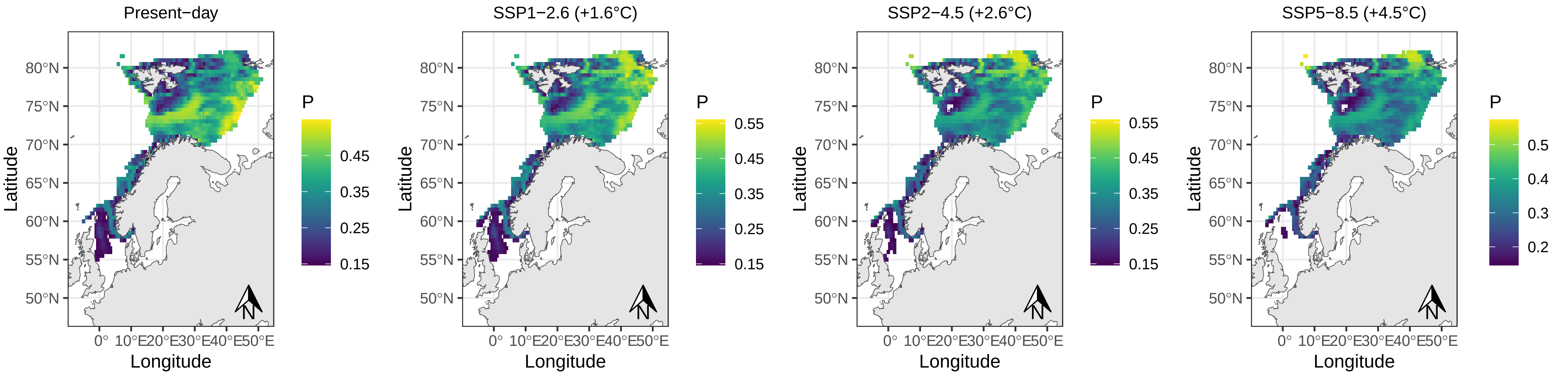

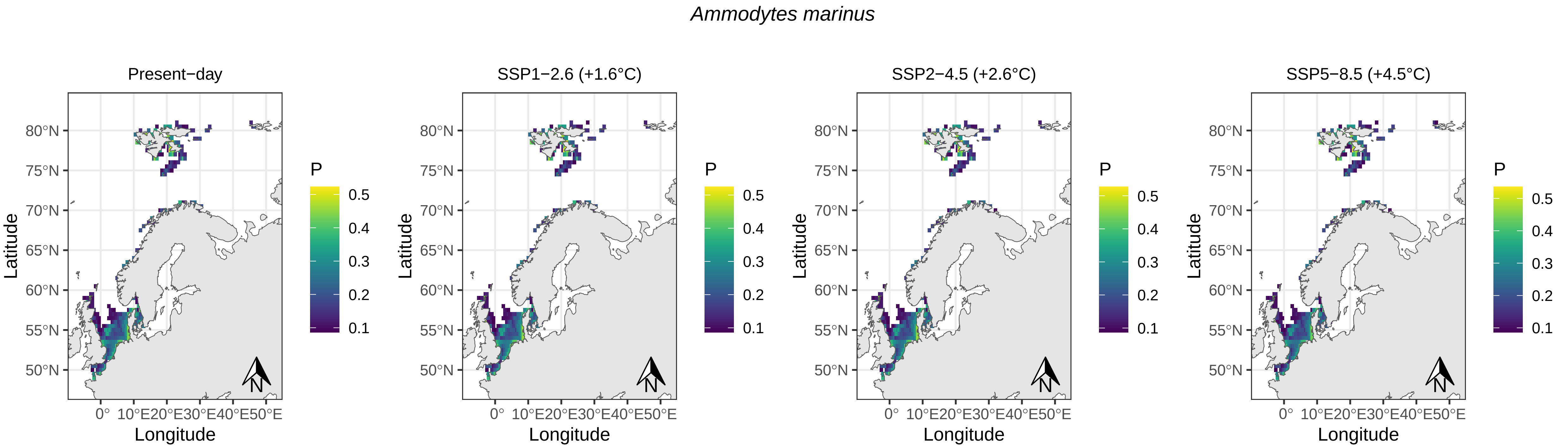

Ammodytes marinus
Present−day
SSP1−2.6 (+1.6°C)
SSP2−4.5 (+2.6°C)
SSP5−8.5 (+4.5°C)
80°N
75°N
70°N
65°N
60°N
55°N
50°N
Latitude
0°
10°E
20°E
30°E
40°E
50°E
Longitude
P
0.5
0.4
0.3
0.2
0.1
N

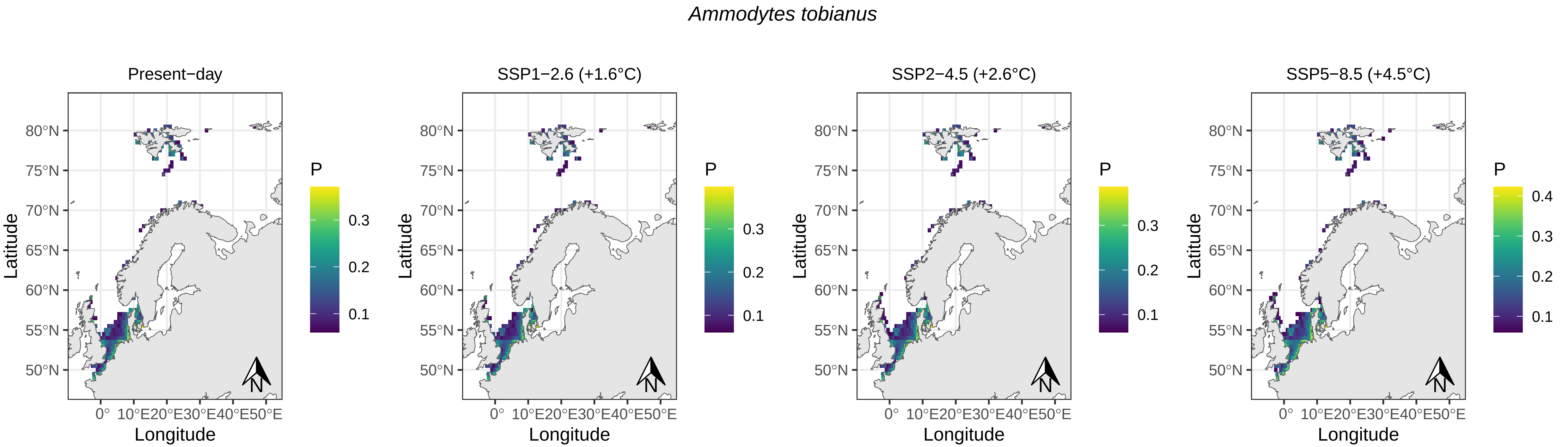

*Ammodytes tobianus*
Present−day
SSP1−2.6 (+1.6°C)
SSP2−4.5 (+2.6°C)
SSP5−8.5 (+4.5°C)
Latitude
Longitude
80°N
75°N
70°N
65°N
60°N
55°N
50°N
0°
10°E20°E30°E40°E50°E
P
0.1
0.2
0.3
0.4
N

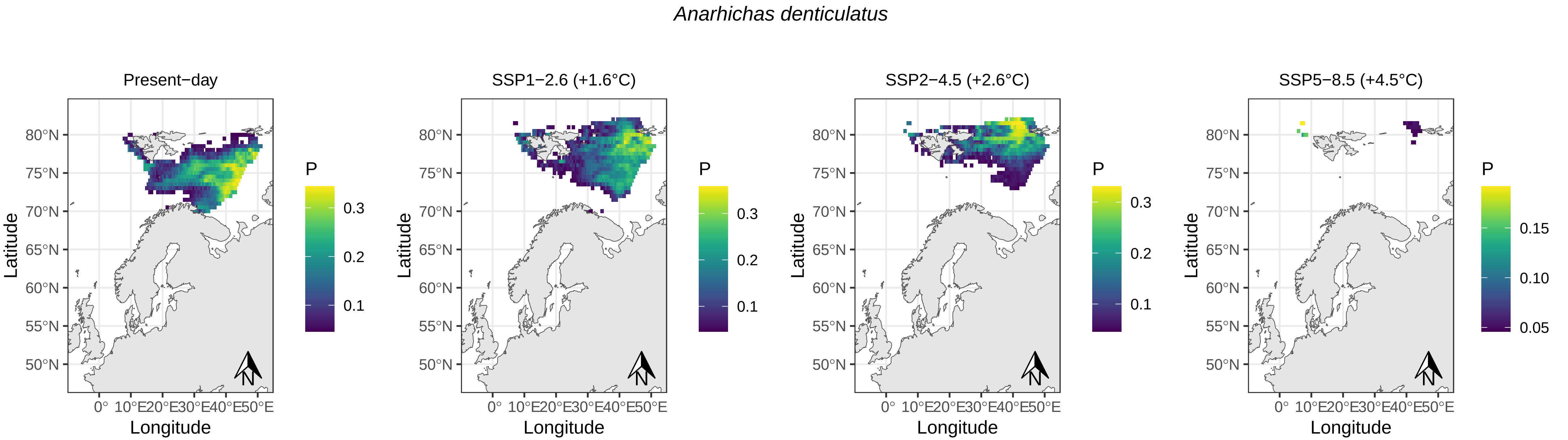
Anarhichas denticulatus
Present−day
SSP1−2.6 (+1.6°C)
SSP2−4.5 (+2.6°C)
SSP5−8.5 (+4.5°C)
Latitude
Longitude
80°N
75°N
70°N
65°N
60°N
55°N
50°N
0°
10°E
20°E
30°E
40°E
50°E
P
0.3
0.2
0.1
0.15
0.10
0.05
N

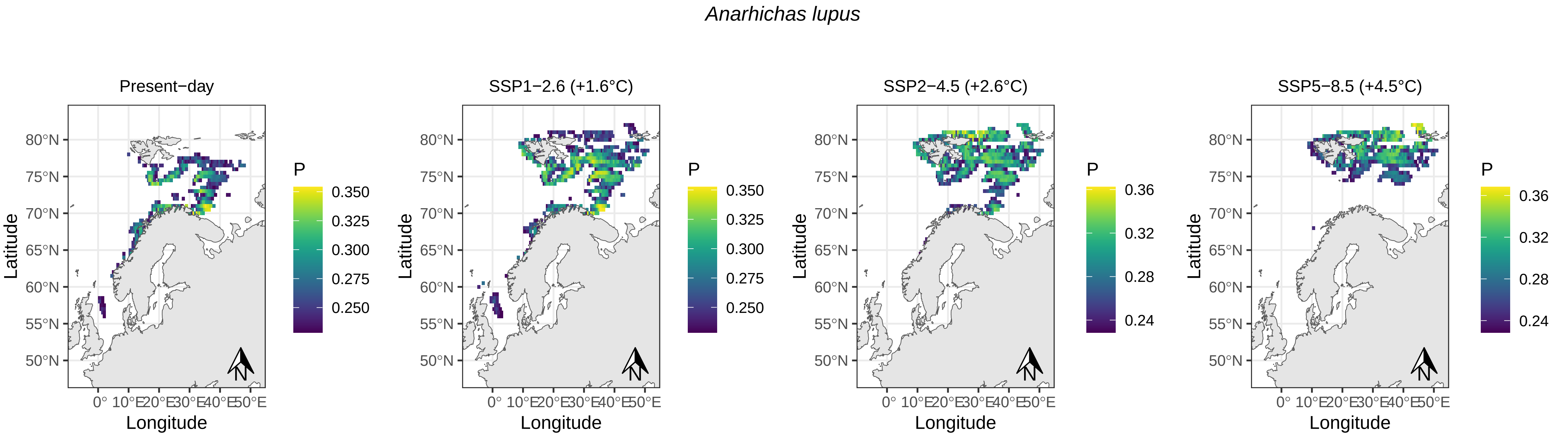
Anarhichas lupus
Present−day
SSP1−2.6 (+1.6°C)
SSP2−4.5 (+2.6°C)
SSP5−8.5 (+4.5°C)
80°N
75°N
70°N
65°N
60°N
55°N
50°N
Latitude
0°
10°E
20°E
30°E
40°E
50°E
Longitude
P
0.350
0.325
0.300
0.275
0.250
0.36
0.32
0.28
0.24
N

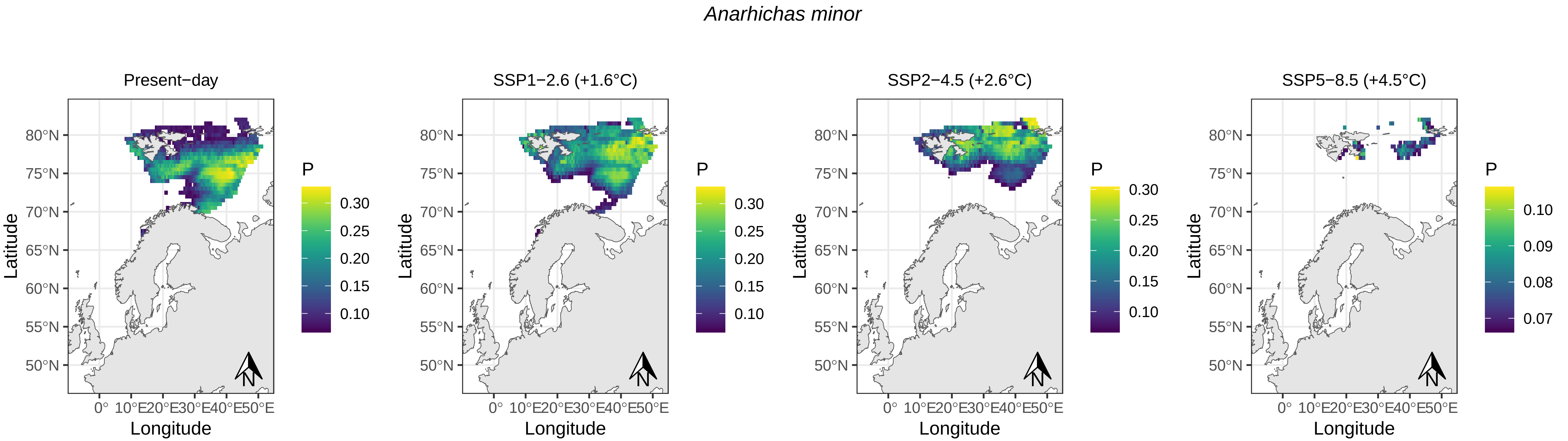
Anarhichas minor
Present−day
SSP1−2.6 (+1.6°C)
SSP2−4.5 (+2.6°C)
SSP5−8.5 (+4.5°C)
80°N
75°N
70°N
65°N
60°N
55°N
50°N
Latitude
0°
10°E
20°E
30°E
40°E
50°E
Longitude
P
0.30
0.25
0.20
0.15
0.10
0.09
0.08
0.07
N

*Anguilla anguilla*

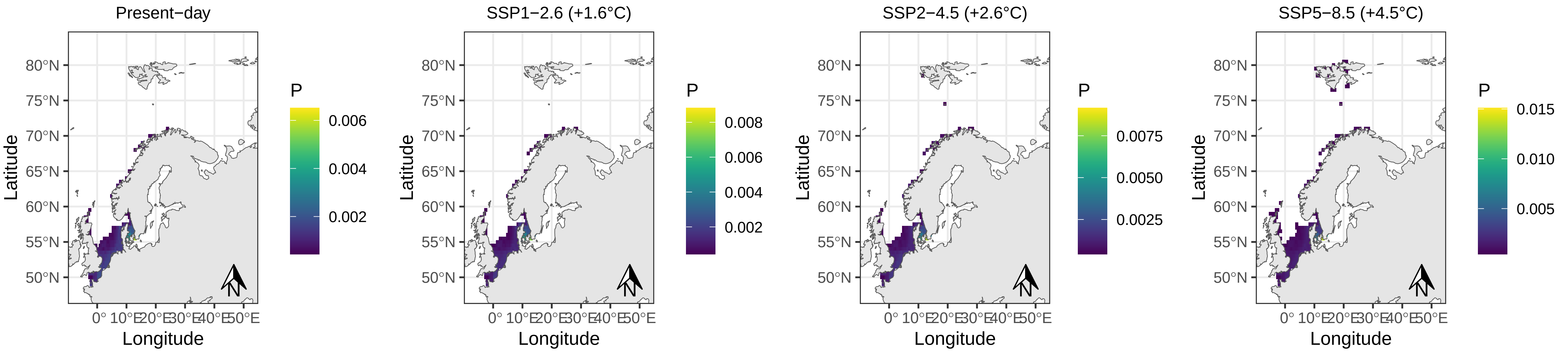

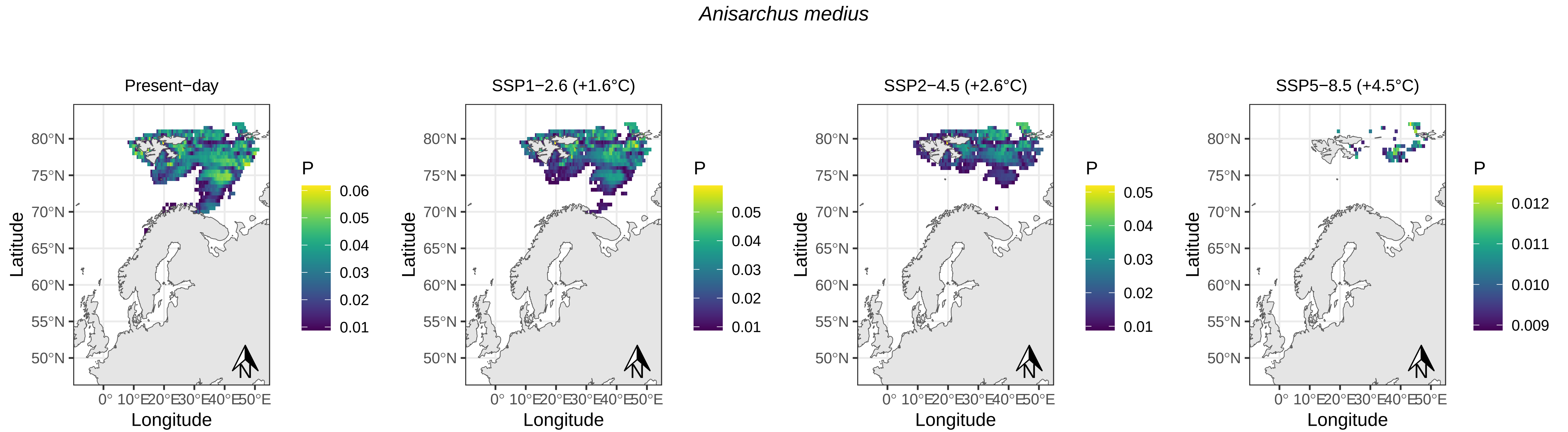

Anisarchus medius
Present−day
SSP1−2.6 (+1.6°C)
SSP2−4.5 (+2.6°C)
SSP5−8.5 (+4.5°C)
Latitude
Longitude
80°N
75°N
70°N
65°N
60°N
55°N
50°N
0°
10°E
20°E
30°E
40°E
50°E
P
0.06
0.05
0.04
0.03
0.02
0.01
0.012
0.011
0.010
0.009
N

# *Aphia minuta*

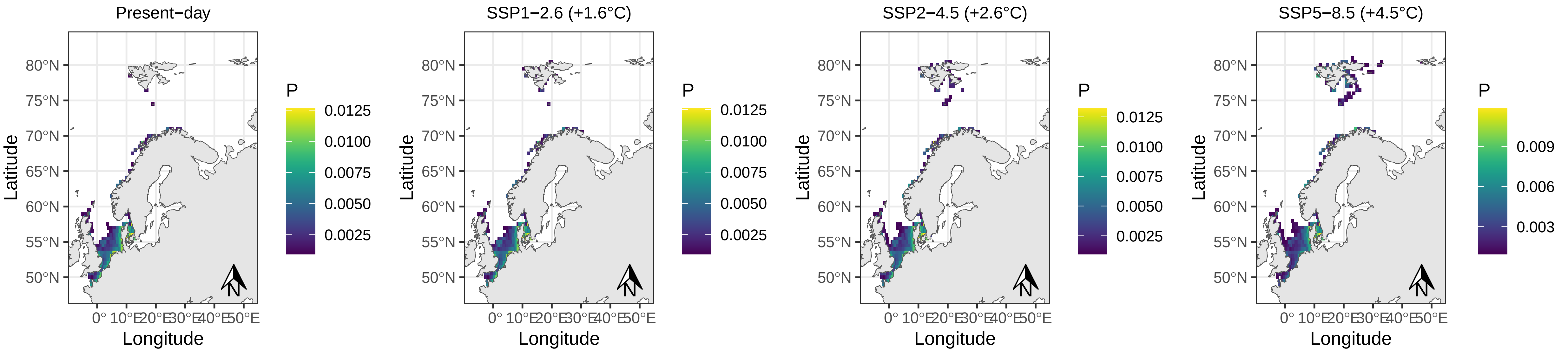

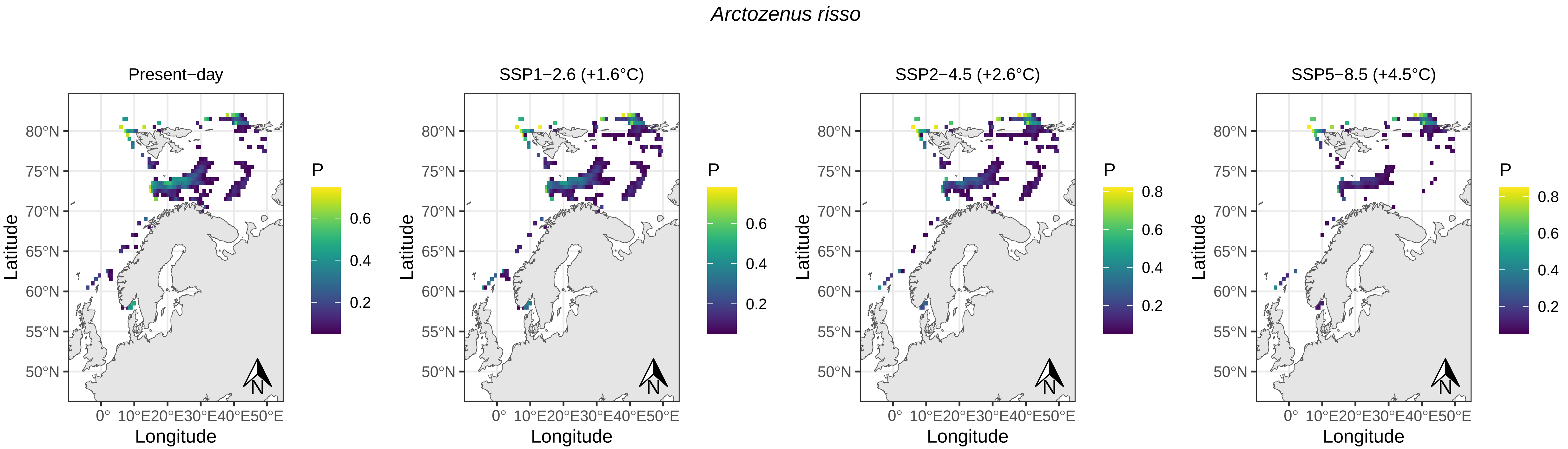

*Arctozenus risso*
Present−day
SSP1−2.6 (+1.6°C)
SSP2−4.5 (+2.6°C)
SSP5−8.5 (+4.5°C)
Latitude
Longitude
80°N
75°N
70°N
65°N
60°N
55°N
50°N
0°
10°E
20°E
30°E
40°E
50°E
P
0.2
0.4
0.6
0.8
N

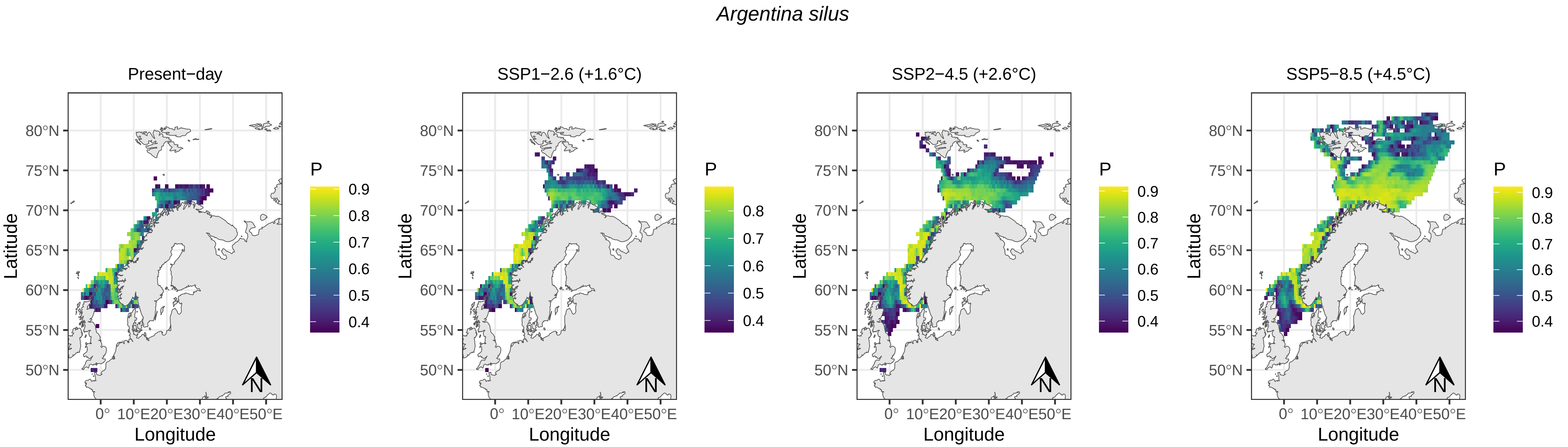

Argentina silus
Present−day
SSP1−2.6 (+1.6°C)
SSP2−4.5 (+2.6°C)
SSP5−8.5 (+4.5°C)
Latitude
Longitude
80°N
75°N
70°N
65°N
60°N
55°N
50°N
0° 10°E20°E30°E40°E50°E
P
0.9
0.8
0.7
0.6
0.5
0.4
N

*Argentina sphyraena*

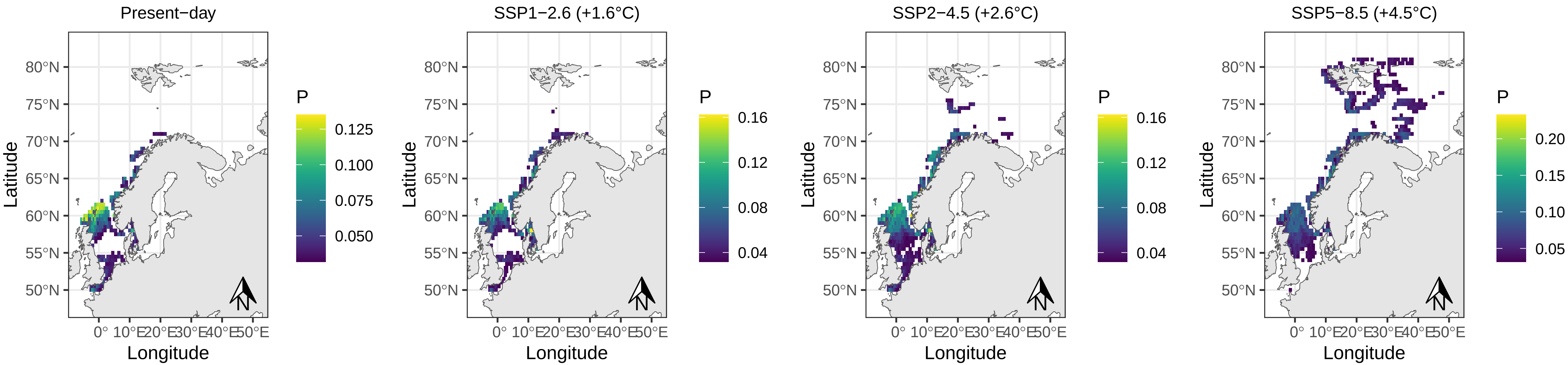

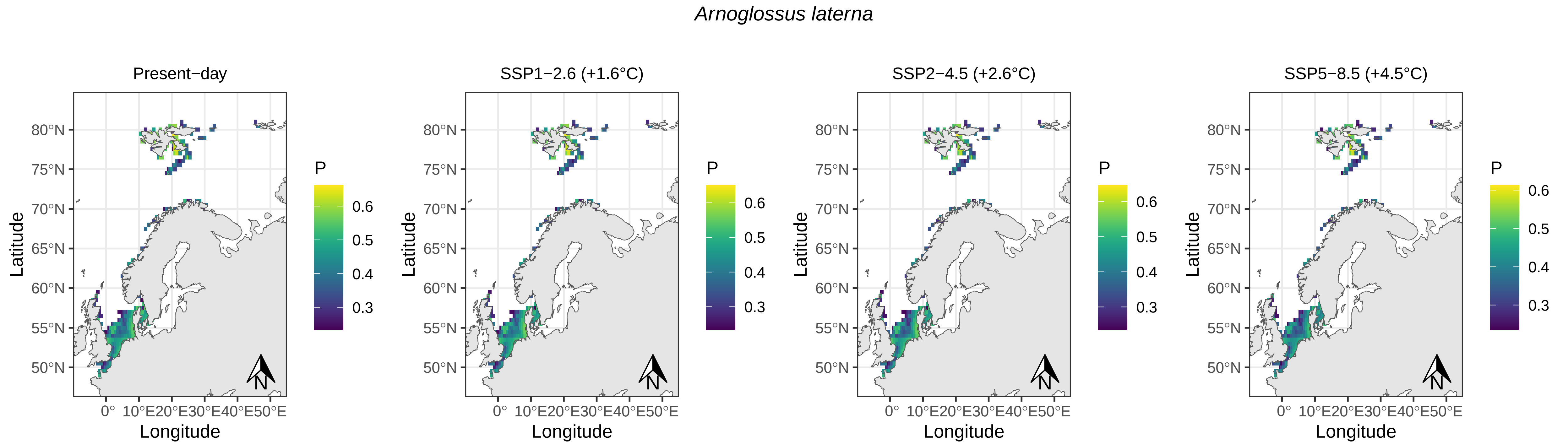
Arnoglossus laterna
Present−day
SSP1−2.6 (+1.6°C)
SSP2−4.5 (+2.6°C)
SSP5−8.5 (+4.5°C)
Latitude
Longitude
80°N
75°N
70°N
65°N
60°N
55°N
50°N
0°
10°E
20°E
30°E
40°E
50°E
P
0.3
0.4
0.5
0.6
N

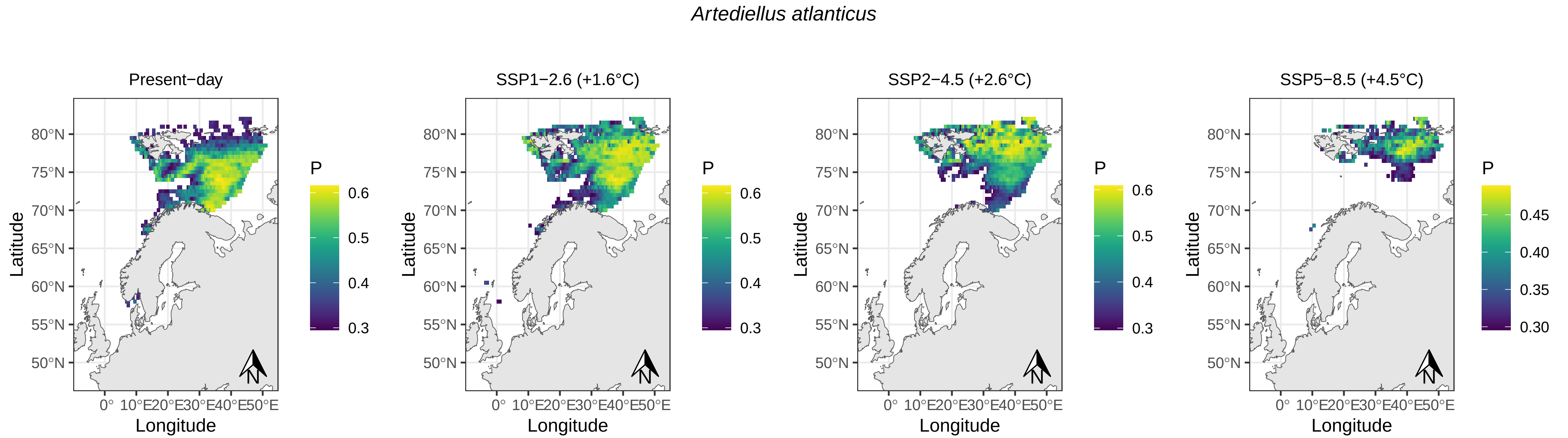

Artediellus atlanticus
Present−day
SSP1−2.6 (+1.6°C)
SSP2−4.5 (+2.6°C)
SSP5−8.5 (+4.5°C)
Latitude
Longitude
80°N
75°N
70°N
65°N
60°N
55°N
50°N
0° 10°E 20°E 30°E 40°E 50°E
P
0.6
0.5
0.4
0.3
0.45
0.40
0.35
0.30
N

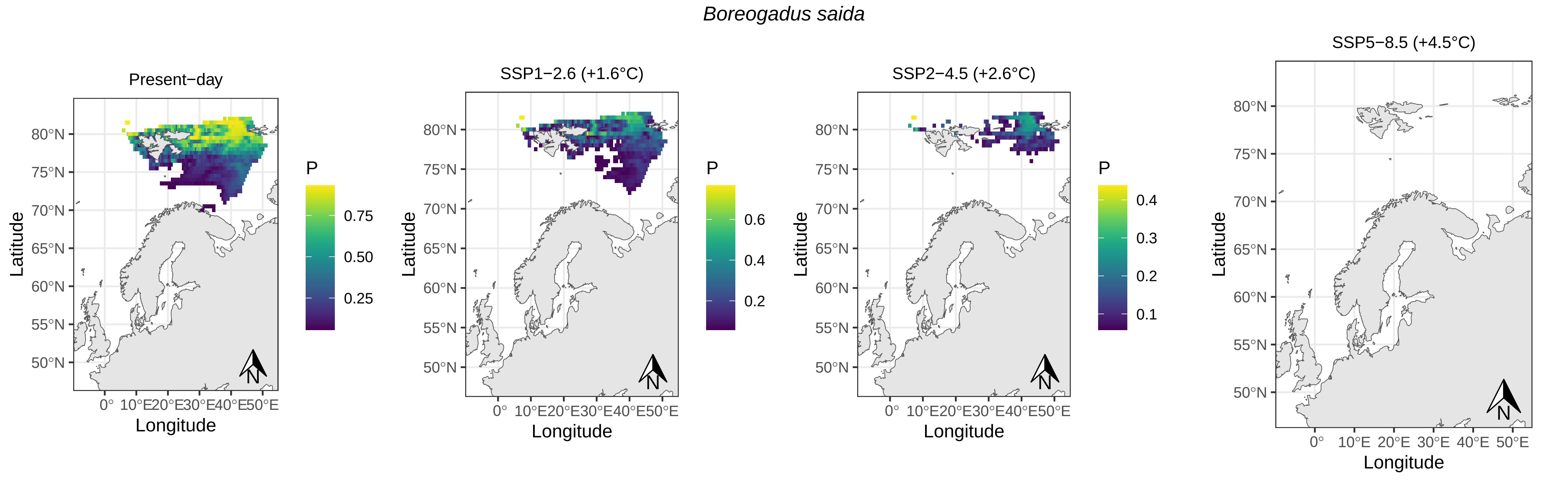

Boreogadus saida
Present−day
SSP1−2.6 (+1.6°C)
SSP2−4.5 (+2.6°C)
SSP5−8.5 (+4.5°C)
Latitude
Longitude
80°N
75°N
70°N
65°N
60°N
55°N
50°N
0°
10°E
20°E
30°E
40°E
50°E
P
0.75
0.50
0.25
0.6
0.4
0.2
0.4
0.3
0.2
0.1
N

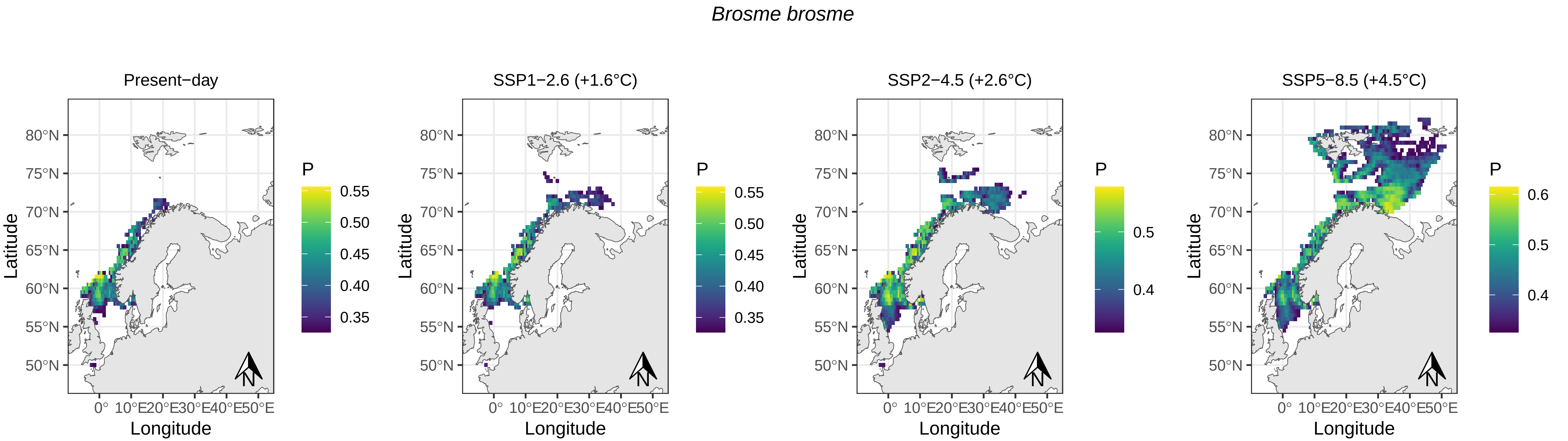
Brosme brosme
Present−day
SSP1−2.6 (+1.6°C)
SSP2−4.5 (+2.6°C)
SSP5−8.5 (+4.5°C)
80°N
75°N
70°N
65°N
60°N
55°N
50°N
Latitude
0° 10°E 20°E 30°E 40°E 50°E
Longitude
N
P
0.55
0.50
0.45
0.40
0.35
0.5
0.4
0.6

*Buglossidium luteum*

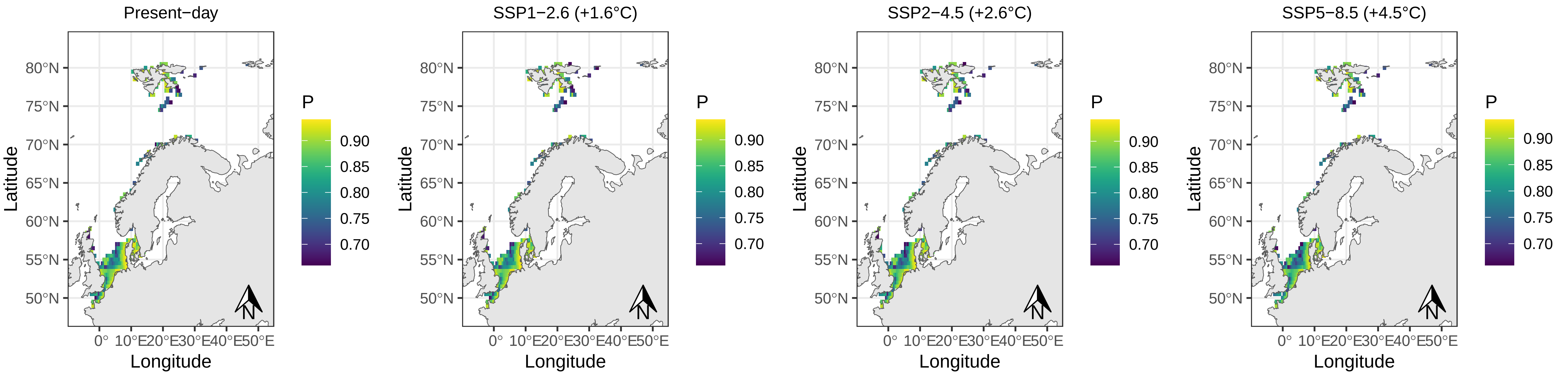

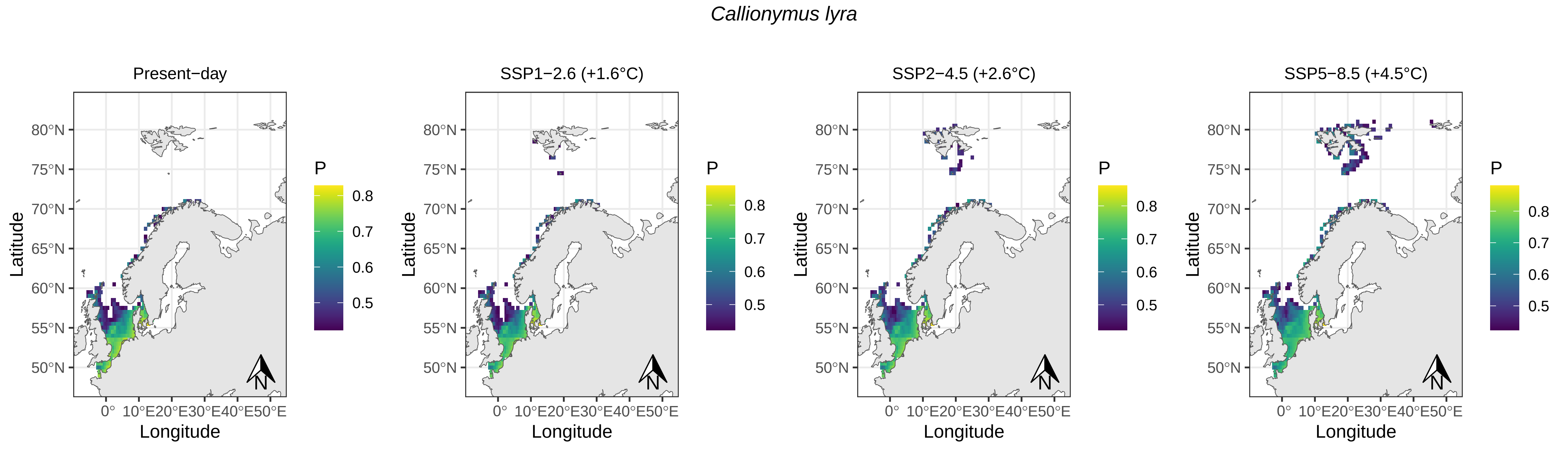
Callionymus lyra
Present−day
SSP1−2.6 (+1.6°C)
SSP2−4.5 (+2.6°C)
SSP5−8.5 (+4.5°C)
Latitude
Longitude
80°N
75°N
70°N
65°N
60°N
55°N
50°N
0°
10°E
20°E
30°E
40°E
50°E
P
0.8
0.7
0.6
0.5
N

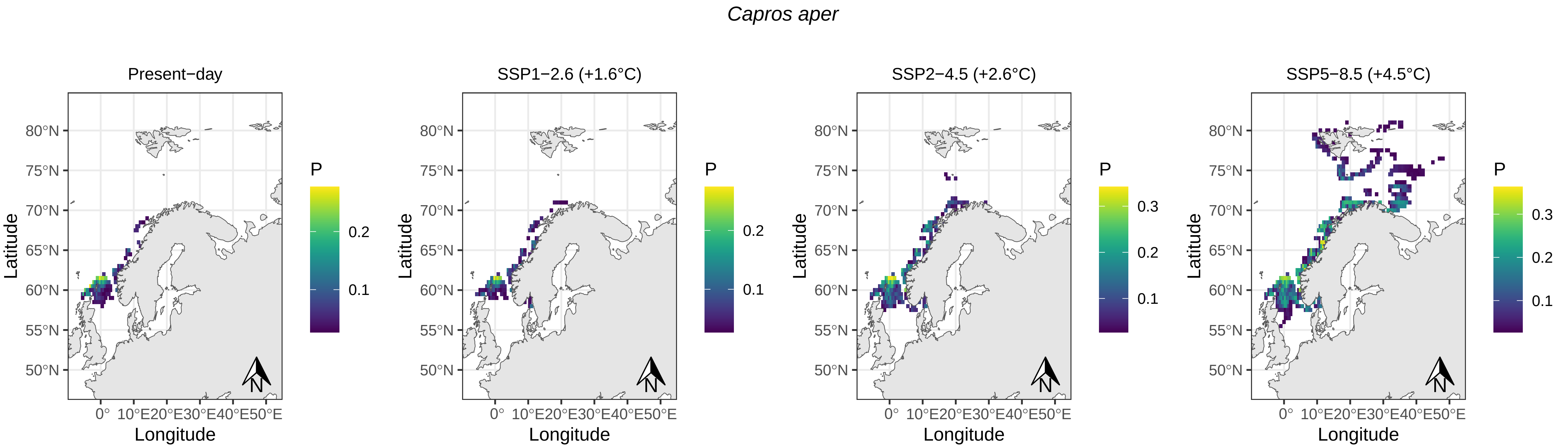

Capros aper
Present−day
SSP1−2.6 (+1.6°C)
SSP2−4.5 (+2.6°C)
SSP5−8.5 (+4.5°C)
80°N
75°N
70°N
65°N
60°N
55°N
50°N
Latitude
0°
10°E
20°E
30°E
40°E
50°E
Longitude
P
0.1
0.2
0.3
N

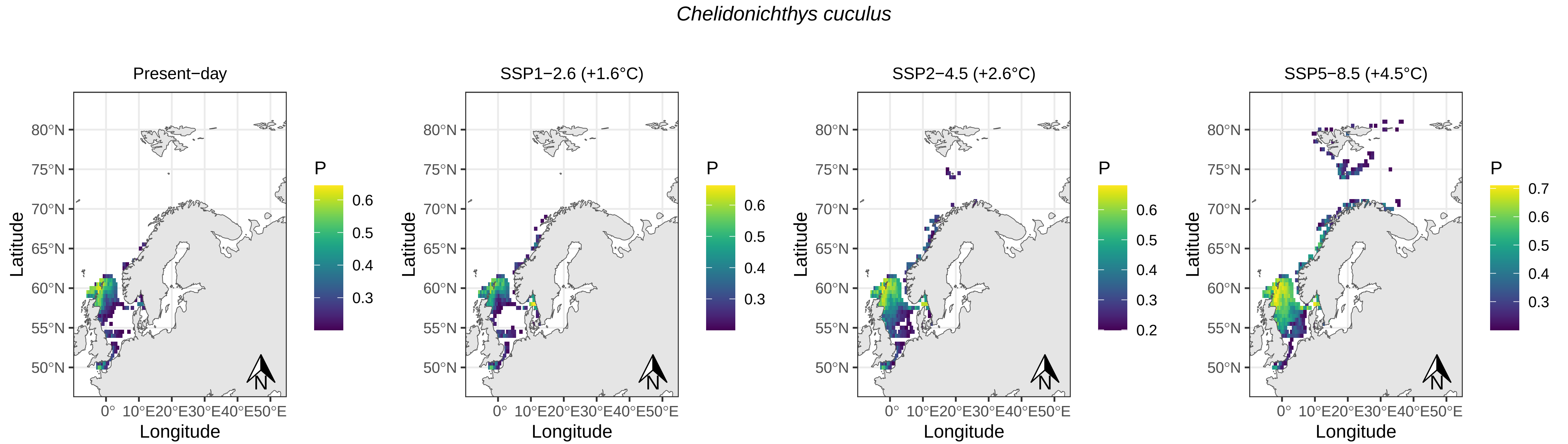
Chelidonichthys cuculus
Present−day
SSP1−2.6 (+1.6°C)
SSP2−4.5 (+2.6°C)
SSP5−8.5 (+4.5°C)
80°N
75°N
70°N
65°N
60°N
55°N
50°N
Latitude
0°
10°E
20°E
30°E
40°E
50°E
Longitude
P
0.2
0.3
0.4
0.5
0.6
0.7
N

*Chelidonichthys lucerna*

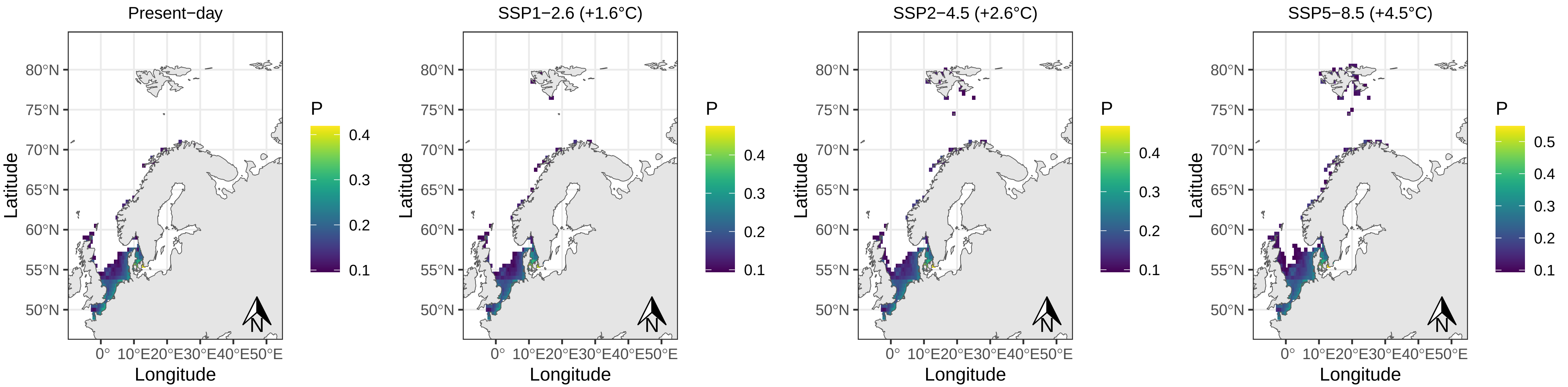

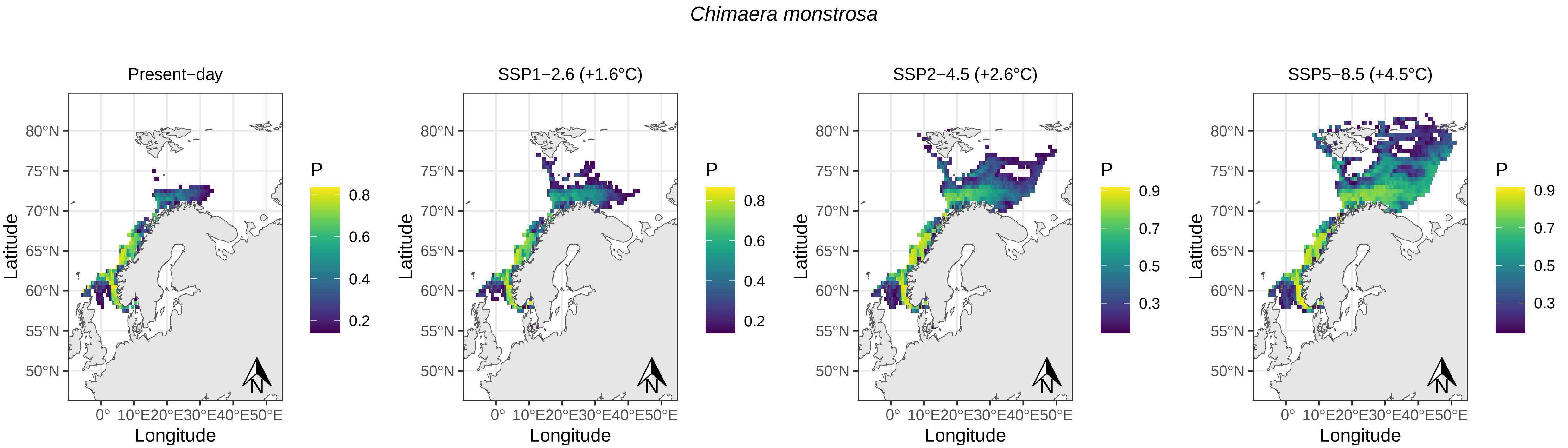
Chimaera monstrosa
Present−day
SSP1−2.6 (+1.6°C)
SSP2−4.5 (+2.6°C)
SSP5−8.5 (+4.5°C)
Latitude
Longitude
80°N
75°N
70°N
65°N
60°N
55°N
50°N
0°
10°E20°E30°E40°E50°E
P
0.8
0.6
0.4
0.2
0.9
0.7
0.5
0.3
N

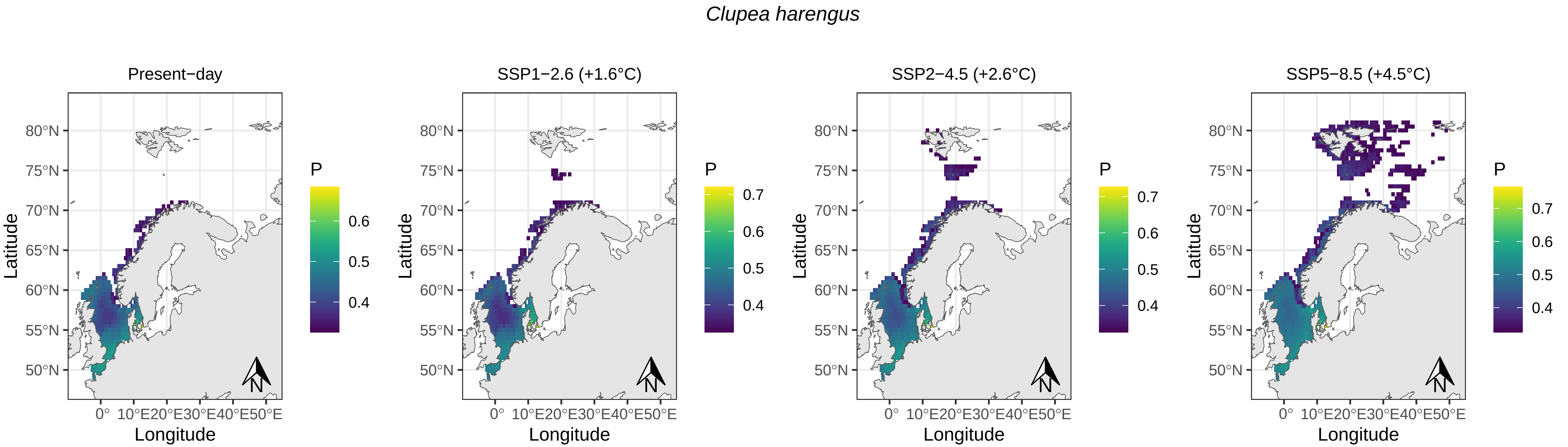

Clupea harengus
Present−day
SSP1−2.6 (+1.6°C)
SSP2−4.5 (+2.6°C)
SSP5−8.5 (+4.5°C)
80°N
75°N
70°N
65°N
60°N
55°N
50°N
Latitude
0°
10°E
20°E
30°E
40°E
50°E
Longitude
P
0.4
0.5
0.6
0.7
N

*Cottunculus microps*

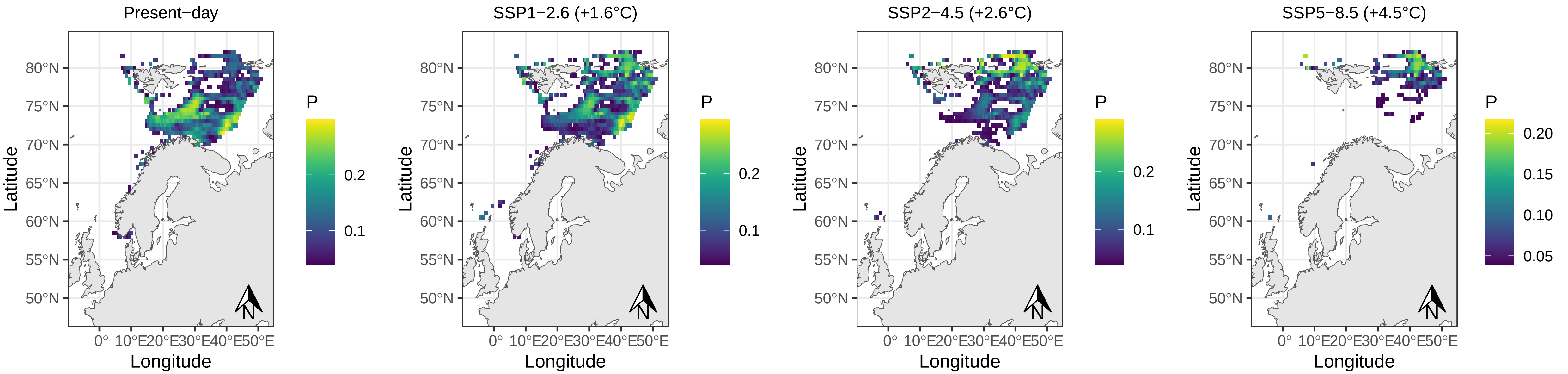

*Crystallogobius linearis*

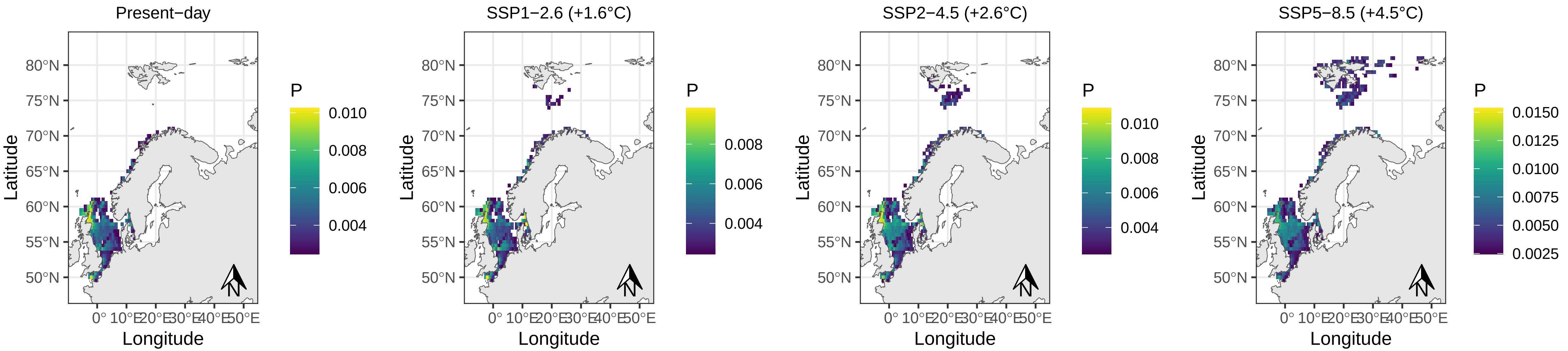

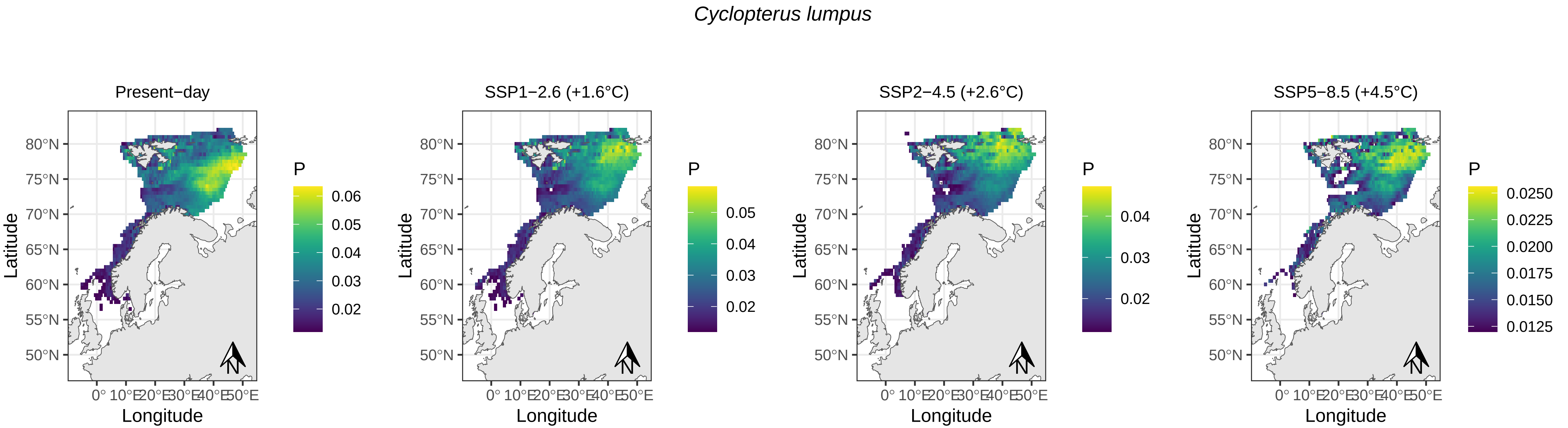
Cyclopterus lumpus
Present−day
SSP1−2.6 (+1.6°C)
SSP2−4.5 (+2.6°C)
SSP5−8.5 (+4.5°C)
Latitude
Longitude
80°N
75°N
70°N
65°N
60°N
55°N
50°N
0°
10°E
20°E
30°E
40°E
50°E
P
0.06
0.05
0.04
0.03
0.02
0.0250
0.0225
0.0200
0.0175
0.0150
0.0125
N

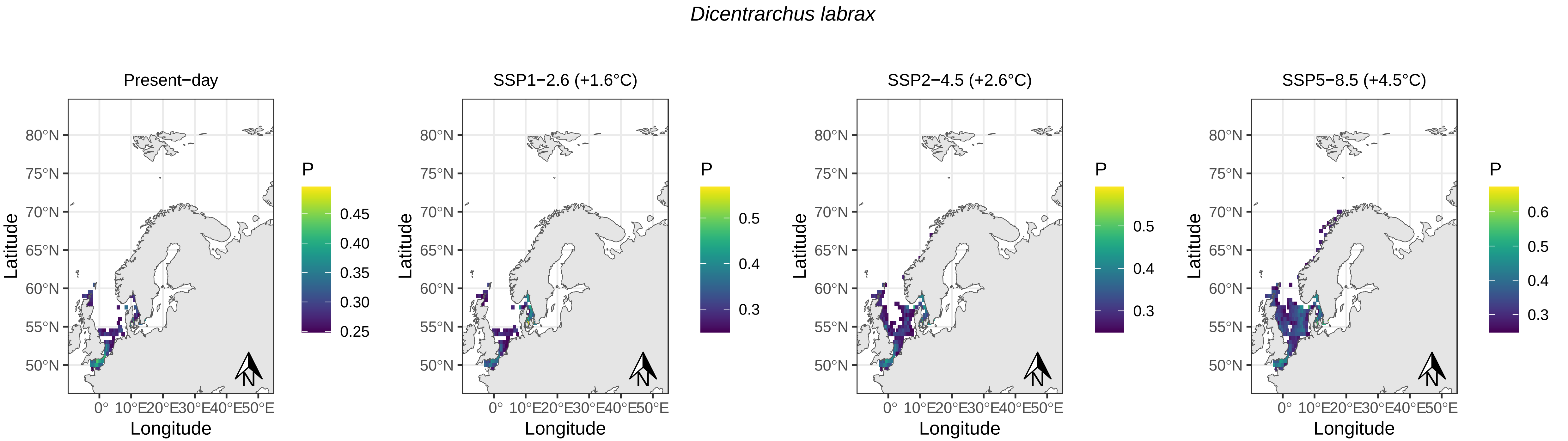
Dicentrarchus labrax
Present−day
SSP1−2.6 (+1.6°C)
SSP2−4.5 (+2.6°C)
SSP5−8.5 (+4.5°C)
Latitude
Longitude
80°N
75°N
70°N
65°N
60°N
55°N
50°N
0° 10°E 20°E 30°E 40°E 50°E
P
0.45
0.40
0.35
0.30
0.25
0.5
0.4
0.3
0.6
N

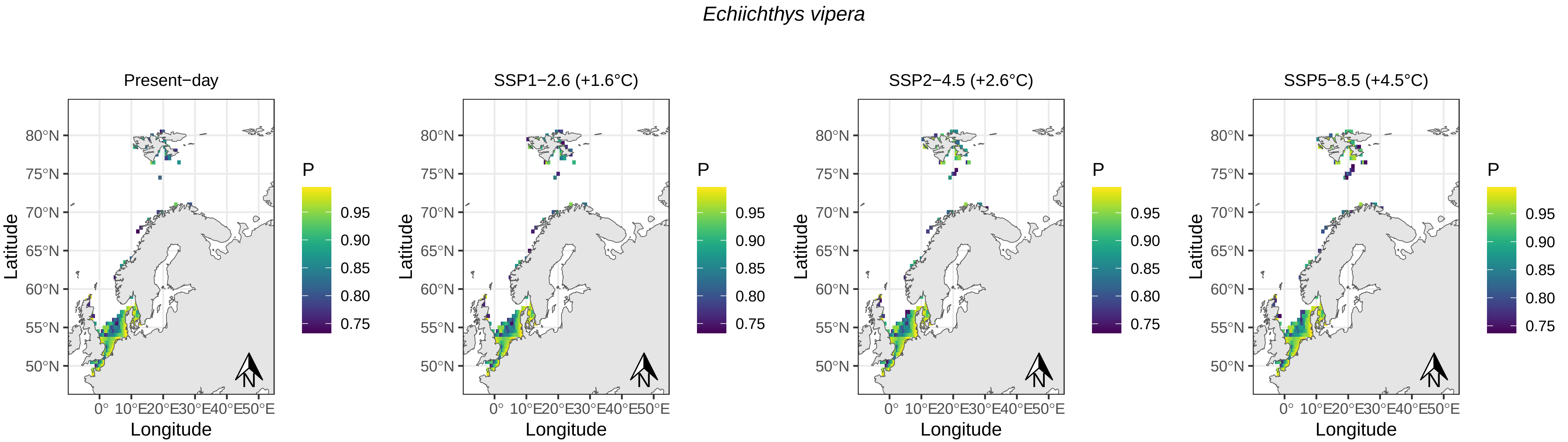

Echiichthys vipera
Present−day
SSP1−2.6 (+1.6°C)
SSP2−4.5 (+2.6°C)
SSP5−8.5 (+4.5°C)
Latitude
Longitude
80°N
75°N
70°N
65°N
60°N
55°N
50°N
0°
10°E
20°E
30°E
40°E
50°E
P
0.95
0.90
0.85
0.80
0.75
N

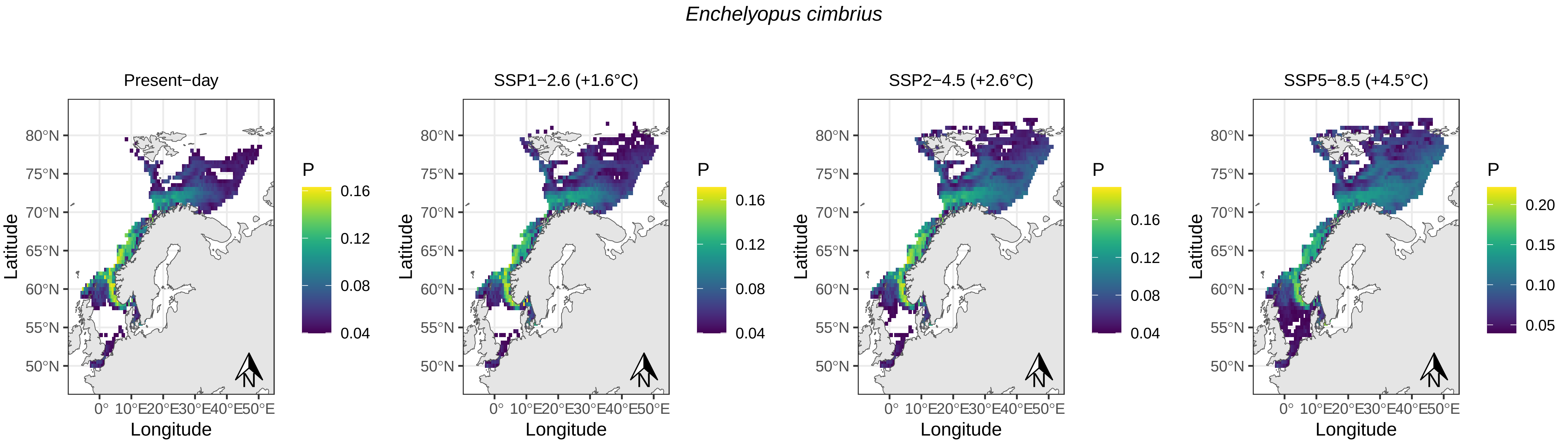

Enchelyopus cimbrius
Present−day
SSP1−2.6 (+1.6°C)
SSP2−4.5 (+2.6°C)
SSP5−8.5 (+4.5°C)
80°N
75°N
70°N
65°N
60°N
55°N
50°N
Latitude
0°
10°E
20°E
30°E
40°E
50°E
Longitude
P
0.16
0.12
0.08
0.04
0.20
0.15
0.10
0.05
N

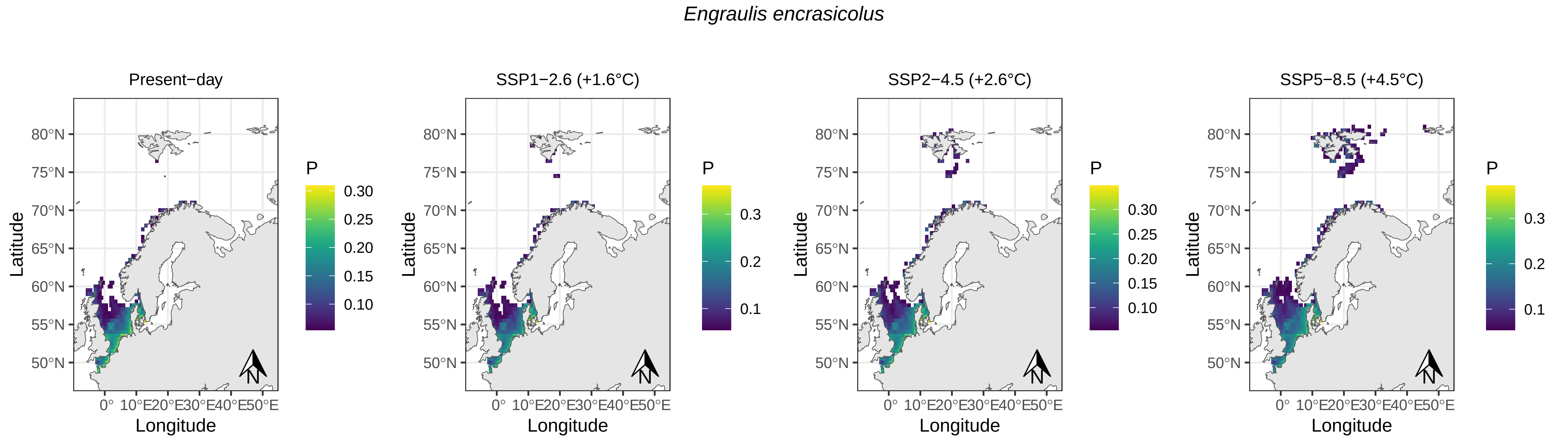
Engraulis encrasicolus
Present−day
SSP1−2.6 (+1.6°C)
SSP2−4.5 (+2.6°C)
SSP5−8.5 (+4.5°C)
Latitude
Longitude
80°N
75°N
70°N
65°N
60°N
55°N
50°N
0° 10°E20°E30°E40°E50°E
P
0.30
0.25
0.20
0.15
0.10
0.3
0.2
0.1
N

*Entelurus aequoreus*

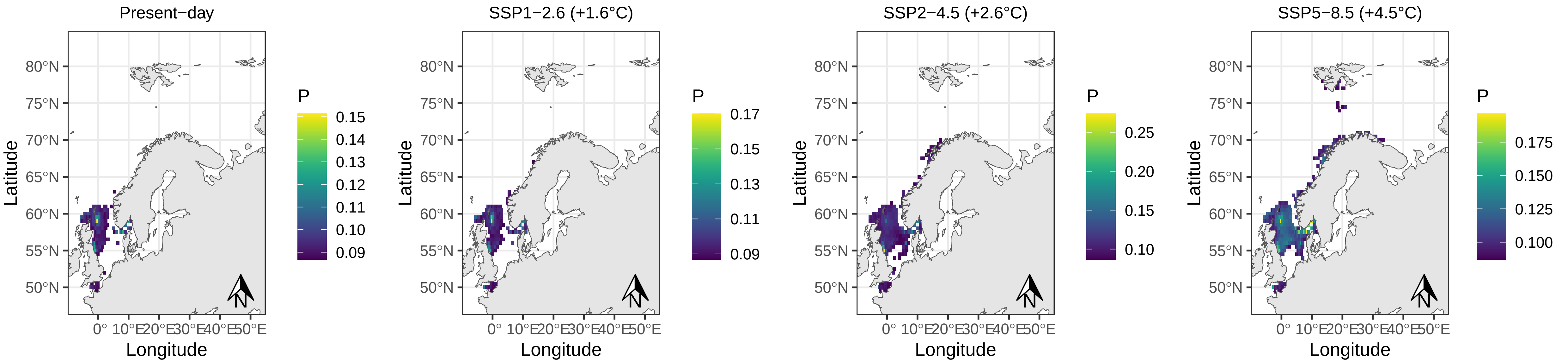

*Etmopterus spinax*

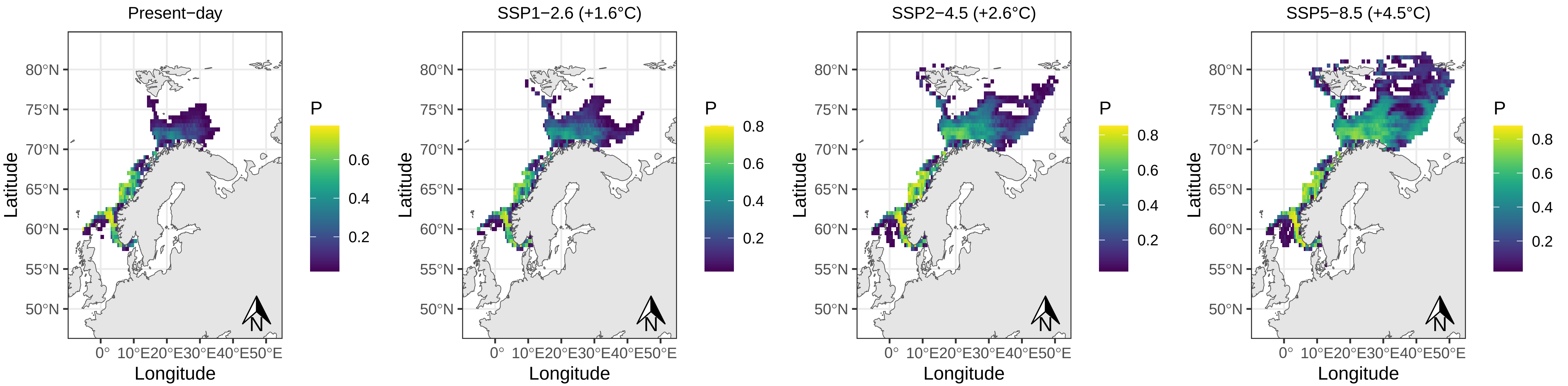

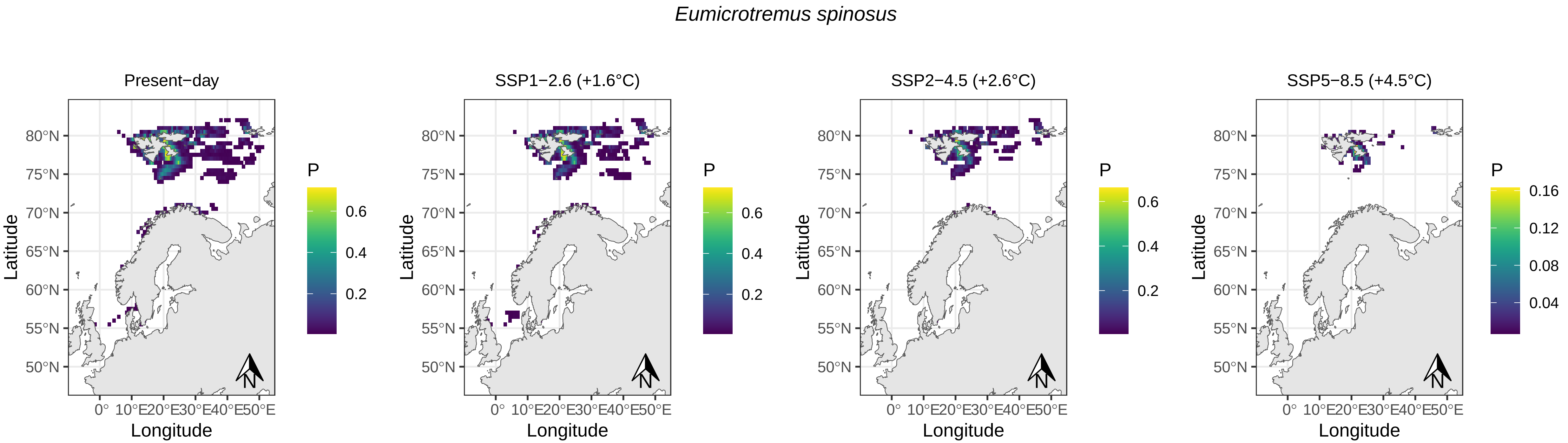

Eumicrotremus spinosus
Present−day
SSP1−2.6 (+1.6°C)
SSP2−4.5 (+2.6°C)
SSP5−8.5 (+4.5°C)
Latitude
Longitude
80°N
75°N
70°N
65°N
60°N
55°N
50°N
0°
10°E
20°E
30°E
40°E
50°E
P
0.2
0.4
0.6
0.04
0.08
0.12
0.16
N

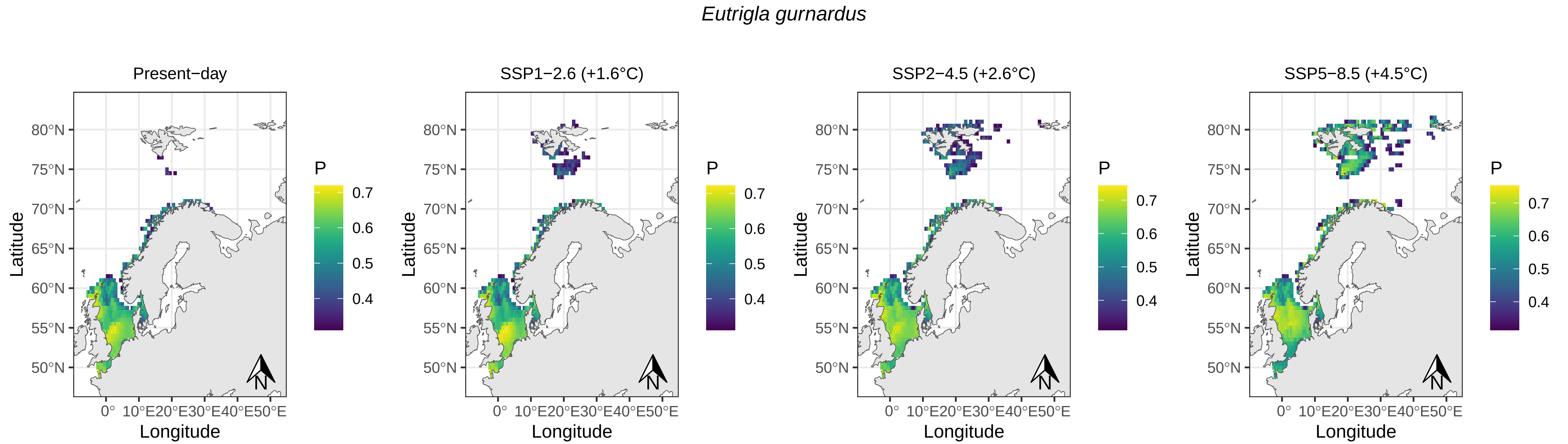

Eutrigla gurnardus
Present−day
SSP1−2.6 (+1.6°C)
SSP2−4.5 (+2.6°C)
SSP5−8.5 (+4.5°C)
Latitude
Longitude
80°N
75°N
70°N
65°N
60°N
55°N
50°N
0°
10°E
20°E
30°E
40°E
50°E
P
0.7
0.6
0.5
0.4
N

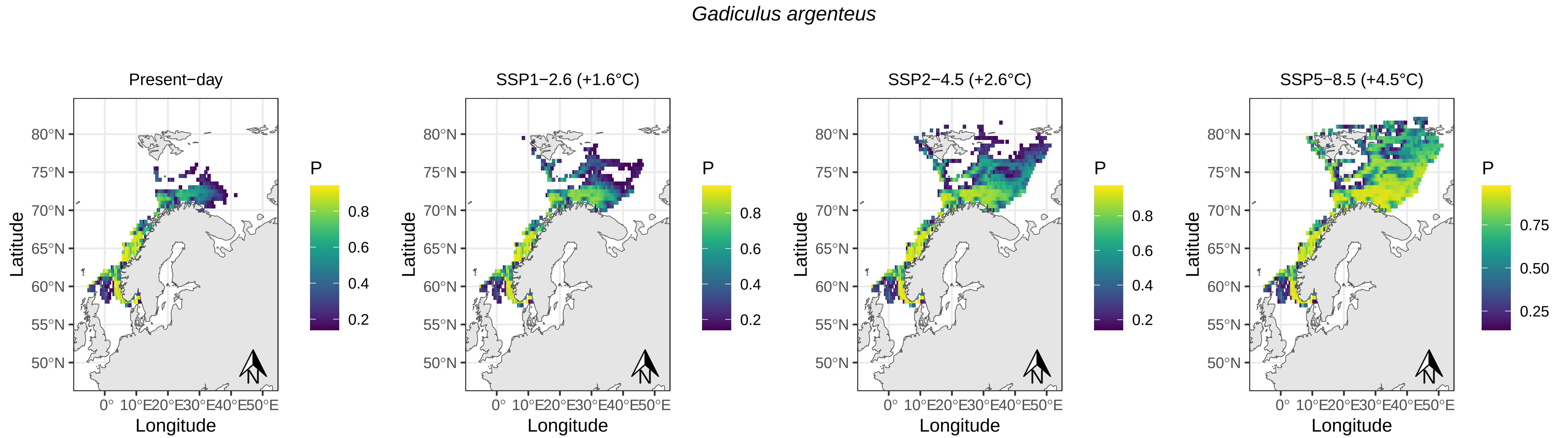

Gadiculus argenteus
Present−day
SSP1−2.6 (+1.6°C)
SSP2−4.5 (+2.6°C)
SSP5−8.5 (+4.5°C)
Latitude
Longitude
80°N
75°N
70°N
65°N
60°N
55°N
50°N
0°
10°E
20°E
30°E
40°E
50°E
P
0.8
0.6
0.4
0.2
0.75
0.50
0.25
N

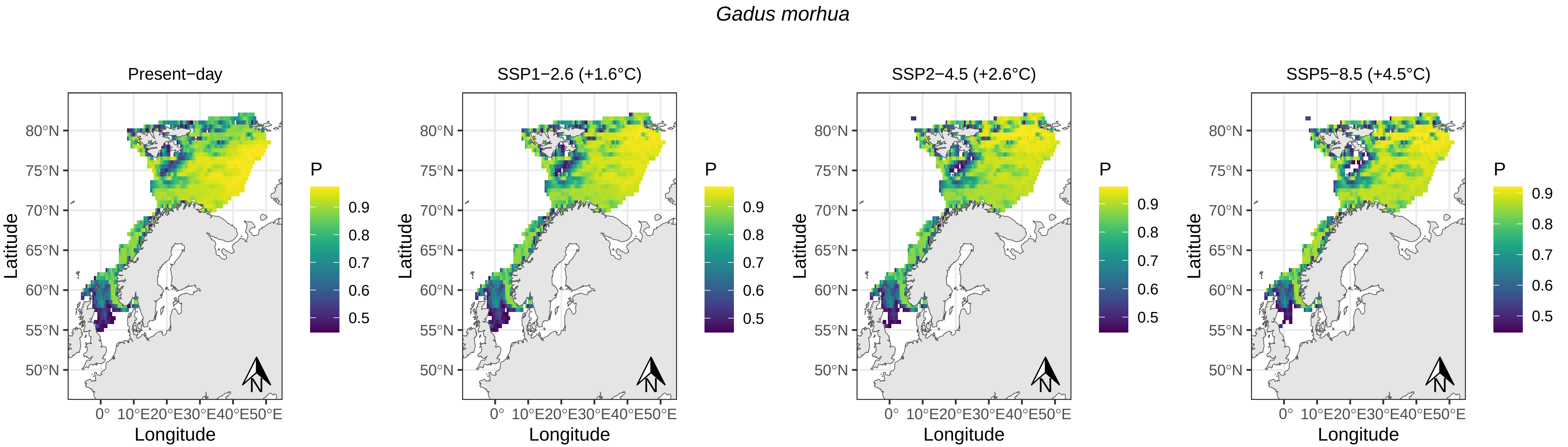
Gadus morhua
Present−day
SSP1−2.6 (+1.6°C)
SSP2−4.5 (+2.6°C)
SSP5−8.5 (+4.5°C)
Latitude
Longitude
80°N
75°N
70°N
65°N
60°N
55°N
50°N
0°
10°E
20°E
30°E
40°E
50°E
P
0.9
0.8
0.7
0.6
0.5
N

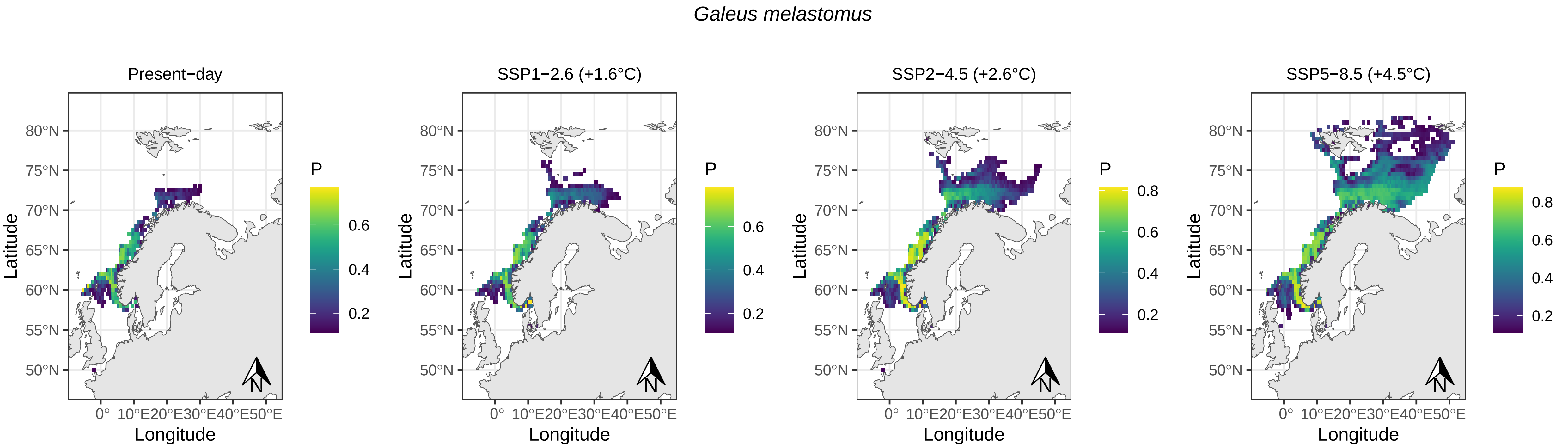

Galeus melastomus
Present−day
SSP1−2.6 (+1.6°C)
SSP2−4.5 (+2.6°C)
SSP5−8.5 (+4.5°C)
80°N
75°N
70°N
65°N
60°N
55°N
50°N
Latitude
0°
10°E
20°E
30°E
40°E
50°E
Longitude
N
P
0.2
0.4
0.6
0.8

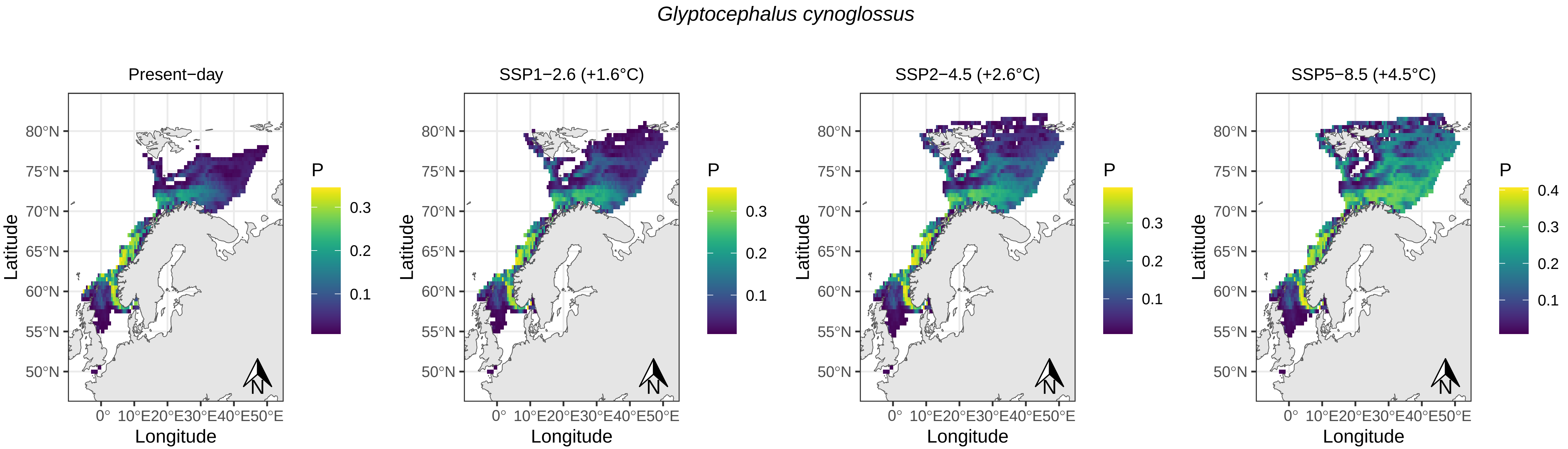
Glyptocephalus cynoglossus
Present−day
SSP1−2.6 (+1.6°C)
SSP2−4.5 (+2.6°C)
SSP5−8.5 (+4.5°C)
Latitude
80°N
75°N
70°N
65°N
60°N
55°N
50°N
Longitude
0° 10°E 20°E 30°E 40°E 50°E
P
0.1
0.2
0.3
0.4
N

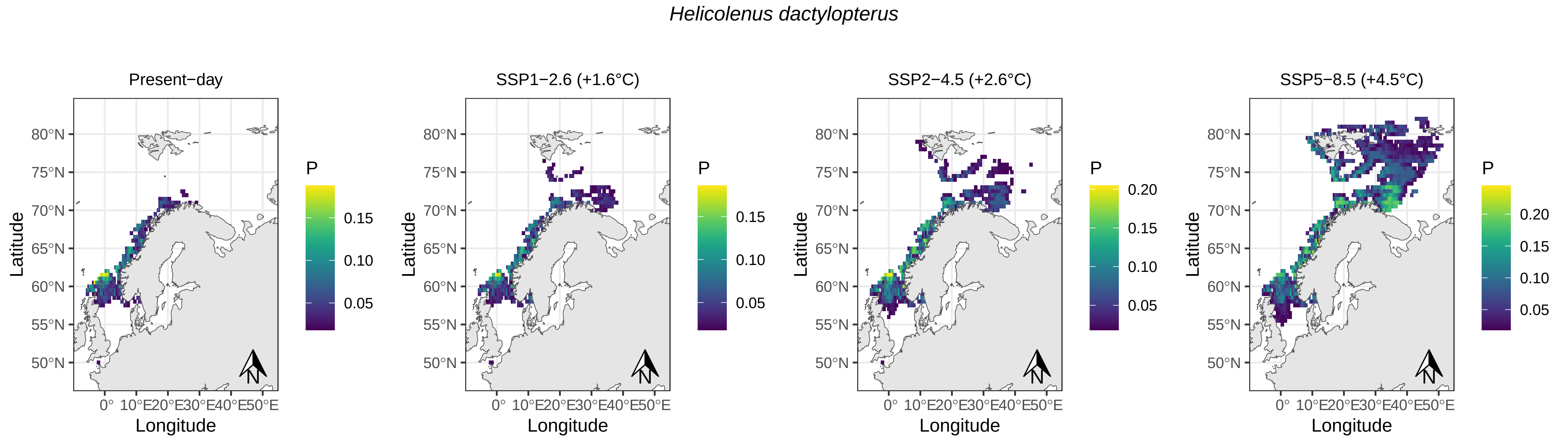

Helicolenus dactylopterus
Present−day
SSP1−2.6 (+1.6°C)
SSP2−4.5 (+2.6°C)
SSP5−8.5 (+4.5°C)
Latitude
Longitude
80°N
75°N
70°N
65°N
60°N
55°N
50°N
0°
10°E
20°E
30°E
40°E
50°E
P
0.05
0.10
0.15
0.20
N

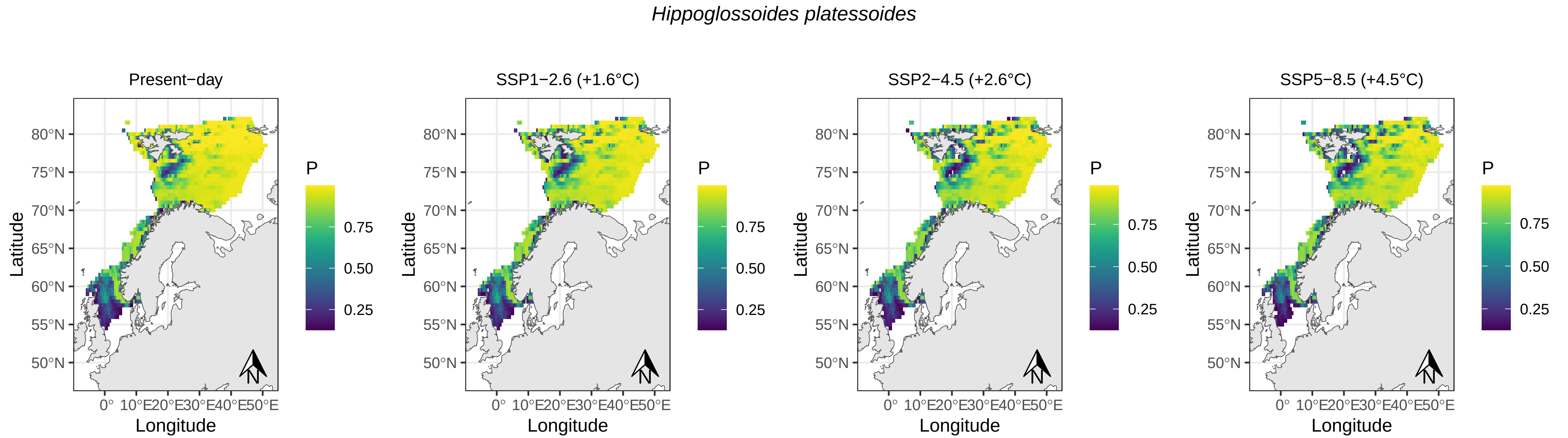

Hippoglossoides platessoides
Present−day
SSP1−2.6 (+1.6°C)
SSP2−4.5 (+2.6°C)
SSP5−8.5 (+4.5°C)
80°N
75°N
70°N
65°N
60°N
55°N
50°N
Latitude
0°
10°E
20°E
30°E
40°E
50°E
Longitude
P
0.75
0.50
0.25
N

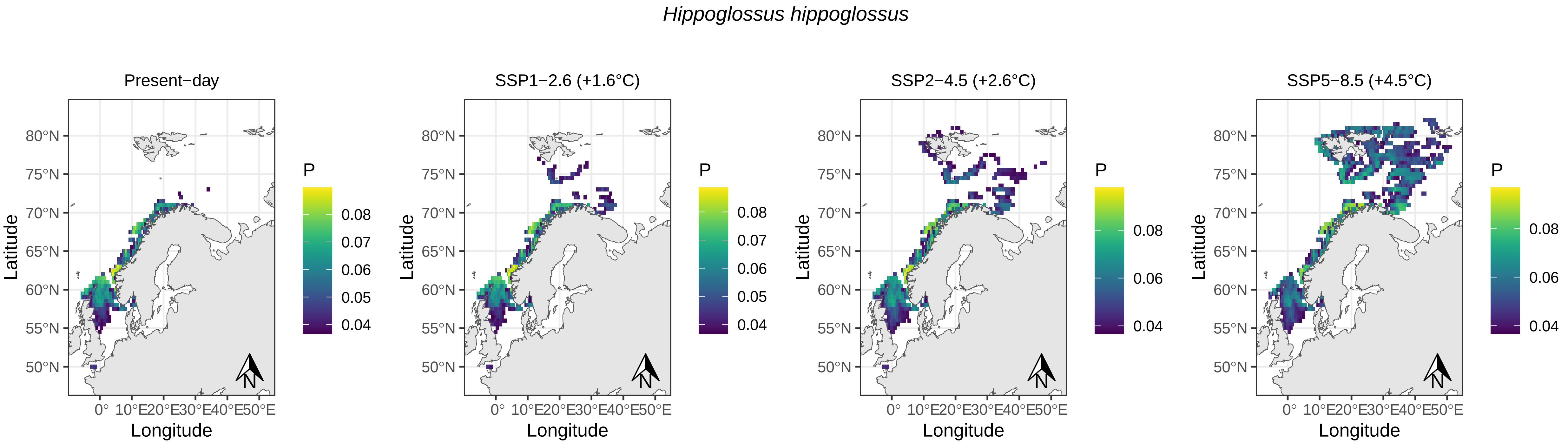

Hippoglossus hippoglossus
Present−day
SSP1−2.6 (+1.6°C)
SSP2−4.5 (+2.6°C)
SSP5−8.5 (+4.5°C)
Latitude
Longitude
80°N
75°N
70°N
65°N
60°N
55°N
50°N
0°
10°E
20°E
30°E
40°E
50°E
P
0.08
0.07
0.06
0.05
0.04
N

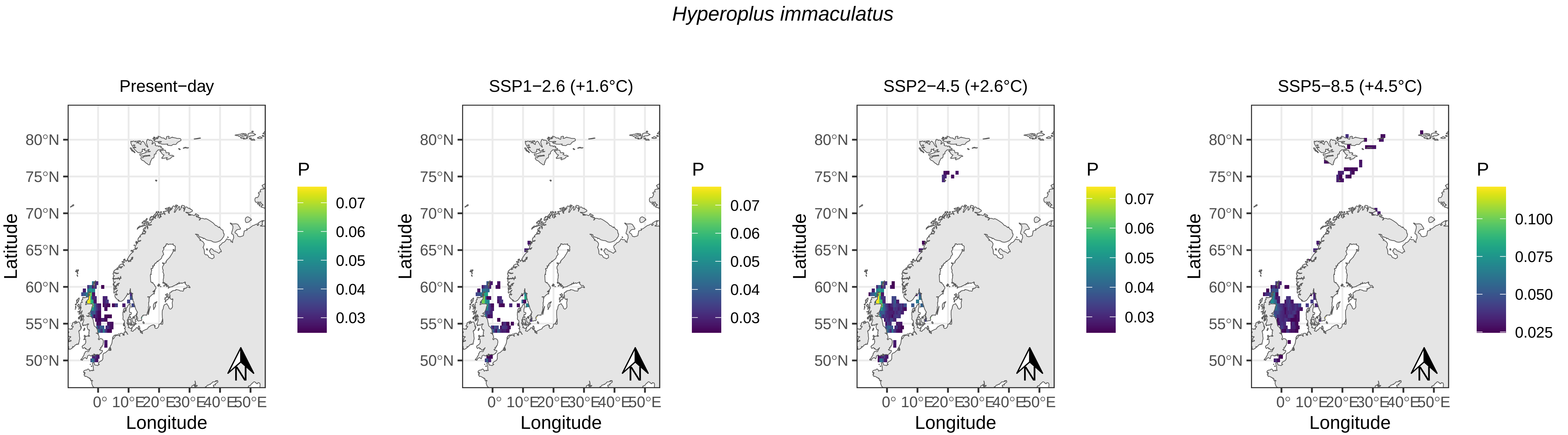
Hyperoplus immaculatus
Present−day
SSP1−2.6 (+1.6°C)
SSP2−4.5 (+2.6°C)
SSP5−8.5 (+4.5°C)
Latitude
Longitude
80°N
75°N
70°N
65°N
60°N
55°N
50°N
0°
10°E
20°E
30°E
40°E
50°E
P
0.07
0.06
0.05
0.04
0.03
0.100
0.075
0.050
0.025
N

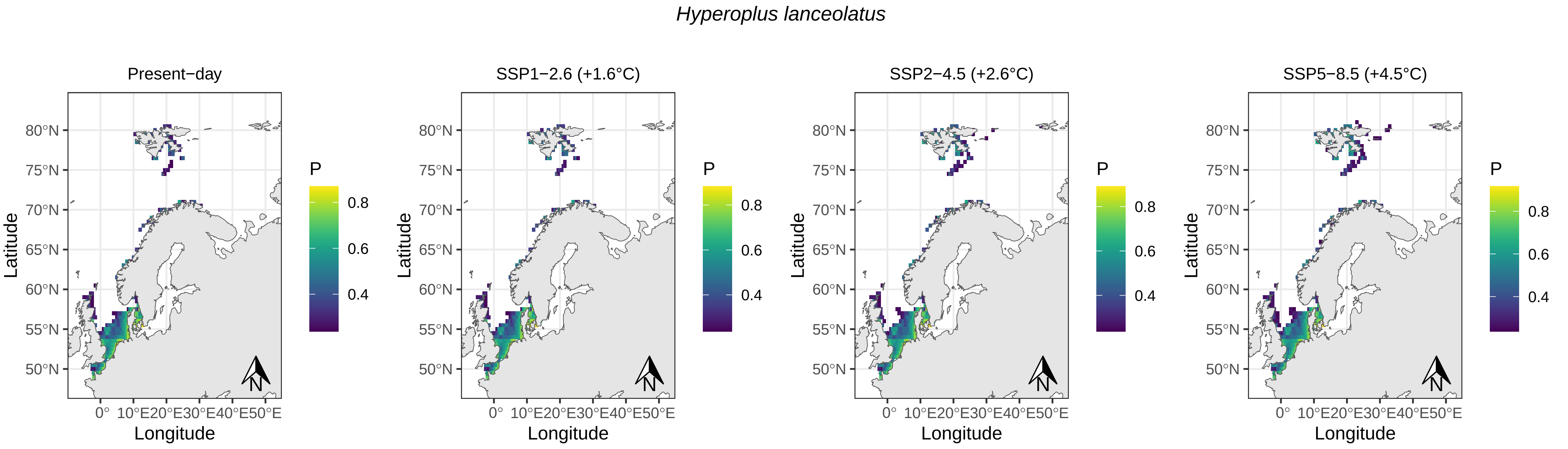
Hyperoplus lanceolatus
Present−day
SSP1−2.6 (+1.6°C)
SSP2−4.5 (+2.6°C)
SSP5−8.5 (+4.5°C)
Latitude
80°N
75°N
70°N
65°N
60°N
55°N
50°N
Longitude
0°
10°E
20°E
30°E
40°E
50°E
P
0.8
0.6
0.4
N

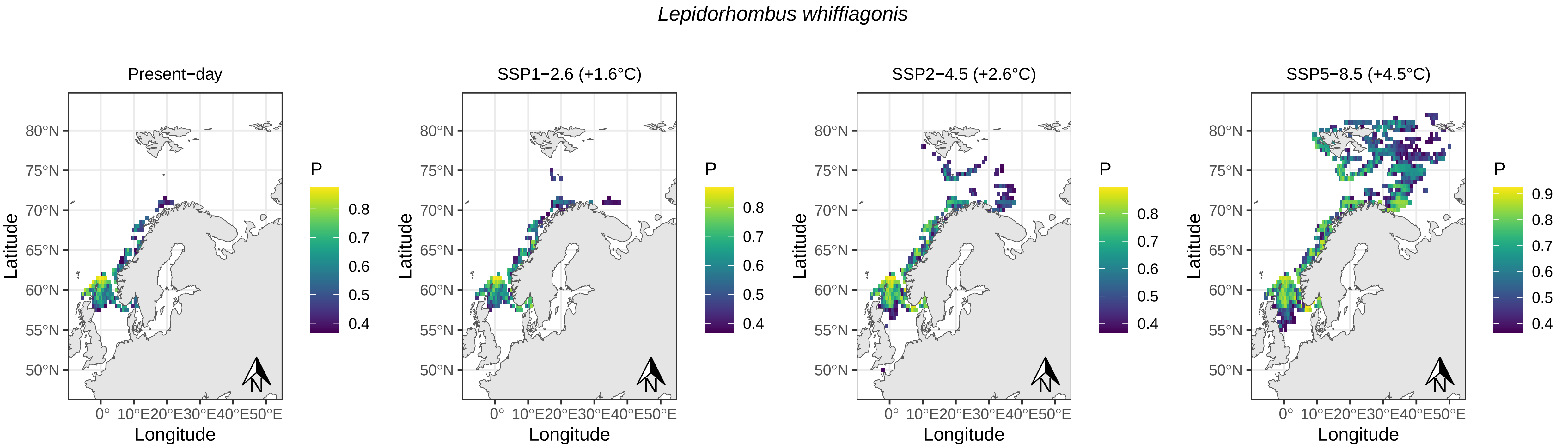

Lepidorhombus whiffiagonis
Present−day
SSP1−2.6 (+1.6°C)
SSP2−4.5 (+2.6°C)
SSP5−8.5 (+4.5°C)
Latitude
Longitude
80°N
75°N
70°N
65°N
60°N
55°N
50°N
0° 10°E 20°E 30°E 40°E 50°E
P
0.9
0.8
0.7
0.6
0.5
0.4
N

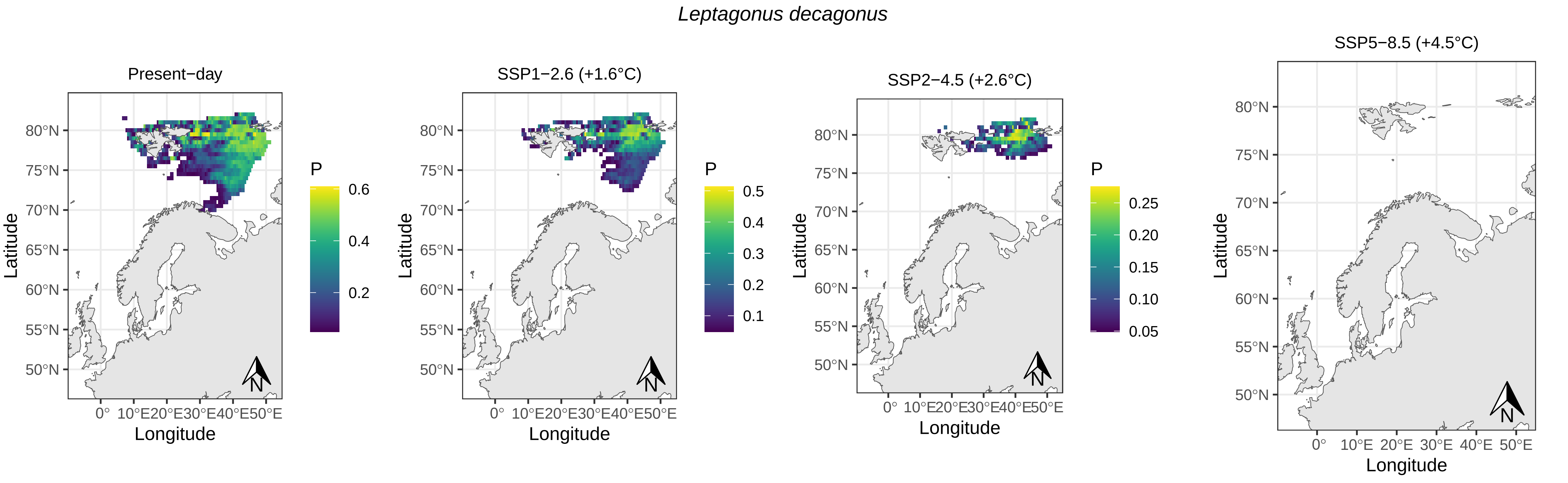

Leptagonus decagonus
Present−day
SSP1−2.6 (+1.6°C)
SSP2−4.5 (+2.6°C)
SSP5−8.5 (+4.5°C)
Latitude
Longitude
80°N
75°N
70°N
65°N
60°N
55°N
50°N
0°
10°E
20°E
30°E
40°E
50°E
P
0.6
0.4
0.2
0.5
0.4
0.3
0.2
0.1
0.25
0.20
0.15
0.10
0.05
N

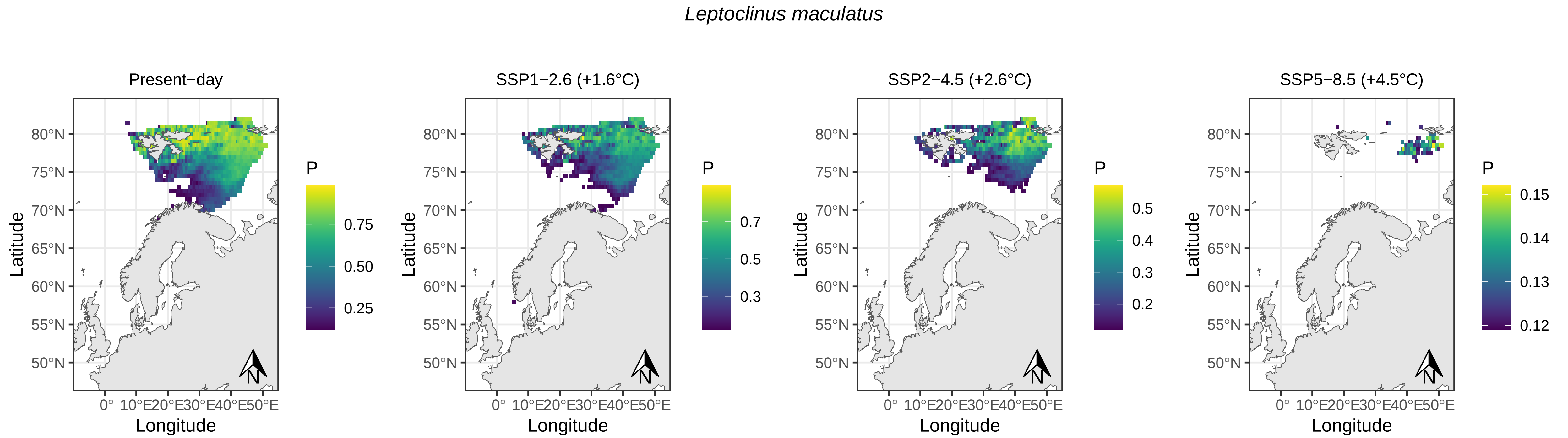
Leptoclinus maculatus
Present−day
SSP1−2.6 (+1.6°C)
SSP2−4.5 (+2.6°C)
SSP5−8.5 (+4.5°C)
80°N
75°N
70°N
65°N
60°N
55°N
50°N
Latitude
0° 10°E 20°E 30°E 40°E 50°E
Longitude
P
0.75
0.50
0.25
0.7
0.5
0.3
0.5
0.4
0.3
0.2
0.15
0.14
0.13
0.12
N

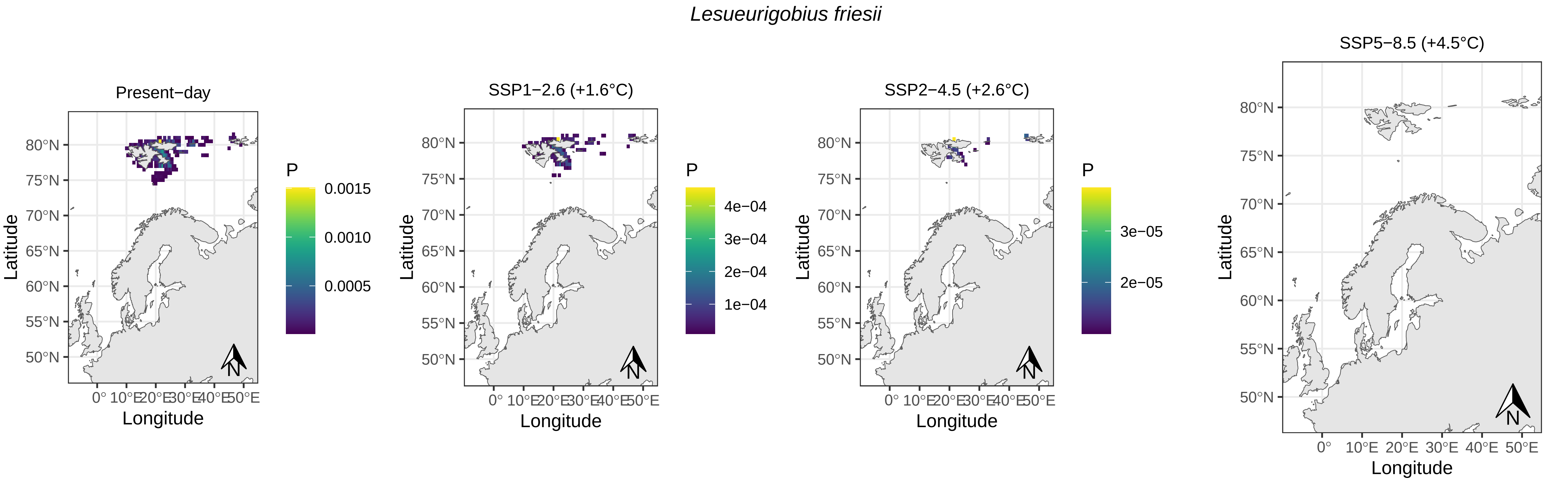

*Lesueurigobius friesii*
Present−day
SSP1−2.6 (+1.6°C)
SSP2−4.5 (+2.6°C)
SSP5−8.5 (+4.5°C)
Latitude
Longitude
80°N
75°N
70°N
65°N
60°N
55°N
50°N
0°
10°E
20°E
30°E
40°E
50°E
P
0.0015
0.0010
0.0005
4e−04
3e−04
2e−04
1e−04
3e−05
2e−05
N

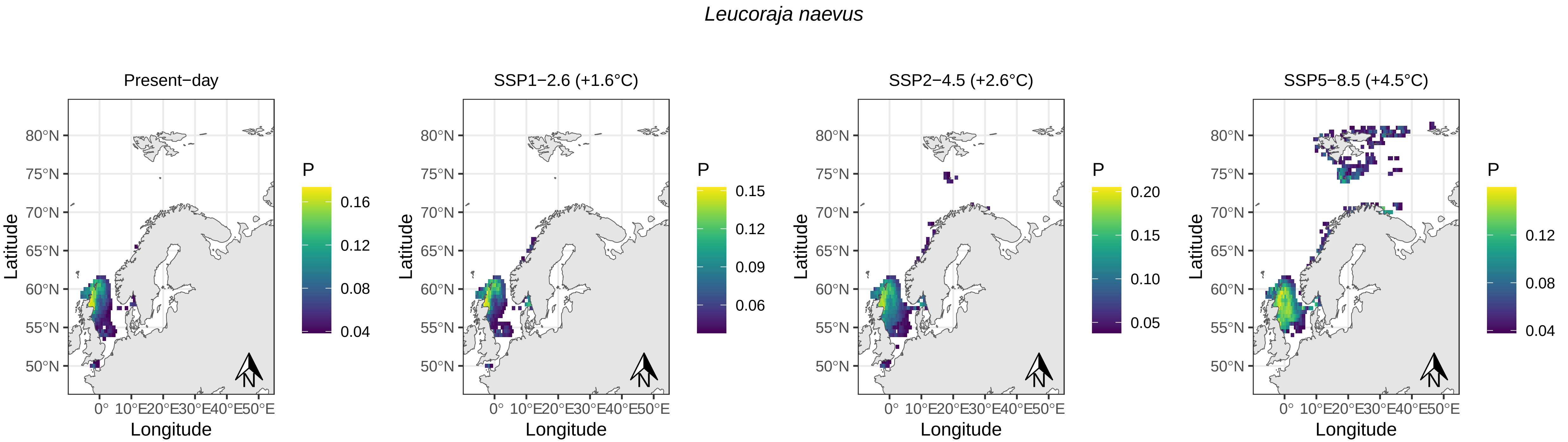

Leucoraja naevus
Present−day
SSP1−2.6 (+1.6°C)
SSP2−4.5 (+2.6°C)
SSP5−8.5 (+4.5°C)
Latitude
Longitude
80°N
75°N
70°N
65°N
60°N
55°N
50°N
0° 10°E 20°E 30°E 40°E 50°E
P
0.16
0.12
0.08
0.04
0.15
0.12
0.09
0.06
0.20
0.15
0.10
0.05
0.12
0.08
0.04
N

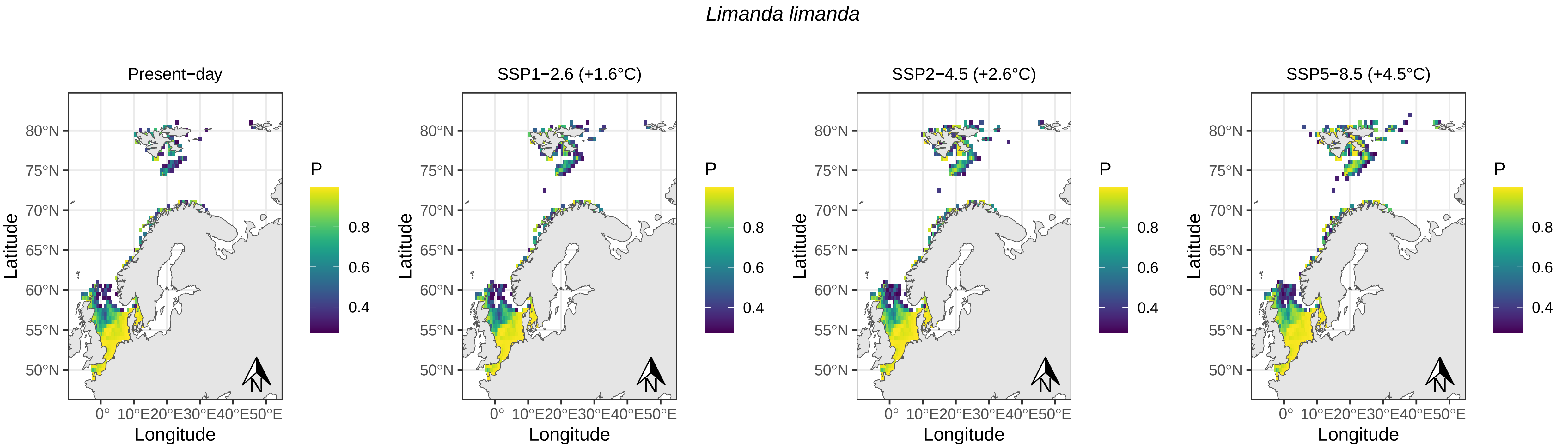
Limanda limanda
Present−day
SSP1−2.6 (+1.6°C)
SSP2−4.5 (+2.6°C)
SSP5−8.5 (+4.5°C)
80°N
75°N
70°N
65°N
60°N
55°N
50°N
Latitude
0°
10°E
20°E
30°E
40°E
50°E
Longitude
P
0.8
0.6
0.4
N

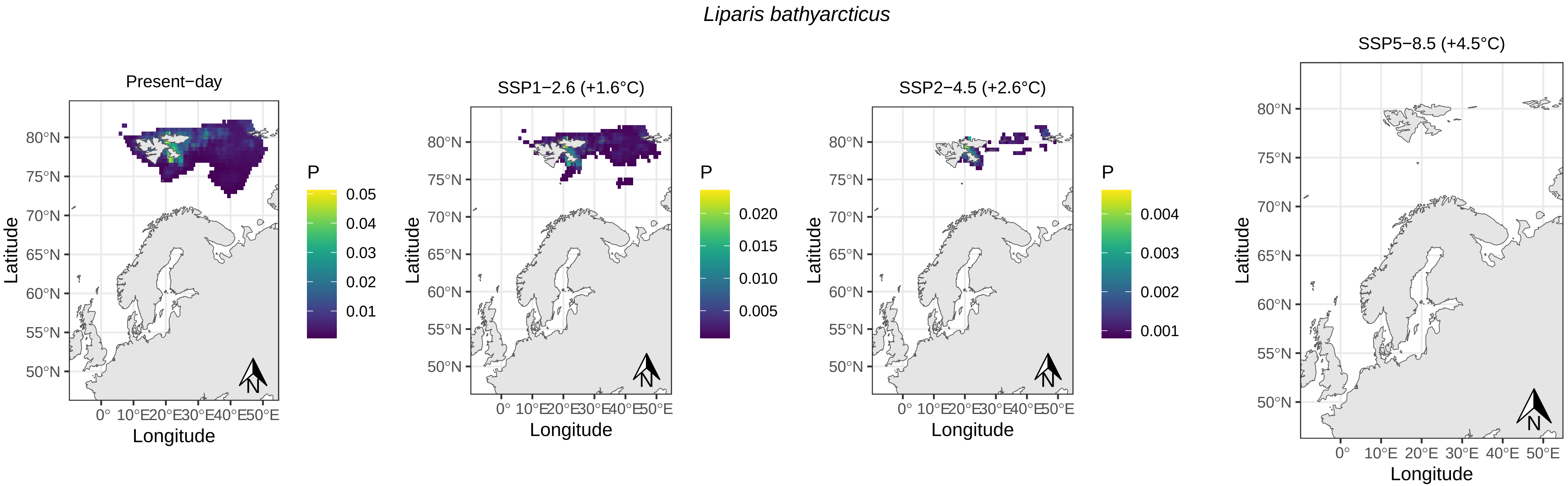

Liparis bathyarcticus
Present−day
SSP1−2.6 (+1.6°C)
SSP2−4.5 (+2.6°C)
SSP5−8.5 (+4.5°C)
Latitude
Longitude
80°N
75°N
70°N
65°N
60°N
55°N
50°N
0°
10°E
20°E
30°E
40°E
50°E
P
0.05
0.04
0.03
0.02
0.01
0.020
0.015
0.010
0.005
0.004
0.003
0.002
0.001
N

*Liparis fabricii*

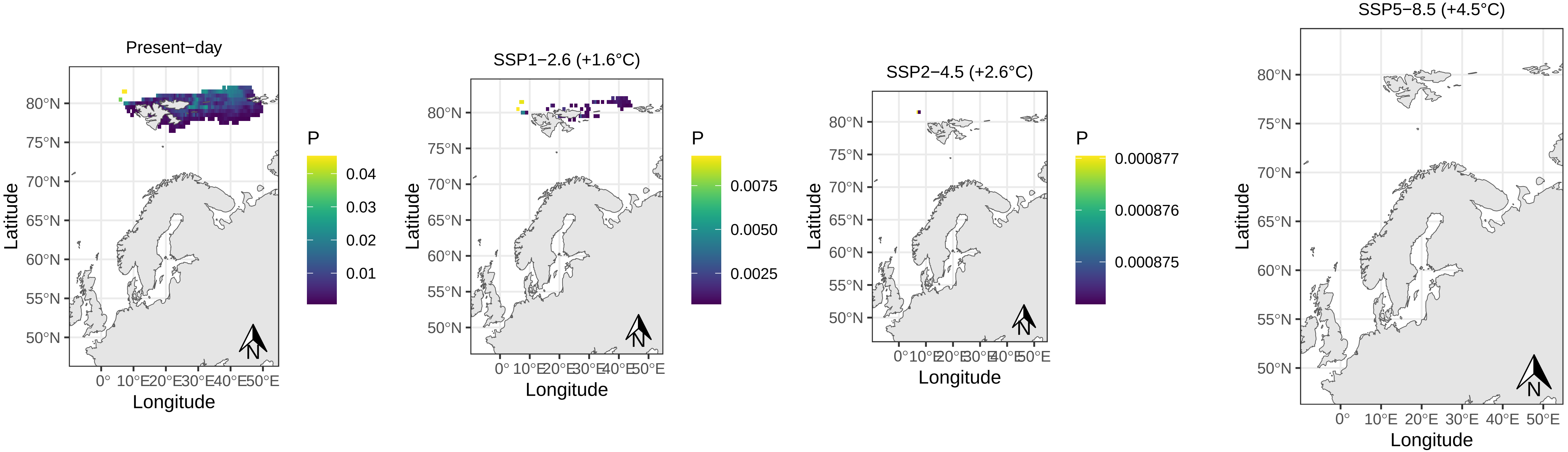

*Lophius budegassa*

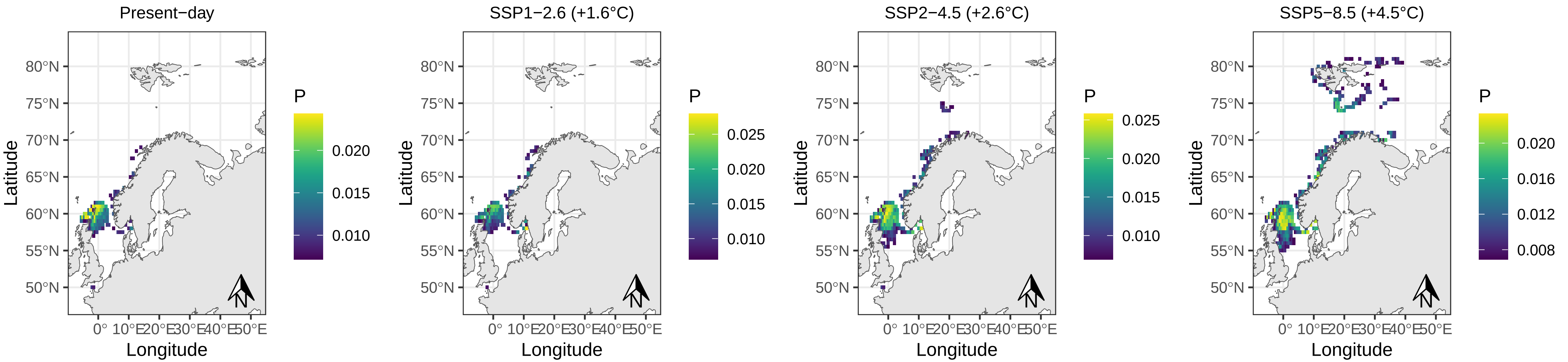

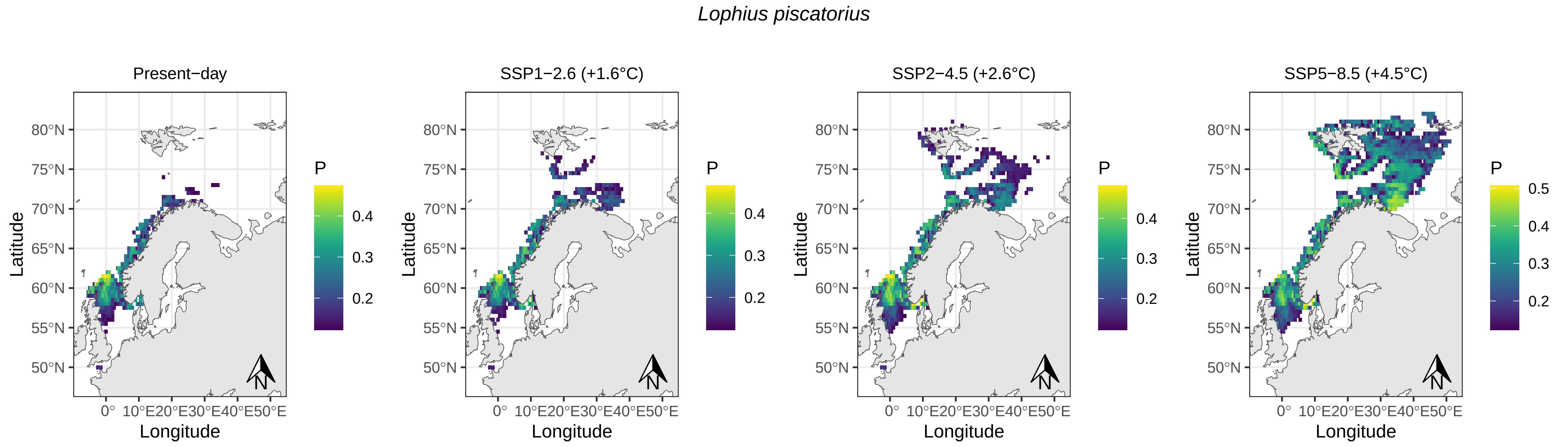
Lophius piscatorius
Present−day
SSP1−2.6 (+1.6°C)
SSP2−4.5 (+2.6°C)
SSP5−8.5 (+4.5°C)
Latitude
Longitude
80°N
75°N
70°N
65°N
60°N
55°N
50°N
0°
10°E
20°E
30°E
40°E
50°E
P
0.2
0.3
0.4
0.5
N

*Lumpenus lampretaeformis*

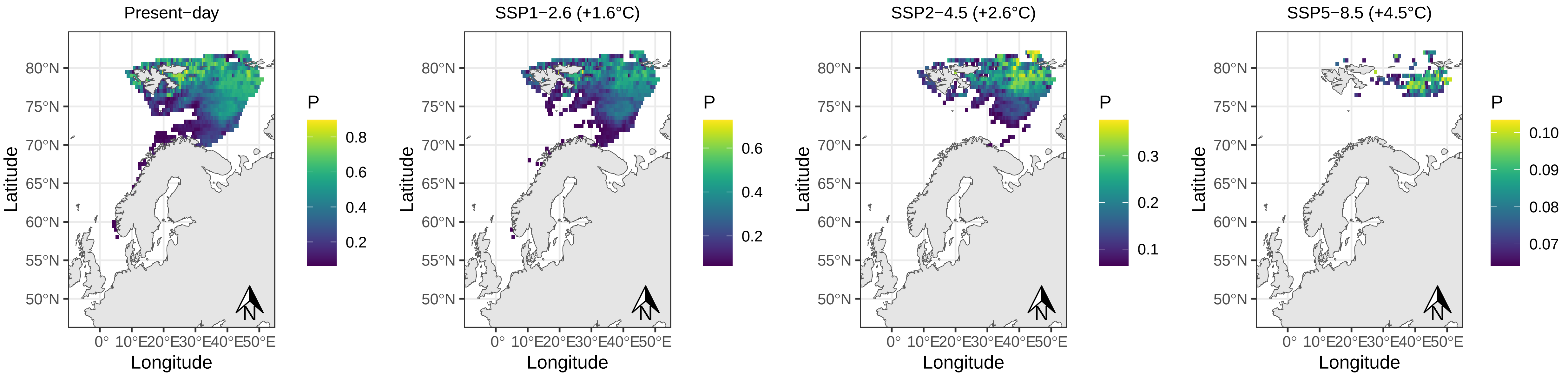

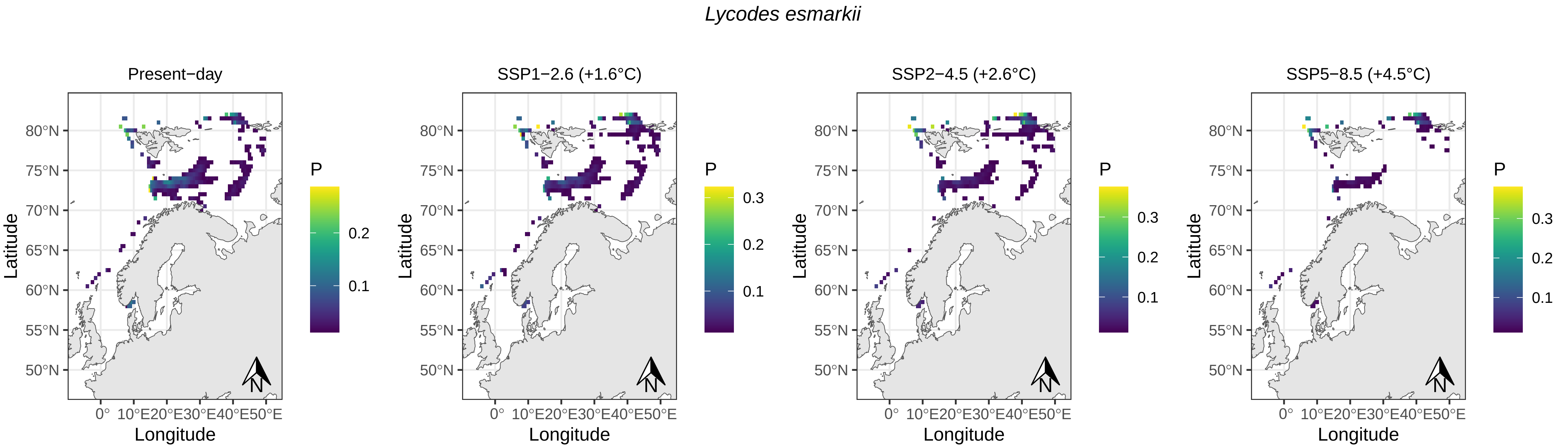

Lycodes esmarkii
Present−day
SSP1−2.6 (+1.6°C)
SSP2−4.5 (+2.6°C)
SSP5−8.5 (+4.5°C)
Latitude
Longitude
80°N
75°N
70°N
65°N
60°N
55°N
50°N
0°
10°E
20°E
30°E
40°E
50°E
P
0.1
0.2
0.3
N

*Lycodes eudipleurostictus*

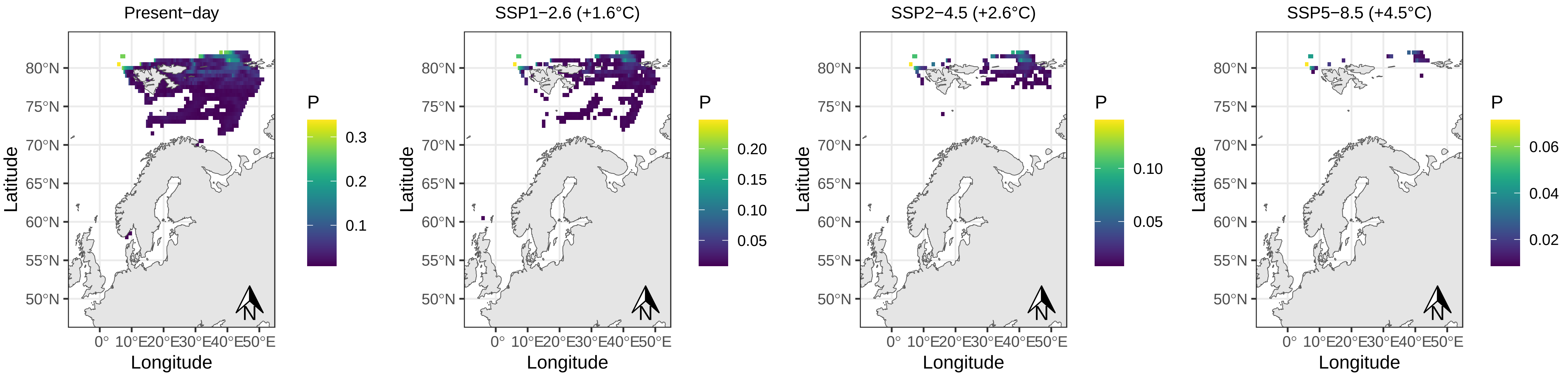

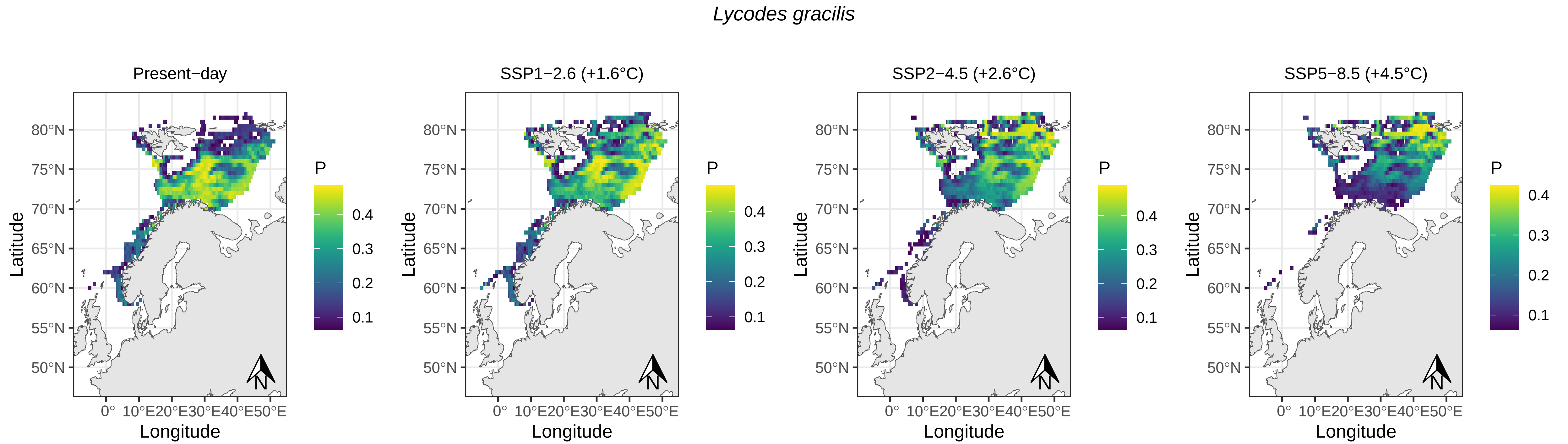
Lycodes gracilis
Present−day
SSP1−2.6 (+1.6°C)
SSP2−4.5 (+2.6°C)
SSP5−8.5 (+4.5°C)
80°N
75°N
70°N
65°N
60°N
55°N
50°N
Latitude
0°
10°E
20°E
30°E
40°E
50°E
Longitude
P
0.4
0.3
0.2
0.1
N

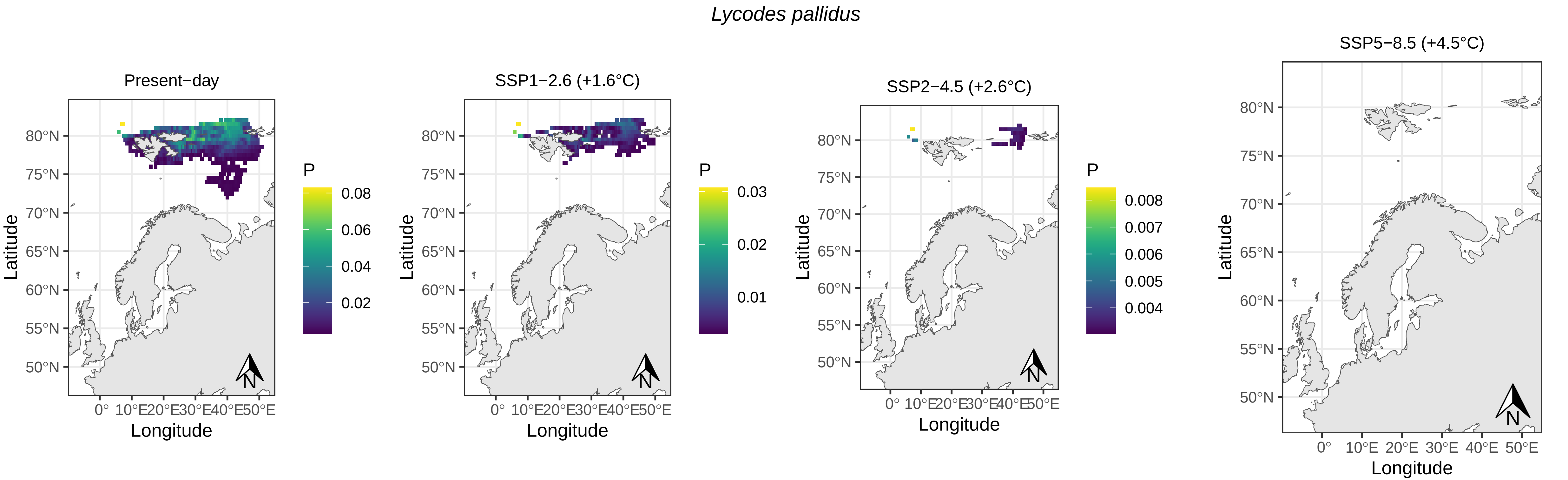

Lycodes pallidus
Present−day
SSP1−2.6 (+1.6°C)
SSP2−4.5 (+2.6°C)
SSP5−8.5 (+4.5°C)
P
0.08
0.06
0.04
0.02
0.03
0.02
0.01
0.008
0.007
0.006
0.005
0.004
Latitude
Longitude
80°N
75°N
70°N
65°N
60°N
55°N
50°N
0°
10°E
20°E
30°E
40°E
50°E
N

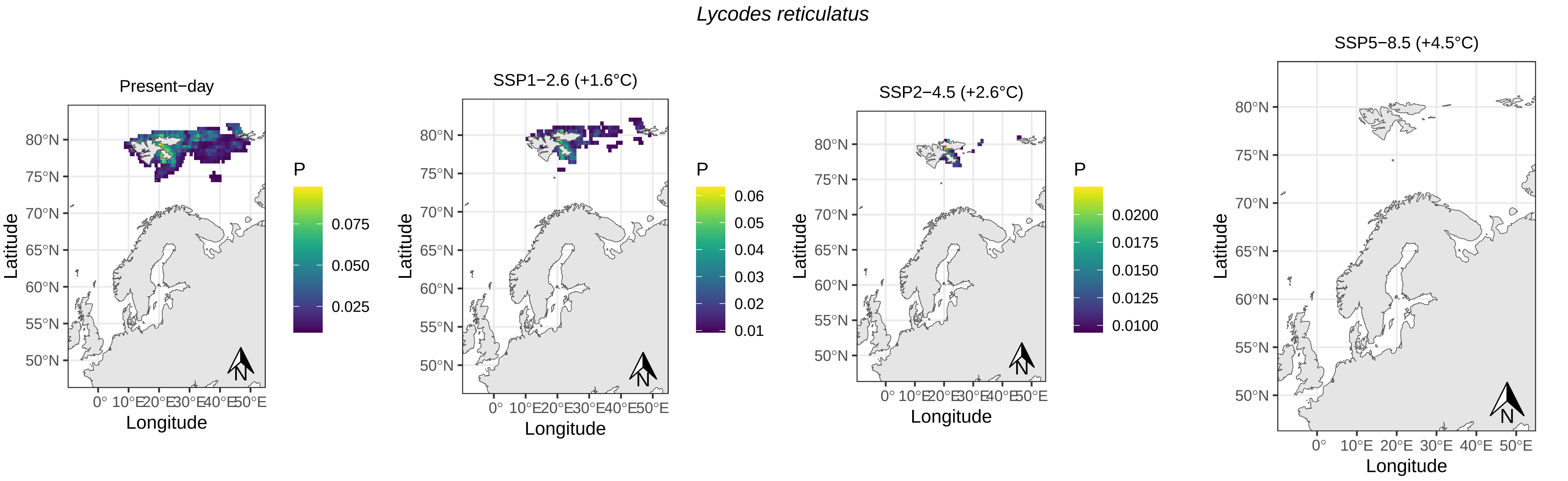

Lycodes reticulatus
Present−day
SSP1−2.6 (+1.6°C)
SSP2−4.5 (+2.6°C)
SSP5−8.5 (+4.5°C)
Latitude
Longitude
80°N
75°N
70°N
65°N
60°N
55°N
50°N
0°
10°E
20°E
30°E
40°E
50°E
P
0.075
0.050
0.025
0.06
0.05
0.04
0.03
0.02
0.01
0.0200
0.0175
0.0150
0.0125
0.0100
N

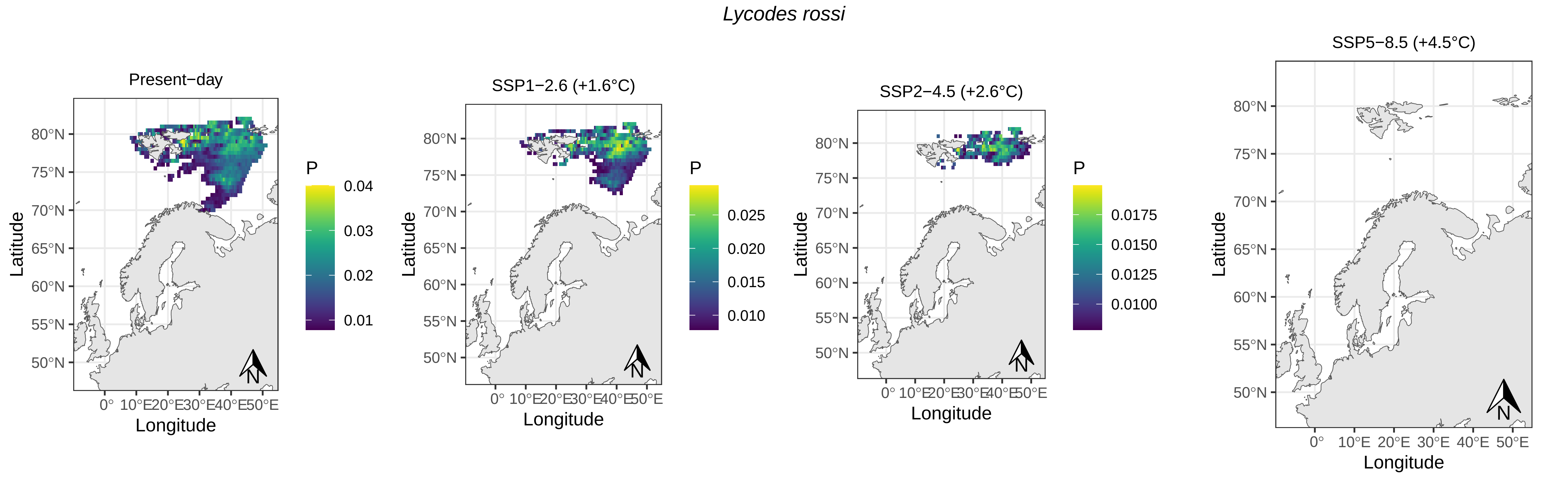

Lycodes rossi
Present−day
SSP1−2.6 (+1.6°C)
SSP2−4.5 (+2.6°C)
SSP5−8.5 (+4.5°C)
Latitude
Longitude
80°N
75°N
70°N
65°N
60°N
55°N
50°N
0°
10°E
20°E
30°E
40°E
50°E
P
0.04
0.03
0.02
0.01
0.025
0.020
0.015
0.010
0.0175
0.0150
0.0125
0.0100
N

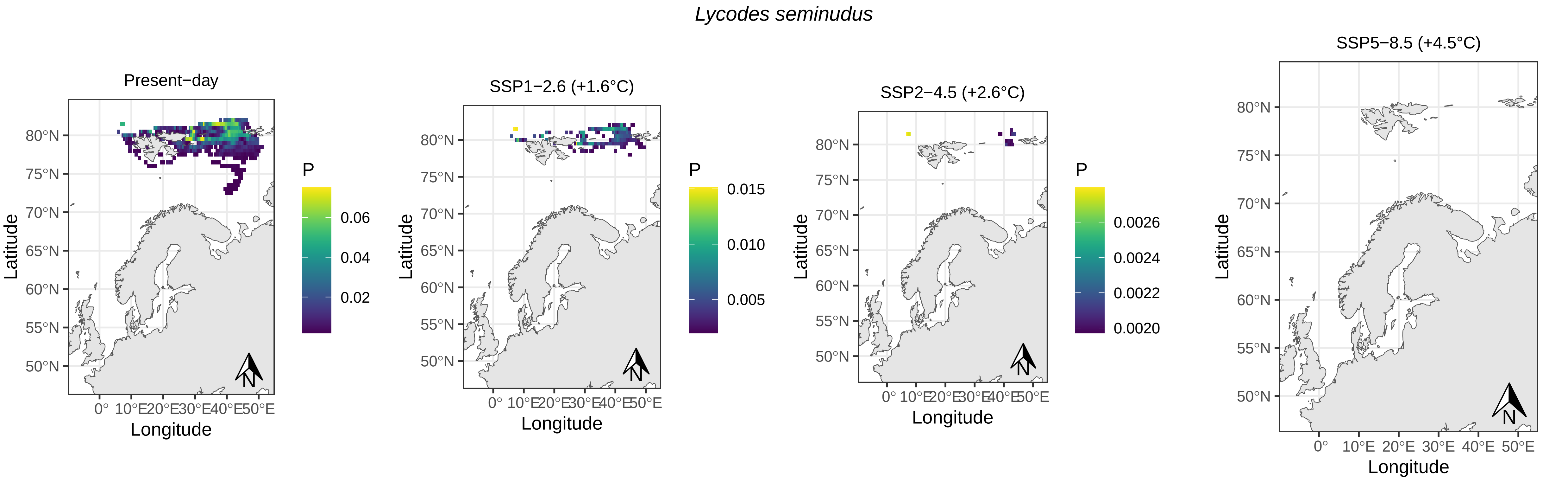
Lycodes seminudus
Present−day
SSP1−2.6 (+1.6°C)
SSP2−4.5 (+2.6°C)
SSP5−8.5 (+4.5°C)
Latitude
Longitude
80°N
75°N
70°N
65°N
60°N
55°N
50°N
0°
10°E
20°E
30°E
40°E
50°E
P
0.06
0.04
0.02
0.015
0.010
0.005
0.0026
0.0024
0.0022
0.0020
N

*Lycodes vahlii*

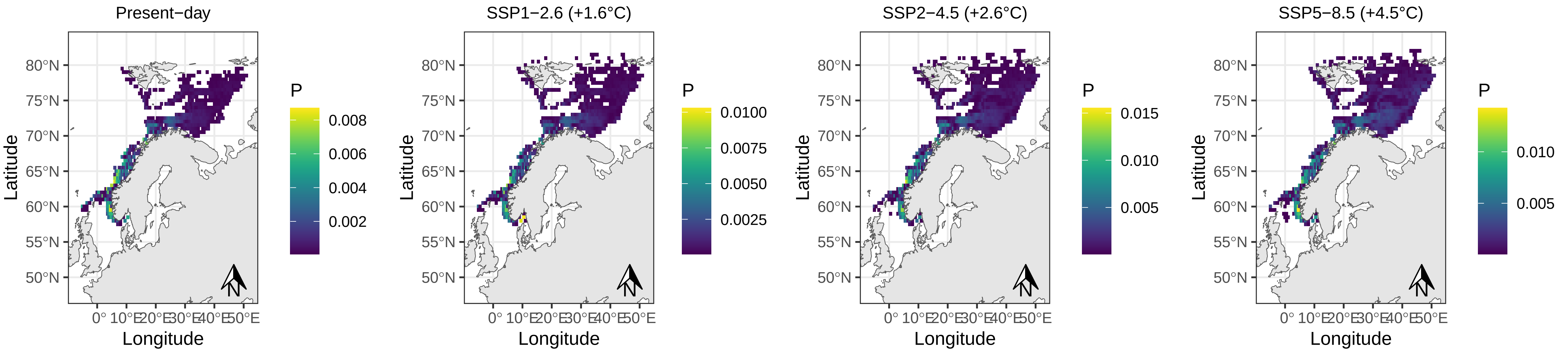

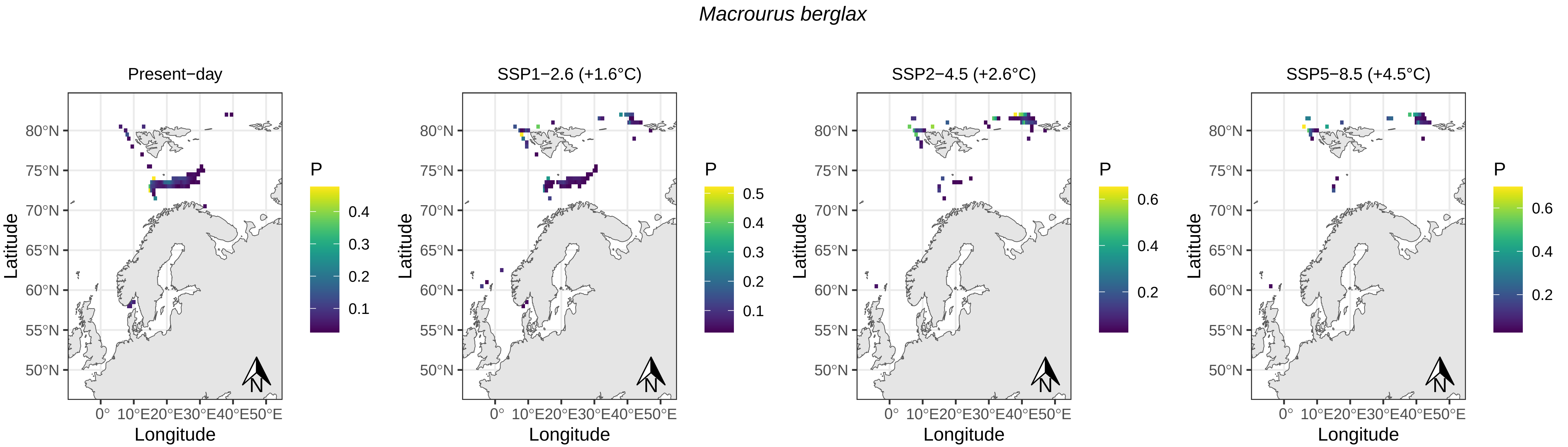
Macrourus berglax
Present−day
SSP1−2.6 (+1.6°C)
SSP2−4.5 (+2.6°C)
SSP5−8.5 (+4.5°C)
80°N
75°N
70°N
65°N
60°N
55°N
50°N
Latitude
0°
10°E
20°E
30°E
40°E
50°E
Longitude
N
P
0.1
0.2
0.3
0.4
0.5
0.6

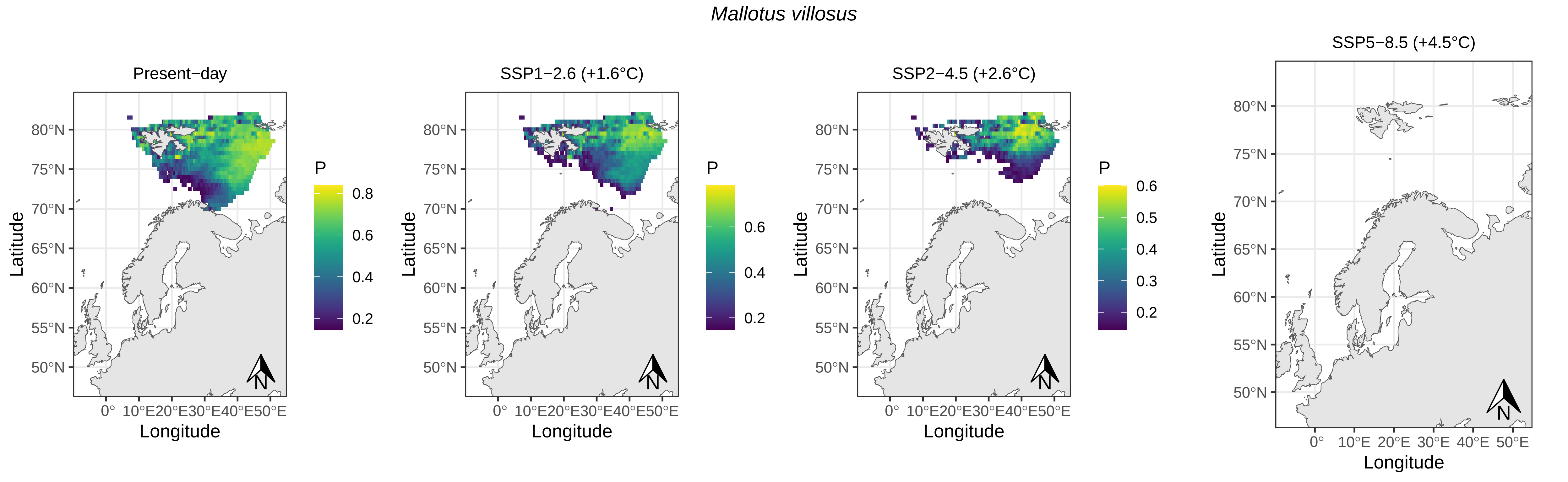

*Mallotus villosus*
Present−day
SSP1−2.6 (+1.6°C)
SSP2−4.5 (+2.6°C)
SSP5−8.5 (+4.5°C)
Latitude
Longitude
80°N
75°N
70°N
65°N
60°N
55°N
50°N
0°
10°E
20°E
30°E
40°E
50°E
P
0.8
0.6
0.4
0.2
0.6
0.4
0.2
0.6
0.5
0.4
0.3
0.2
N

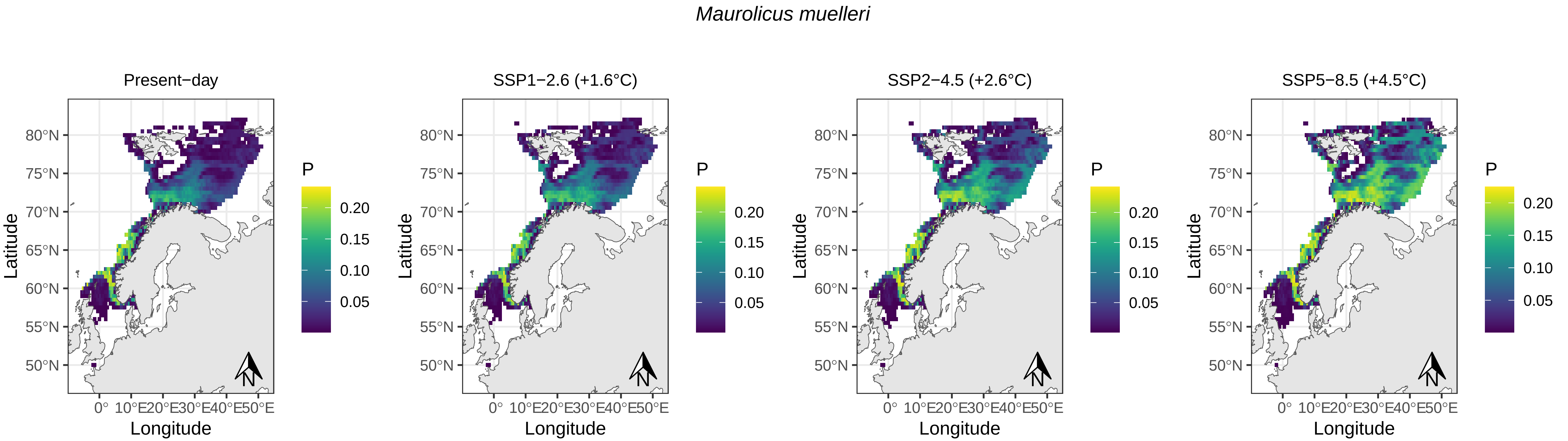

Maurolicus muelleri
Present−day
SSP1−2.6 (+1.6°C)
SSP2−4.5 (+2.6°C)
SSP5−8.5 (+4.5°C)
Latitude
Longitude
P
0.20
0.15
0.10
0.05
80°N
75°N
70°N
65°N
60°N
55°N
50°N
0° 10°E20°E30°E40°E50°E
N

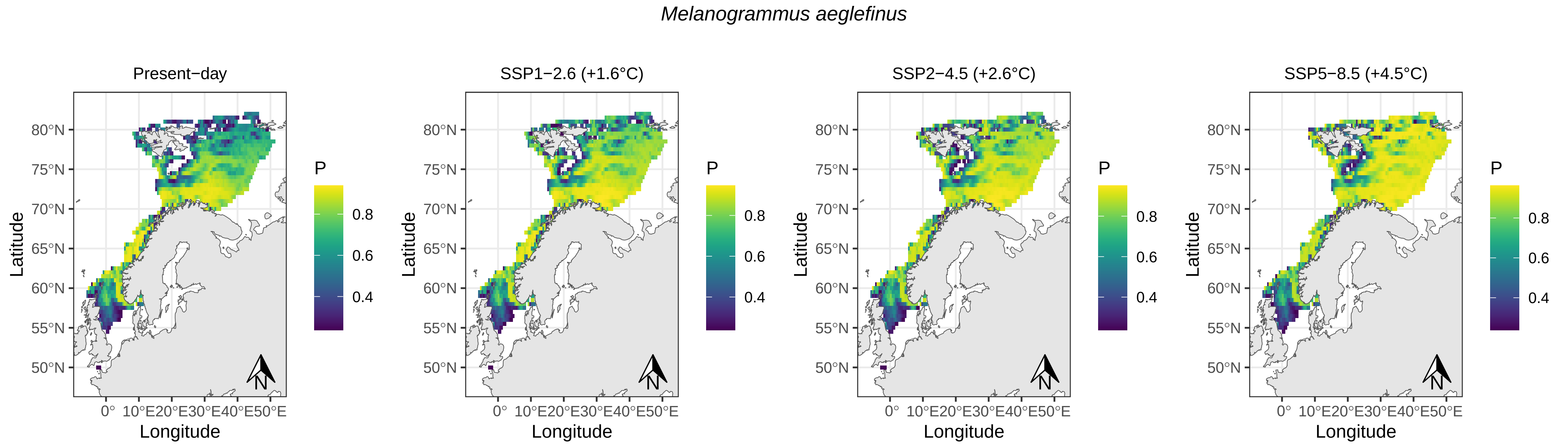
Melanogrammus aeglefinus
Present−day
SSP1−2.6 (+1.6°C)
SSP2−4.5 (+2.6°C)
SSP5−8.5 (+4.5°C)
Latitude
Longitude
80°N
75°N
70°N
65°N
60°N
55°N
50°N
0° 10°E 20°E 30°E 40°E 50°E
P
0.8
0.6
0.4
N

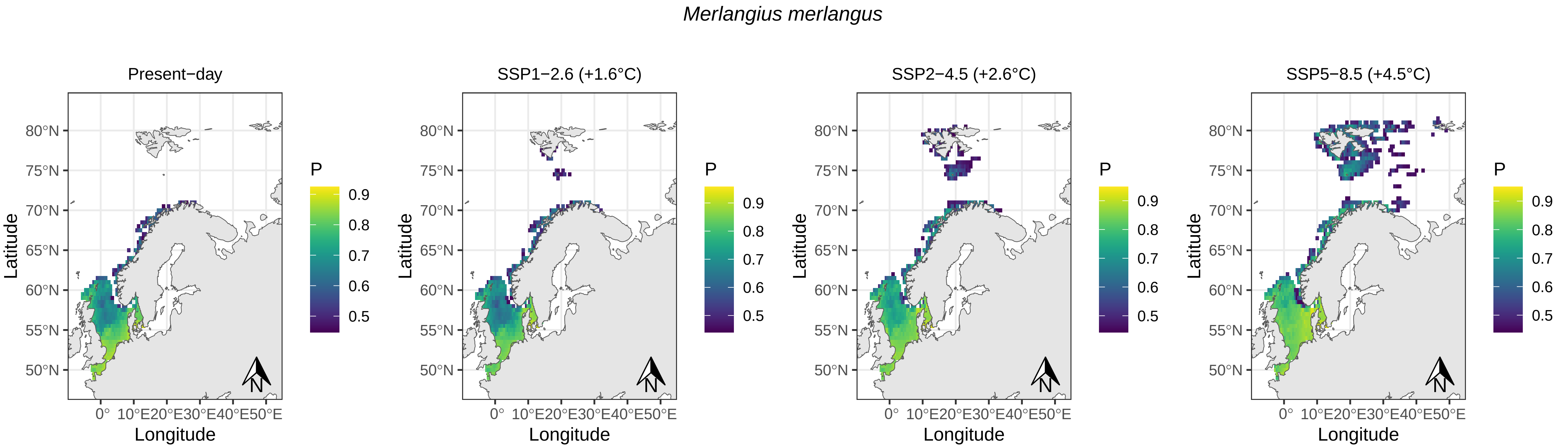

Merlangius merlangus
Present−day
SSP1−2.6 (+1.6°C)
SSP2−4.5 (+2.6°C)
SSP5−8.5 (+4.5°C)
Latitude
Longitude
80°N
75°N
70°N
65°N
60°N
55°N
50°N
0°
10°E
20°E
30°E
40°E
50°E
P
0.9
0.8
0.7
0.6
0.5
N

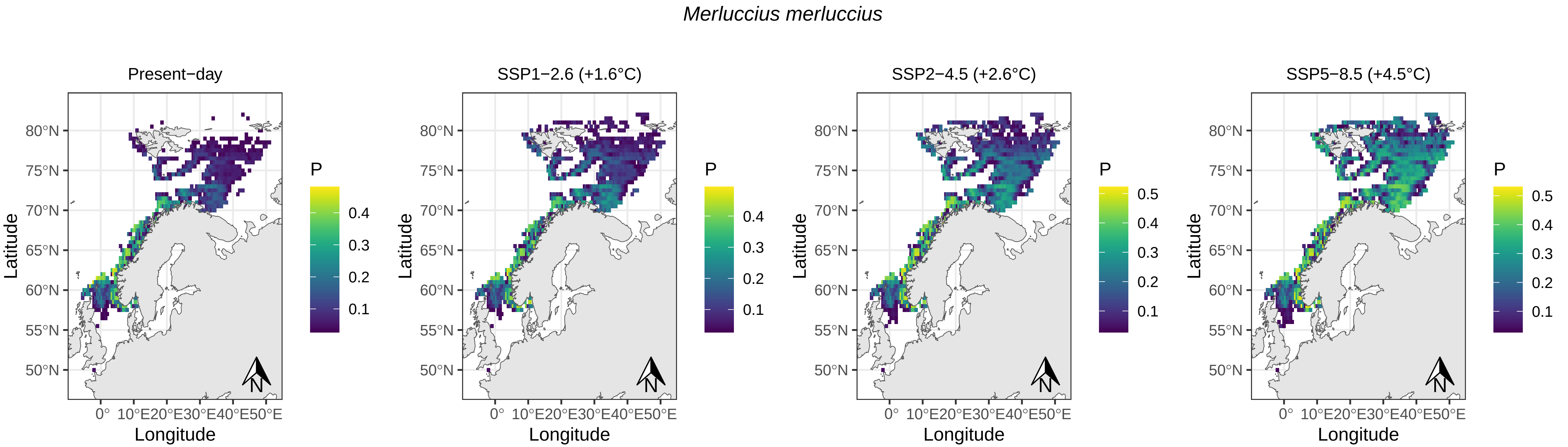

Merluccius merluccius
Present−day
SSP1−2.6 (+1.6°C)
SSP2−4.5 (+2.6°C)
SSP5−8.5 (+4.5°C)
Latitude
Longitude
80°N
75°N
70°N
65°N
60°N
55°N
50°N
0°
10°E
20°E
30°E
40°E
50°E
P
0.5
0.4
0.3
0.2
0.1
N

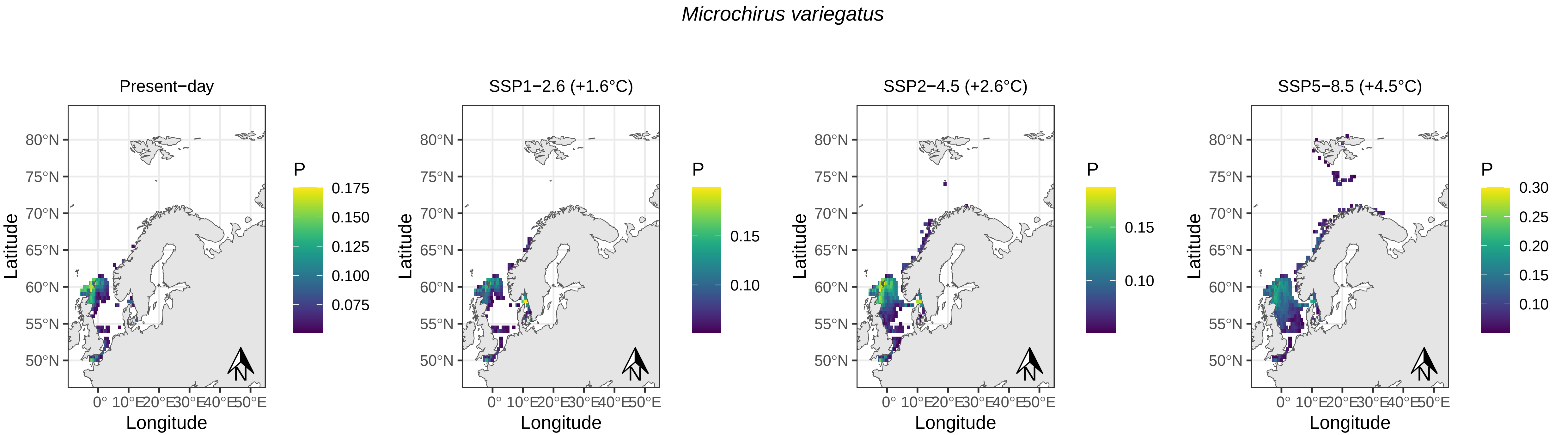
Microchirus variegatus
Present−day
SSP1−2.6 (+1.6°C)
SSP2−4.5 (+2.6°C)
SSP5−8.5 (+4.5°C)
Latitude
Longitude
80°N
75°N
70°N
65°N
60°N
55°N
50°N
0° 10°E 20°E 30°E 40°E 50°E
P
0.175
0.150
0.125
0.100
0.075
0.15
0.10
0.30
0.25
0.20
N

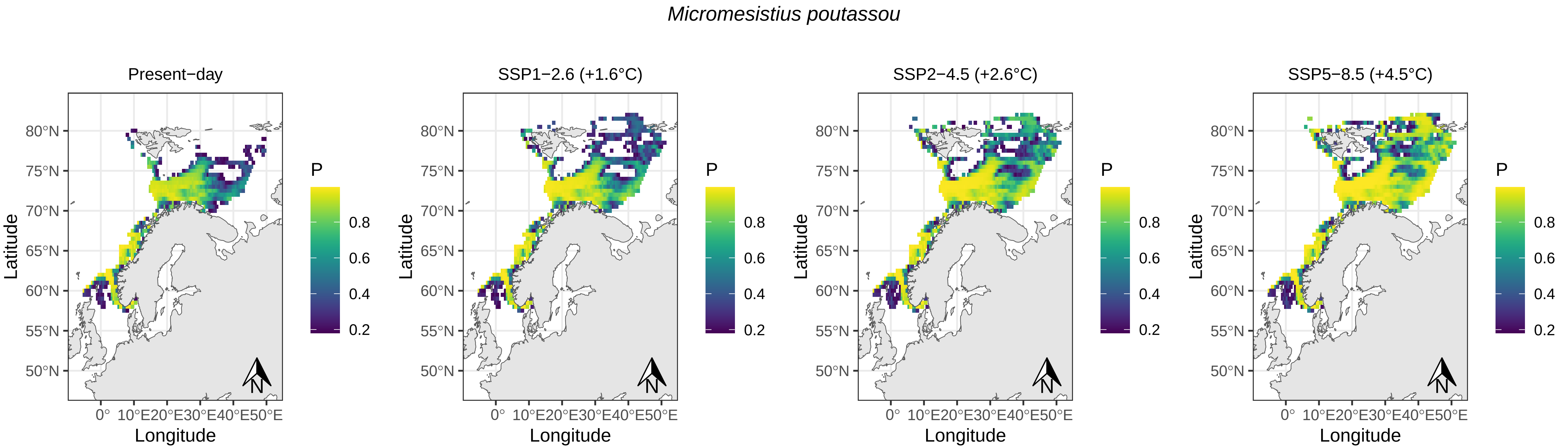

*Micromesistius poutassou*
Present−day
SSP1−2.6 (+1.6°C)
SSP2−4.5 (+2.6°C)
SSP5−8.5 (+4.5°C)
80°N
75°N
70°N
65°N
60°N
55°N
50°N
Latitude
0°
10°E
20°E
30°E
40°E
50°E
Longitude
P
0.8
0.6
0.4
0.2
N

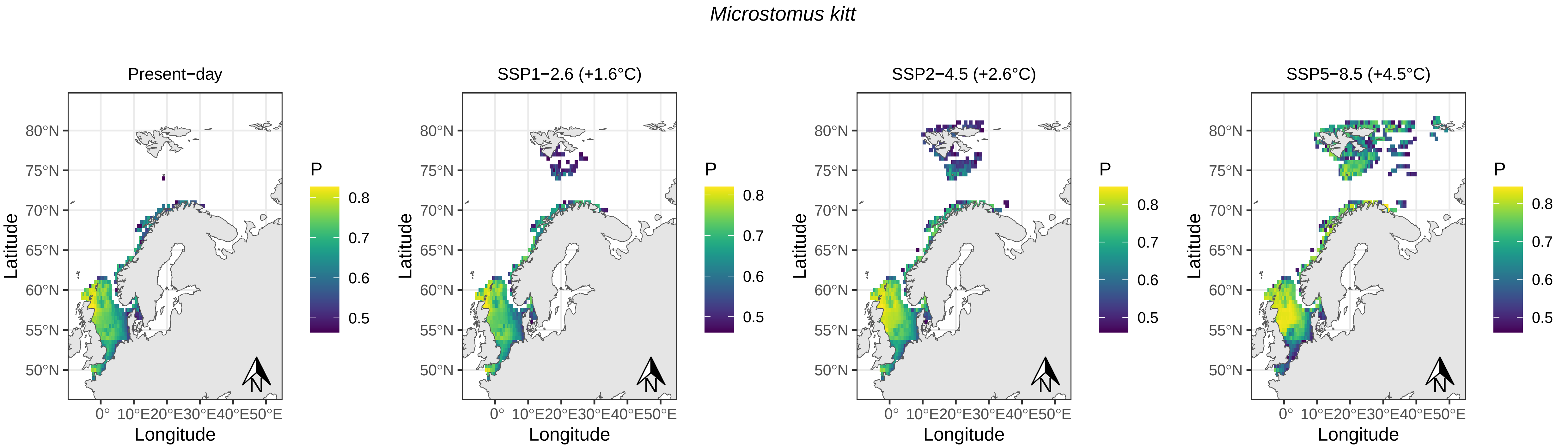

Microstomus kitt
Present−day
SSP1−2.6 (+1.6°C)
SSP2−4.5 (+2.6°C)
SSP5−8.5 (+4.5°C)
Latitude
Longitude
80°N
75°N
70°N
65°N
60°N
55°N
50°N
0°
10°E
20°E
30°E
40°E
50°E
P
0.8
0.7
0.6
0.5
N

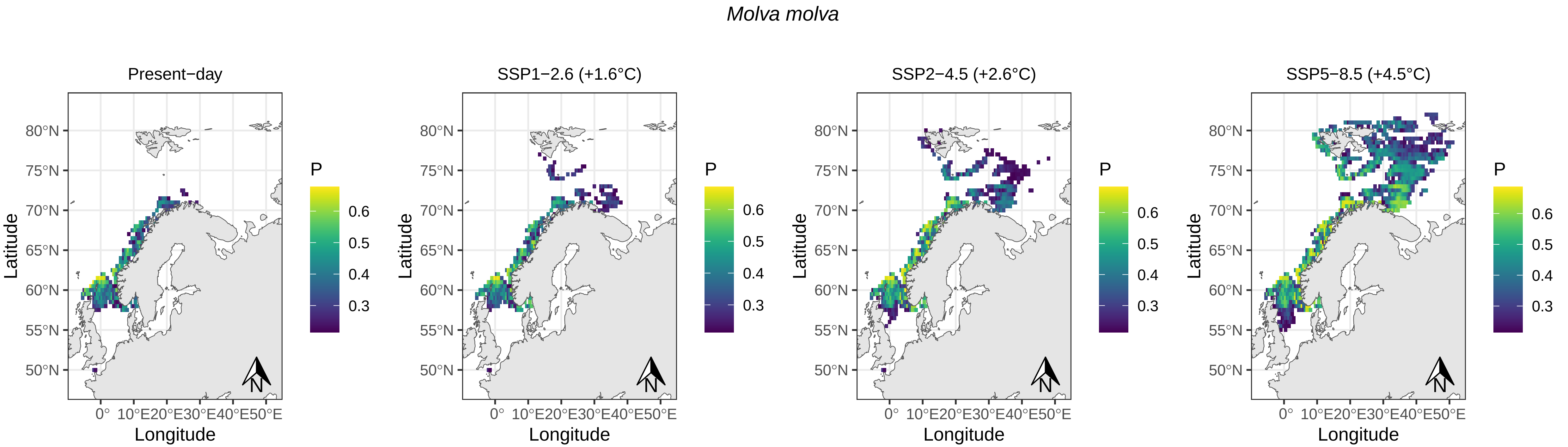
Molva molva
Present−day
SSP1−2.6 (+1.6°C)
SSP2−4.5 (+2.6°C)
SSP5−8.5 (+4.5°C)
Latitude
Longitude
80°N
75°N
70°N
65°N
60°N
55°N
50°N
0°
10°E
20°E
30°E
40°E
50°E
P
0.6
0.5
0.4
0.3
N

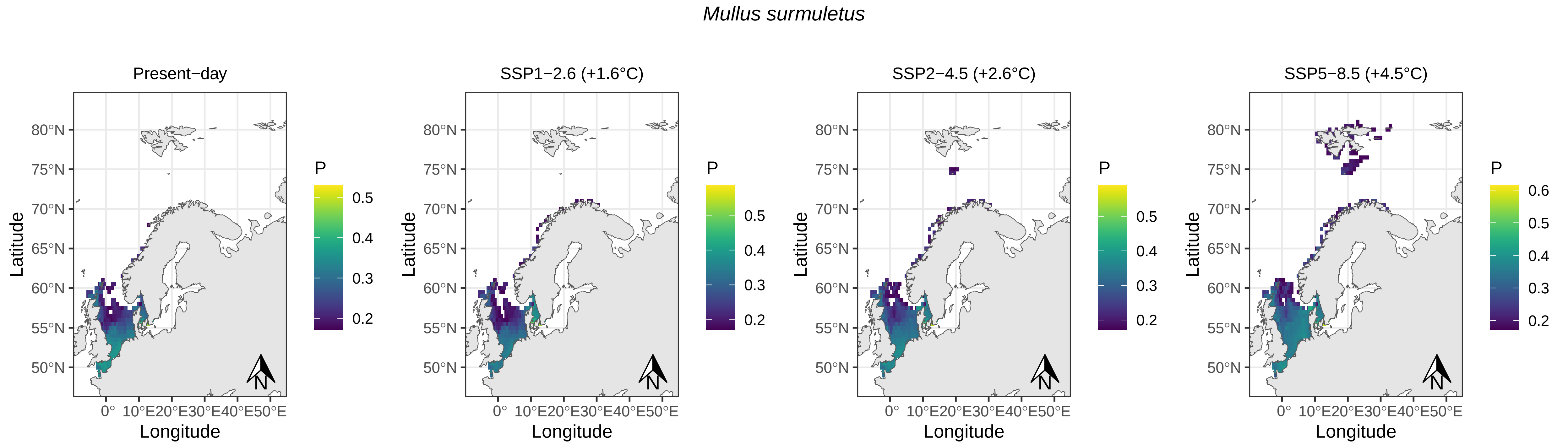
Mullus surmuletus
Present−day
SSP1−2.6 (+1.6°C)
SSP2−4.5 (+2.6°C)
SSP5−8.5 (+4.5°C)
Latitude
Longitude
80°N
75°N
70°N
65°N
60°N
55°N
50°N
0°
10°E
20°E
30°E
40°E
50°E
P
0.2
0.3
0.4
0.5
0.6
N

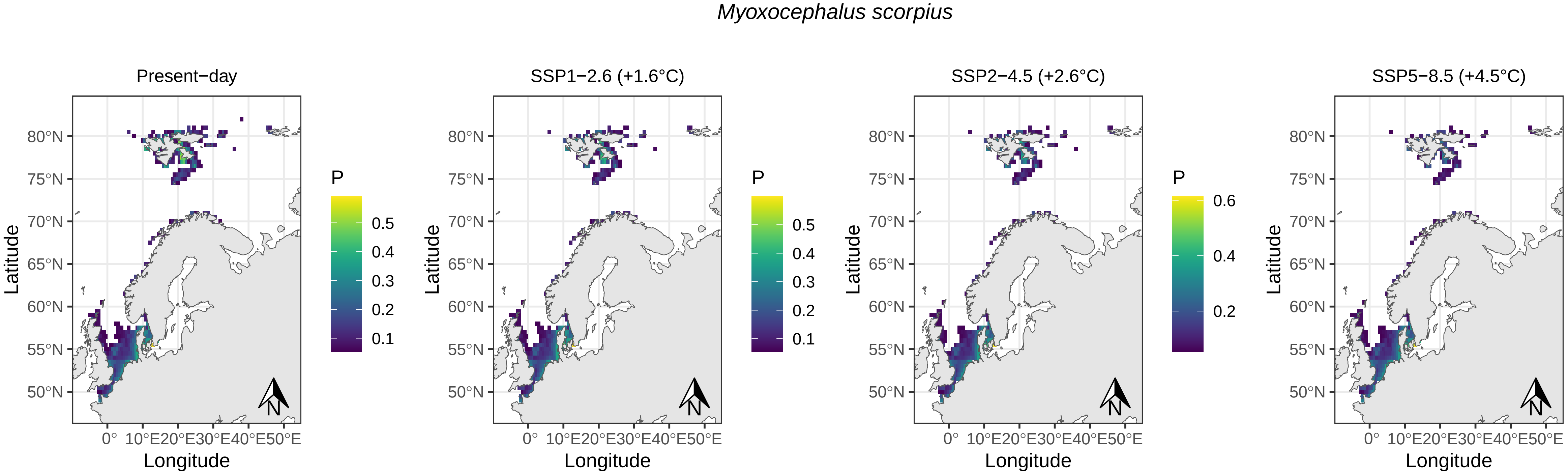

Myoxocephalus scorpius
Present−day
SSP1−2.6 (+1.6°C)
SSP2−4.5 (+2.6°C)
SSP5−8.5 (+4.5°C)
80°N
75°N
70°N
65°N
60°N
55°N
50°N
Latitude
0°
10°E
20°E
30°E
40°E
50°E
Longitude
P
0.1
0.2
0.3
0.4
0.5
0.6
N

*Myxine glutinosa*

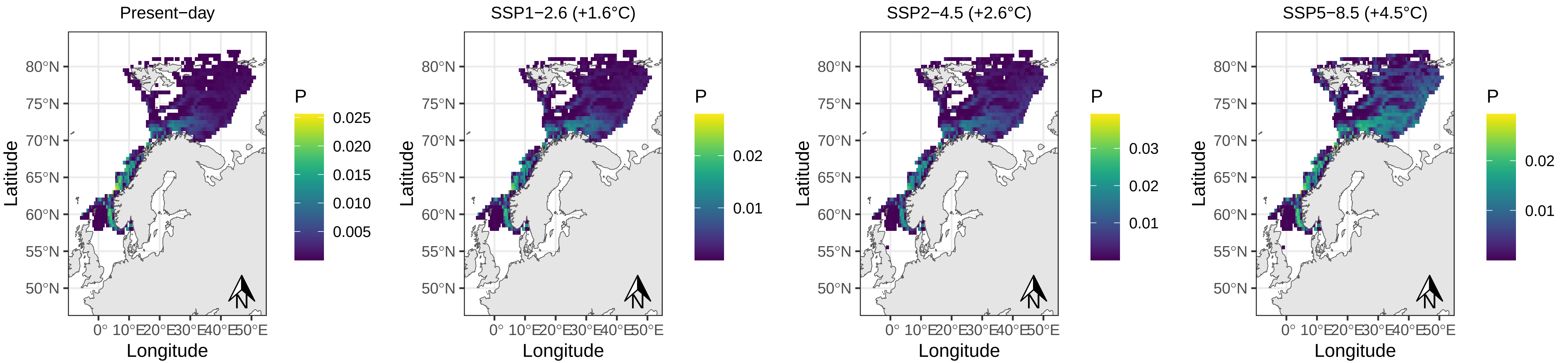

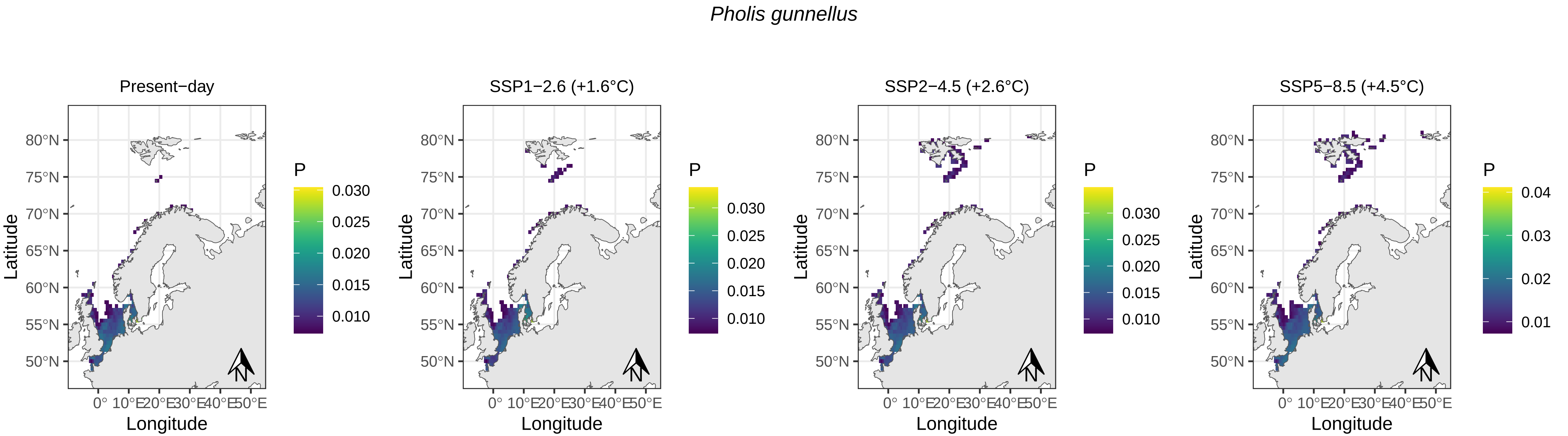

Pholis gunnellus
Present−day
SSP1−2.6 (+1.6°C)
SSP2−4.5 (+2.6°C)
SSP5−8.5 (+4.5°C)
Latitude
Longitude
80°N
75°N
70°N
65°N
60°N
55°N
50°N
0° 10°E 20°E 30°E 40°E 50°E
P
0.030
0.025
0.020
0.015
0.010
0.04
0.03
0.02
0.01
N

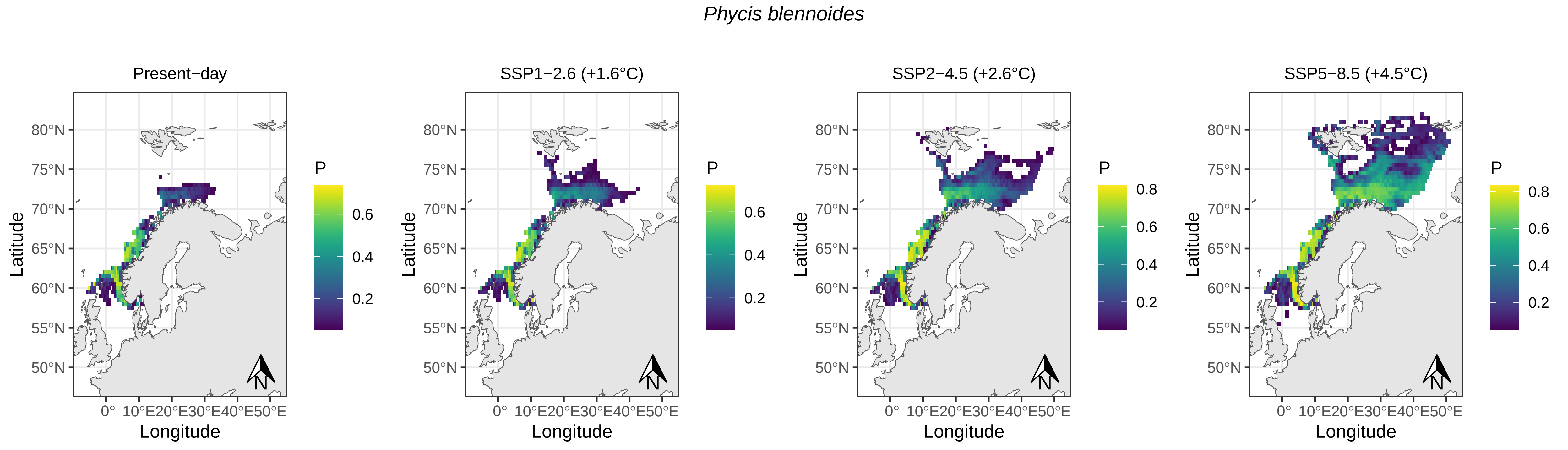
Phycis blennoides
Present−day
SSP1−2.6 (+1.6°C)
SSP2−4.5 (+2.6°C)
SSP5−8.5 (+4.5°C)
Latitude
Longitude
80°N
75°N
70°N
65°N
60°N
55°N
50°N
0°
10°E20°E30°E40°E50°E
P
0.2
0.4
0.6
0.8
N

*Platichthys flesus*

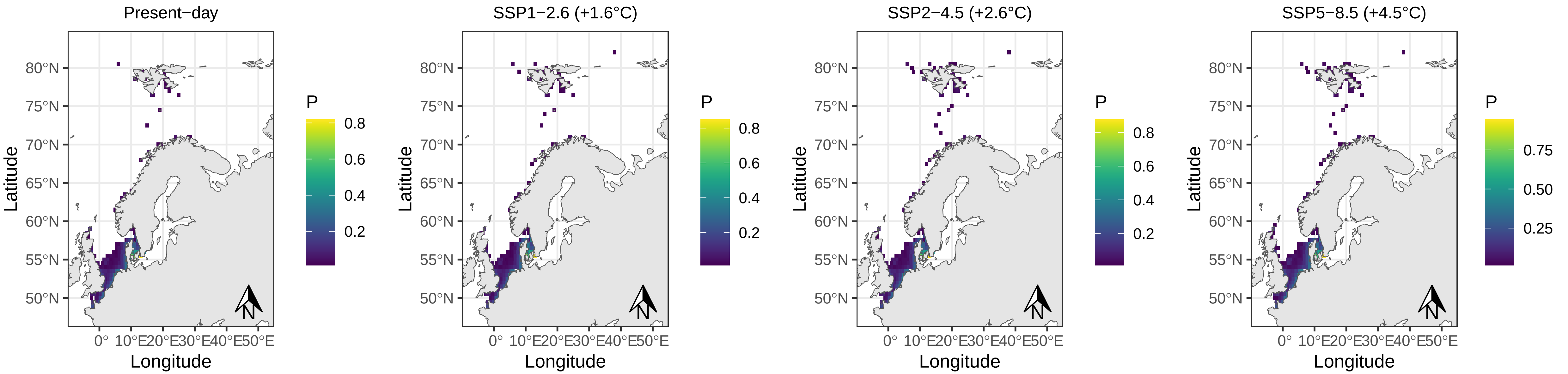

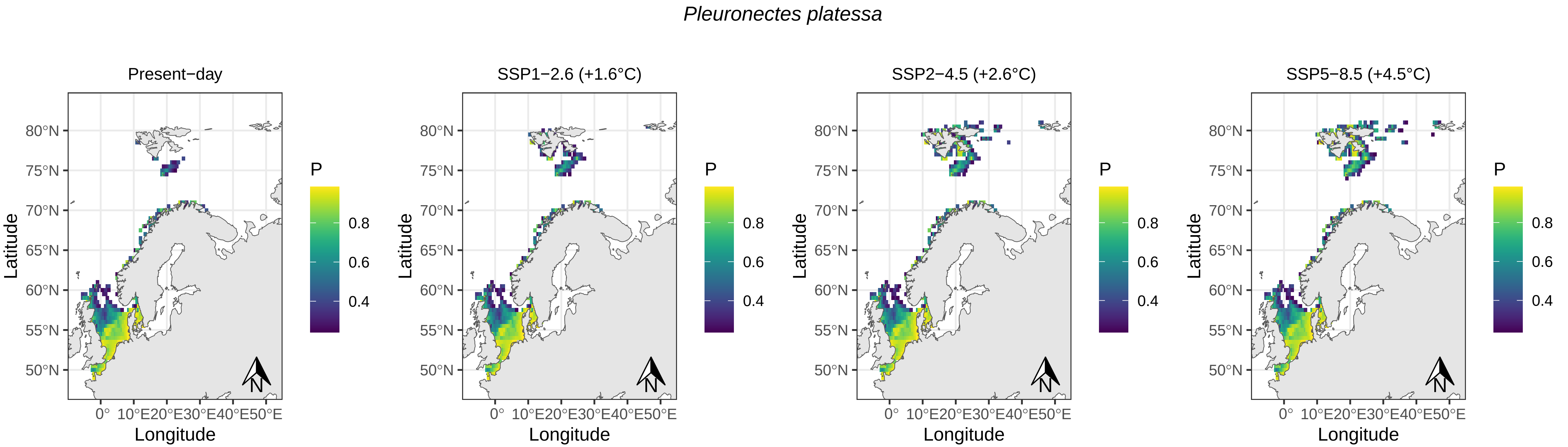
Pleuronectes platessa
Present−day
SSP1−2.6 (+1.6°C)
SSP2−4.5 (+2.6°C)
SSP5−8.5 (+4.5°C)
80°N
75°N
70°N
65°N
60°N
55°N
50°N
Latitude
0° 10°E 20°E 30°E 40°E 50°E
Longitude
P
0.8
0.6
0.4
N

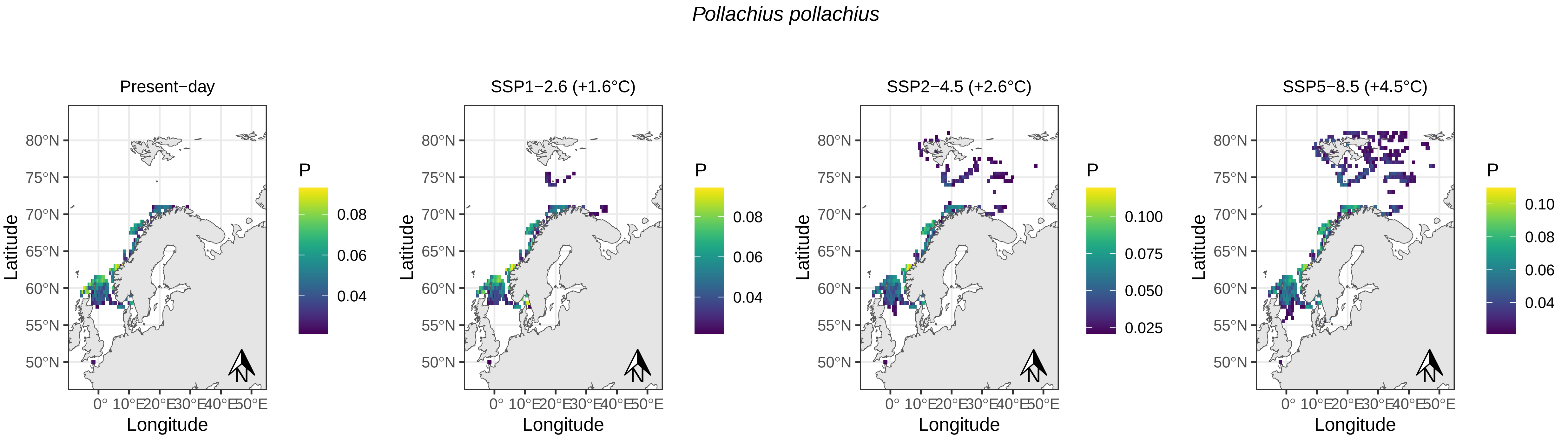

Pollachius pollachius
Present−day
SSP1−2.6 (+1.6°C)
SSP2−4.5 (+2.6°C)
SSP5−8.5 (+4.5°C)
80°N
75°N
70°N
65°N
60°N
55°N
50°N
Latitude
0° 10°E 20°E 30°E 40°E 50°E
Longitude
N
P
0.08
0.06
0.04
0.100
0.075
0.050
0.025
0.10

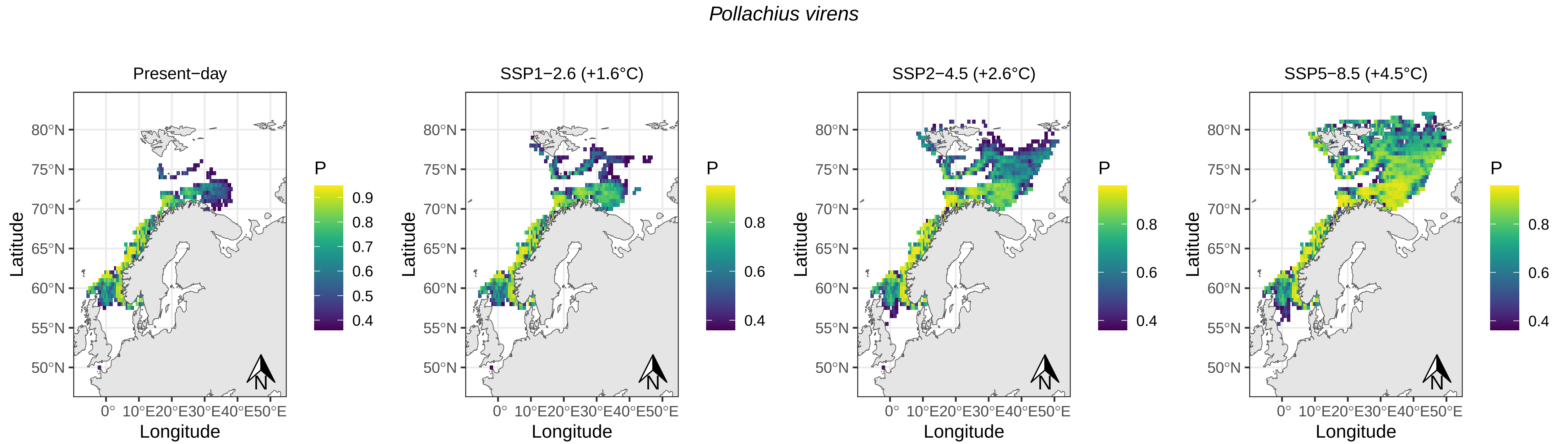

Pollachius virens
Present−day
SSP1−2.6 (+1.6°C)
SSP2−4.5 (+2.6°C)
SSP5−8.5 (+4.5°C)
Latitude
Longitude
80°N
75°N
70°N
65°N
60°N
55°N
50°N
0°
10°E
20°E
30°E
40°E
50°E
P
0.9
0.8
0.7
0.6
0.5
0.4
N

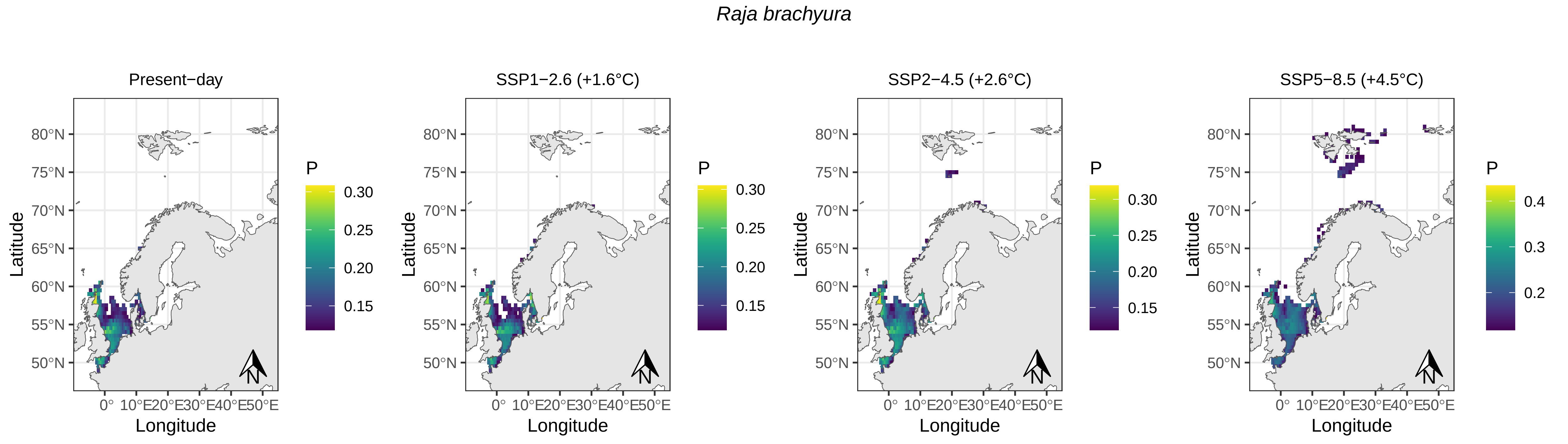
Raja brachyura
Present−day
SSP1−2.6 (+1.6°C)
SSP2−4.5 (+2.6°C)
SSP5−8.5 (+4.5°C)
Latitude
Longitude
80°N
75°N
70°N
65°N
60°N
55°N
50°N
0°
10°E
20°E
30°E
40°E
50°E
P
0.30
0.25
0.20
0.15
0.4
0.3
0.2
N

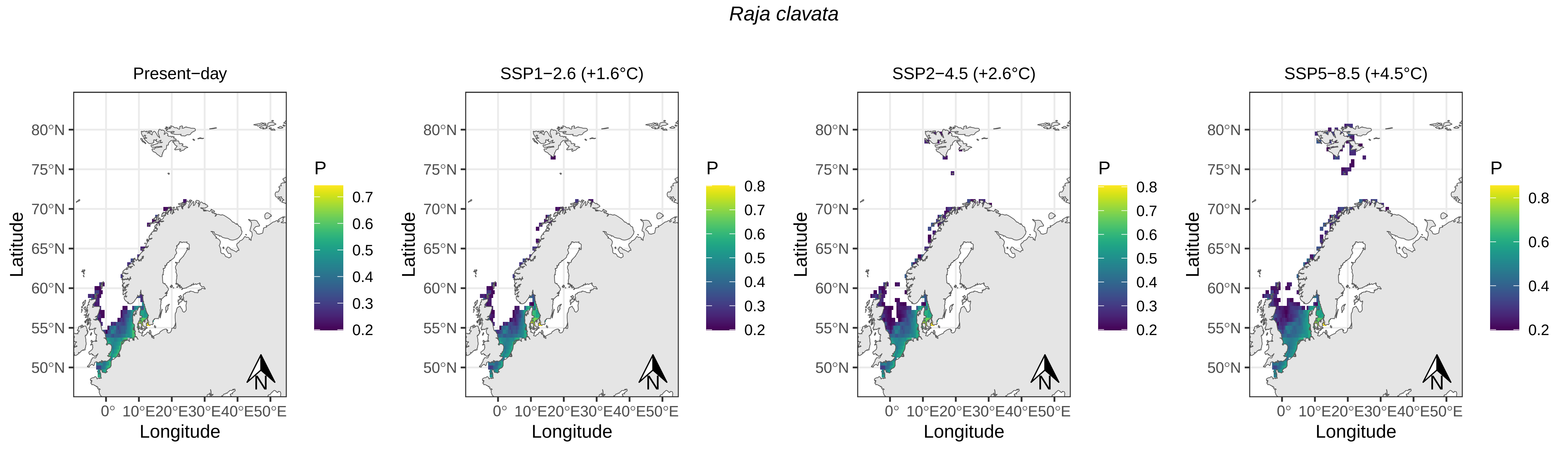
Raja clavata
Present−day
SSP1−2.6 (+1.6°C)
SSP2−4.5 (+2.6°C)
SSP5−8.5 (+4.5°C)
Latitude
Longitude
80°N
75°N
70°N
65°N
60°N
55°N
50°N
0°
10°E
20°E
30°E
40°E
50°E
P
0.2
0.3
0.4
0.5
0.6
0.7
0.8
N

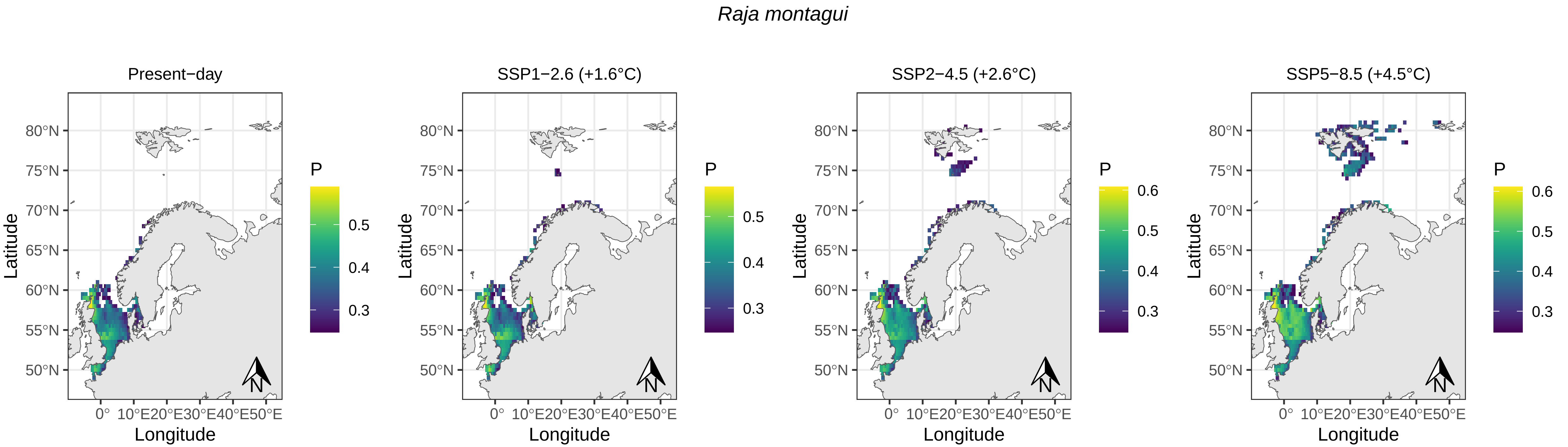
Raja montagui
Present−day
SSP1−2.6 (+1.6°C)
SSP2−4.5 (+2.6°C)
SSP5−8.5 (+4.5°C)
80°N
75°N
70°N
65°N
60°N
55°N
50°N
Latitude
0°
10°E
20°E
30°E
40°E
50°E
Longitude
P
0.3
0.4
0.5
0.6
N

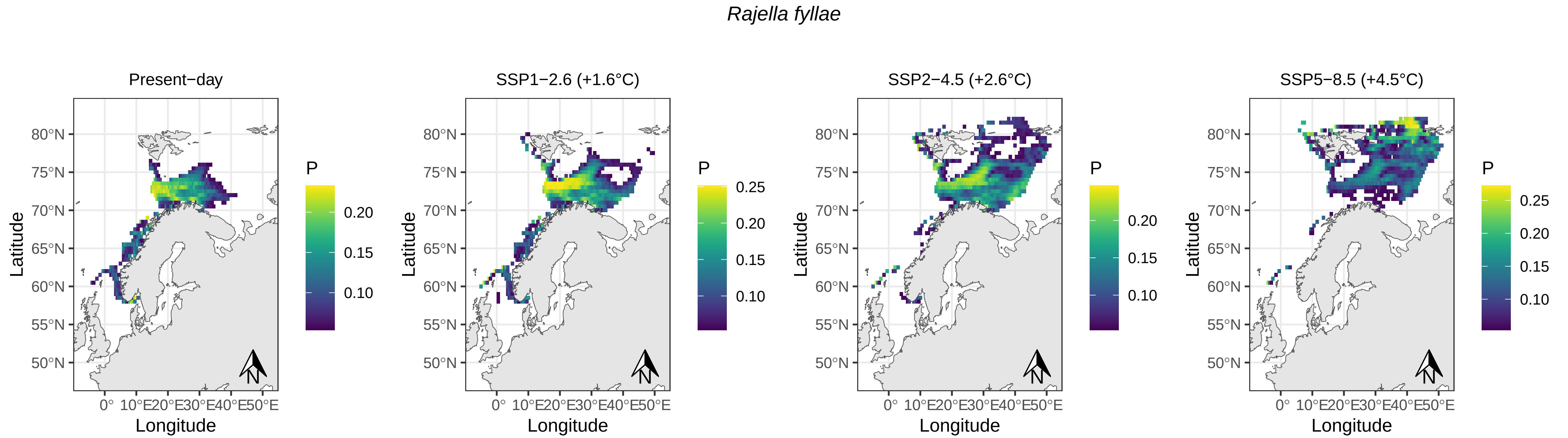
Rajella fyllae
Present−day
SSP1−2.6 (+1.6°C)
SSP2−4.5 (+2.6°C)
SSP5−8.5 (+4.5°C)
80°N
75°N
70°N
65°N
60°N
55°N
50°N
Latitude
0°
10°E
20°E
30°E
40°E
50°E
Longitude
P
0.25
0.20
0.15
0.10
N

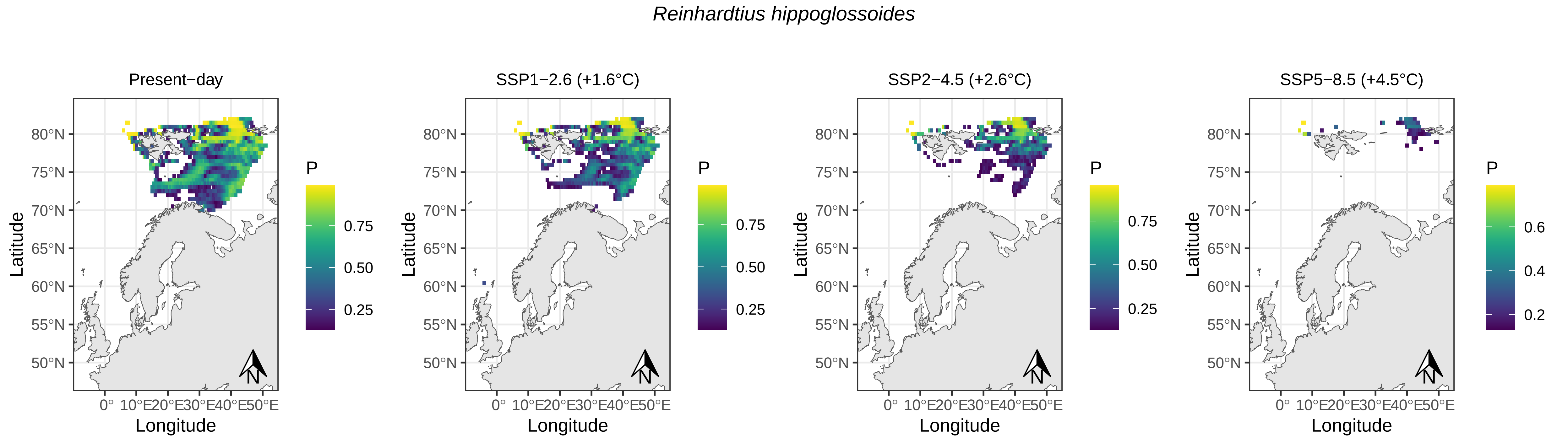

Reinhardtius hippoglossoides
Present−day
SSP1−2.6 (+1.6°C)
SSP2−4.5 (+2.6°C)
SSP5−8.5 (+4.5°C)
80°N
75°N
70°N
65°N
60°N
55°N
50°N
Latitude
0°
10°E
20°E
30°E
40°E
50°E
Longitude
P
0.75
0.50
0.25
0.6
0.4
0.2
N

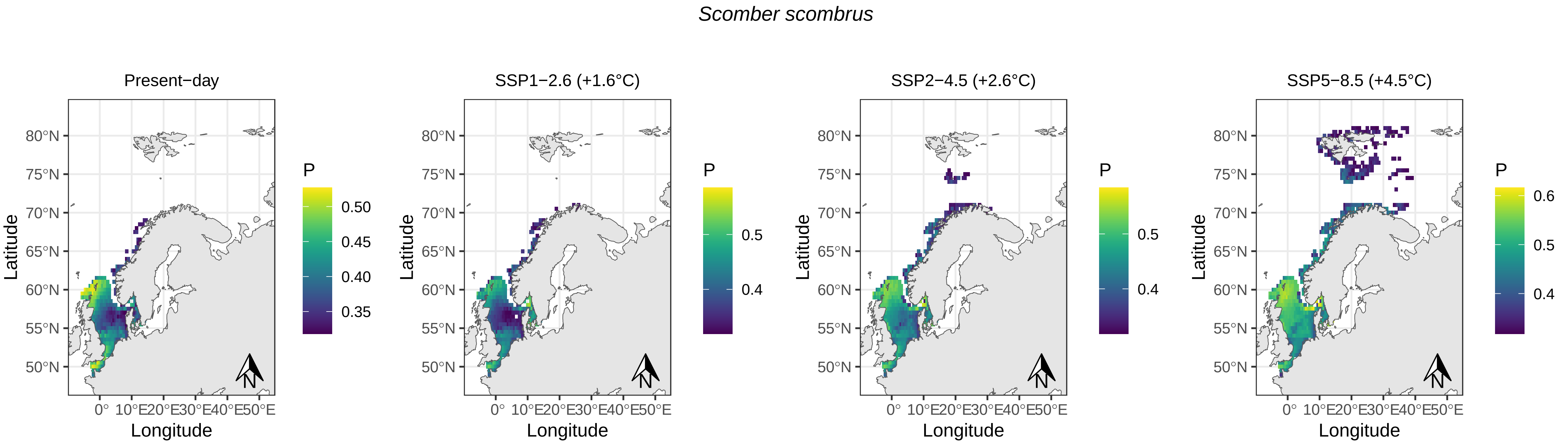
Scomber scombrus
Present−day
SSP1−2.6 (+1.6°C)
SSP2−4.5 (+2.6°C)
SSP5−8.5 (+4.5°C)
80°N
75°N
70°N
65°N
60°N
55°N
50°N
Latitude
0°
10°E
20°E
30°E
40°E
50°E
Longitude
P
0.50
0.45
0.40
0.35
0.5
0.4
0.6
N

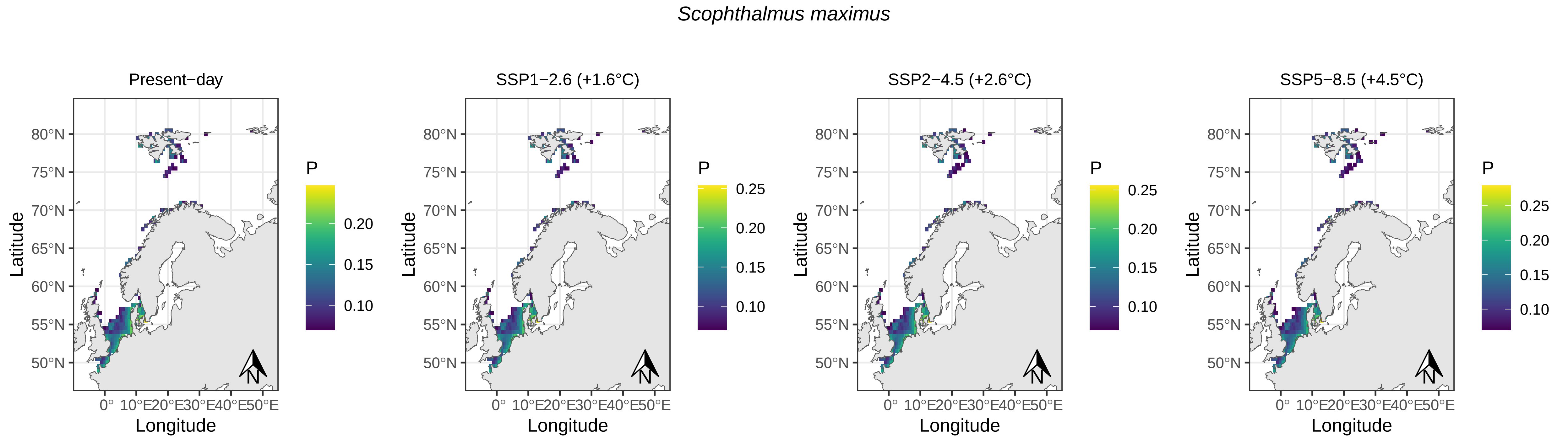

Scophthalmus maximus
Present−day
SSP1−2.6 (+1.6°C)
SSP2−4.5 (+2.6°C)
SSP5−8.5 (+4.5°C)
Latitude
Longitude
P
80°N
75°N
70°N
65°N
60°N
55°N
50°N
0°
10°E
20°E
30°E
40°E
50°E
0.10
0.15
0.20
0.25
N

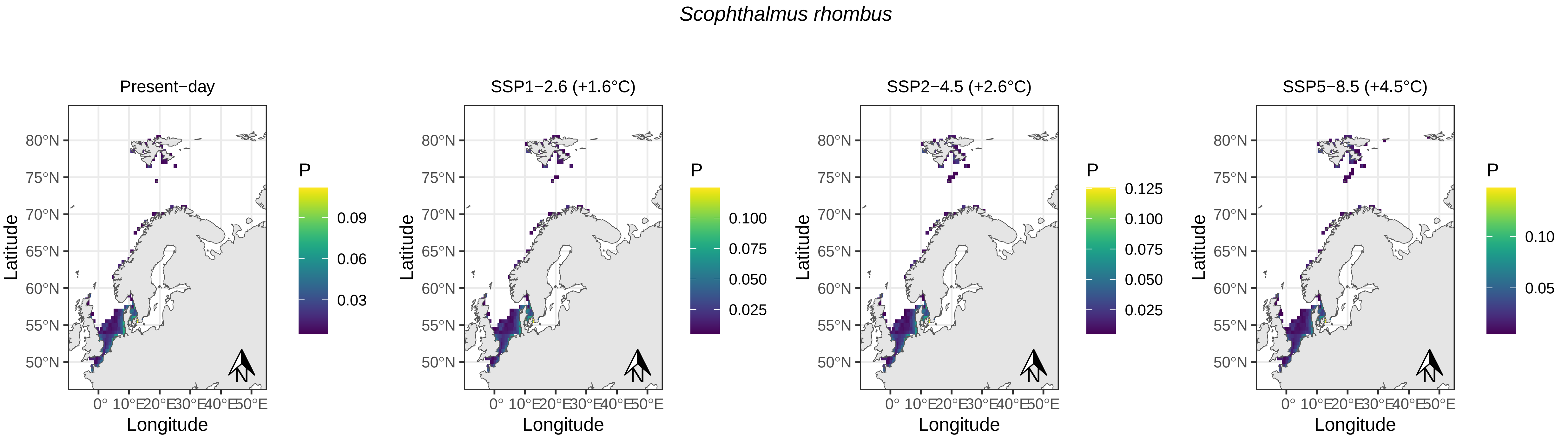
Scophthalmus rhombus
Present−day
SSP1−2.6 (+1.6°C)
SSP2−4.5 (+2.6°C)
SSP5−8.5 (+4.5°C)
80°N
75°N
70°N
65°N
60°N
55°N
50°N
Latitude
0°
10°E
20°E
30°E
40°E
50°E
Longitude
P
0.09
0.06
0.03
0.100
0.075
0.050
0.025
0.125
0.10
0.05
N

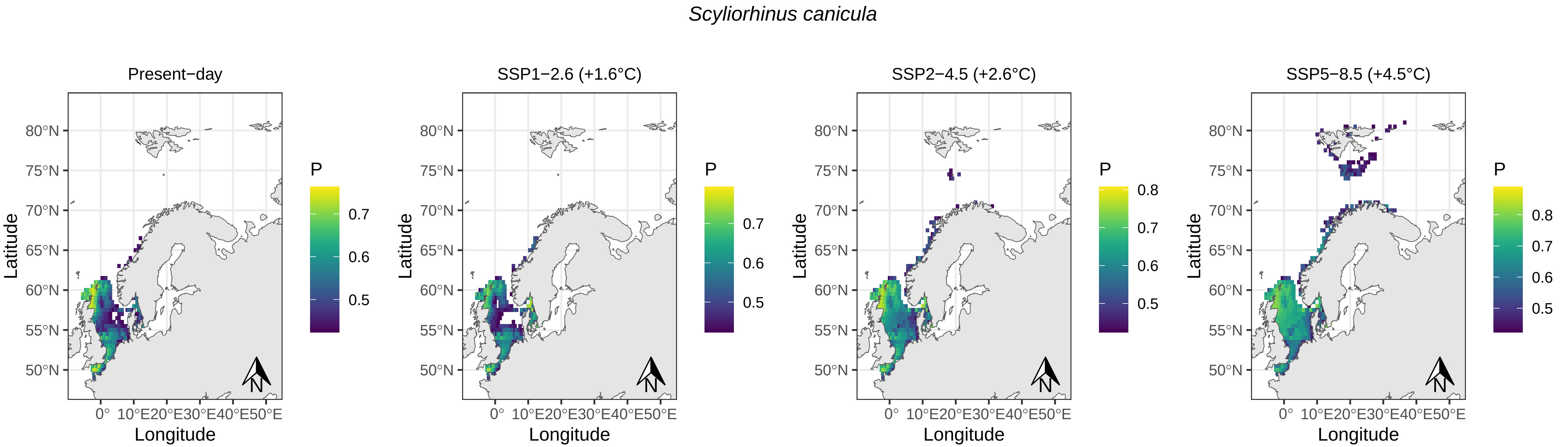
*Scyliorhinus canicula*
Present−day
SSP1−2.6 (+1.6°C)
SSP2−4.5 (+2.6°C)
SSP5−8.5 (+4.5°C)
Latitude
Longitude
80°N
75°N
70°N
65°N
60°N
55°N
50°N
0°
10°E
20°E
30°E
40°E
50°E
P
0.5
0.6
0.7
0.8
N

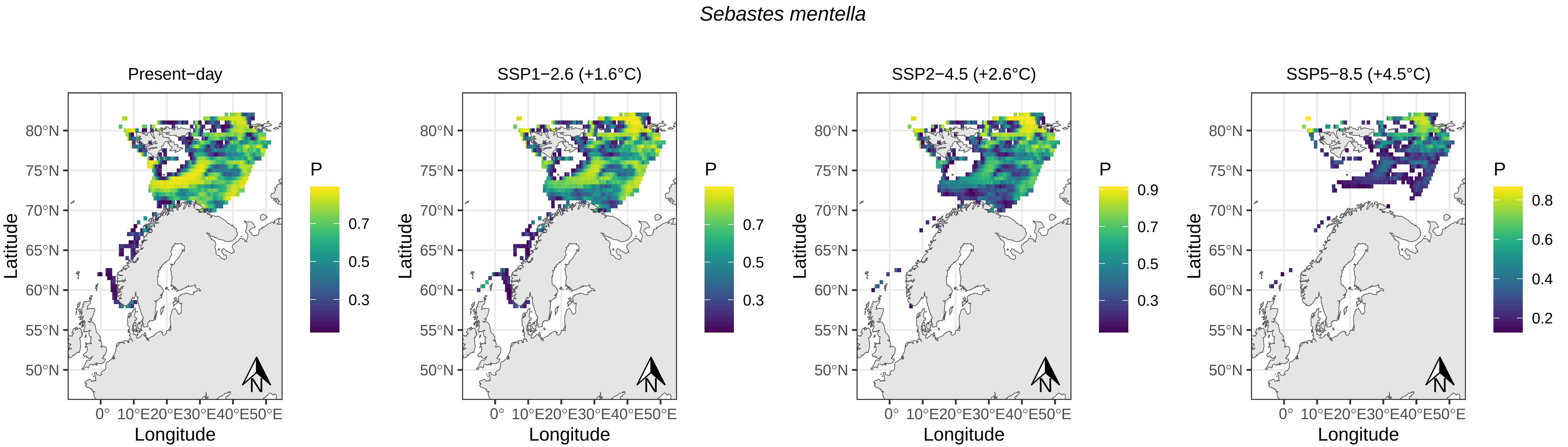
*Sebastes mentella*
Present−day
SSP1−2.6 (+1.6°C)
SSP2−4.5 (+2.6°C)
SSP5−8.5 (+4.5°C)
Latitude
Longitude
80°N
75°N
70°N
65°N
60°N
55°N
50°N
0°
10°E
20°E
30°E
40°E
50°E
N
P
0.9
0.8
0.7
0.6
0.5
0.4
0.3
0.2

*Sebastes norvegicus*

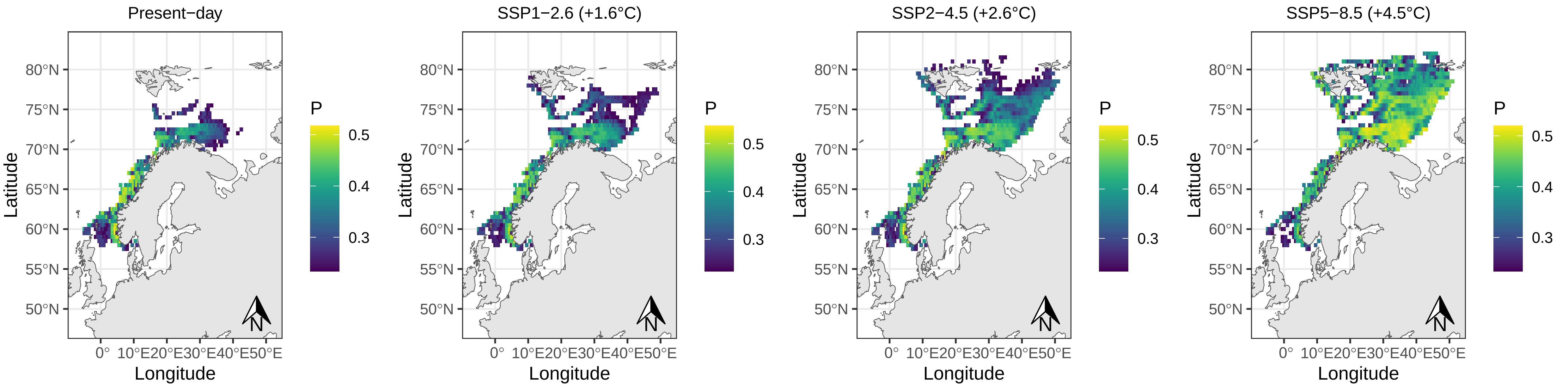

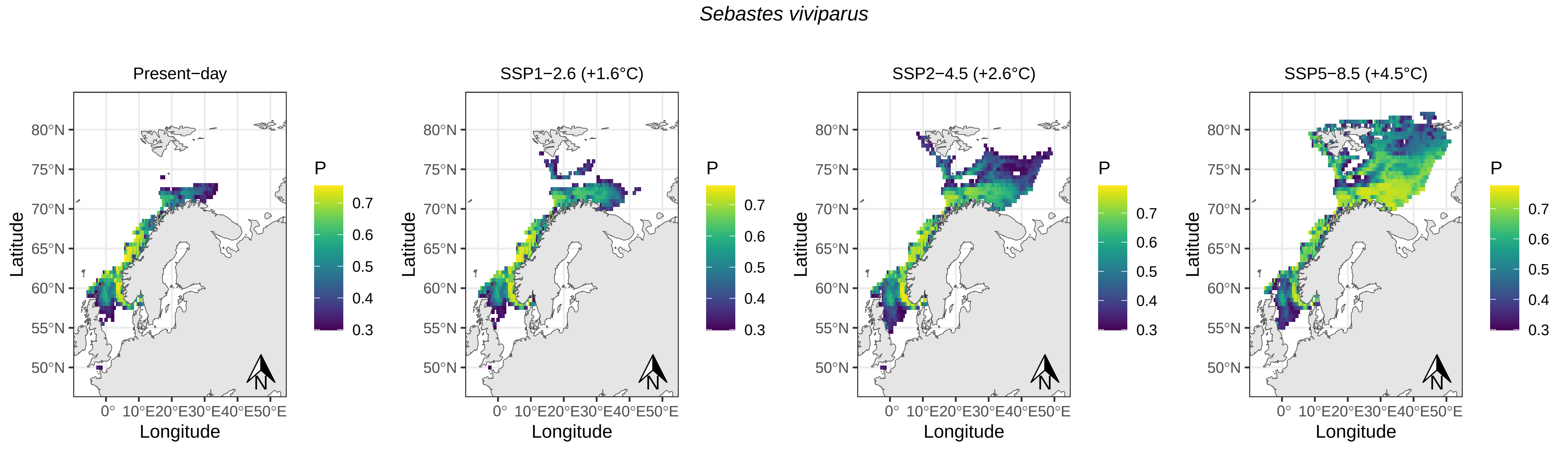
Sebastes viviparus
Present−day
SSP1−2.6 (+1.6°C)
SSP2−4.5 (+2.6°C)
SSP5−8.5 (+4.5°C)
80°N
75°N
70°N
65°N
60°N
55°N
50°N
Latitude
0°
10°E
20°E
30°E
40°E
50°E
Longitude
P
0.7
0.6
0.5
0.4
0.3
N

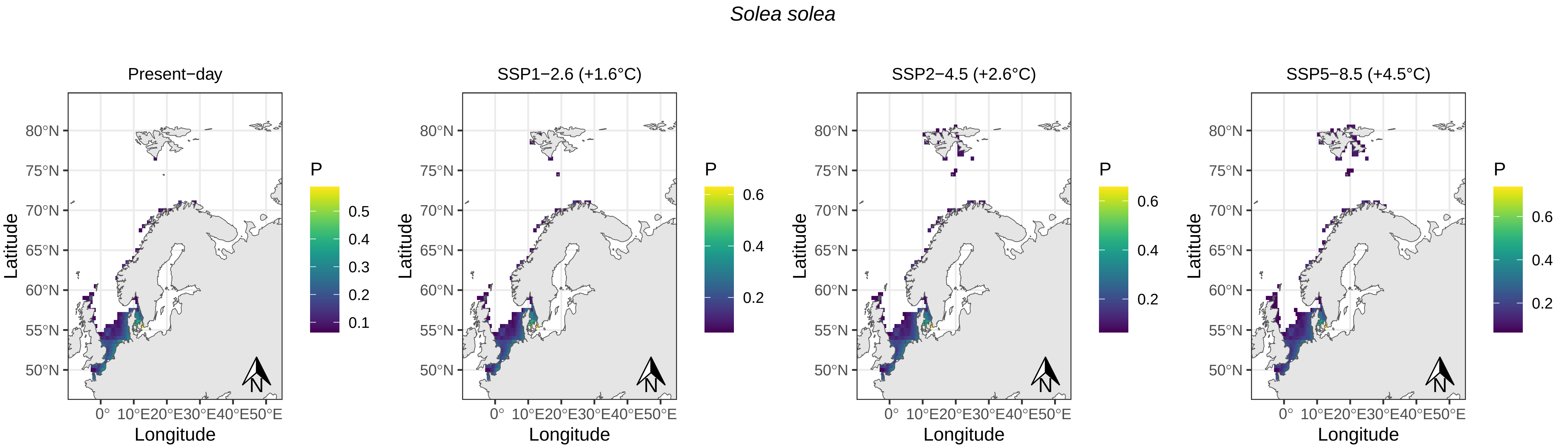
Solea solea
Present−day
SSP1−2.6 (+1.6°C)
SSP2−4.5 (+2.6°C)
SSP5−8.5 (+4.5°C)
80°N
75°N
70°N
65°N
60°N
55°N
50°N
Latitude
0°
10°E
20°E
30°E
40°E
50°E
Longitude
P
0.5
0.4
0.3
0.2
0.1
0.6
N

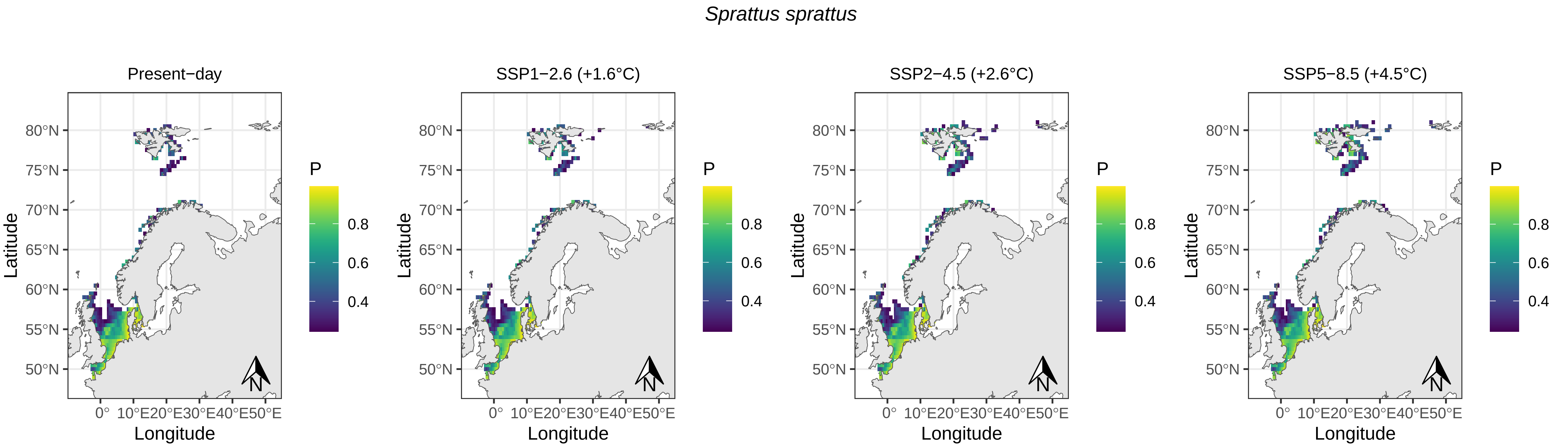
Sprattus sprattus
Present−day
SSP1−2.6 (+1.6°C)
SSP2−4.5 (+2.6°C)
SSP5−8.5 (+4.5°C)
80°N
75°N
70°N
65°N
60°N
55°N
50°N
Latitude
0°
10°E
20°E
30°E
40°E
50°E
Longitude
P
0.8
0.6
0.4
N

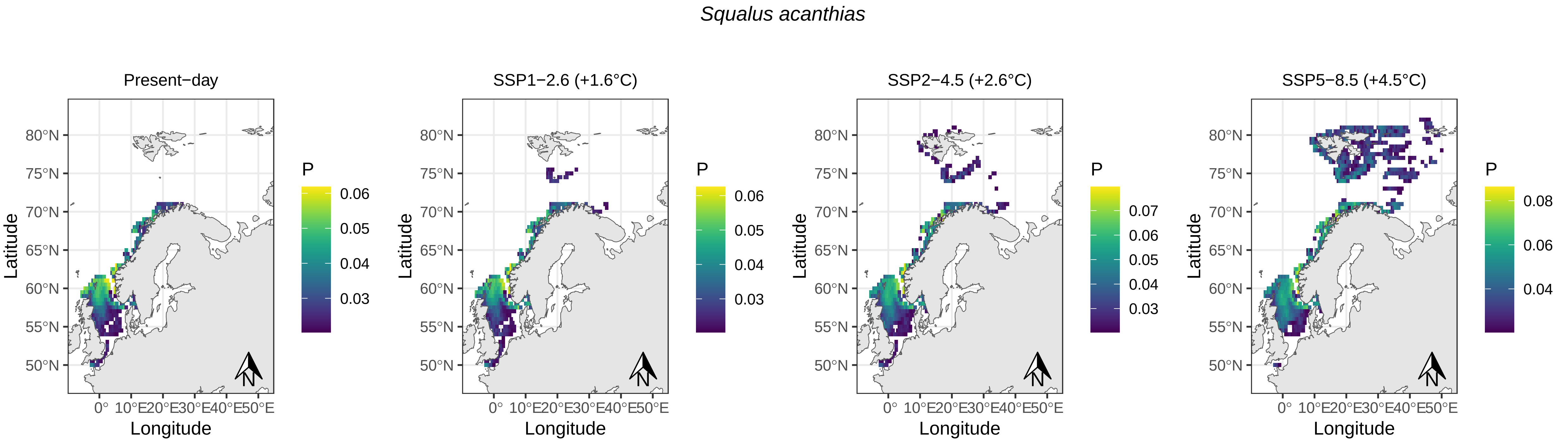

Squalus acanthias
Present−day
SSP1−2.6 (+1.6°C)
SSP2−4.5 (+2.6°C)
SSP5−8.5 (+4.5°C)
Latitude
Longitude
80°N
75°N
70°N
65°N
60°N
55°N
50°N
0°
10°E
20°E
30°E
40°E
50°E
P
0.06
0.05
0.04
0.03
0.07
0.08
N

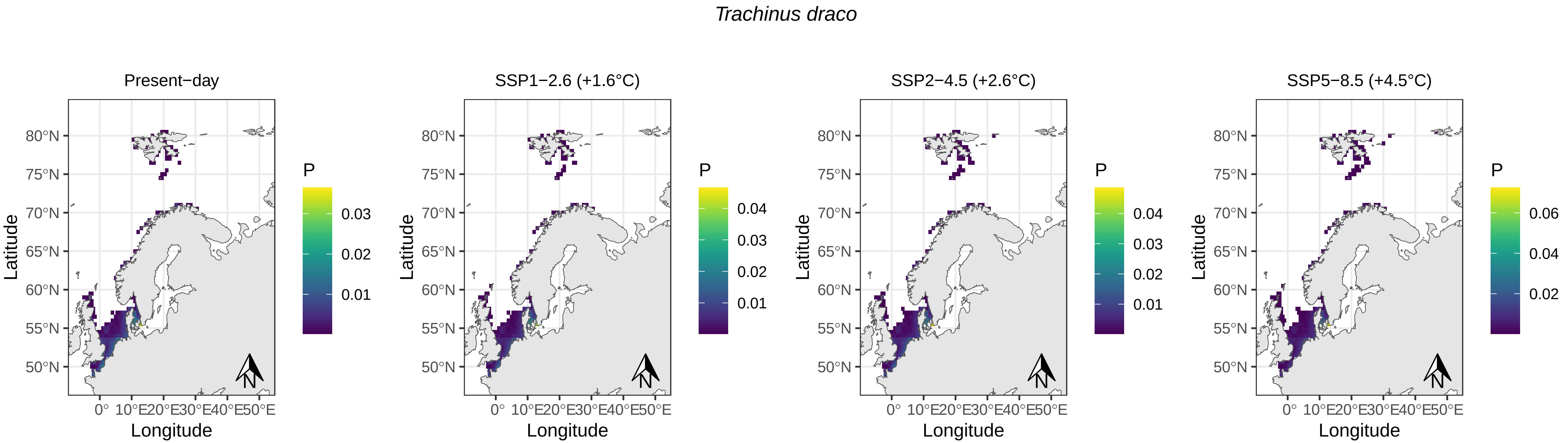

Trachinus draco
Present−day
SSP1−2.6 (+1.6°C)
SSP2−4.5 (+2.6°C)
SSP5−8.5 (+4.5°C)
Latitude
Longitude
80°N
75°N
70°N
65°N
60°N
55°N
50°N
0° 10°E 20°E 30°E 40°E 50°E
P
0.03
0.02
0.01
0.04
0.03
0.02
0.01
0.04
0.03
0.02
0.01
0.06
0.04
0.02
N

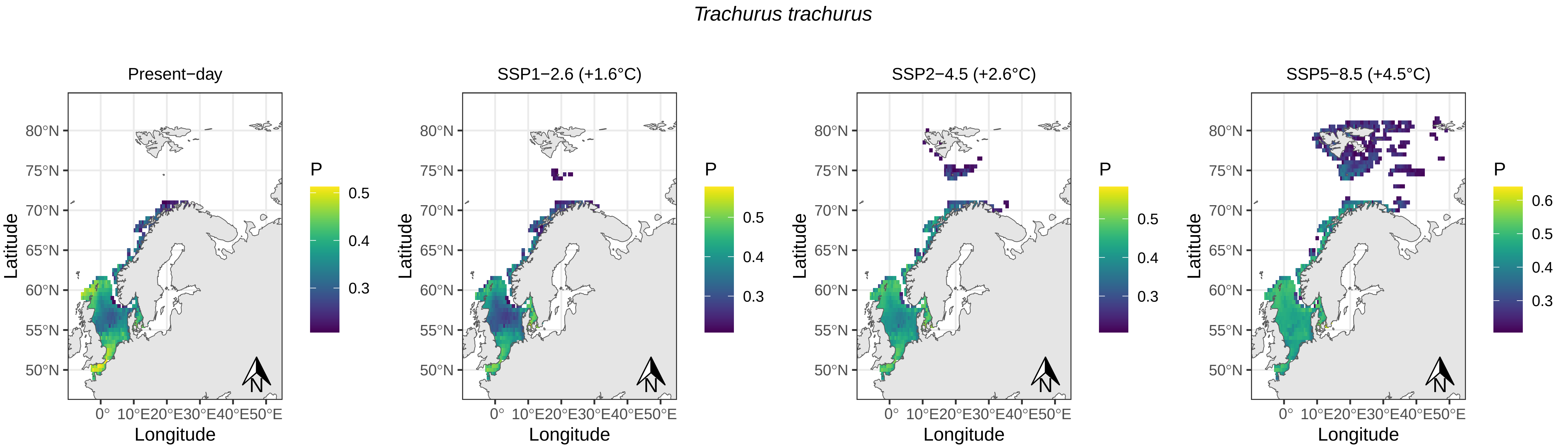
Trachurus trachurus
Present−day
SSP1−2.6 (+1.6°C)
SSP2−4.5 (+2.6°C)
SSP5−8.5 (+4.5°C)
Latitude
Longitude
80°N
75°N
70°N
65°N
60°N
55°N
50°N
0° 10°E 20°E 30°E 40°E 50°E
P
0.3 0.4 0.5
0.3 0.4 0.5
0.3 0.4 0.5
0.3 0.4 0.5 0.6
N

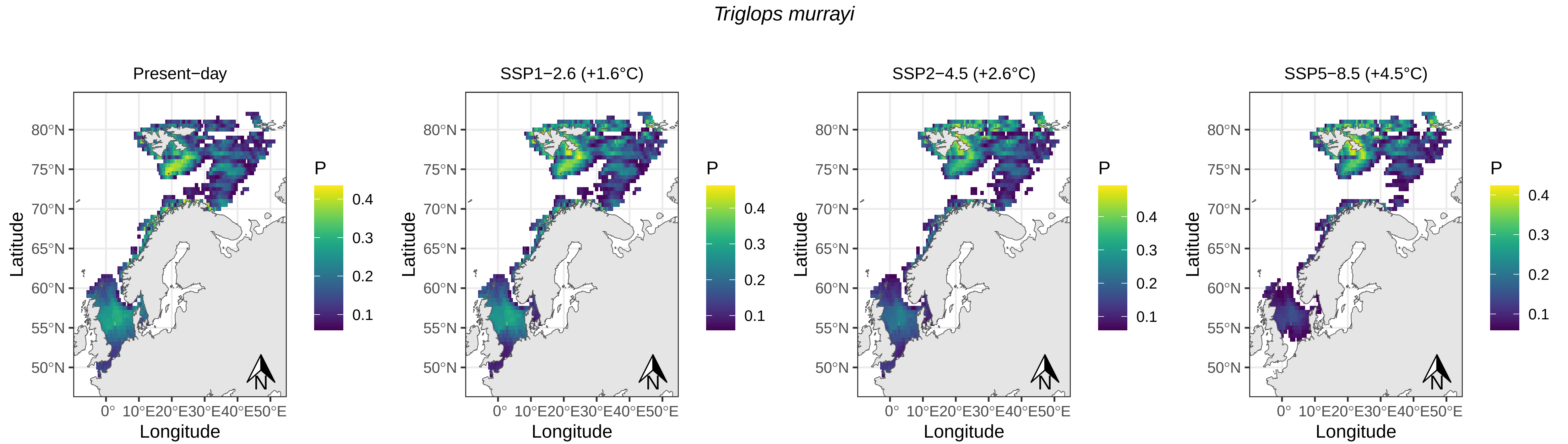
Triglops murrayi
Present−day
SSP1−2.6 (+1.6°C)
SSP2−4.5 (+2.6°C)
SSP5−8.5 (+4.5°C)
80°N
75°N
70°N
65°N
60°N
55°N
50°N
Latitude
0°
10°E
20°E
30°E
40°E
50°E
Longitude
P
0.4
0.3
0.2
0.1
N

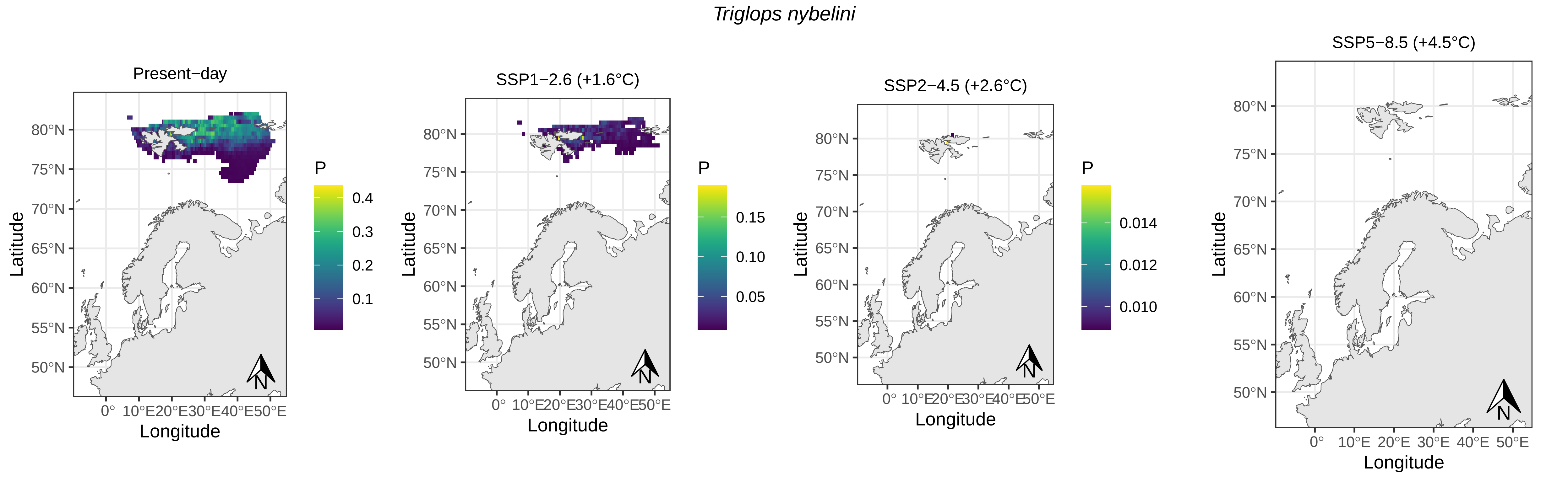
Triglops nybelini
Present−day
SSP1−2.6 (+1.6°C)
SSP2−4.5 (+2.6°C)
SSP5−8.5 (+4.5°C)
P
0.4
0.3
0.2
0.1
0.15
0.10
0.05
0.014
0.012
0.010
Latitude
Longitude
80°N
75°N
70°N
65°N
60°N
55°N
50°N
0°
10°E
20°E
30°E
40°E
50°E
N

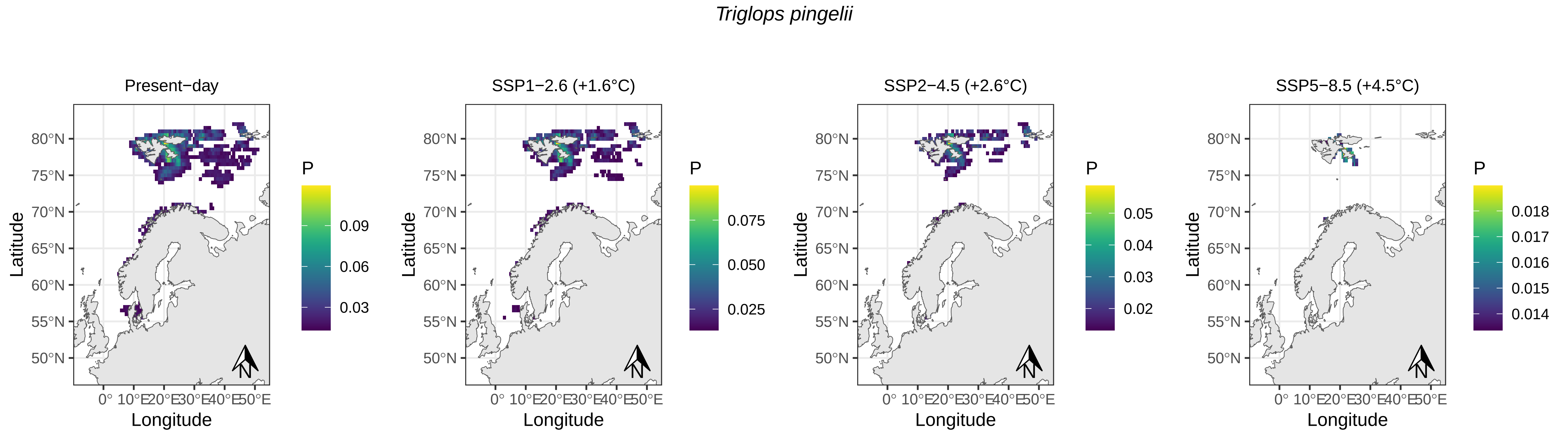
Triglops pingelii
Present−day
SSP1−2.6 (+1.6°C)
SSP2−4.5 (+2.6°C)
SSP5−8.5 (+4.5°C)
Latitude
Longitude
80°N
75°N
70°N
65°N
60°N
55°N
50°N
0° 10°E 20°E 30°E 40°E 50°E
P
0.09
0.06
0.03
0.075
0.050
0.025
0.05
0.04
0.03
0.02
0.018
0.017
0.016
0.015
0.014
N

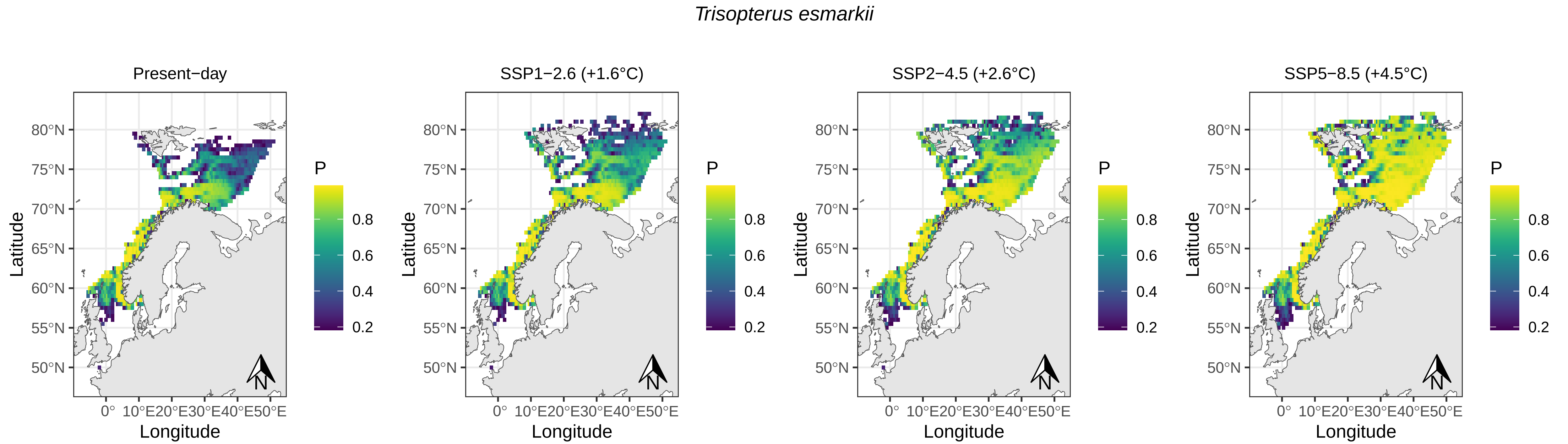
Trisopterus esmarkii
Present−day
SSP1−2.6 (+1.6°C)
SSP2−4.5 (+2.6°C)
SSP5−8.5 (+4.5°C)
80°N
75°N
70°N
65°N
60°N
55°N
50°N
Latitude
0°
10°E
20°E
30°E
40°E
50°E
Longitude
P
0.8
0.6
0.4
0.2
N

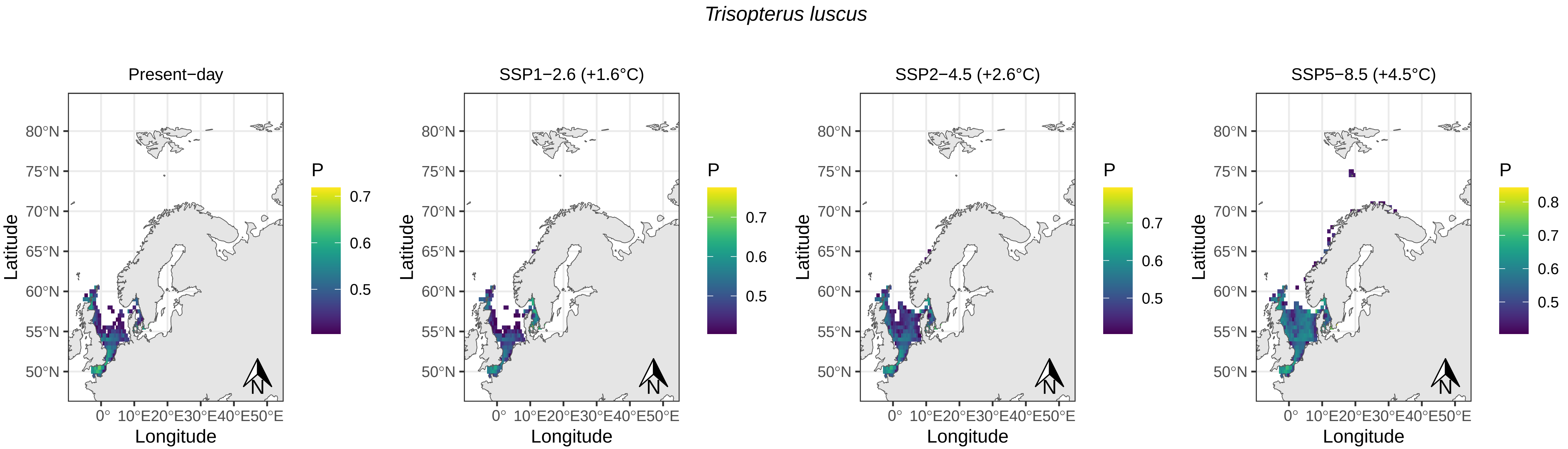

Trisopterus luscus
Present−day
SSP1−2.6 (+1.6°C)
SSP2−4.5 (+2.6°C)
SSP5−8.5 (+4.5°C)
80°N
75°N
70°N
65°N
60°N
55°N
50°N
Latitude
0°
10°E
20°E
30°E
40°E
50°E
Longitude
P
0.5
0.6
0.7
0.8
N

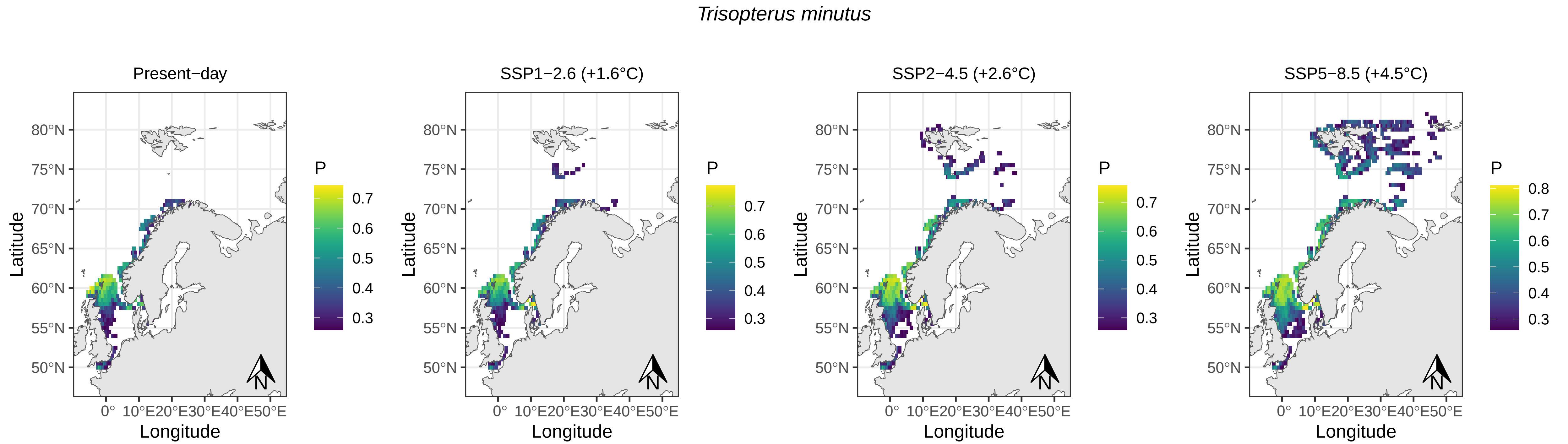

Trisopterus minutus
Present−day
SSP1−2.6 (+1.6°C)
SSP2−4.5 (+2.6°C)
SSP5−8.5 (+4.5°C)
Latitude
Longitude
80°N
75°N
70°N
65°N
60°N
55°N
50°N
0°
10°E
20°E
30°E
40°E
50°E
P
0.3
0.4
0.5
0.6
0.7
0.8
N

*Zeus faber*

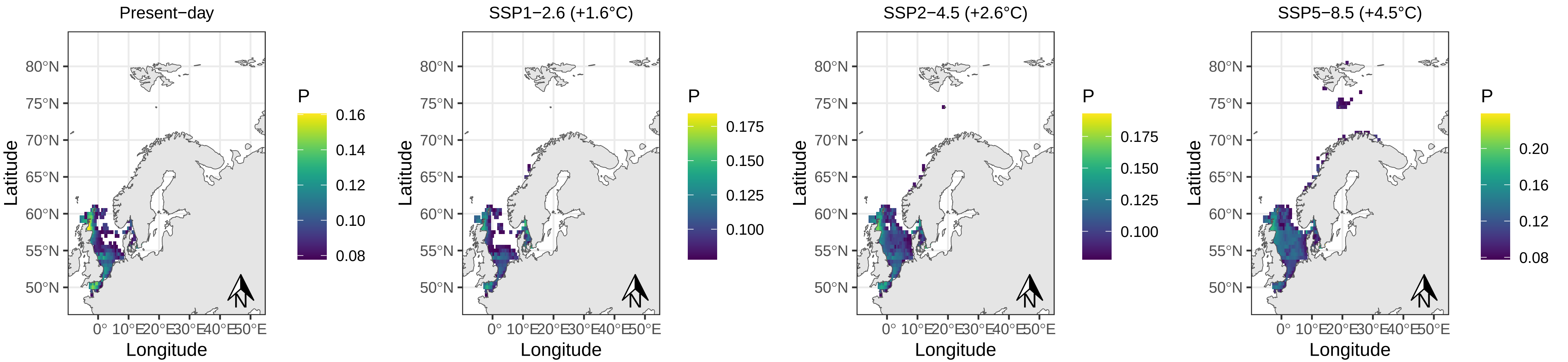
Present−day
SSP1−2.6 (+1.6°C)
SSP2−4.5 (+2.6°C)
SSP5−8.5 (+4.5°C)
80°N
75°N
70°N
65°N
60°N
55°N
50°N
Latitude
0° 10°E 20°E 30°E 40°E 50°E
Longitude
P
0.16
0.14
0.12
0.10
0.08
0.175
0.150
0.125
0.100
0.175
0.150
0.125
0.100
0.20
0.16
0.12
0.08
N
