# Supplementary material for: Future trends of marine fish biomass distributions from the North Sea to the Barents Sea
Source: Nat Commun. 2024 Jul 5;15:5637. doi: 10.1038/s41467-024-49911-9 (PMC11224334; doi:10.1038/s41467-024-49911-9)
Supplement: Supplementary file 6 — Supplementary Data 3 [file 41467_2024_49911_MOESM6_ESM.zip › Supplementary Data 3.pdf]

Amblyraja radiata

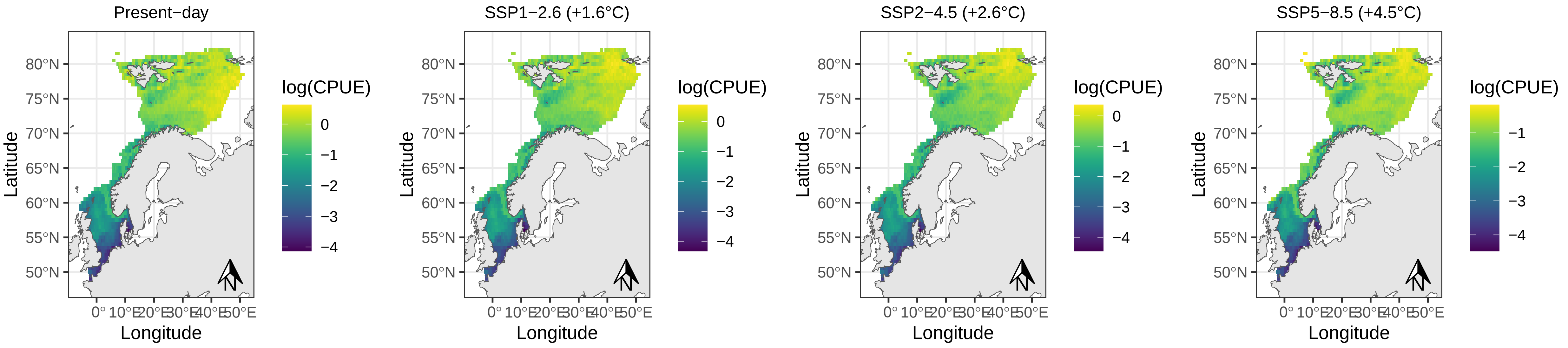

Anarhichas lupus

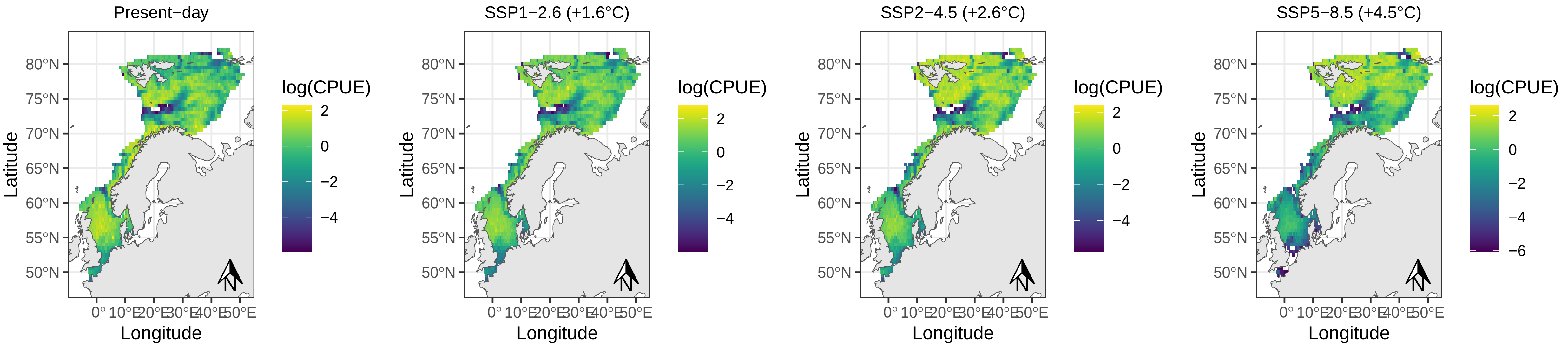

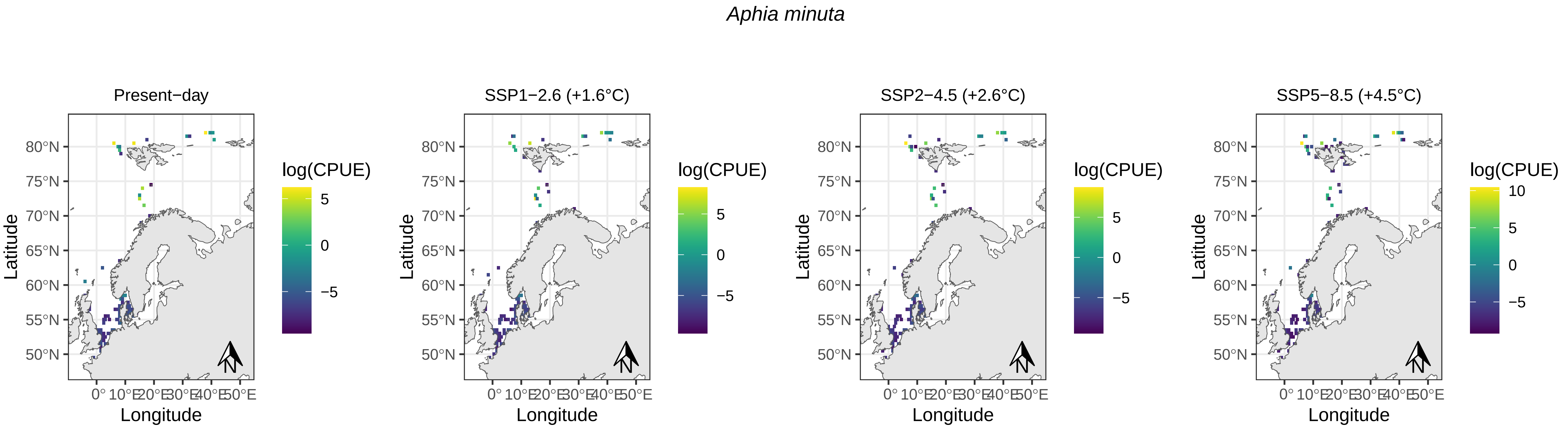
Aphia minuta
Present−day
SSP1−2.6 (+1.6°C)
SSP2−4.5 (+2.6°C)
SSP5−8.5 (+4.5°C)
log(CPUE)
Latitude
Longitude
80°N
75°N
70°N
65°N
60°N
55°N
50°N
0°
10°E
20°E
30°E
40°E
50°E
N
5
0
−5
10

Argentina silus

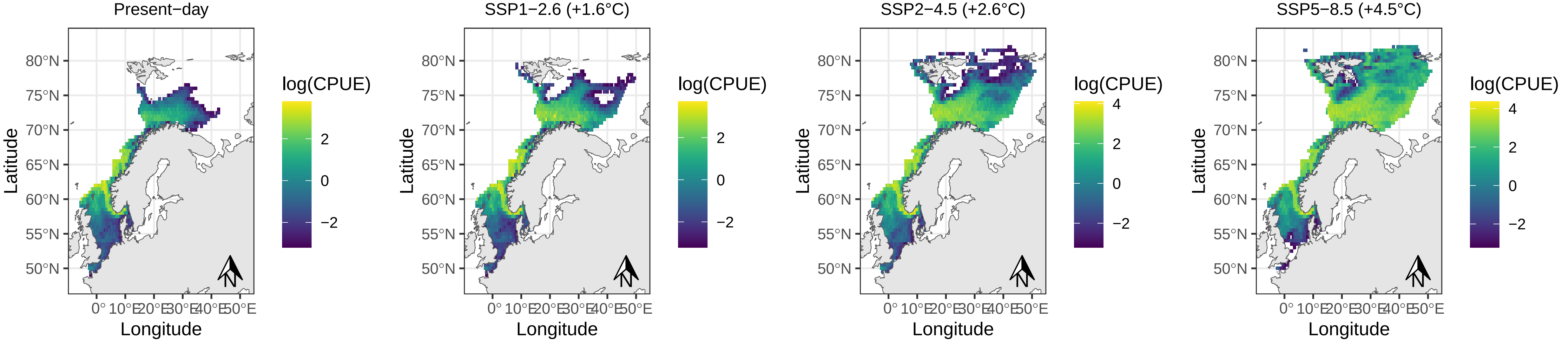

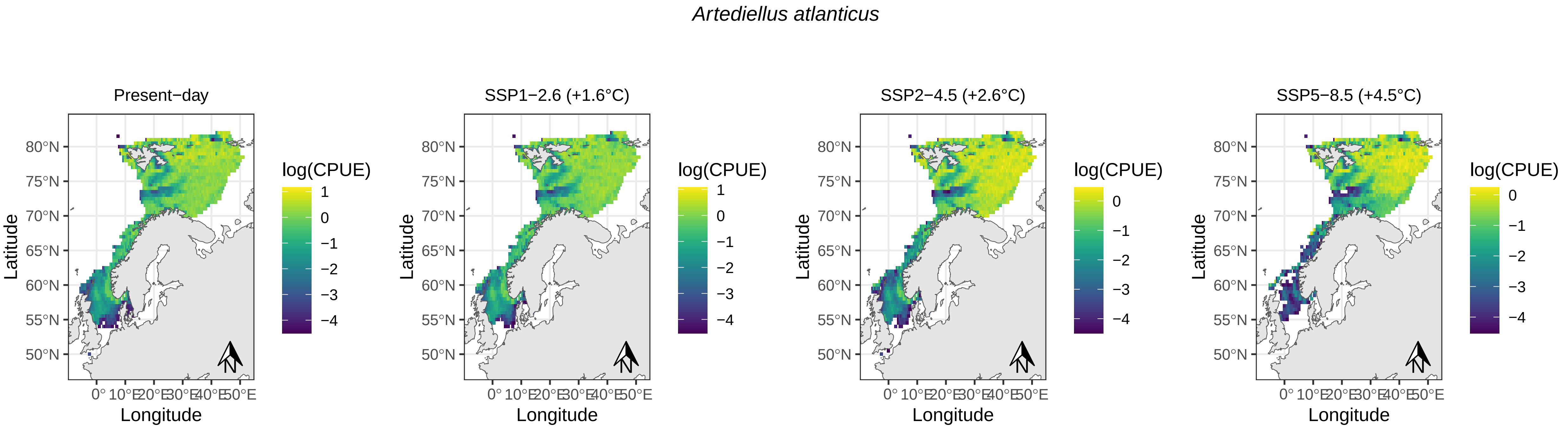

Artediellus atlanticus
Present−day
SSP1−2.6 (+1.6°C)
SSP2−4.5 (+2.6°C)
SSP5−8.5 (+4.5°C)
Latitude
Longitude
80°N
75°N
70°N
65°N
60°N
55°N
50°N
0°
10°E
20°E
30°E
40°E
50°E
log(CPUE)
1
0
−1
−2
−3
−4
N

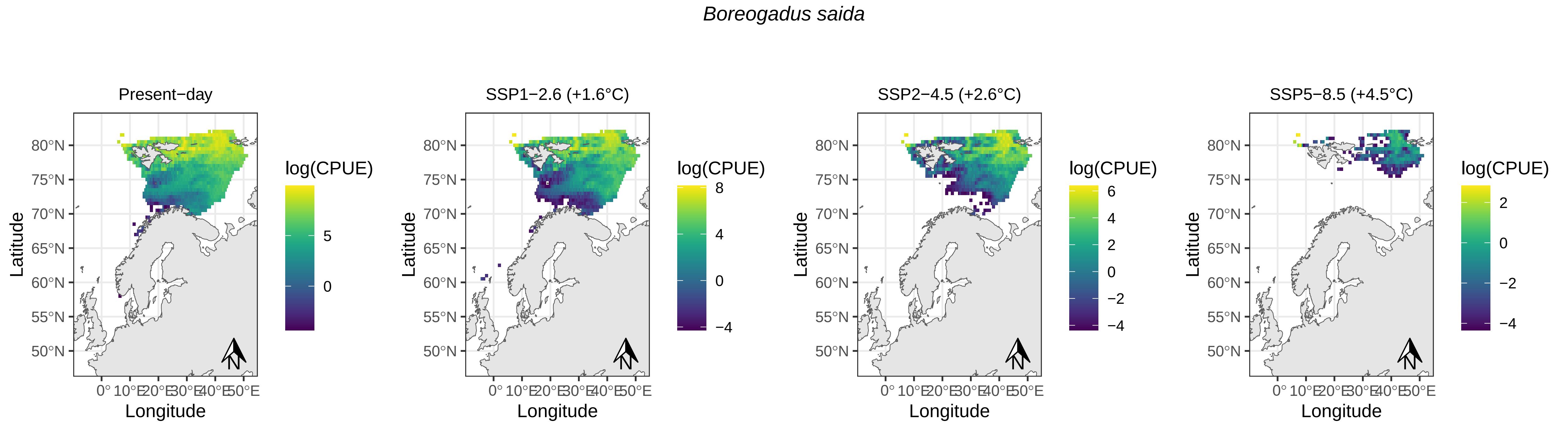
Boreogadus saida
Present−day
SSP1−2.6 (+1.6°C)
SSP2−4.5 (+2.6°C)
SSP5−8.5 (+4.5°C)
Latitude
80°N
75°N
70°N
65°N
60°N
55°N
50°N
Longitude
0°
10°E
20°E
30°E
40°E
50°E
log(CPUE)
5
0
8
4
0
−4
6
4
2
0
−2
−4
2
0
−2
−4
N

Brosme brosme

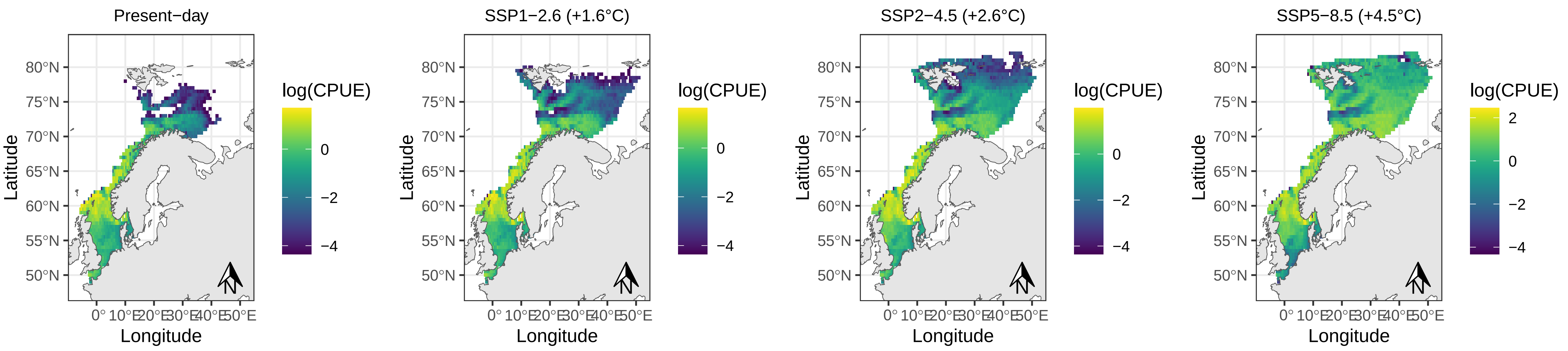

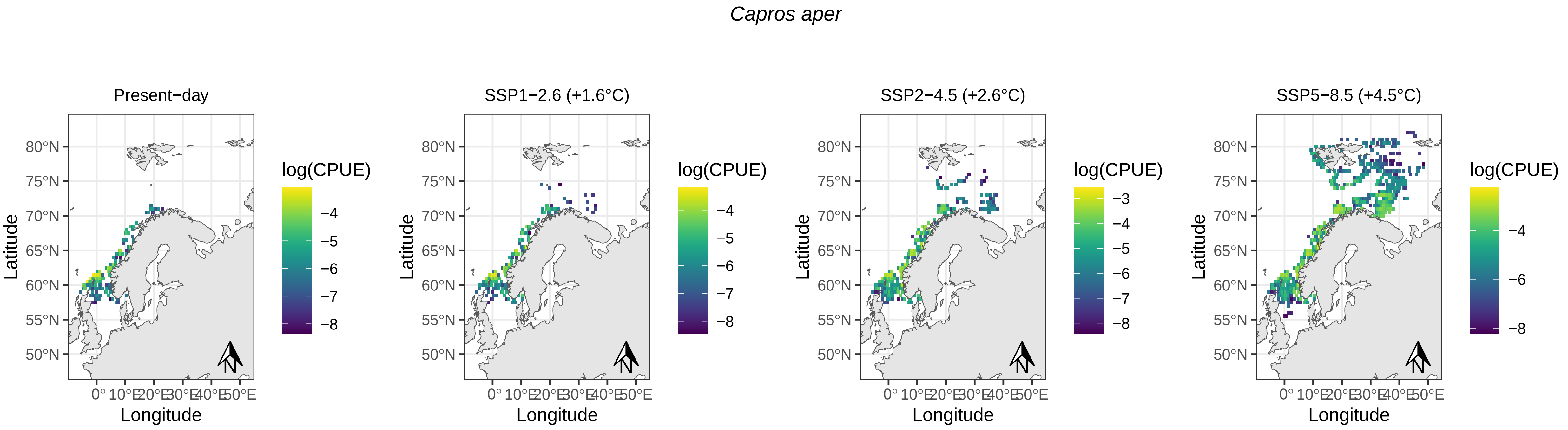
Capros aper
Present−day
SSP1−2.6 (+1.6°C)
SSP2−4.5 (+2.6°C)
SSP5−8.5 (+4.5°C)
80°N
75°N
70°N
65°N
60°N
55°N
50°N
Latitude
0° 10°E 20°E 30°E 40°E 50°E
Longitude
log(CPUE)
−3
−4
−5
−6
−7
−8
N

Chelidonichthys cuculus

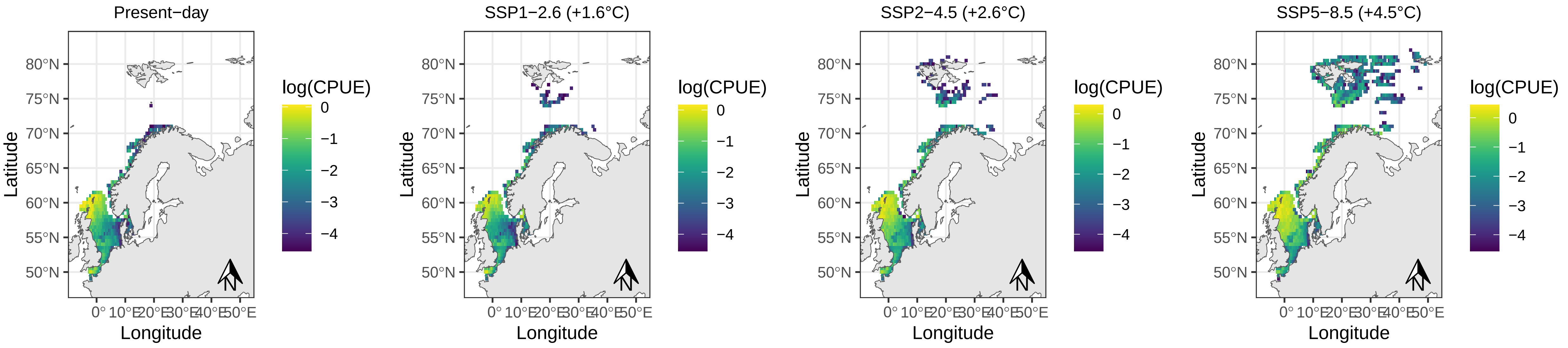

Chimaera monstrosa

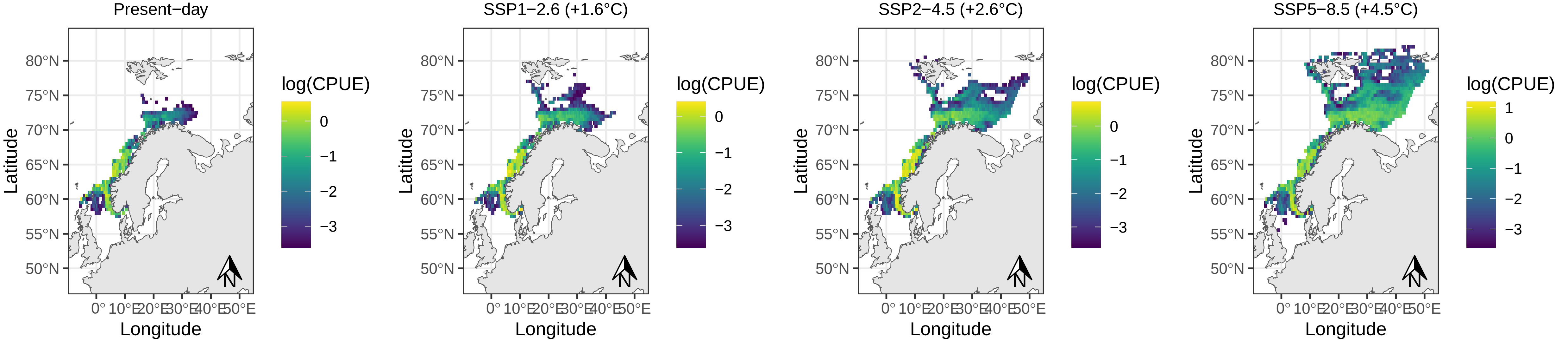

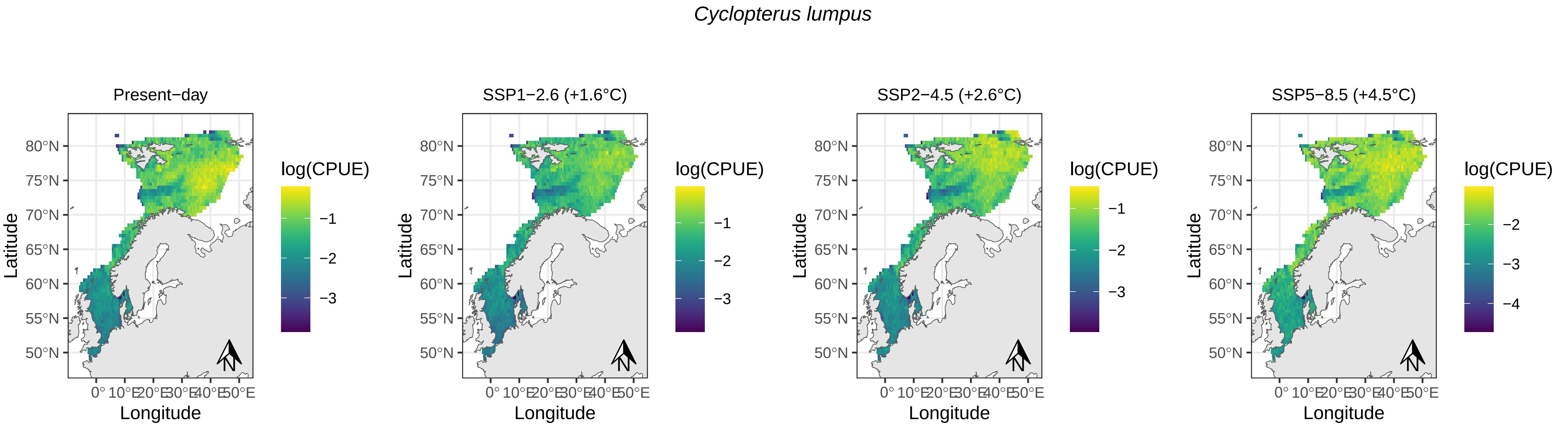

Cyclopterus lumpus
Present−day
SSP1−2.6 (+1.6°C)
SSP2−4.5 (+2.6°C)
SSP5−8.5 (+4.5°C)
log(CPUE)
Latitude
Longitude
80°N
75°N
70°N
65°N
60°N
55°N
50°N
0°
10°E
20°E
30°E
40°E
50°E
N

Enchelyopus cimbrius

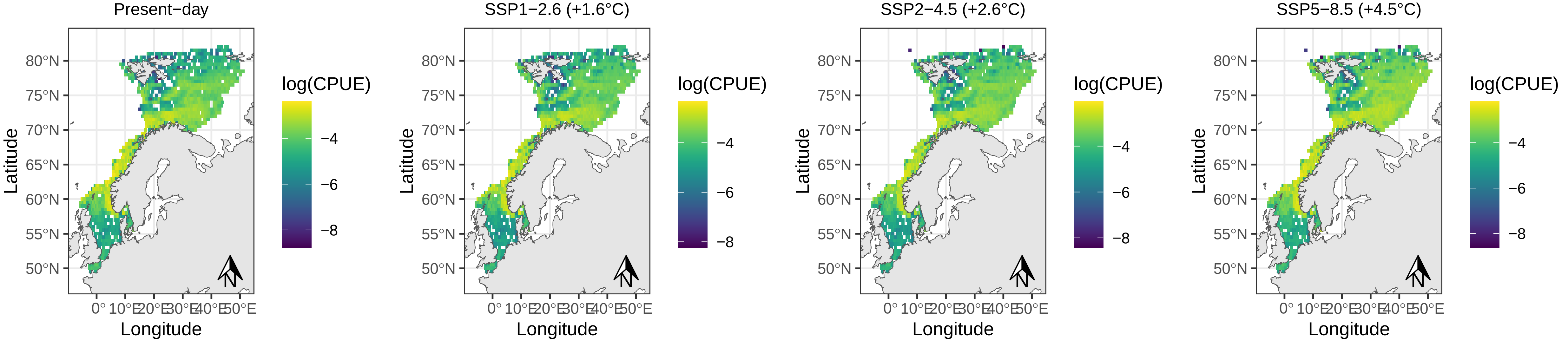

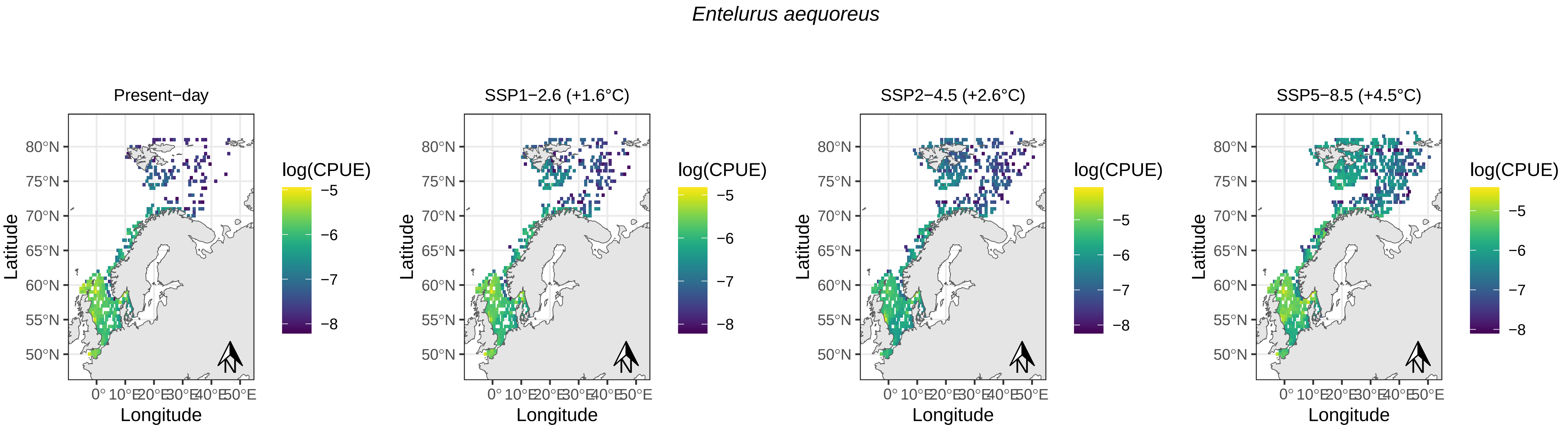

Entelurus aequoreus
Present−day
SSP1−2.6 (+1.6°C)
SSP2−4.5 (+2.6°C)
SSP5−8.5 (+4.5°C)
log(CPUE)
Latitude
Longitude
80°N
75°N
70°N
65°N
60°N
55°N
50°N
0°
10°E
20°E
30°E
40°E
50°E
−5
−6
−7
−8
N

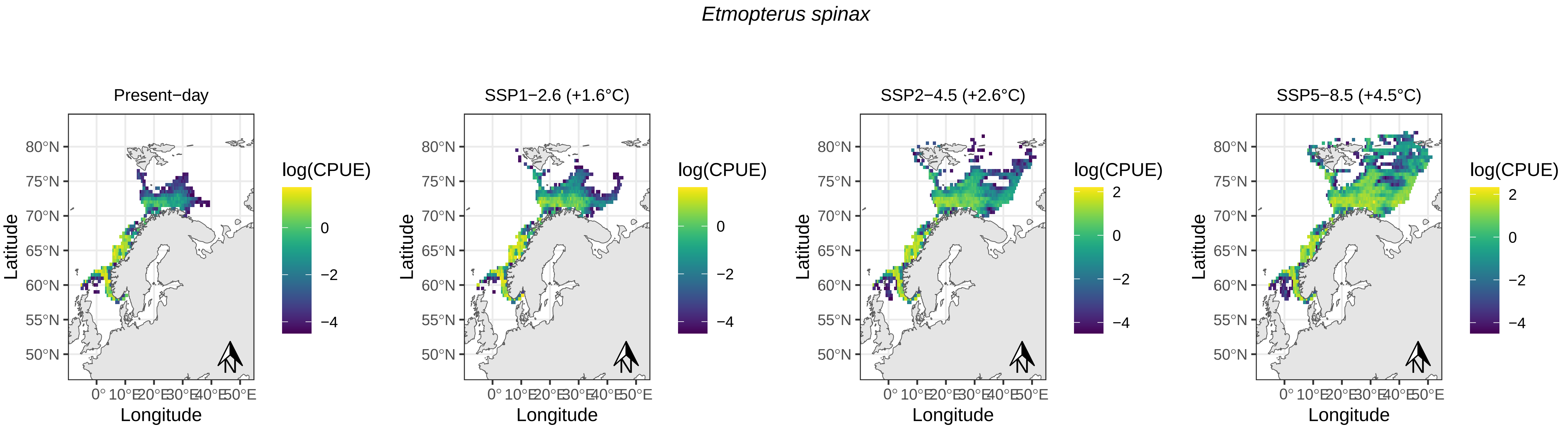

Etmopterus spinax
Present−day
SSP1−2.6 (+1.6°C)
SSP2−4.5 (+2.6°C)
SSP5−8.5 (+4.5°C)
log(CPUE)
Latitude
Longitude
80°N
75°N
70°N
65°N
60°N
55°N
50°N
0° 10°E 20°E 30°E 40°E 50°E
N

Eutrigla gurnardus

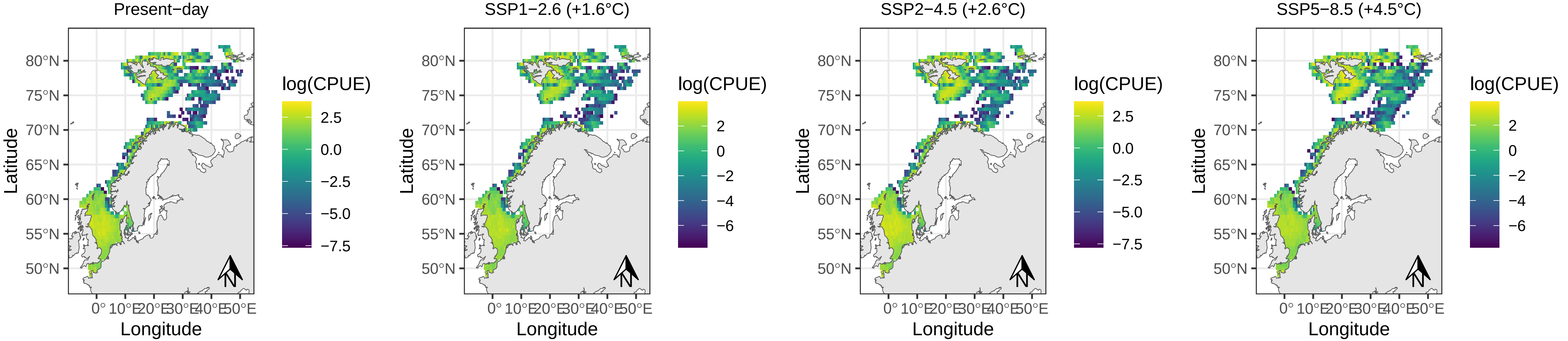

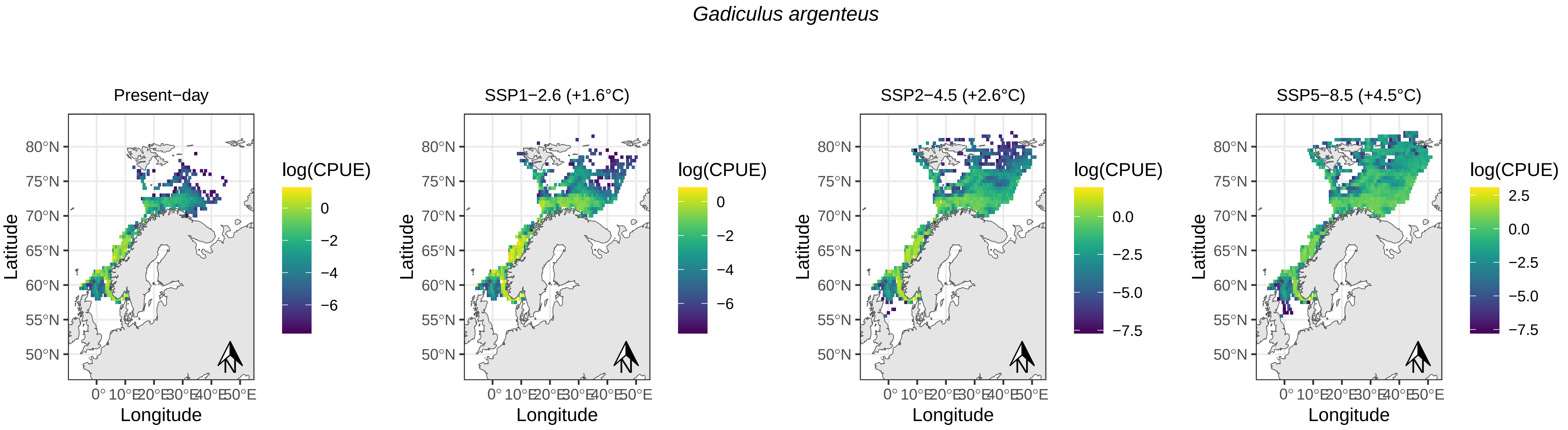

Gadiculus argenteus
Present−day
SSP1−2.6 (+1.6°C)
SSP2−4.5 (+2.6°C)
SSP5−8.5 (+4.5°C)
log(CPUE)
Latitude
Longitude
80°N
75°N
70°N
65°N
60°N
55°N
50°N
0° 10°E 20°E 30°E 40°E 50°E
0
−2
−4
−6
0.0
−2.5
−5.0
−7.5
2.5
N

Gadus morhua

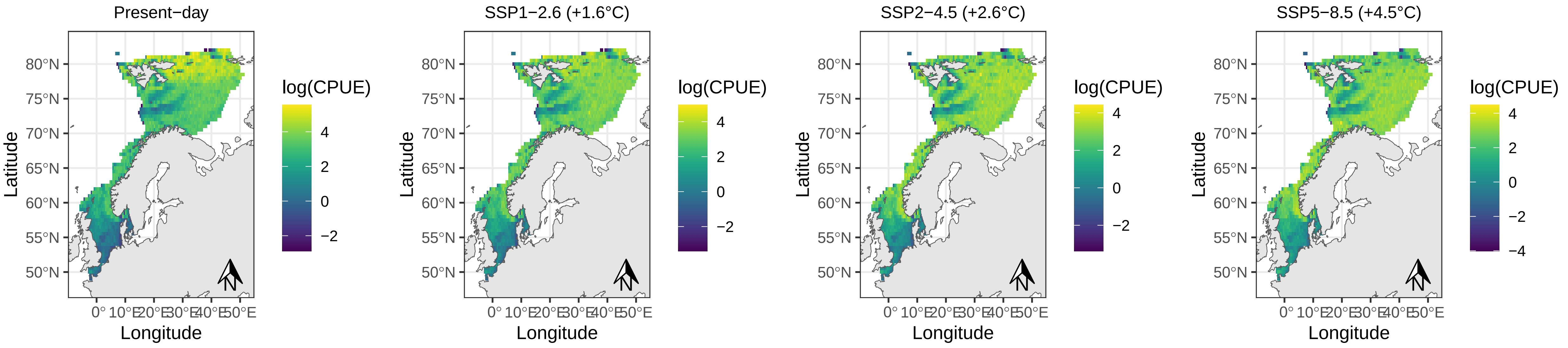

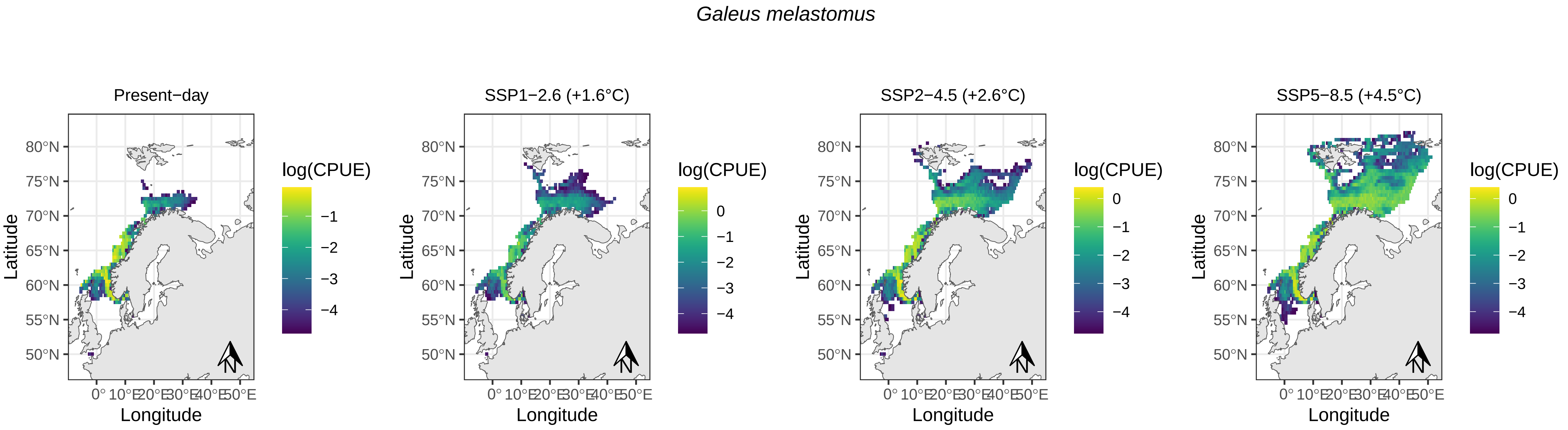

Galeus melastomus
Present−day
SSP1−2.6 (+1.6°C)
SSP2−4.5 (+2.6°C)
SSP5−8.5 (+4.5°C)
Latitude
Longitude
80°N
75°N
70°N
65°N
60°N
55°N
50°N
0° 10°E 20°E 30°E 40°E 50°E
log(CPUE)
0
−1
−2
−3
−4
N

Glyptocephalus cynoglossus

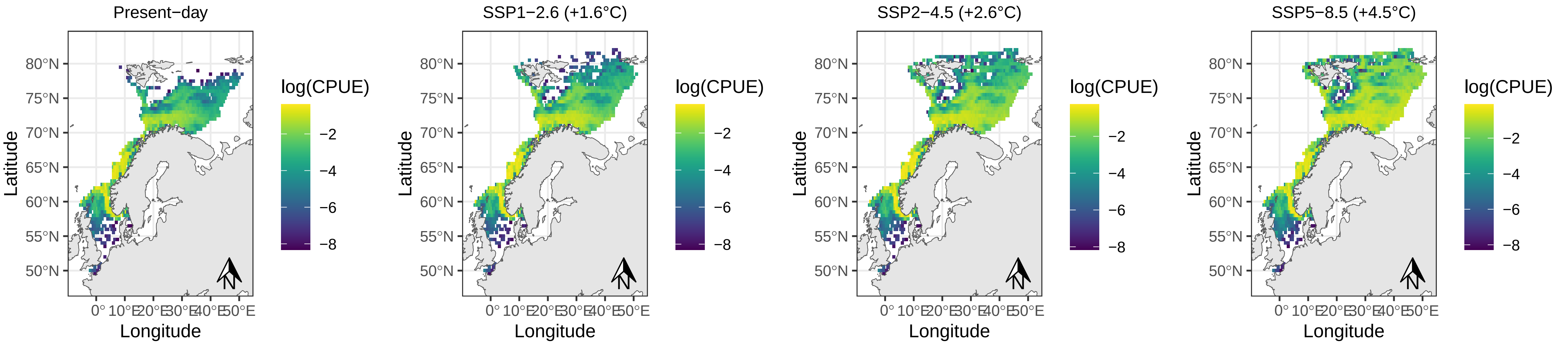

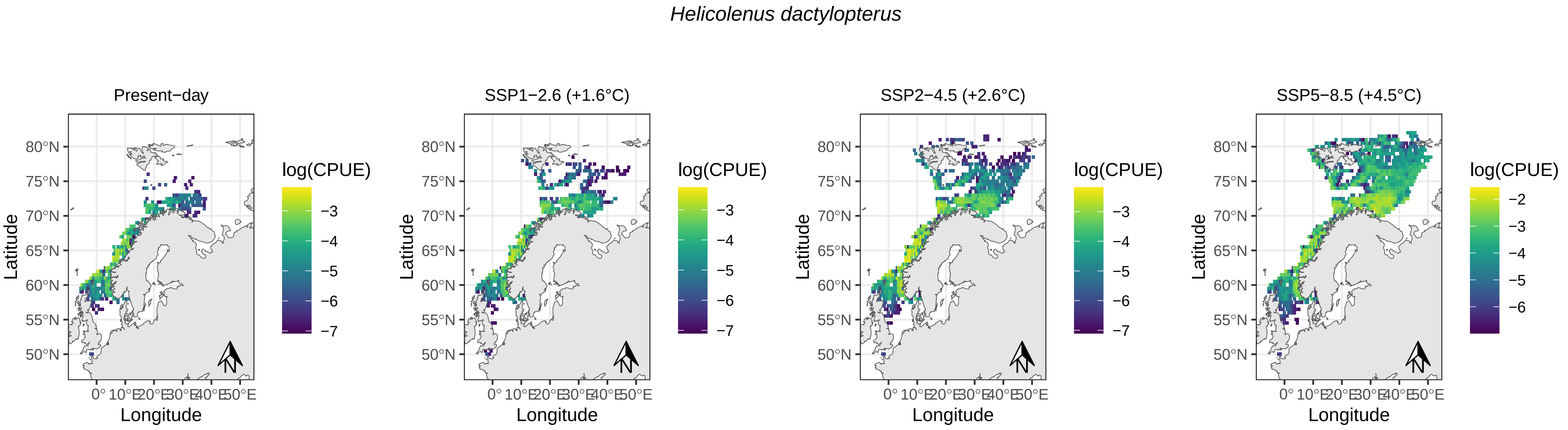

Helicolenus dactylopterus
Present−day
SSP1−2.6 (+1.6°C)
SSP2−4.5 (+2.6°C)
SSP5−8.5 (+4.5°C)
log(CPUE)
Latitude
Longitude
80°N
75°N
70°N
65°N
60°N
55°N
50°N
0°
10°E
20°E
30°E
40°E
50°E
−2
−3
−4
−5
−6
−7
N

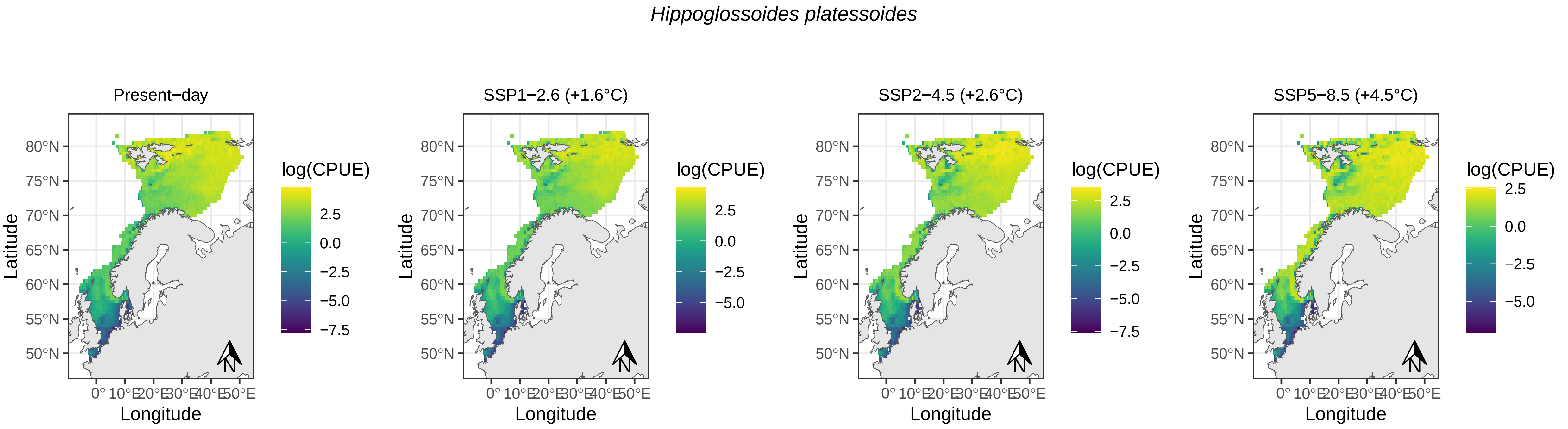

Hippoglossoides platessoides
Present−day
SSP1−2.6 (+1.6°C)
SSP2−4.5 (+2.6°C)
SSP5−8.5 (+4.5°C)
log(CPUE)
Latitude
Longitude
80°N
75°N
70°N
65°N
60°N
55°N
50°N
0° 10°E 20°E 30°E 40°E 50°E
2.5
0.0
−2.5
−5.0
−7.5
N

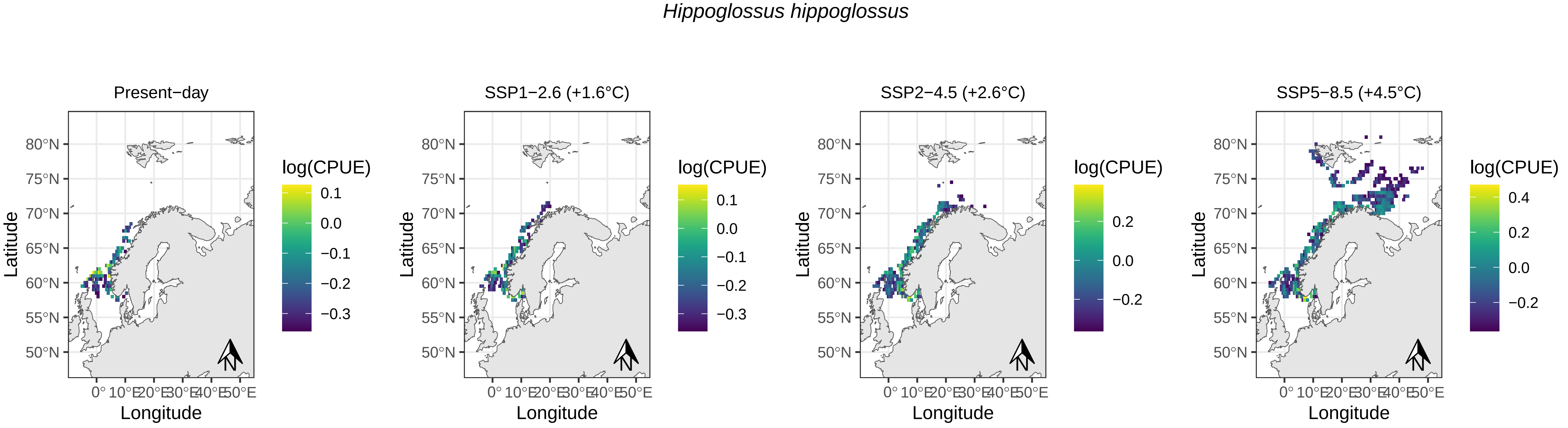

Hippoglossus hippoglossus
Present−day
SSP1−2.6 (+1.6°C)
SSP2−4.5 (+2.6°C)
SSP5−8.5 (+4.5°C)
Latitude
Longitude
80°N
75°N
70°N
65°N
60°N
55°N
50°N
0° 10°E 20°E 30°E 40°E 50°E
log(CPUE)
0.1
0.0
−0.1
−0.2
−0.3
0.2
0.4
N

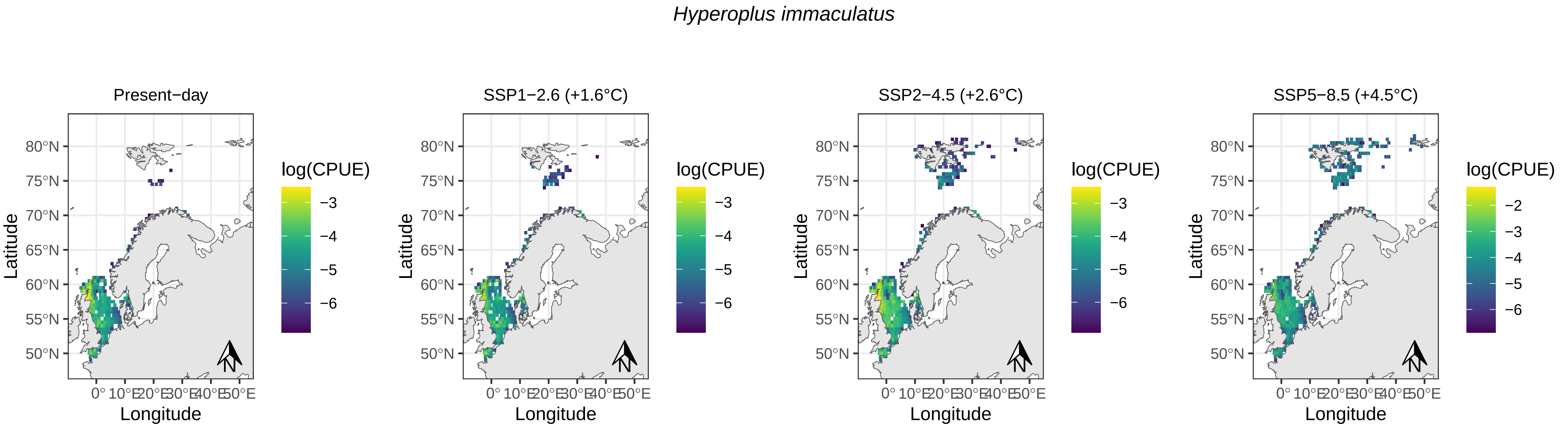
Hyperoplus immaculatus
Present−day
SSP1−2.6 (+1.6°C)
SSP2−4.5 (+2.6°C)
SSP5−8.5 (+4.5°C)
log(CPUE)
80°N
75°N
70°N
65°N
60°N
55°N
50°N
Latitude
0° 10°E 20°E 30°E 40°E 50°E
Longitude
N
−2
−3
−4
−5
−6

Lepidorhombus whiffiagonis

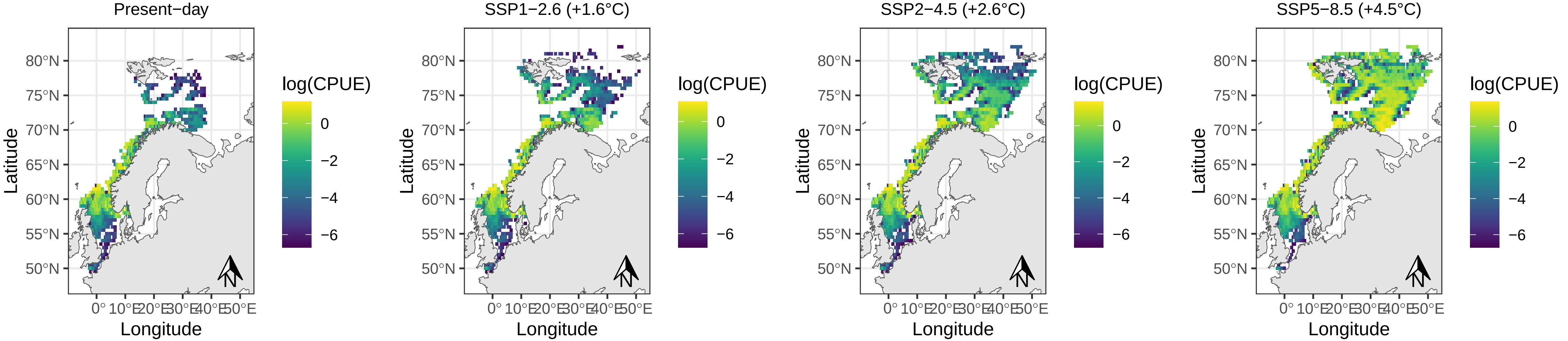

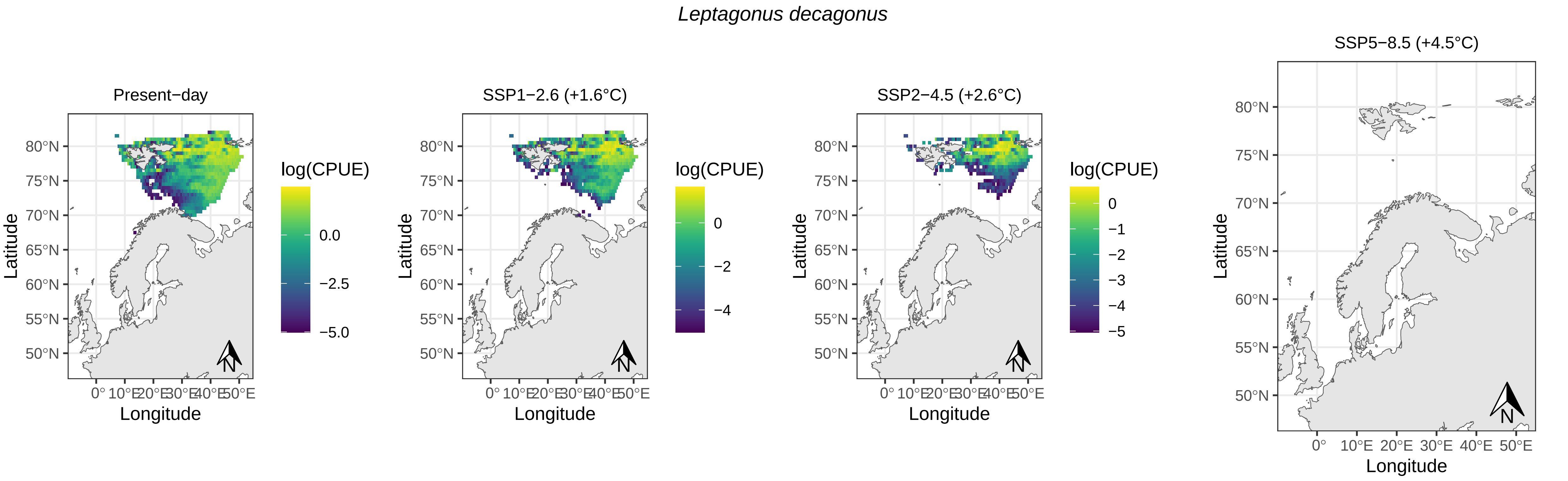

Leptagonus decagonus
Present−day
SSP1−2.6 (+1.6°C)
SSP2−4.5 (+2.6°C)
SSP5−8.5 (+4.5°C)
log(CPUE)
0.0
−2.5
−5.0
0
−2
−4
−1
−3
−5
Latitude
Longitude
80°N
75°N
70°N
65°N
60°N
55°N
50°N
0°
10°E
20°E
30°E
40°E
50°E
N

Leptoclinus maculatus

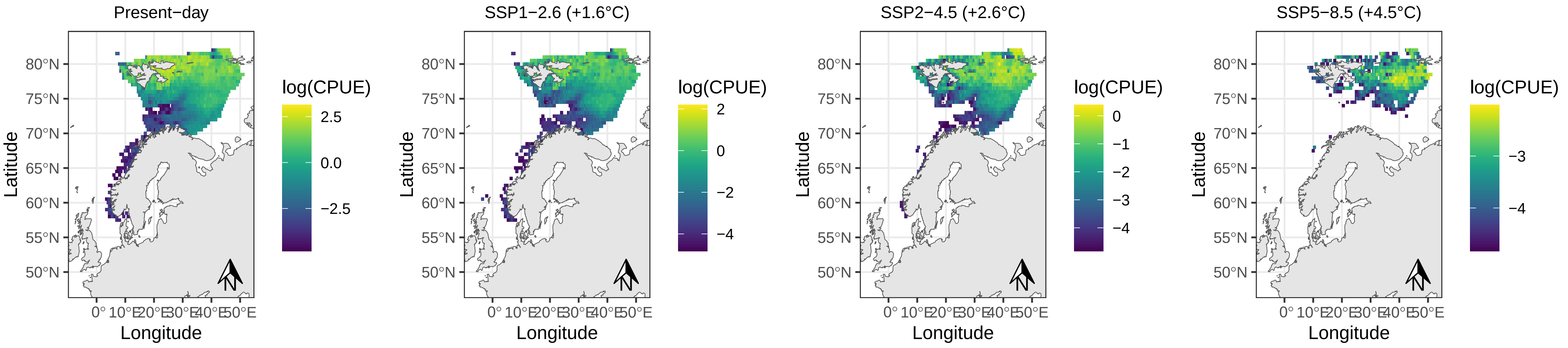

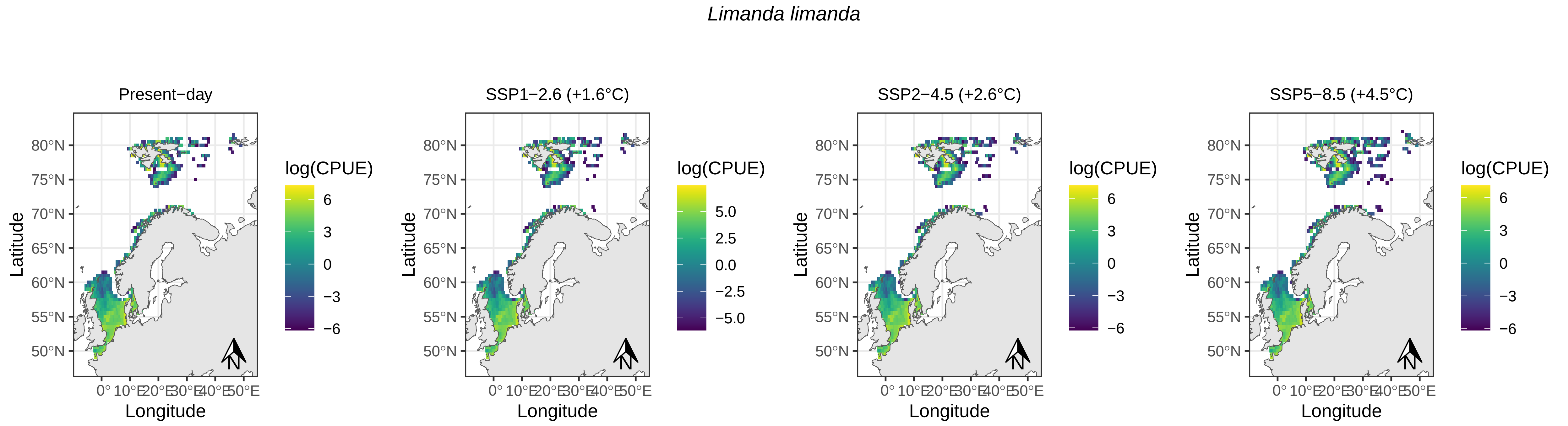

Limanda limanda
Present−day
SSP1−2.6 (+1.6°C)
SSP2−4.5 (+2.6°C)
SSP5−8.5 (+4.5°C)
log(CPUE)
Latitude
Longitude
80°N
75°N
70°N
65°N
60°N
55°N
50°N
0°
10°E
20°E
30°E
40°E
50°E
6
3
0
−3
−6
5.0
2.5
0.0
−2.5
−5.0
N

Lophius piscatorius

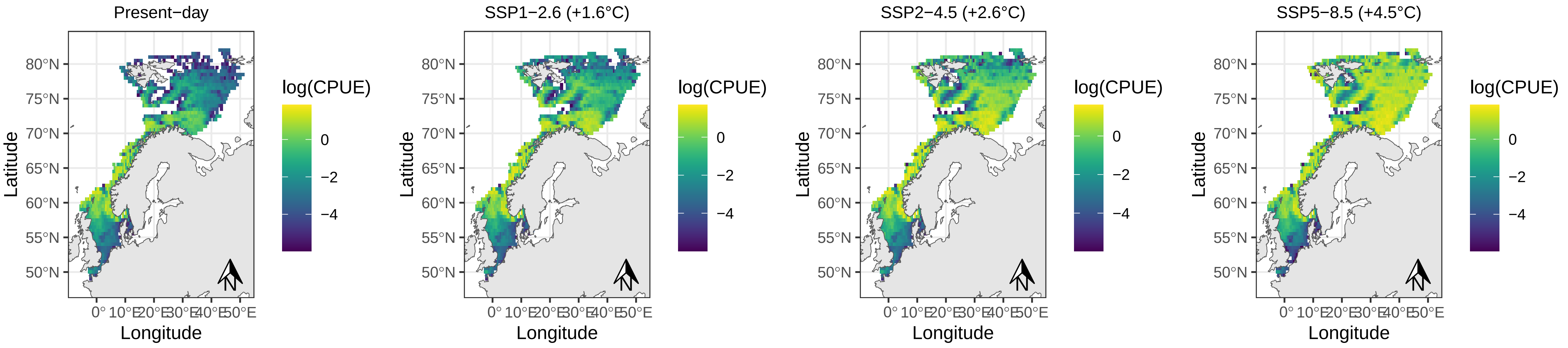

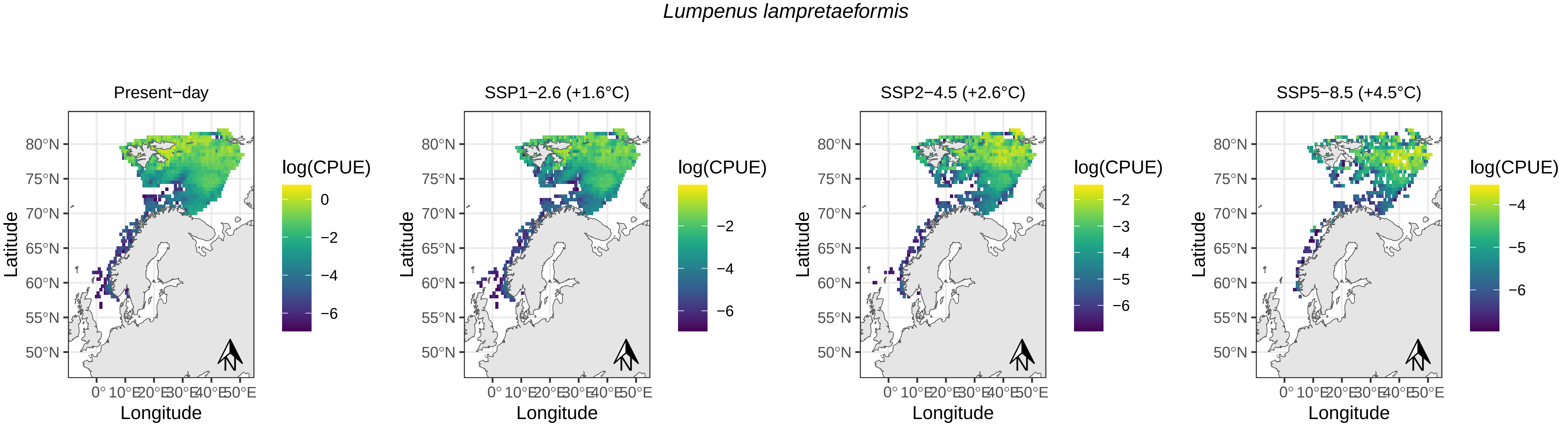

Lumpenus lampretaeformis
Present−day
SSP1−2.6 (+1.6°C)
SSP2−4.5 (+2.6°C)
SSP5−8.5 (+4.5°C)
log(CPUE)
Latitude
Longitude
80°N
75°N
70°N
65°N
60°N
55°N
50°N
0° 10°E 20°E 30°E 40°E 50°E
N

Lycodes gracilis

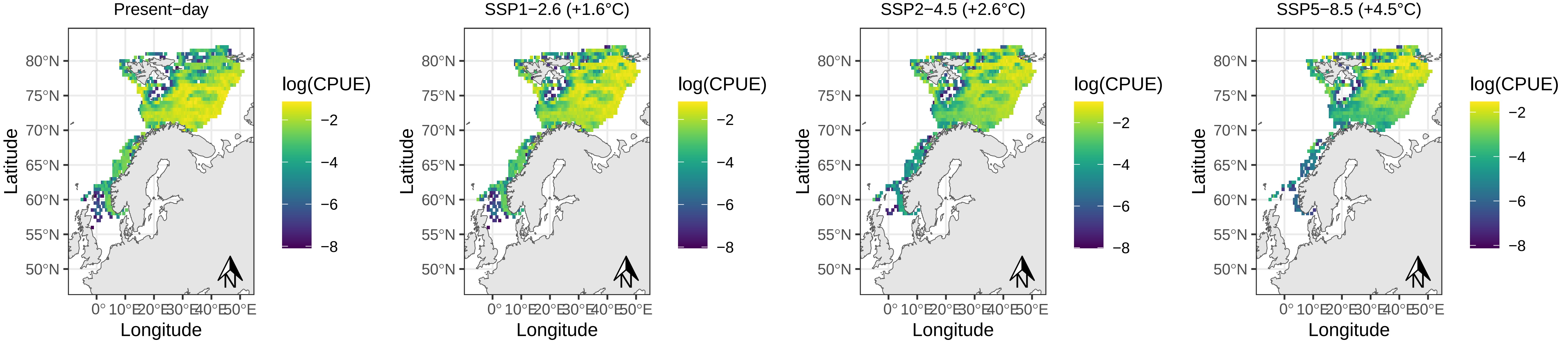

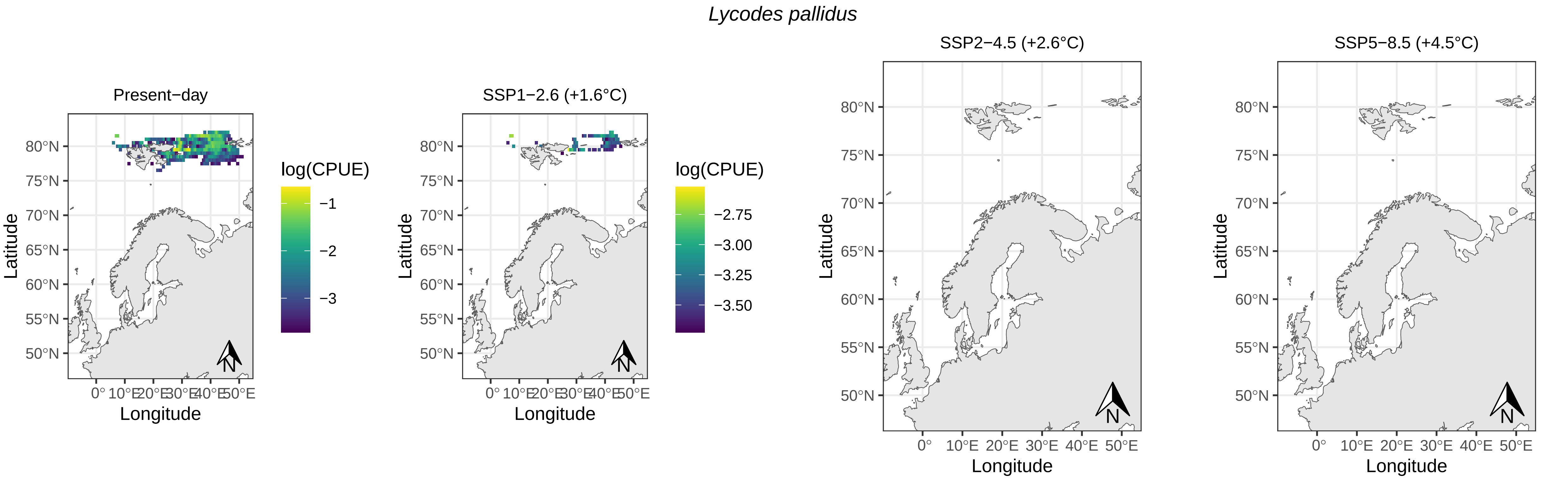

Lycodes pallidus
Present−day
SSP1−2.6 (+1.6°C)
SSP2−4.5 (+2.6°C)
SSP5−8.5 (+4.5°C)
log(CPUE)
−1
−2
−3
log(CPUE)
−2.75
−3.00
−3.25
−3.50
80°N
75°N
70°N
65°N
60°N
55°N
50°N
Latitude
0°
10°E
20°E
30°E
40°E
50°E
Longitude
N

Lycodes vahlii

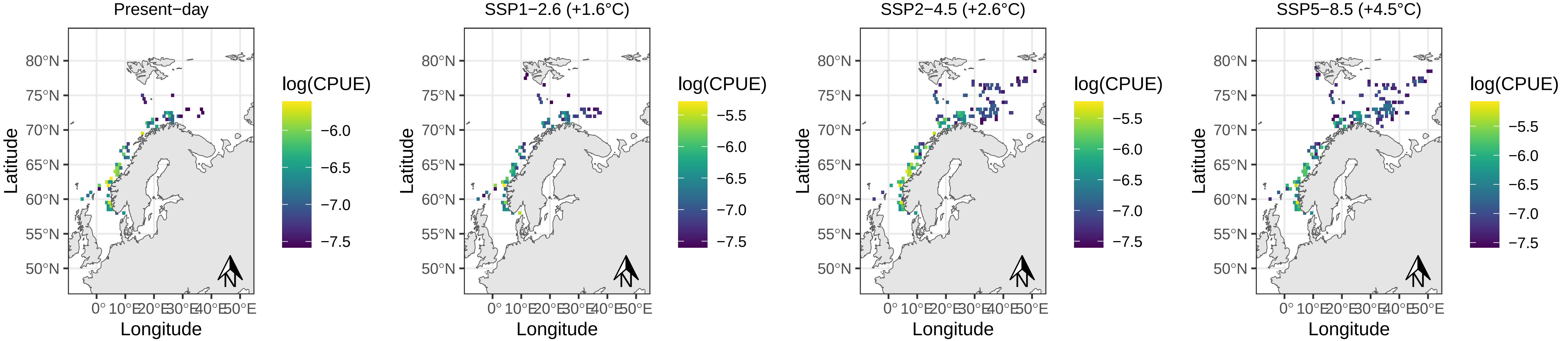

Mallotus villosus

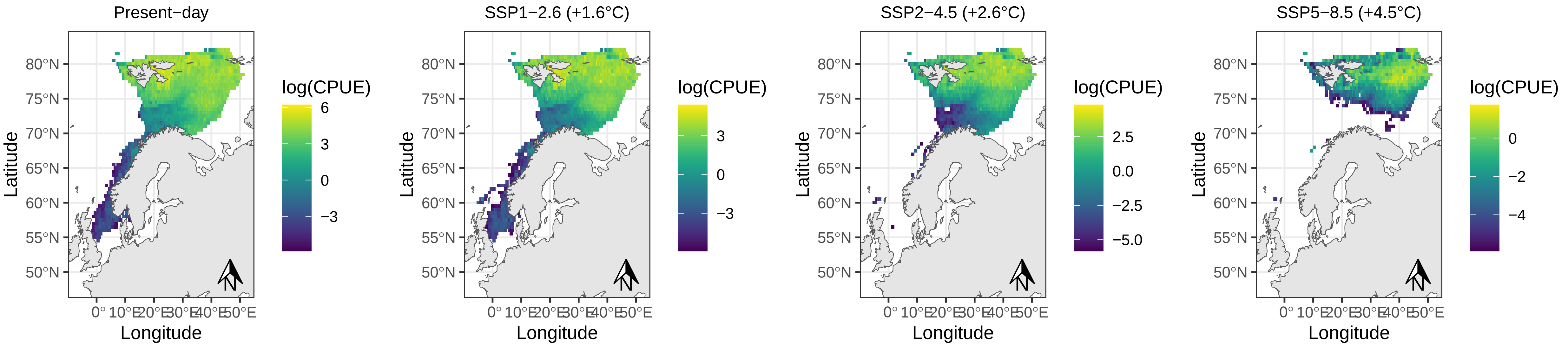

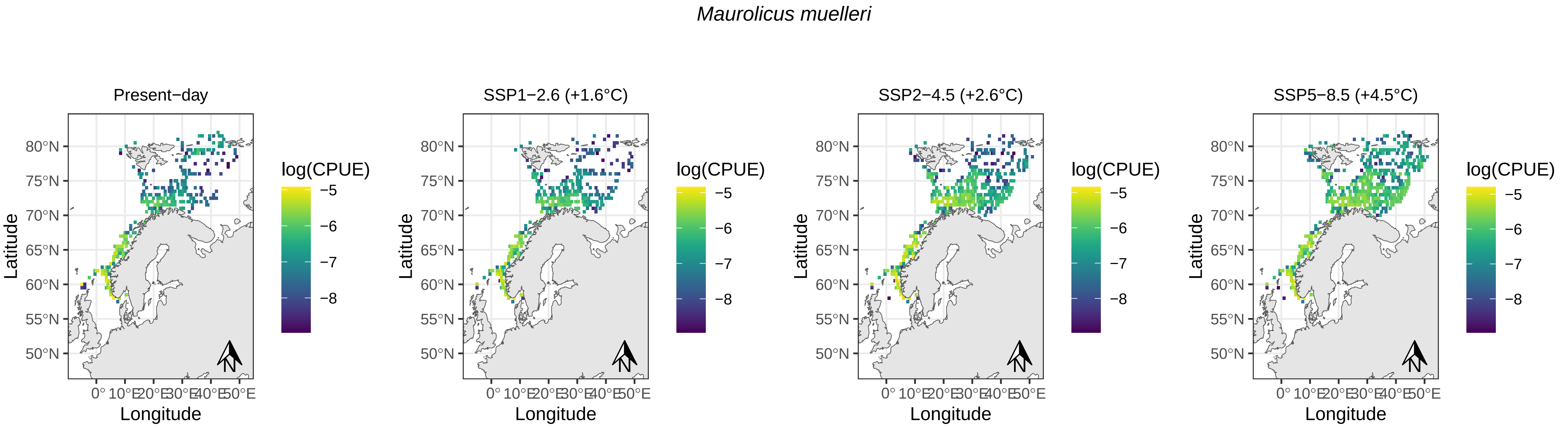

Maurolicus muelleri
Present−day
SSP1−2.6 (+1.6°C)
SSP2−4.5 (+2.6°C)
SSP5−8.5 (+4.5°C)
80°N
75°N
70°N
65°N
60°N
55°N
50°N
Latitude
0°
10°E
20°E
30°E
40°E
50°E
Longitude
N
log(CPUE)
−5
−6
−7
−8

Melanogrammus aeglefinus

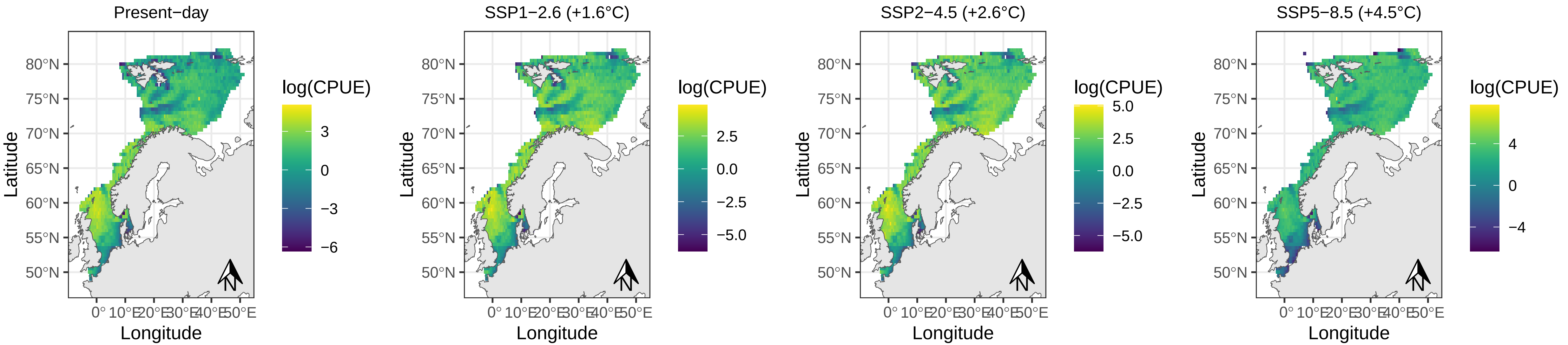

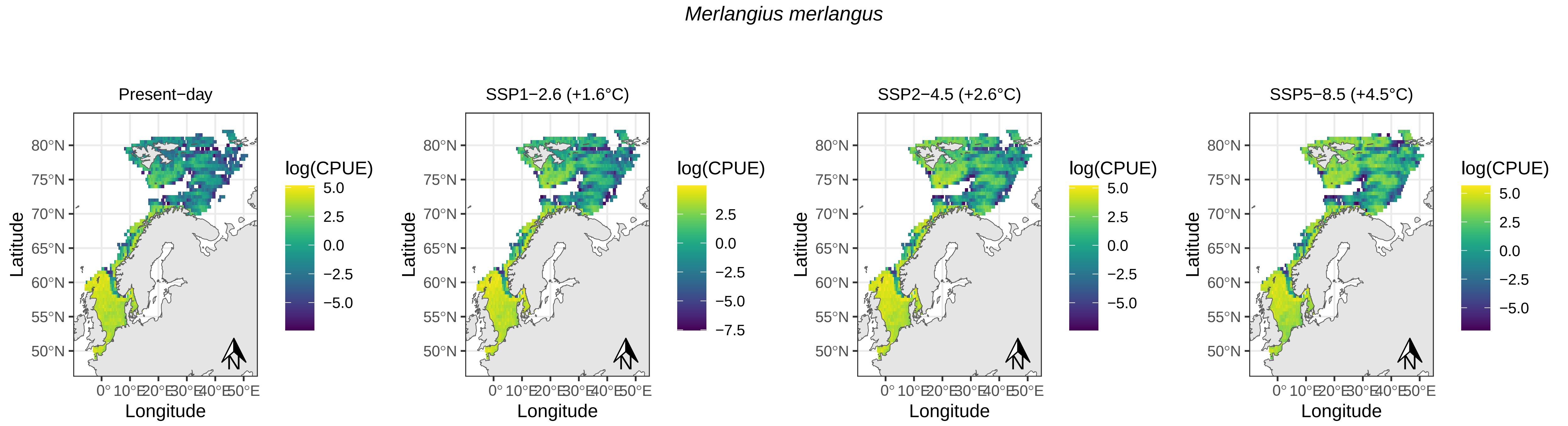

Merlangius merlangus
Present−day
SSP1−2.6 (+1.6°C)
SSP2−4.5 (+2.6°C)
SSP5−8.5 (+4.5°C)
log(CPUE)
5.0
2.5
0.0
−2.5
−5.0
−7.5
Latitude
80°N
75°N
70°N
65°N
60°N
55°N
50°N
Longitude
0°
10°E
20°E
30°E
40°E
50°E
N

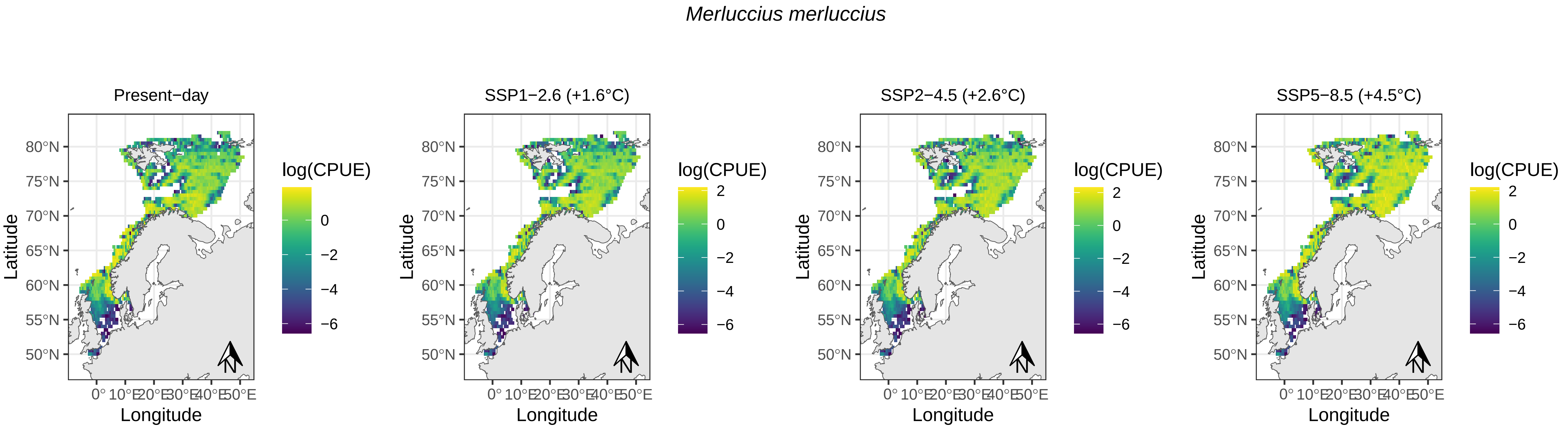

Merluccius merluccius
Present−day
SSP1−2.6 (+1.6°C)
SSP2−4.5 (+2.6°C)
SSP5−8.5 (+4.5°C)
Latitude
Longitude
80°N
75°N
70°N
65°N
60°N
55°N
50°N
0° 10°E 20°E 30°E 40°E 50°E
log(CPUE)
2
0
−2
−4
−6
N

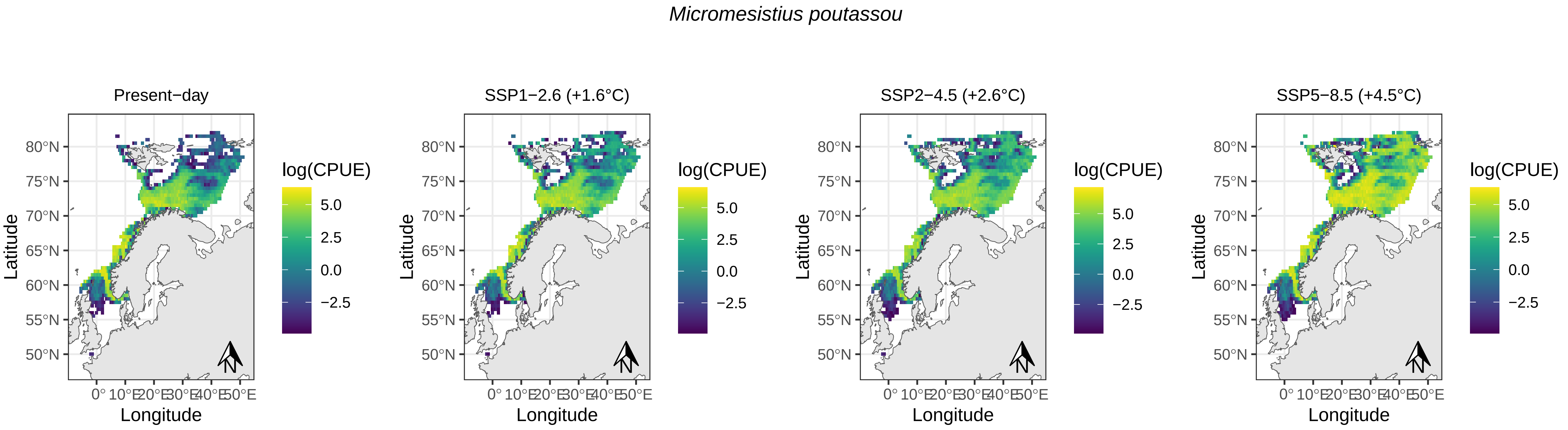
Micromesistius poutassou
Present−day
SSP1−2.6 (+1.6°C)
SSP2−4.5 (+2.6°C)
SSP5−8.5 (+4.5°C)
Latitude
Longitude
80°N
75°N
70°N
65°N
60°N
55°N
50°N
0°
10°E
20°E
30°E
40°E
50°E
log(CPUE)
5.0
2.5
0.0
−2.5
N

Microstomus kitt

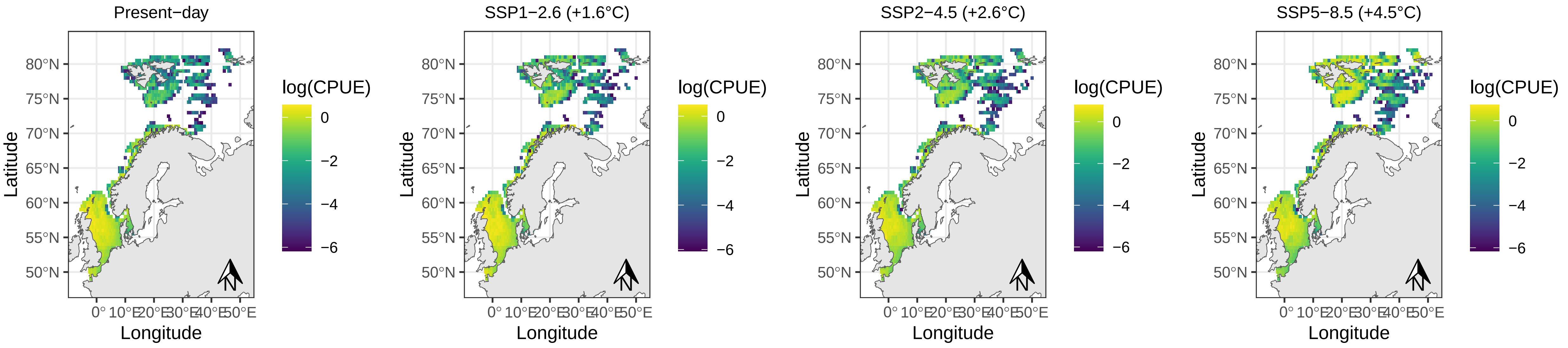

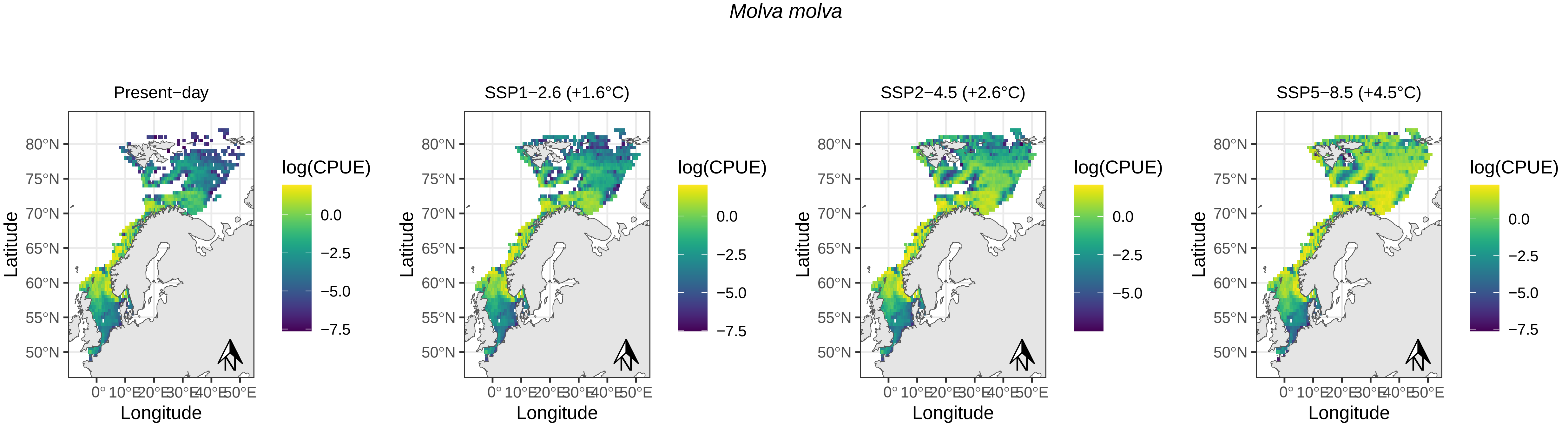

Molva molva
Present−day
SSP1−2.6 (+1.6°C)
SSP2−4.5 (+2.6°C)
SSP5−8.5 (+4.5°C)
log(CPUE)
0.0
−2.5
−5.0
−7.5
Latitude
Longitude
80°N
75°N
70°N
65°N
60°N
55°N
50°N
0°
10°E
20°E
30°E
40°E
50°E
N

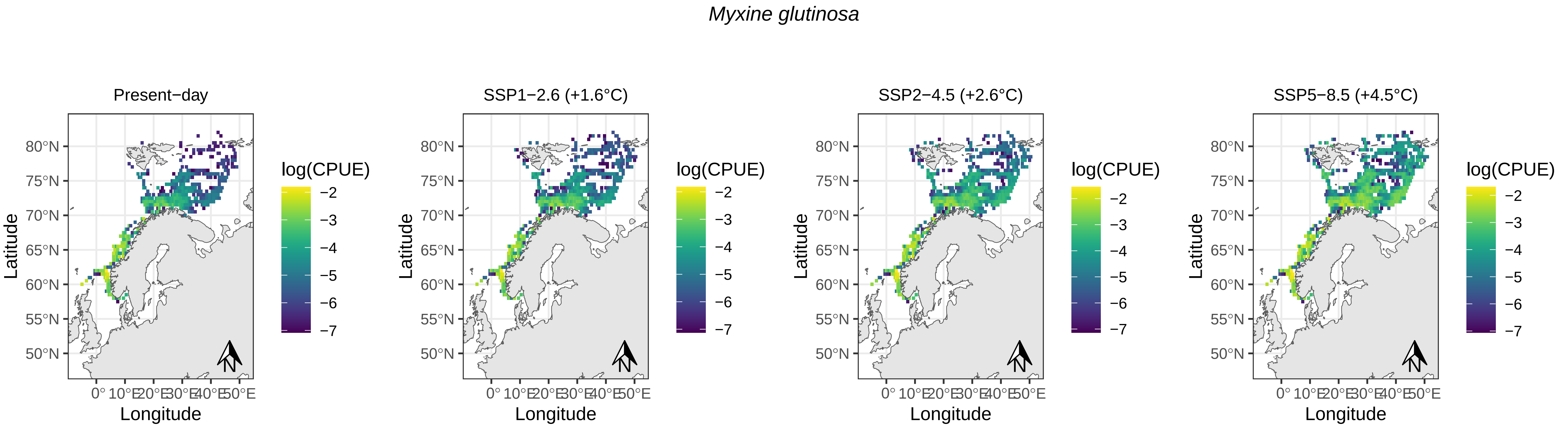

Myxine glutinosa
Present−day
SSP1−2.6 (+1.6°C)
SSP2−4.5 (+2.6°C)
SSP5−8.5 (+4.5°C)
log(CPUE)
−2
−3
−4
−5
−6
−7
80°N
75°N
70°N
65°N
60°N
55°N
50°N
0°
10°E
20°E
30°E
40°E
50°E
Latitude
Longitude
N

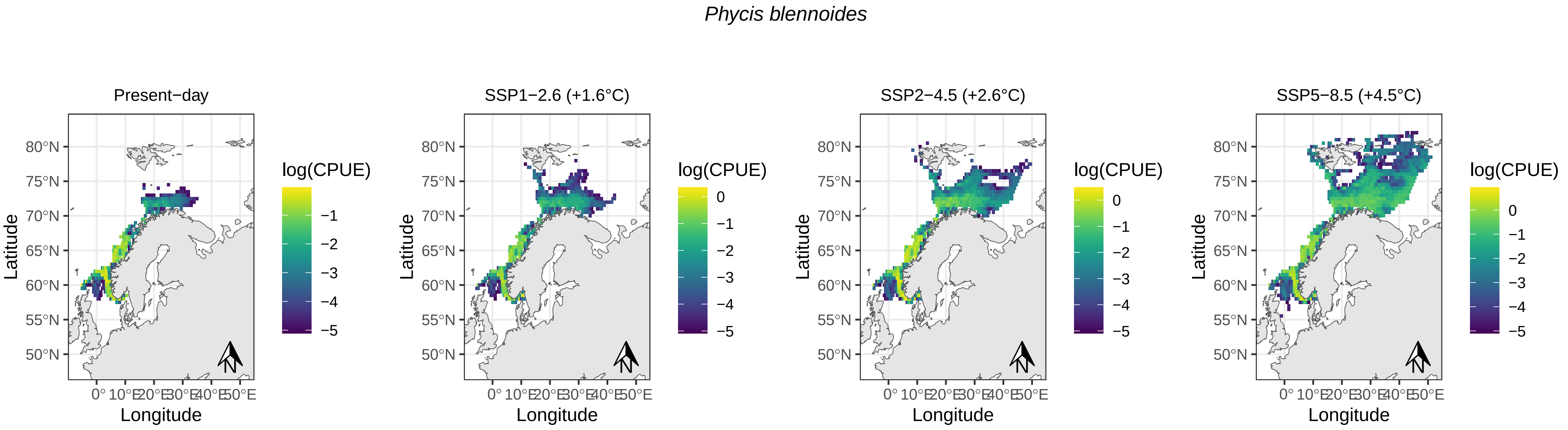

Phycis blennoides
Present−day
SSP1−2.6 (+1.6°C)
SSP2−4.5 (+2.6°C)
SSP5−8.5 (+4.5°C)
Latitude
Longitude
80°N
75°N
70°N
65°N
60°N
55°N
50°N
0° 10°E 20°E 30°E 40°E 50°E
log(CPUE)
0
−1
−2
−3
−4
−5
N

Pollachius pollachius

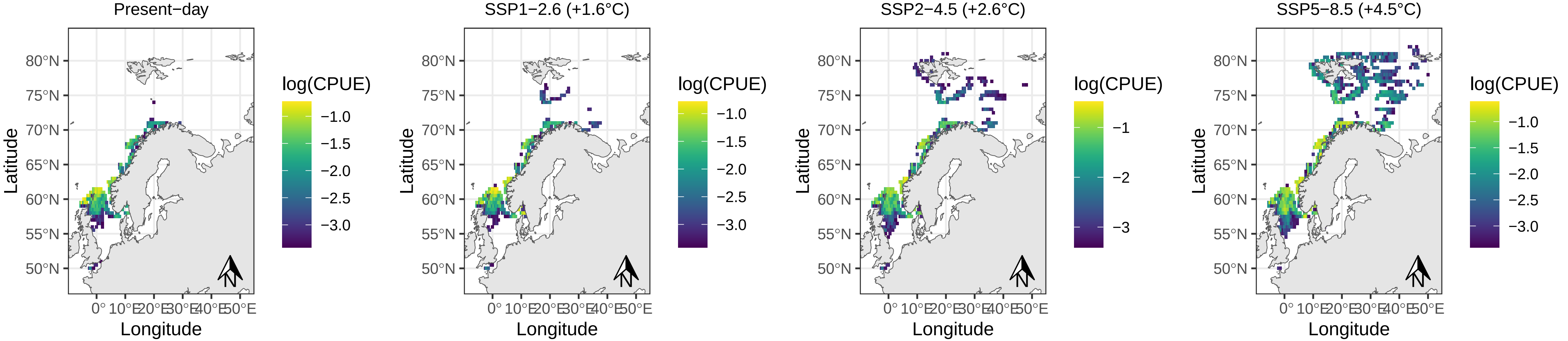

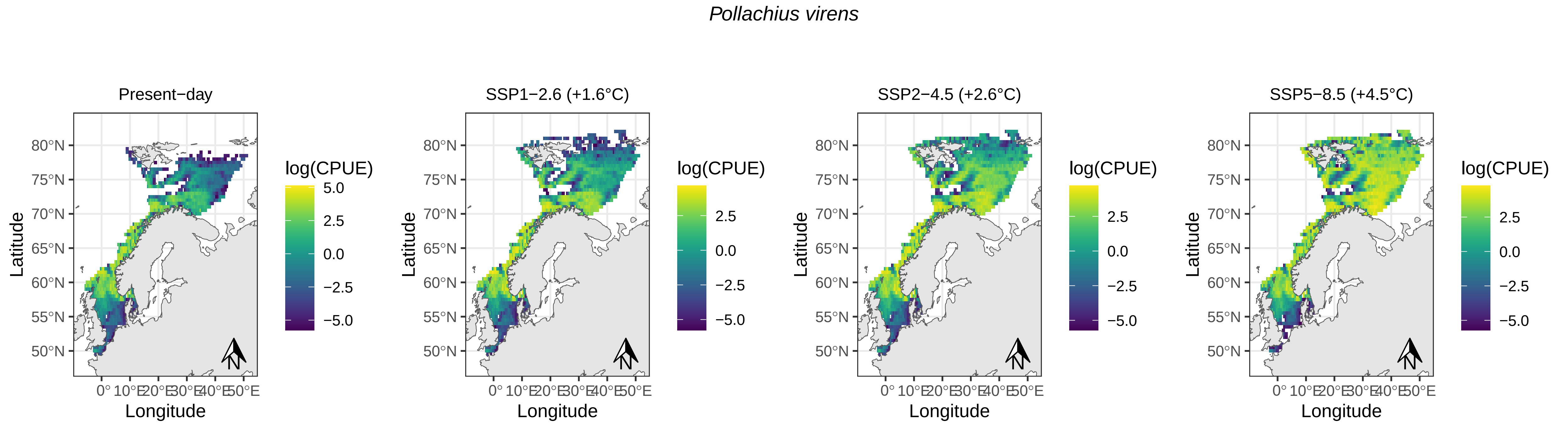

Pollachius virens
Present−day
SSP1−2.6 (+1.6°C)
SSP2−4.5 (+2.6°C)
SSP5−8.5 (+4.5°C)
log(CPUE)
5.0
2.5
0.0
−2.5
−5.0
80°N
75°N
70°N
65°N
60°N
55°N
50°N
Latitude
0°
10°E
20°E
30°E
40°E
50°E
Longitude
N

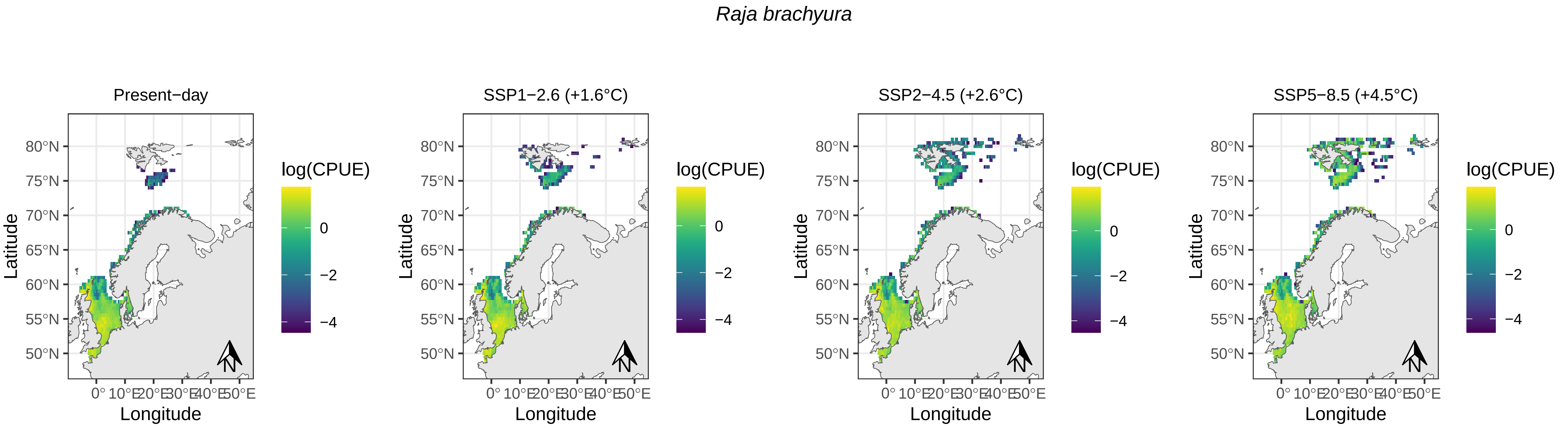
Raja brachyura
Present−day
SSP1−2.6 (+1.6°C)
SSP2−4.5 (+2.6°C)
SSP5−8.5 (+4.5°C)
log(CPUE)
0
−2
−4
80°N
75°N
70°N
65°N
60°N
55°N
50°N
Latitude
0° 10°E 20°E 30°E 40°E 50°E
Longitude
N

Raja montagui

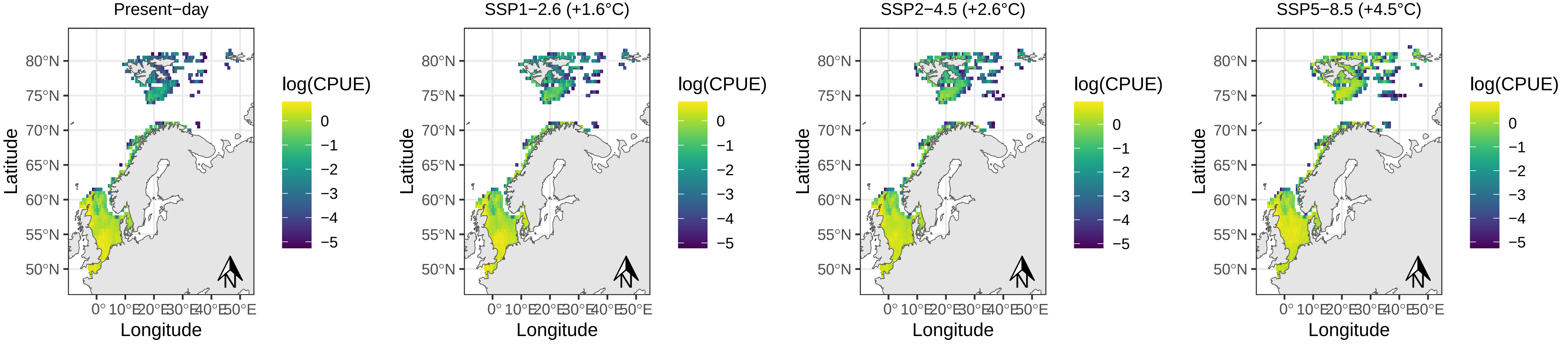

Rajella fyllae

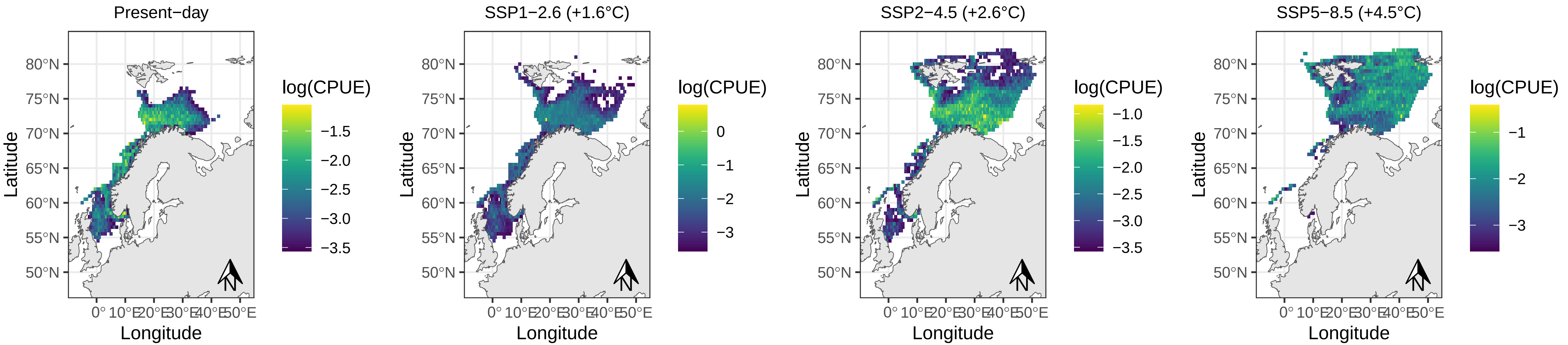

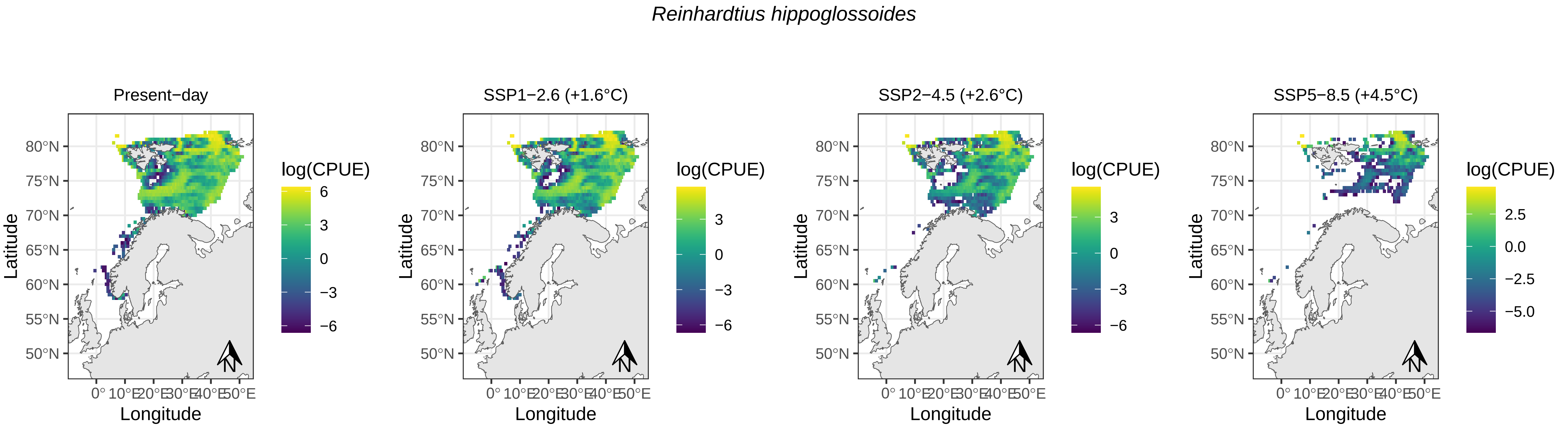

Reinhardtius hippoglossoides
Present−day
SSP1−2.6 (+1.6°C)
SSP2−4.5 (+2.6°C)
SSP5−8.5 (+4.5°C)
Latitude
Longitude
log(CPUE)
80°N
75°N
70°N
65°N
60°N
55°N
50°N
0°
10°E
20°E
30°E
40°E
50°E
6
3
0
−3
−6
2.5
0.0
−2.5
−5.0
N

Scophthalmus maximus

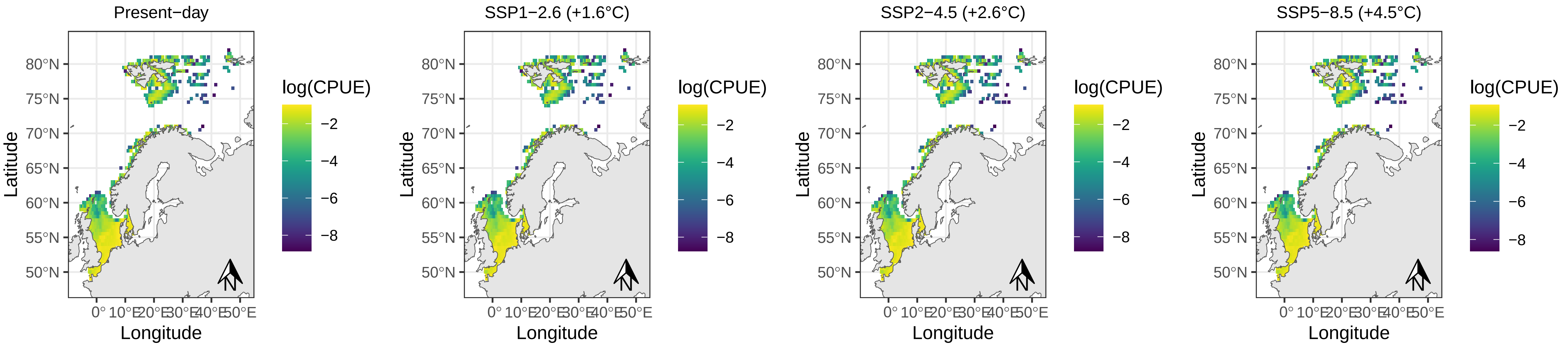

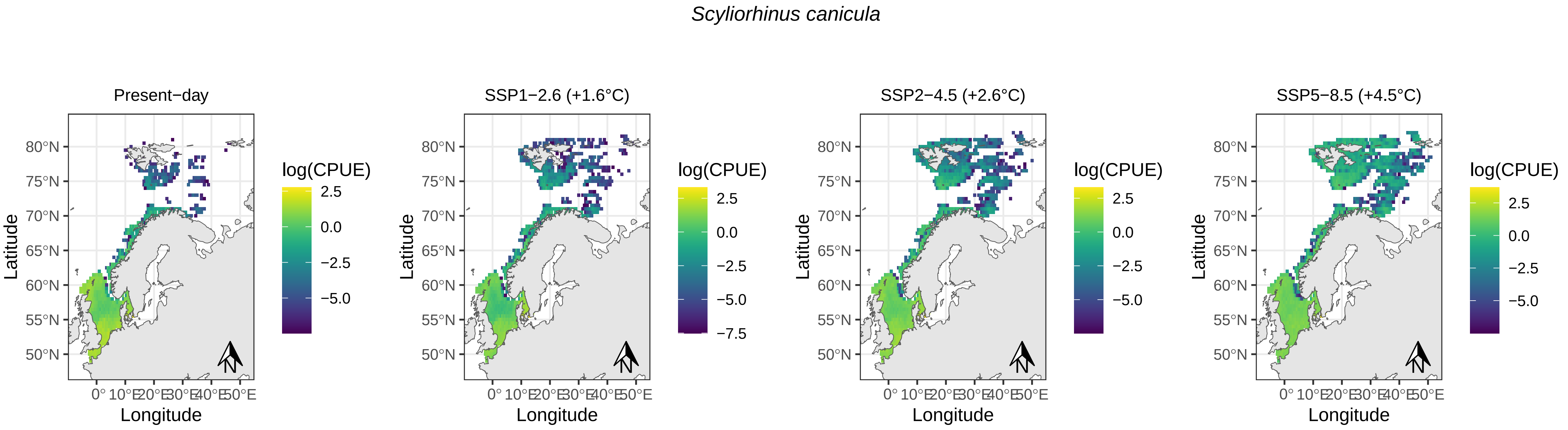

Scyliorhinus canicula
Present−day
SSP1−2.6 (+1.6°C)
SSP2−4.5 (+2.6°C)
SSP5−8.5 (+4.5°C)
Latitude
80°N
75°N
70°N
65°N
60°N
55°N
50°N
Longitude
0°
10°E
20°E
30°E
40°E
50°E
log(CPUE)
2.5
0.0
−2.5
−5.0
−7.5
N

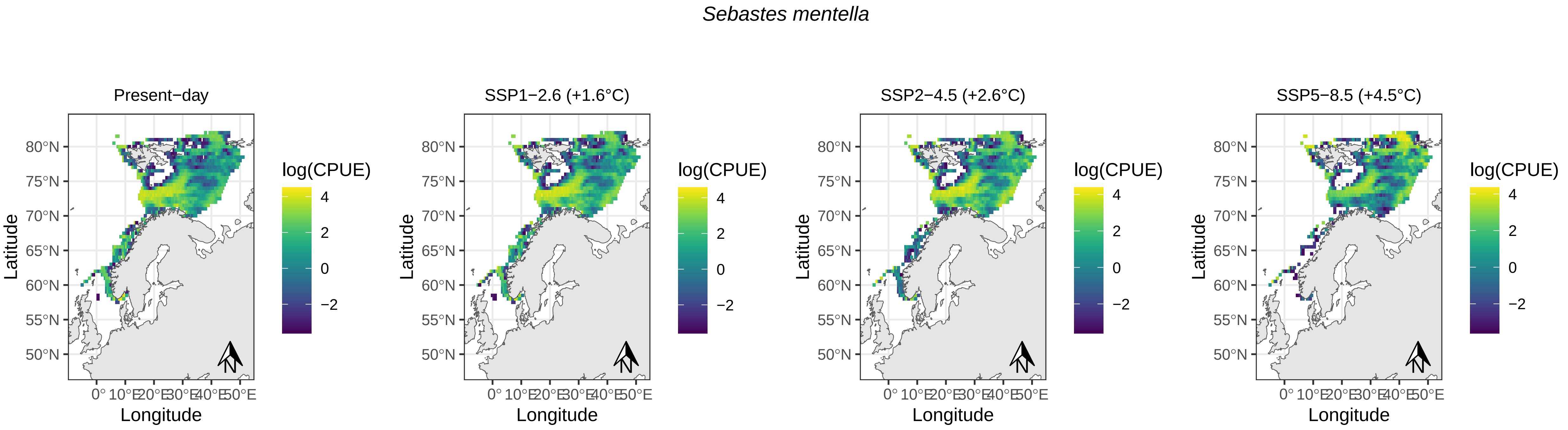

Sebastes mentella
Present−day
SSP1−2.6 (+1.6°C)
SSP2−4.5 (+2.6°C)
SSP5−8.5 (+4.5°C)
80°N
75°N
70°N
65°N
60°N
55°N
50°N
Latitude
0°
10°E
20°E
30°E
40°E
50°E
Longitude
log(CPUE)
4
2
0
−2
N

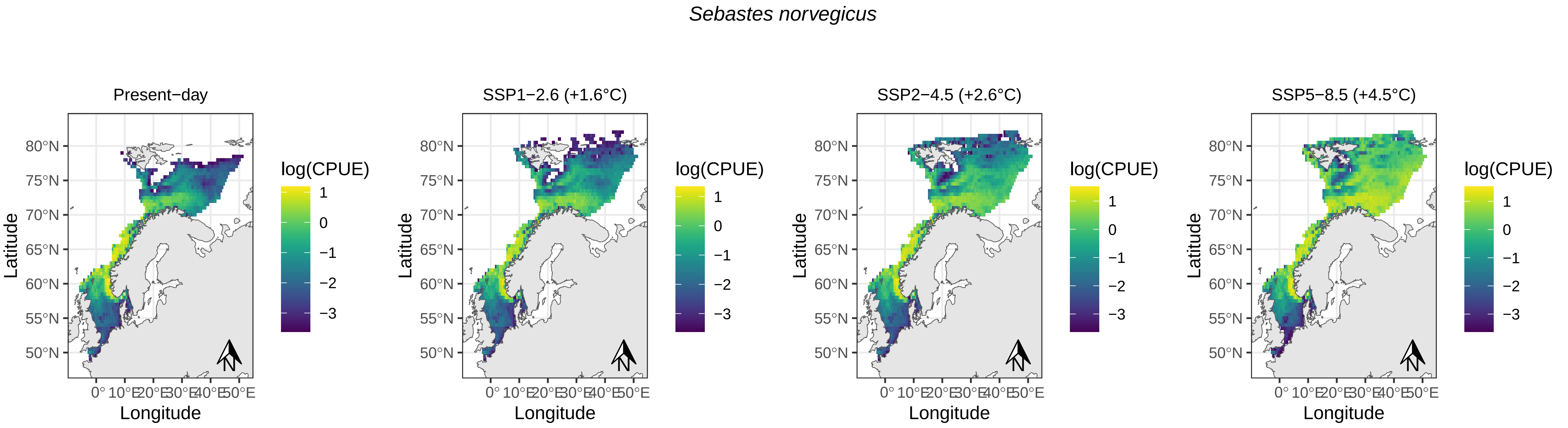
Sebastes norvegicus
Present−day
SSP1−2.6 (+1.6°C)
SSP2−4.5 (+2.6°C)
SSP5−8.5 (+4.5°C)
Latitude
80°N
75°N
70°N
65°N
60°N
55°N
50°N
Longitude
0°
10°E
20°E
30°E
40°E
50°E
log(CPUE)
1
0
−1
−2
−3
N

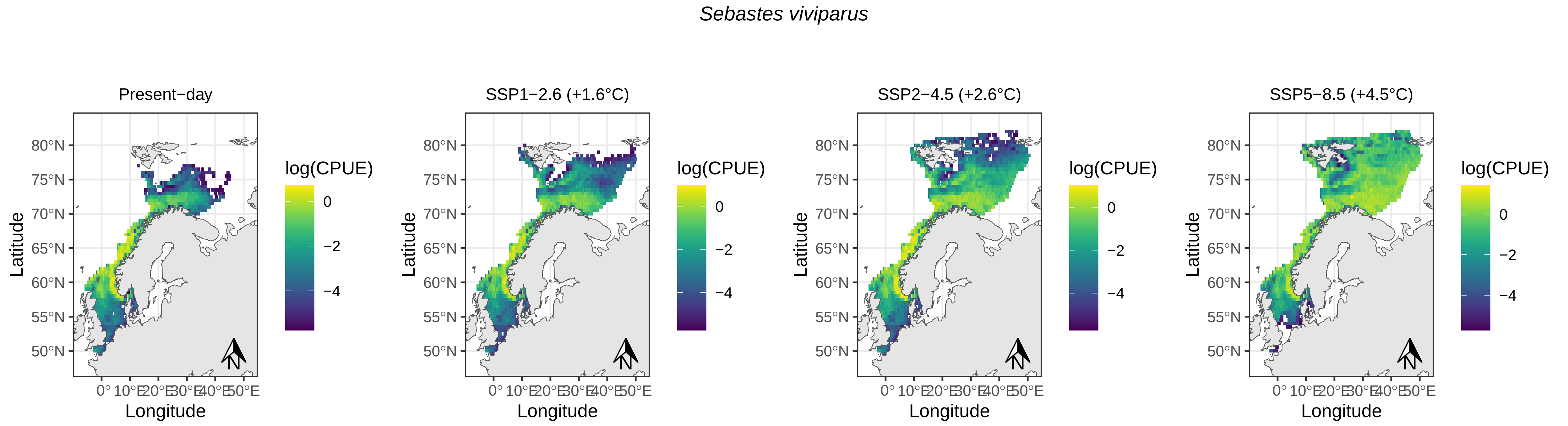
Sebastes viviparus
Present−day
SSP1−2.6 (+1.6°C)
SSP2−4.5 (+2.6°C)
SSP5−8.5 (+4.5°C)
log(CPUE)
Latitude
Longitude
80°N
75°N
70°N
65°N
60°N
55°N
50°N
0° 10°E 20°E 30°E 40°E 50°E
0
−2
−4
N

Sprattus sprattus

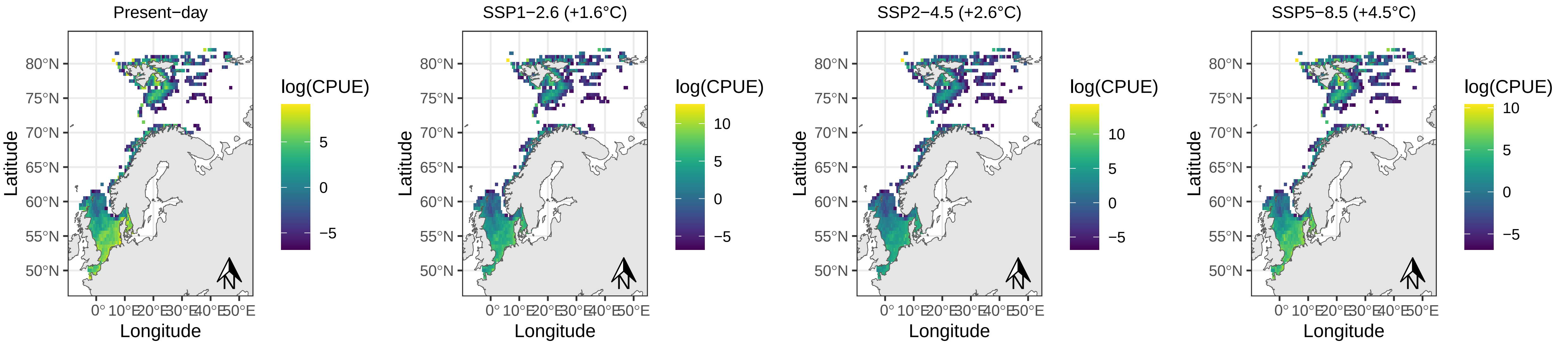

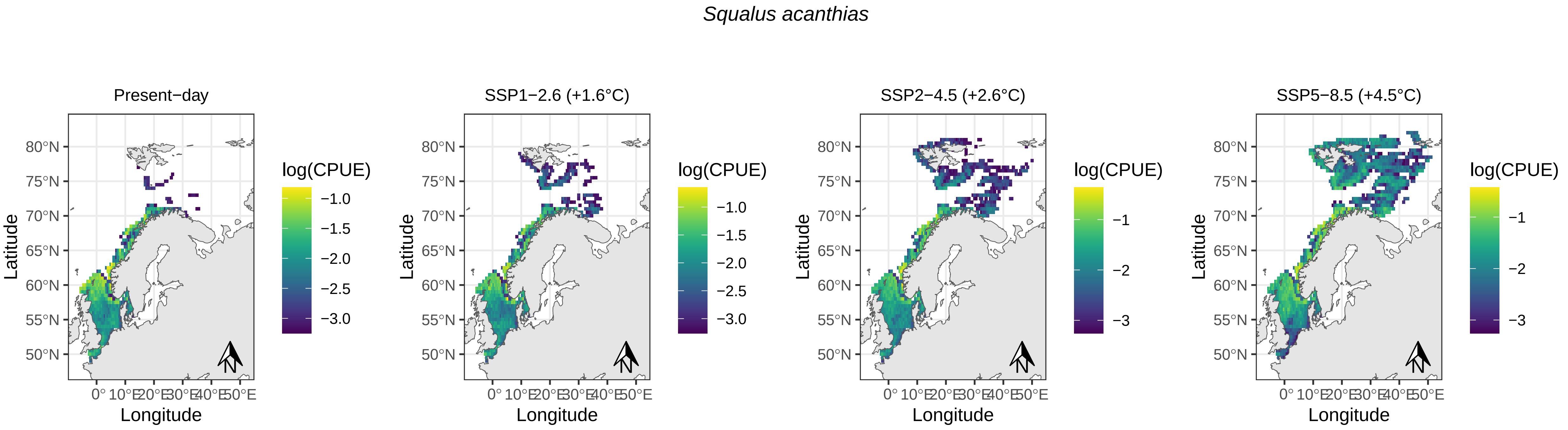

Squalus acanthias
Present−day
SSP1−2.6 (+1.6°C)
SSP2−4.5 (+2.6°C)
SSP5−8.5 (+4.5°C)
Latitude
Longitude
log(CPUE)
80°N
75°N
70°N
65°N
60°N
55°N
50°N
0° 10°E 20°E 30°E 40°E 50°E
−1.0
−1.5
−2.0
−2.5
−3.0
−1
−2
−3
N

Trachinus draco

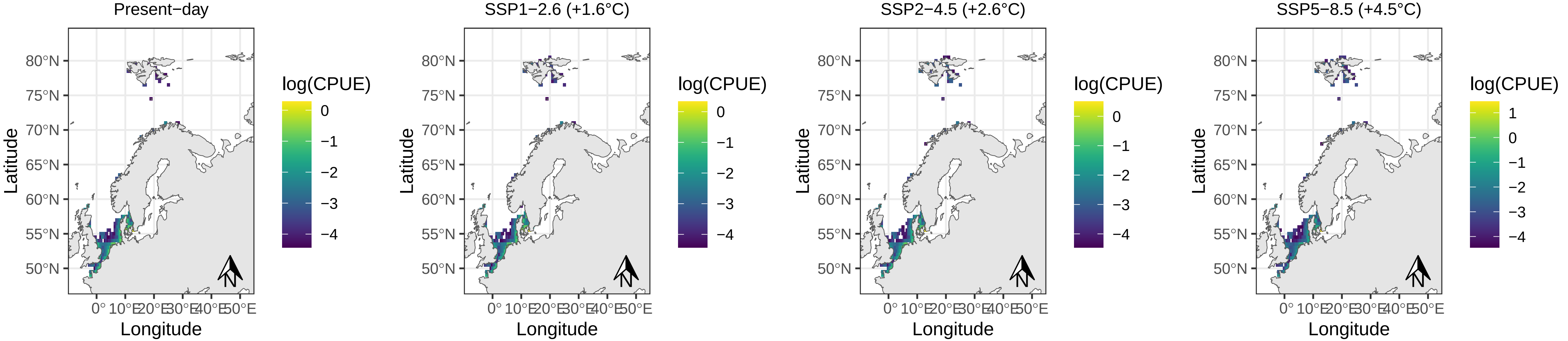

Trachurus trachurus

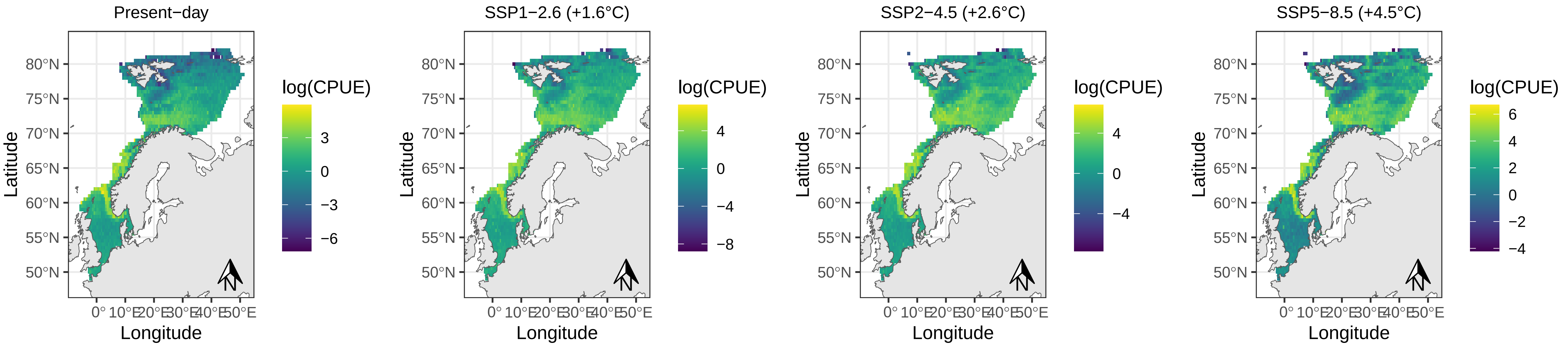

Triglops murrayi

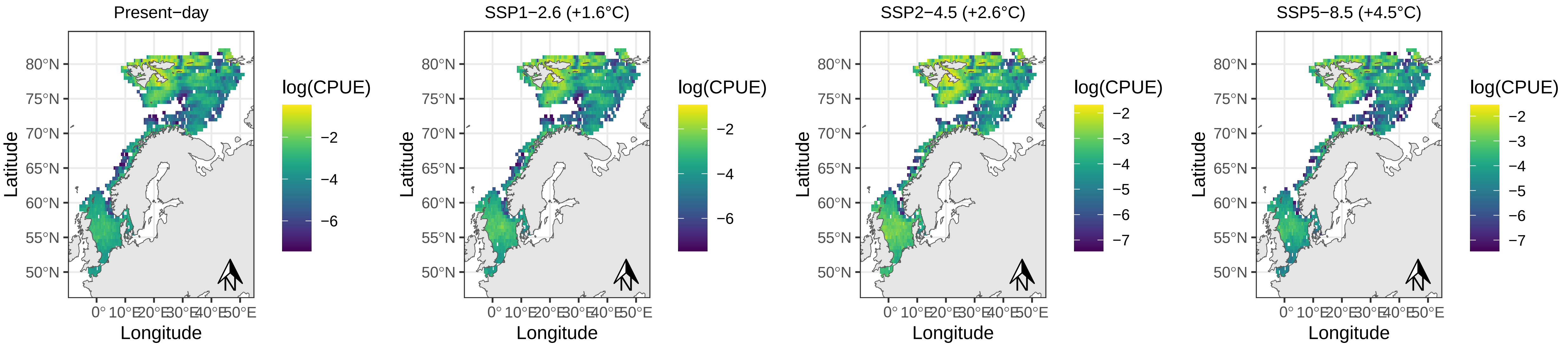

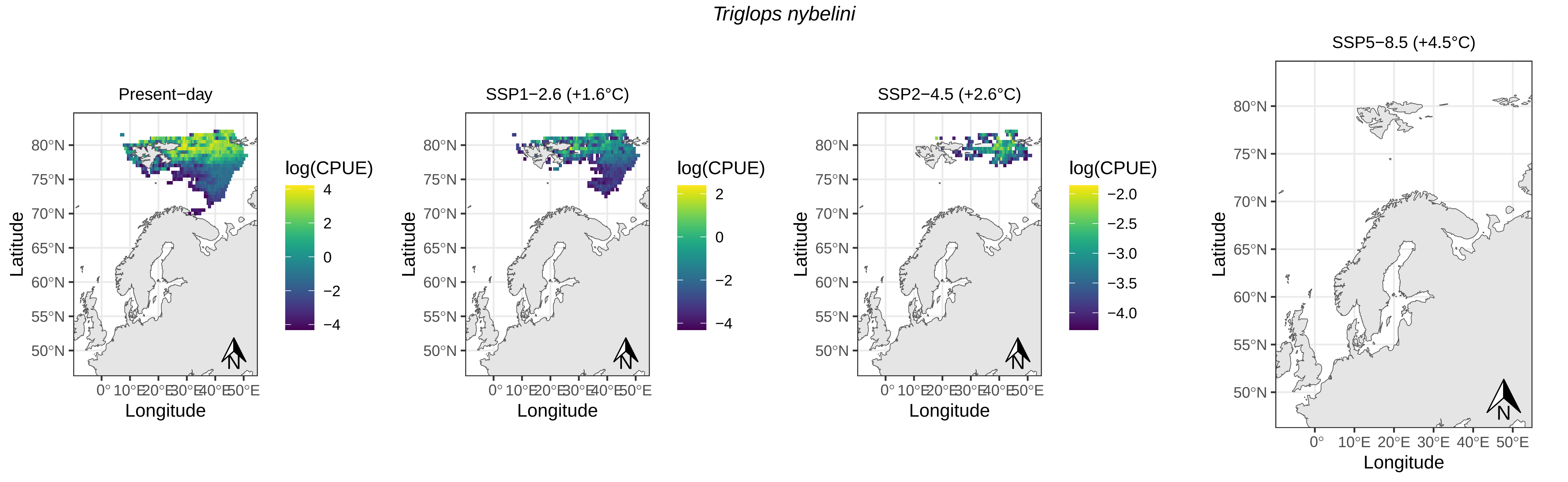

Triglops nybelini
Present−day
SSP1−2.6 (+1.6°C)
SSP2−4.5 (+2.6°C)
SSP5−8.5 (+4.5°C)
log(CPUE)
Latitude
Longitude
80°N
75°N
70°N
65°N
60°N
55°N
50°N
0°
10°E
20°E
30°E
40°E
50°E
N
4
2
0
−2
−4
−2.0
−2.5
−3.0
−3.5
−4.0

Trisopterus esmarkii

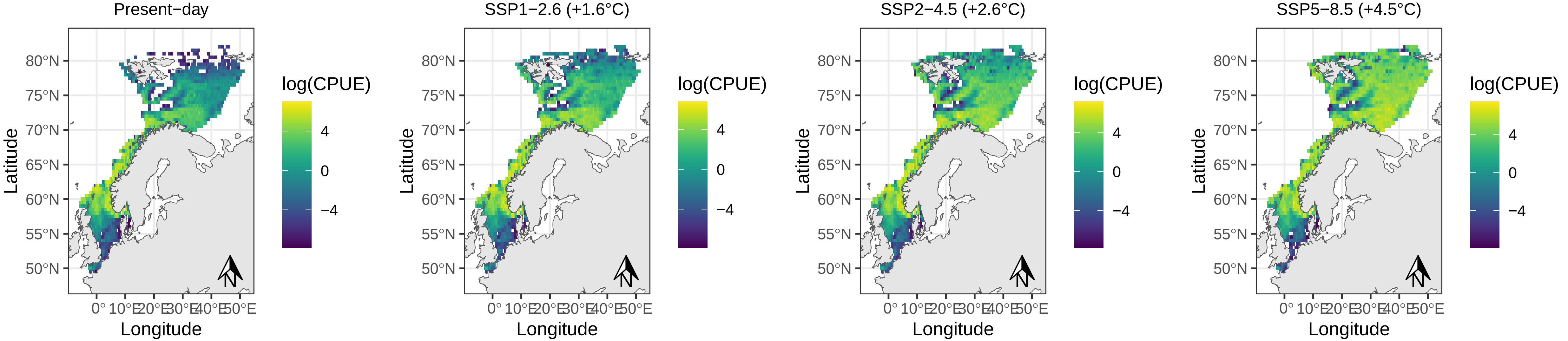

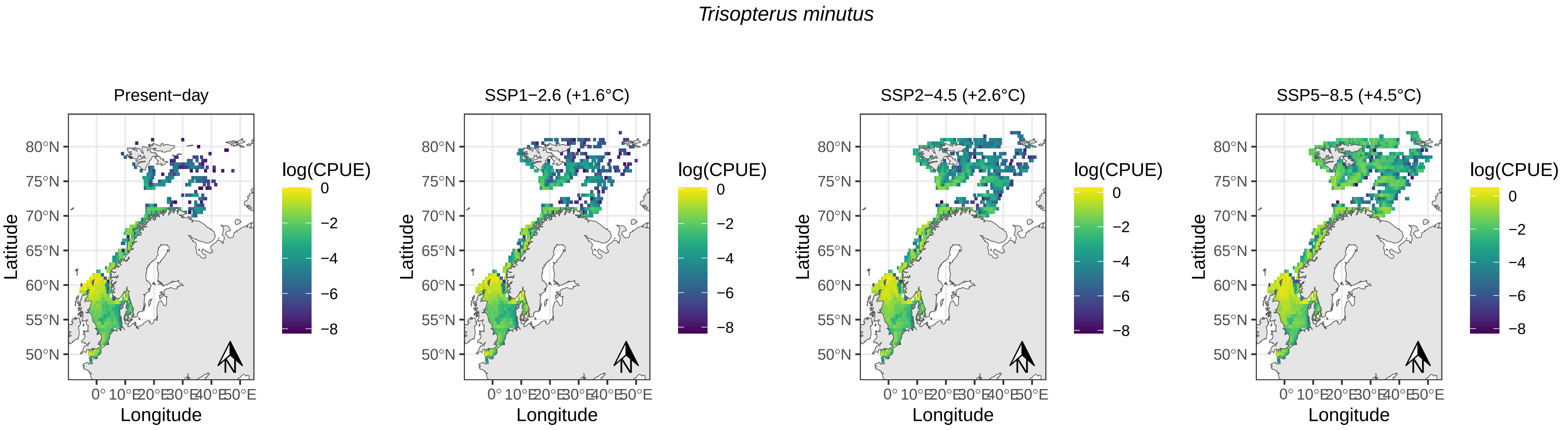
Trisopterus minutus
Present−day
SSP1−2.6 (+1.6°C)
SSP2−4.5 (+2.6°C)
SSP5−8.5 (+4.5°C)
Latitude
Longitude
80°N
75°N
70°N
65°N
60°N
55°N
50°N
0° 10°E 20°E 30°E 40°E 50°E
log(CPUE)
0
−2
−4
−6
−8
N
