# Supplementary material for: Future trends of marine fish biomass distributions from the North Sea to the Barents Sea
Source: Nat Commun. 2024 Jul 5;15:5637. doi: 10.1038/s41467-024-49911-9 (PMC11224334; doi:10.1038/s41467-024-49911-9)
Supplement: Supplementary file 9 — Reporting Summary [file 41467_2024_49911_MOESM9_ESM.pdf]

nature portfolio

Corresponding author(s): Cesc Gordó-Vilaseca

Last updated by author(s): 1/18/2024

Reporting Summary

Nature Portfolio wishes to improve the reproducibility of the work that we publish. This form provides structure for consistency and transparency in reporting. For further information on Nature Portfolio policies, see our Editorial Policies and the Editorial Policy Checklist.

Statistics

For all statistical analyses, confirm that the following items are present in the figure legend, table legend, main text, or Methods section.

n/a	Confirmed	
☐	☒	The exact sample size (*n*) for each experimental group/condition, given as a discrete number and unit of measurement
☒	☐	A statement on whether measurements were taken from distinct samples or whether the same sample was measured repeatedly
☐	☒	The statistical test(s) used AND whether they are one- or two-sided<br>*Only common tests should be described solely by name; describe more complex techniques in the Methods section.*
☐	☒	A description of all covariates tested
☒	☐	A description of any assumptions or corrections, such as tests of normality and adjustment for multiple comparisons
☐	☒	A full description of the statistical parameters including central tendency (e.g. means) or other basic estimates (e.g. regression coefficient) AND variation (e.g. standard deviation) or associated estimates of uncertainty (e.g. confidence intervals)
☐	☒	For null hypothesis testing, the test statistic (e.g. F, t, r) with confidence intervals, effect sizes, degrees of freedom and P value noted<br>*Give P values as exact values whenever suitable.*
☐	☒	For Bayesian analysis, information on the choice of priors and Markov chain Monte Carlo settings
☒	☐	For hierarchical and complex designs, identification of the appropriate level for tests and full reporting of outcomes
☐	☒	Estimates of effect sizes (e.g. Cohen's *d*, Pearson's *r*), indicating how they were calculated

Our web collection on statistics for biologists contains articles on many of the points above.

Software and code

Policy information about availability of computer code

Data collection	The data comes from the FishGlob database and the Norwegian Marine Data Center (NMDC)
Data analysis	All statistical analyses were performed using R (version 4.0.2, R Foundation for Statistical Computing, Vienna, Austria), and all code is available at GitHub (https://github.com/CescGV/JSDM-Barents-Norwegian-North). https://doi.org/DOI: 10.5281/zenodo.11395792

For manuscripts utilizing custom algorithms or software that are central to the research but not yet described in published literature, software must be made available to editors and reviewers. We strongly encourage code deposition in a community repository (e.g. GitHub). See the Nature Portfolio guidelines for submitting code & software for further information.

Data

Policy information about availability of data

All manuscripts must include a data availability statement. This statement should provide the following information, where applicable:

- Accession codes, unique identifiers, or web links for publicly available datasets
- A description of any restrictions on data availability
- For clinical datasets or third party data, please ensure that the statement adheres to our policy

The data used in this study was obtained from bottom trawling data collated within the FishGlob (Maureaud et al., 2023) (Accessible at https://github.com/AquaAuma/fishglob_data). The Norwegian Sea section of this data is no longer available in FishGlob, and needs to be directly asked to the Norwegian Marine Data Centre (https://metadata.nmdc.no/metadata-api/landingpage/f77112db062b5924d079a54b311260fb) . The data generated in this study, and used for figures 3, 6 and 7 is provided in the Source Data file. The trait database gathered is available at https://github.com/CescGV/JSDM-Barents-Norwegian-North. (Gordó-Vilaseca,

2024). World administrative boundaries polygons were obtained and are available from opendatasoft (Accessible at https://public.opendatasoft.com/explore/dataset/world-administrative-boundaries/information/).

Research involving human participants, their data, or biological material

Policy information about studies with human participants or human data. See also policy information about sex, gender (identity/presentation), and sexual orientation and race, ethnicity and racism.

Reporting on sex and gender	*Use the terms sex (biological attribute) and gender (shaped by social and cultural circumstances) carefully in order to avoid confusing both terms. Indicate if findings apply to only one sex or gender; describe whether sex and gender were considered in study design; whether sex and/or gender was determined based on self-reporting or assigned and methods used.*<br>*Provide in the source data disaggregated sex and gender data, where this information has been collected, and if consent has been obtained for sharing of individual-level data; provide overall numbers in this Reporting Summary. Please state if this information has not been collected.*<br>*Report sex- and gender-based analyses where performed, justify reasons for lack of sex- and gender-based analysis.*
Reporting on race, ethnicity, or other socially relevant groupings	*Please specify the socially constructed or socially relevant categorization variable(s) used in your manuscript and explain why they were used. Please note that such variables should not be used as proxies for other socially constructed/relevant variables (for example, race or ethnicity should not be used as a proxy for socioeconomic status).*<br>*Provide clear definitions of the relevant terms used, how they were provided (by the participants/respondents, the researchers, or third parties), and the method(s) used to classify people into the different categories (e.g. self-report, census or administrative data, social media data, etc.)*<br>*Please provide details about how you controlled for confounding variables in your analyses.*
Population characteristics	*Describe the covariate-relevant population characteristics of the human research participants (e.g. age, genotypic information, past and current diagnosis and treatment categories). If you filled out the behavioural & social sciences study design questions and have nothing to add here, write "See above."*
Recruitment	*Describe how participants were recruited. Outline any potential self-selection bias or other biases that may be present and how these are likely to impact results.*
Ethics oversight	*Identify the organization(s) that approved the study protocol.*

Note that full information on the approval of the study protocol must also be provided in the manuscript.

Field-specific reporting

Please select the one below that is the best fit for your research. If you are not sure, read the appropriate sections before making your selection.

☐ Life sciences ☐ Behavioural & social sciences ☒ Ecological, evolutionary & environmental sciences

For a reference copy of the document with all sections, see nature.com/documents/nr-reporting-summary-flat.pdf

Ecological, evolutionary & environmental sciences study design

All studies must disclose on these points even when the disclosure is negative.

Study description	We fitted a joint species distribution modelling framework to independent research survey data from the North Sea to the Barents Sea, and projected the model into the future. We analyse predicted changes in species distributions and biomass.
Research sample	We modelled 107 species including boreal and Arctic species from the North Sea to the Barents Sea, and we used community data from scientific research trawls to fit the models.
Sampling strategy	The sampling strategy differed across time and space, because we used a collection of different surveys and data collectors (IMR and ICES) as described in methods. The protocols and data particularities are available online:<br><br>- North Sea Data NS-IBTS (ICES-DATRAS) : https://ices-library.figshare.com/articles/report/SISP_10_Manual_for_the_North_Sea_International_Bottom_Trawl_Surveys/19051361<br><br>- Barents Sea data: This dataset is a collection of many coastal and offshore surveys: https://github.com/AquaAuma/FishGlob_data/tree/main/metadata_docs<br><br>- Norwegian Sea data: This dataset is a collection of many coastal and offshore surveys.<br>We visually checked adequate survey coverage, and show the spatial distribution of the surveys in Figure 1. Moreover, temporal homogeneity and randomness distribution is not key, because we extracted environmental data at the month when the sampling was conducted, and the number of samples per species is relatively high even for the rarest species included in the study area.
Data collection	The data was collected by the Institute of Marine Research (IMR) and the International Council for the exploration of the Sea (ICES), and is publicly available, as specified in the data availability statement. The data standardisation was done by the FISHGLOB team, and is not part of this study. In any case, the data collection was done using bottom trawling gear (GOV trawl) with 20 mm mesh size,

	and each haul catch was standardized by sampling effort (https://github.com/AquaAuma/FishGlob_data/tree/main/metadata_docs).
Timing and spatial scale	We used data from 2004 to 2022 covering the North Sea, the Norwegian Sea and the Barents Sea. All the processing and cleaning of the data is available through the Github repository (<br>Original FISHGLOB data: https://github.com/AquaAuma/FishGlob_data/tree<br>Our analysis: https://github.com/CescGV/JSDM-Barents-Norwegian-North).
Data exclusions	As specified in the methods, we eliminated very rare species that were sampled in less than 100 hauls, or in less than 10 years, and some other trawls (< 1%) were eliminated due to lack of environmental data. The final database included 16,345 unique hauls and included 107 fish species.
Reproducibility	To verify reproducibility, we made available all R code from the raw data to the final results.
Randomization	*Describe how samples/organisms/participants were allocated into groups. If allocation was not random, describe how covariates were controlled. If this is not relevant to your study, explain why.*
Blinding	*Describe the extent of blinding used during data acquisition and analysis. If blinding was not possible, describe why OR explain why blinding was not relevant to your study.*

Did the study involve field work? ☐ Yes ☒ No

Reporting for specific materials, systems and methods

We require information from authors about some types of materials, experimental systems and methods used in many studies. Here, indicate whether each material, system or method listed is relevant to your study. If you are not sure if a list item applies to your research, read the appropriate section before selecting a response.

Materials & experimental systems

n/a	Involved in the study
☒	☐ Antibodies
☒	☐ Eukaryotic cell lines
☒	☐ Palaeontology and archaeology
☒	☐ Animals and other organisms
☒	☐ Clinical data
☒	☐ Dual use research of concern
☒	☐ Plants

Methods

n/a	Involved in the study
☒	☐ ChIP-seq
☒	☐ Flow cytometry
☒	☐ MRI-based neuroimaging

Plants

Seed stocks	*Report on the source of all seed stocks or other plant material used. If applicable, state the seed stock centre and catalogue number. If plant specimens were collected from the field, describe the collection location, date and sampling procedures.*
Novel plant genotypes	*Describe the methods by which all novel plant genotypes were produced. This includes those generated by transgenic approaches, gene editing, chemical/radiation-based mutagenesis and hybridization. For transgenic lines, describe the transformation method, the number of independent lines analyzed and the generation upon which experiments were performed. For gene-edited lines, describe the editor used, the endogenous sequence targeted for editing, the targeting guide RNA sequence (if applicable) and how the editor was applied.*
Authentication	*Describe any authentication procedures for each seed stock used or novel genotype generated. Describe any experiments used to assess the effect of a mutation and, where applicable, how potential secondary effects (e.g. second site T-DNA insertions, mosiacism, off-target gene editing) were examined.*
